# Evidence of Gene Conversion in Genes Encoding the Gal/GalNac Lectin Complex of *Entamoeba*

**DOI:** 10.1371/journal.pntd.0001209

**Published:** 2011-06-28

**Authors:** Gareth D. Weedall, James Sherrington, Steve Paterson, Neil Hall

**Affiliations:** Institute of Integrative Biology, University of Liverpool, Liverpool, United Kingdom; Bose Institute, India

## Abstract

The human gut parasite *Entamoeba histolytica*, uses a lectin complex on its cell surface to bind to mucin and to ligands on the intestinal epithelia. Binding to mucin is necessary for colonisation and binding to intestinal epithelia for invasion, therefore blocking this binding may protect against amoebiasis. Acquired protective immunity raised against the lectin complex should create a selection pressure to change the amino acid sequence of lectin genes in order to avoid future detection. We present evidence that gene conversion has occurred in lineages leading to *E. histolytica* strain HM1:IMSS and *E. dispar* strain SAW760. This evolutionary mechanism generates diversity and could contribute to immune evasion by the parasites.

## Author Summary

Gene conversion is a process of recombination that can generate diversity among genes. Gene conversion occurs in some pathogenic species of protozoa to generate diversity among gene families encoding important antigens. The process may contribute to immune evasion by the parasites. Gene conversion, or indeed recombination of any kind, has not previously been demonstrated in human intestinal parasites of the genus *Entamoeba*. Here, we analysed genes encoding members of an important antigenic protein complex on the surface of *Entamoeba* parasites which is involved in invasion of the intestinal wall. Three gene families encode heavy-, light- and intermediate-subunits of the complex. We estimated genetic divergence between related genes from two species of *Entamoeba*, *E. histolytica* and *E. dispar*, and compared them to divergence among neighbouring genes and to the average across the whole genome, initially looking for evidence that the genes were evolving under positive selection. However, instead we saw patterns of genetic difference between some of the light- and intermediate-subunit genes indicating the action of gene conversion among members of these gene families. This indicates that recombinational mechanisms may play a part in the molecular evolution of these parasites.

## Introduction

*Entamoeba histolytica* causes a significant amount of death and disease, an annual estimate made in the 1980s indicated that 40,000–110,000 people died and 34–50 million people developed severe amoebiasis (dysentery or liver abscess) in 1981 [1]. Infection commonly results from the consumption of contaminated food and water and occurs predominantly among the poor in developing countries. Virulence is a rare outcome of infection, caused by the parasite attacking and crossing the gut wall. It can manifest as dysentery and in some cases as abscesses in the liver and other organs [2]. Most infected people clear their infection within a few months. The related species *Entamoeba dispar* is not generally believed to cause disease, but rather to live in the gut as a commensal.

A number of genes are implicated in *E. histolytica* virulence, among them the genes encoding the Gal/GalNAc lectin complex on the parasite's surface. The lectin complex binds galactose and the N-acetyl-D-galactosamine on mucin glycoproteins and on host cell surfaces and mediates both colonisation and contact-dependent cytotoxicity [2]. Anti-lectin immunoglobulin A is associated with protection from amoebiasis [3] and the Gal/GalNAc lectin heavy-chain subunit is a leading vaccine candidate [4]. Immune responses raised against Gal/GalNAc lectin components can protect against virulence [5], [6], although whether this protection is mediated by T-cells or by immuoglobulins is unclear [7], [8]. Immune mediated selection can be a powerful driver of diversity in parasite surface proteins [9]–.

Three components of the Gal/GalNAc lectin complex have been described: the heavy chain subunit, *hgl*; the light chain subunit, *lgl*; and the intermediate chain subunit, *igl*. Each is encoded by a gene family. Heavy- and light-chain lectin subunits are linked by disulphide bonds to form heterodimers. The heavy-chain subunit (*hgl*) genes contain a transmembrane domain linking a short cytoplasmic and a large extracellular, cysteine-rich domain which appears to mediate binding [12]. Heavy-chain subunit genes show 89–95% amino acid identity. The light-chain subunit is GPI-anchored to the cell membrane. It appears not to mediate adherence but may be associated with virulence, as downregulated virulence is associated with reduced *lgl* expression [13]. Light-chain subunit genes show more diversity than *hgl*, with 79–85% amino acid identity among proteins. The intermediate subunit (*igl*) is GPI-anchored and is non-covalently associated with the other members of the complex [2]. Members of both heavy- and light-chain lectin families have been identified in distantly related *Entamoeba* species, but *igl* genes have been identified only in *E. histolytica* and *E. dispar* [14], [15].

We reasoned that the difference in virulence between *E. histolytica* and *E. dispar* may in part be mediated by adaptive differences in the Gal/GalNAc lectin complex and that immune evasion may drive the evolution of lectin gene families. Therefore, it should be possible to see signatures of this adaptation in the patterns of sequence divergence between the species. However, rather than evidence for positive selection on single nucleotide mutations, we found evidence that gene conversion had occurred among members of the Gal/GalNAc lectin gene families.

## Materials and Methods

Data sets were initially defined using a text search for the term “lectin” on the amoebaDB website (www.amoebadb.org) [16]. Annotated lectin genes were used to query the database of predicted proteins for unannotated gene family members, using BLASTp. Results were assessed by sequence similarity and predicted gene length of the putative lectin. Searches indicated that *igl* was represented twice in *E. histolytica* and twice in *E. dispar*; *hgl* was represented five times in *E. histolytica* and twice in *E. dispar*; *lgl* was represented seven times in *E. histolytica* and six times in *E. dispar*.

The genomic context of each lectin gene was viewed and synteny with *E. dispar* assessed to define ‘positional orthology’ (i.e. orthology defined by being in the same genomic location). Syntenic genome regions were trimmed such that the lectin was bounded by at least one gene with a putative *E. dispar* orthologue on each side and aligned using MUSCLE [17] in the SEAVIEW sequence aligment editor [18]. Sequence alignments were checked and manually edited to ensure that nucleotide alignments across coding regions matched the corresponding amino acid alignments. For a small number of genes, the gene models were altered so that both species' gene models matched. The region surrounding the *lgl* gene EHI_049690 was orthologous to two scaffolds in *E. dispar*, the ends of which were almost identical when overlapped. A consensus was made of the region, in which the *E. dispar* genes EDI_071410 and EDI_071300 from scaffold DS548095 were merged with EDI_253210 and EDI_253220 from scaffold DS550857, respectively.

Divergence (*d*) was estimated across the region for a sliding window (window = 200 bp; step size = 1 bp), using R (Pi was calculated as the number of mismatches per window, rather than per non-gap-site, to reduce spikes caused by very short ‘windows’). In addition, the number of gaps in the alignment (an indicator of alignment quality) was calculated per window. The number of synonymous changes per synonymous site (*dS*), nonsynonymous changes per nonsynonymous site (*dN*) and their ratio (*dN*/*dS*) were estimated using codeml in the PAML software package [19]. Values were calculated using the maximum likelihood method of Goldman and Yang [20].

To investigate the possibility of gene conversion in the evolution of the lectin gene families, dS values between positional orthologues were compared to a genomic average in order to assess whether they were unusually high. All *E. histolytica* and *E. dispar* genes were downloaded from amoebaDB and grouped by orthMCL orthologue group (data from amoebaDB) [21]. To reduce the number of wrongly-aligned non-orthologous genes in the dataset only orthologue groups with exactly one gene from each species were analysed. 4770 orthologue pairs were aligned at the codon level, using PRANK [22] and dS estimated using codeml [19]. To further reduce the effect of misalignments of non-orthologous genes or incorrectly predicted gene models, pairs with extremely high overall divergence (the top 5% of pairwise branch length ‘t’ values from codeml) were removed from the analysis, leaving 4531 orthologue pairs. The frequency distribution of dS values for these gene pairs was plotted in R [23].

Phylogenetic trees were genearted for *igl* and *lgl* gene familes. Multiple alignments were generated for each family using MUSCLE [17]. Neighbour-joining phylogenies with bootstrap confidence values were generated using Seqboot, Protdist, Neighbour and Consense programs from the PHYLIP package (http://evolution.genetics.washington.edu/phylip.html), and displayed using the Dendroscope software [24]. A short, possibly truncated, *lgl* gene (EDI_023210) was removed from the analysis in order to increase the number of sites used to build the phylogeny.

Sequence similarity among members of the *igl* gene family was assessed. A dotplot was generated for using SEAVIEW [18], in which 40 bp windows were compared across all *igl* genes and plotted if they showed 100% identity. In addition, a sequence similarity plot was generated across a multiple alignments of the *igl* genes, using functions from seqinr and base packages of R [23], [25].

## Results

### Unusually high dS values in members of lectin gene families compared to neighbouring genes and the genome-wide average

Text and BLAST searches of predicted gene sets of *E. histolytica* and *E. dispar* in amoebaDB (www.amoebadb.org) defined the members of the heavy-, intermediate- and light-chain subunit gene families *hgl*, *igl* and *lgl*. The *hgl* family contained *E. histolytica* genes EHI_042370 , EHI_077500 , EHI_133900 , EHI_012270 and EHI_046650 and *E. dispar* genes EDI_213670 and EDI_123980. The *igl* family contained *E. histolytica* genes EHI_006980 and EHI_065330 and *E. dispar* genes EDI_276450 and EDI_244250. The *lgl* family contained *E. histolytica* genes EHI_049690, EHI_159870, EHI_058330, EHI_148790, EHI_183400, EHI_135690 and EHI_027800 and *E. dispar* genes EDI_071530, EDI_325130, EDI_131690, EDI_213170, EDI_352500 and EDI_023210.

**Figure 1:**
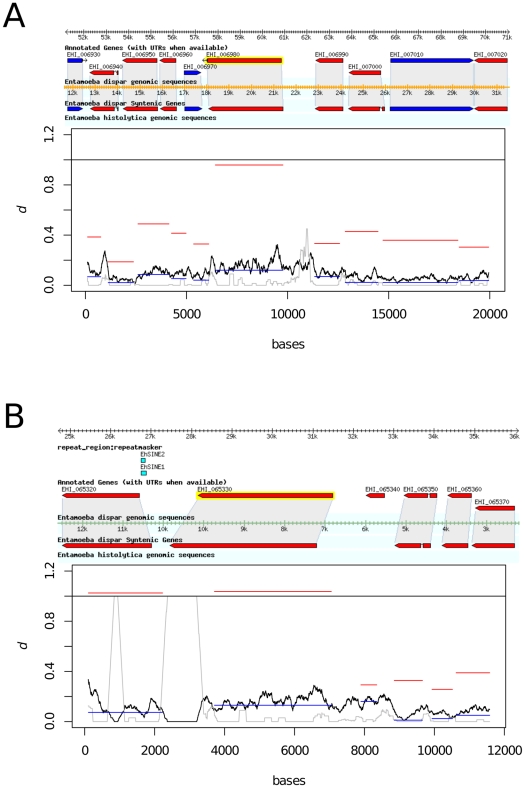
Divergence across chromosomal regions genes of *E. histolytica* and *E. dispar* containing intermediate-chain lecin genes. (A) *igl1* (EHI_006980:EDI_276450); (B) *igl2* (EHI_065330:EDI_244250). Divergence (*d*) for a 200 bp sliding window is shown (black line), and dN (blue bars) and dS (red bars) are plotted for putative coding regions. The grey line shows the proportion of gapped positions in each window, an indication of poor alignment quality. Both lectin genes show dS values near to 1, notably higher than for surrounding genes, except gene EHI_065320 which also shows dS>1.

**Figure 2:**
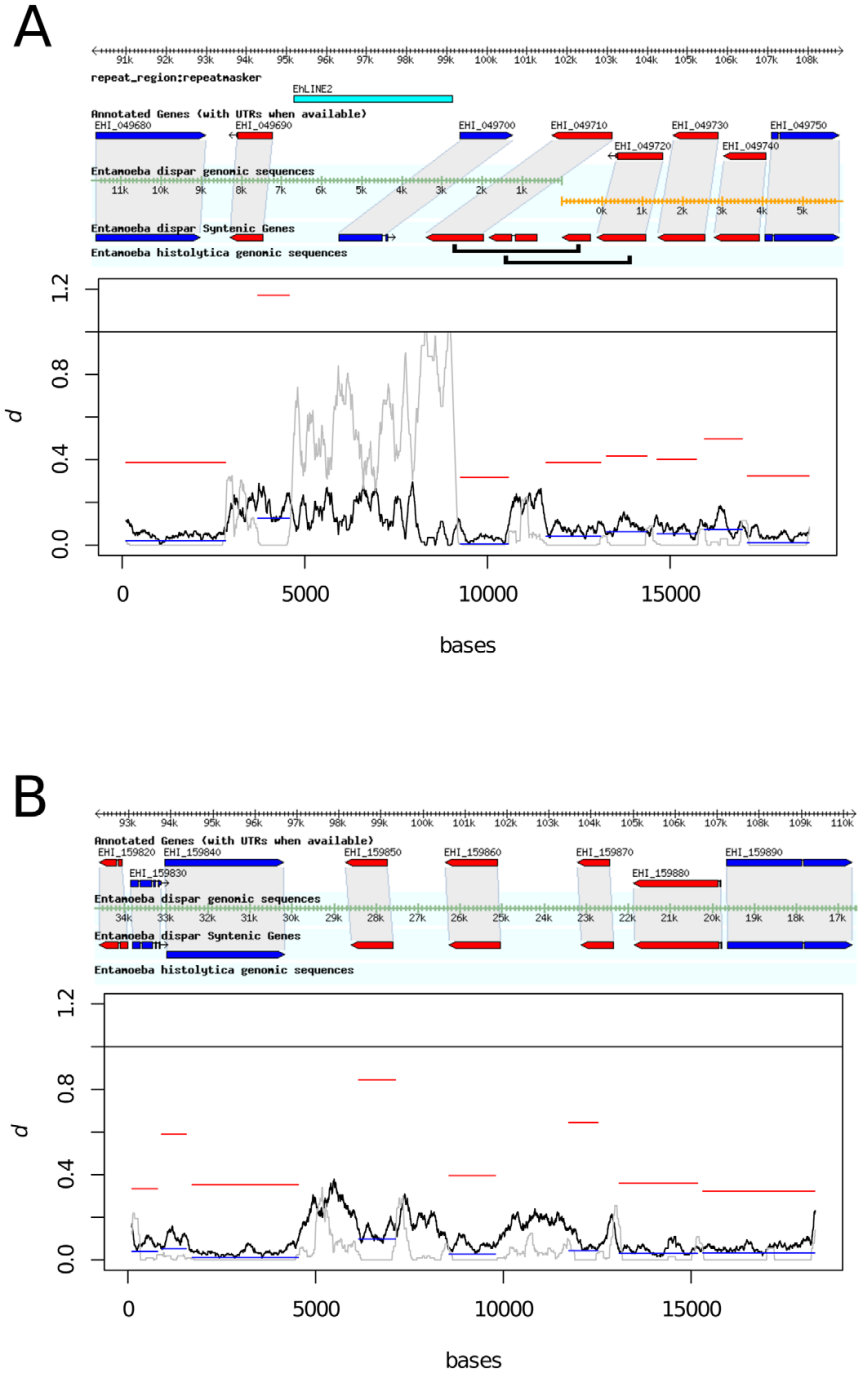
Divergence across chromosomal regions of *E. histolytica* and *E. dispar* containing light-chain lectin genes. (A) *lgl* (EHI_049690:EDI_071530); (B) *lgl* (EHI_159870:EDI_325130). Divergence (*d*) for a 200 bp sliding window is shown (black line), and dN (blue bars) and dS (red bars) are plotted for putative coding regions. The grey line shows the proportion of gapped positions in each window, an indication of poor alignment quality. Light-chain lectin gene EHI_049690 shows dS>1, it occurs adjacent to a LINE2 sequence in *E. histolytica*. Black bars indicate putative genes merged by overlapping the two *E. dispar* scaffolds.

Visual inspection of aligned scaffolds of *E. histolytica* and *E. dispar* in amoebaDB identified six pairs of genes which could be identified as orthologous by surrounding synteny (Figures 1, 2 and Figure S1). These orthologous pairs were: *hgl* genes EHI_012270:EDI_213670 and EHI_046650:EDI_123980; *igl* genes EHI_006980:EDI_276450 and EHI_065330:EDI_244250; and *lgl* genes EHI_049690:EDI_071530 and EHI_159870:EDI_325130. Genomic regions encompassing these genes were aligned (Figures S2, S3, S4, S5, S6, S7) and interspecific diversity estimated across them, under the hypothesis that positive selection in lectin genes should produce a peak of divergence compared to surrounding genes not under such selection. Of the six genes analysed, the two *igl* genes and an *lgl* gene (EHI_049690) showed slightly elevated divergence relative to their neighbours. However, on testing for positive selection driving divergence between these genes, the number of synonymous differences per synonymous site (dS) between orthologous genes was particularly high (Figure 1, Figure 2), with dS>1 for two of the genes. We would expect, under positive selection, that the number of nonsynonymous differences per nonsynonymous site (dN) would be high, but that dS would not differ from dS in neighbouring genes. The pattern we observed for the *igl* genes EHI_006980:EDI_276450 and EHI_065330:EDI_244250 and *lgl* genes EHI_049690:EDI_071530, where dS was notably higher than for neighbouring genes, suggested a mechanism generating diversity other than positive selection on nonsynonymous mutations. The pattern indicated gene conversion, in which gene regions are changed to match a paralogue that may be more divergent than a gene's true orthologue, hence the elevated dS.

**Figure 3:**
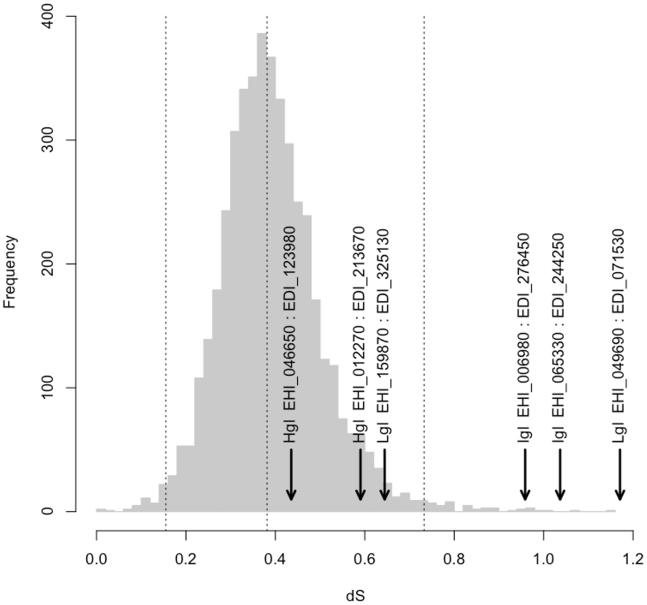
Divergence at synonymous sites (dS) is unusually high between *igl* and *lgl* positional orthologues. The frequency distribution of pairwise dS between 4531 putative orthologue pairs is plotted (grey bars). Dashed lines indicate 1st, 50th and 99th centile values. Pairwise dS values between *hgl*, *igl* and *lgl* positional orthologues are indicated. Both *igl* pairs and one *lgl* pair fall within the top 1% of dS values.

To confirm that dS values between positional orthologues were unusually high, they were compared to a genomic average (Figure 3). The median dS between orthologous genes of *E. histolytica* and *E. dispar* was 0.38, and 99% of dS values were below 0.73. The *igl* genes EHI_006980:EDI_276450 and EHI_065330:EDI_244250 and the *lgl* genes EHI_049690:EDI_071530 all had dS values in the top 1%, strongly supporting the hypothesis that gene conversion has occurred among these genes.

**Figure 4:**
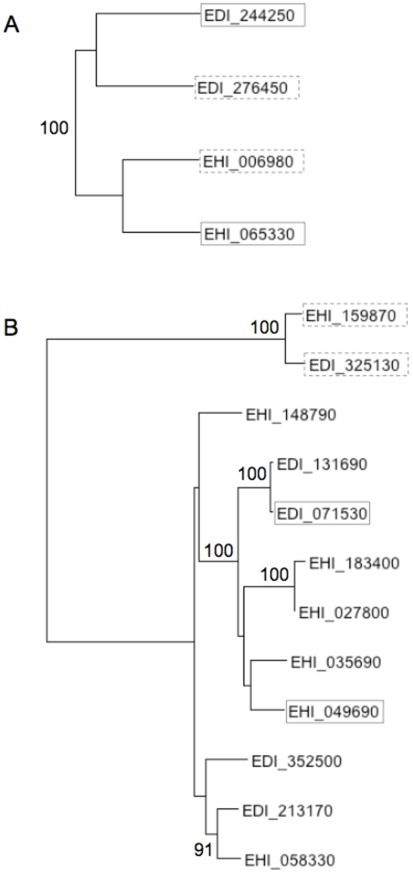
Phylogenies of *igl* and *lgl* gene familes. Amino acid neighbour-joining trees with % bootstrap support shown where values are >70%. Grey solid and dashed boxed indicate positional orthologue pairs. (A) The *igl* tree clearly clusters paralogues. (B) The *lgl* tree clusters the orthologue pair EHI_159870:EDI_325130, which do not show unusually high dS (Figure 2B, Figure 3). The tree is not well resolved in the clade containing positional orthologues EHI_049690 and EDI_071530.

Phylogenies of multiple alignments of *igl* and *lgl* gene familes (Figures S8, S9) further supported gene conversion between *igl* genes. The expected pattern, given no gene conversion, is that orthologues should cluster together, yet the tree shows strong bootstrap support for the clustering of paralogous *igl* genes (Figure 4A). In the *lgl* family, the orthologues EHI_159870 and EDI_325130 cluster together and are quite divergent from other *lgl* genes (Figure 4B). This pair showed no evidence for gene conversion in Figure 2B and Figure 3. The other pair, EHI_049690 and EDI_071530, occurred in a poorly resolved part of the tree. Amino acid divergence between EDI_071530 and EHI_049690 is 25.2% (dS = 1.17), slightly higher than between EDI_071530 and EHI_035690 (24.8%, dS = 0.98). Between EHI_049690 and EHI_035690, dS was higher (dS = 0.73) than the average between *E. histolytica* and *E. dispar* (median dS = 0.38), suggesting that they did not arise from a recent duplication, yet they are more closely related to each other than either is to EDI_071530. Without more information on positional orthology it is difficult to draw any conclusion about the evolutionary history of the genes, other than noting the unexpectedly high synonymous divergence.

### Sequence similarity across *igl* genes

**Figure 5:**
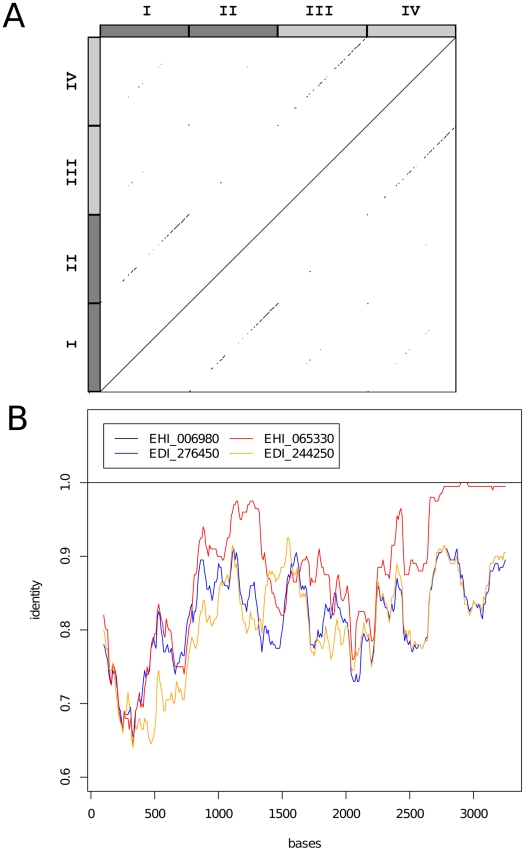
Sequence similarity among intermediate-chain lectin subunit genes. (A) Dotplot indicating identical 40 bp windows among all *igl* genes. Dark grey boxes I and II represent *E. histolytica* genes EHI_006980, EHI_065330; light grey boxes III and IV represent *E. dispar* genes EDI_276450 and EDI_244250. (B) Sequence identity (to gene EHI_006980) calculated for a 200 bp window, moving in 10 bp increments across a multiple alignment of *igl* genes.

To further explore and locate possible gene conversion among the *igl* genes, we assessed sequence similarity among members of the gene family across the length of the genes. We generated a dotplot showing identical 40 bp windows between genes and calculated sequence similarity across a multiple alignment (Figure S10). Sequence similarity between paralogues is particularly notable at the 3′ end and between bases 1000 and 1500 (Figure 5), covering two of several putative growth factor receptor domains predicted in the genes.

## Discussion

We set out to determine whether members of the Gal/GalNAc lectin complex have evolved under positive selection. However, we were unable to test this due to an unusual pattern of apparent synonymous divergence in some lectin genes. These unusually high values (in some cases >1 synonymous mutation per synonymous site) indicated that the genes of *E. histolytica* and *E. dispar* were not in fact orthologous, despite occurring in syntenic genome regions. We showed that dS significantly exceeded the genomic average for *igl1*, *igl2* and an *lgl* (EHI_049690:EDI_071530). Phylogenetic analysis supported the hypothesis that gene conversion had made paralogous *igl* genes more similar than orthologous *igl* genes. In these intermediate-chain lectin genes we saw regions, most notably in the central region and at the 3′ end, where paralogous sequences were highly similar to each other: a pattern expected if gene conversion has occurred.

While it should be noted that the lower sequence coverage of *E. dispar*, compared to *E. histolytica*, could result in more errors in its sequence, such errors would inflate the estimated divergence (*d*) for all genes. Thus, the unusually high dS in lectin genes relative to their neighbours and the the genomic average would not be affected. For the light-chain lectins it is difficult to infer gene conversion from the phylogeny, due to the existence of more gene family members with uncertain orthology. The phylogeny is complicated by the possibility both of incomplete sampling of genes and of recent gene duplications within a species, and by the lack of positional orthology information to compare to the tree. Further sequencing and analysis may help to clarify the picture. In contrast, support for gene conversion among the *igl* genes was stronger.

Some genes near to lectin genes also showed similarly high dS values. A putative heat shock protein 70 gene (EHI_065320) occurs adjacent to *Ehigl2* and shows dS>1. A BLASTp search against *E. histolytica* and *E. dispar* protein sequences (data not shown) shows stretches of sequence identity with other genes (EHI_006560 and EDI_169350), suggesting a similar process may have occurred among these genes. A gene encoding an unknown product (EHI_159850) near to a light chain lectin gene (EHI_159870) also shows high dS and a BLASTp search (data not shown) showed high sequence identity with another *E. dispar* hypothetical protein (EDI_285400). A notable feature of several of the lectin genes is their close proximity to repetitive elements. It is possible that repetitive elements, by creating regions of sequence homology adjacent to non-homologous genes, might promote gene conversion.

Gene conversion is a process of non-reciprocal homologous recombination whereby one region of the genome is ‘converted’ to become identical to another region. Since sequence homology is required, it occurs preferentially among members of multi-gene families. Our results indicate that homologous recombination can occur in *E. histolytica*. This is significant since the same mechanism is required for sexual reproduction, which has not been demonstrated to occur in *E. histolytica* despite its genome encoding the necessary genes [26], [27]. Our results do not prove that sexual reproduction (genetic exchange between individuals) occurs.

Although over the long term gene conversion will homogenise sequence, by displacing diversity accumulated during the divergence of paralogous sequences, in the short term it may act as a generator of diversity by creating new haplotypes which may exist alongside ancestral haplotypes in a population. Gene conversion is a mechanism utilised by a number of eukaryotic and prokaryotic pathogen species to generate antigenic diversity within large gene familes and evade the immune response of the host [28], [29]. It has also been identified in a number of smaller gene families in *Plasmodium falciparum* [30]–[32] and in a large family in *Trichomonas vaginalis* [33]. The Gal/GalNAc lectin does appear to be a target of protective immunity [3] so it is possible that gene conversion enables the generation of genetic diversity for immune evasion. However, the specific target and vaccine candidate molecule ‘lecA’, a part of the heavy-chain subunit gene EHI_133900, does not have a clear orthologue in *E. dispar*, so evidence of gene conversion could not be seen in this gene. The *hgl* genes that were tested did not show clear evidence for gene conversion. It will be interesting to discover whether variant genes arising from gene conversion segregate within *E. histolytica* and *E. dispar* populations, to assess the importance of this mechanism in these species.

Genes analysed in this manuscript were: putative heavy chain lectin genes EHI_042370, EHI_077500, EHI_133900, EHI_012270 and EHI_046650 of *E. histolytica* and EDI_213670 and EDI_123980 of *E. dispar*; putative intermediate chain lectin genes EHI_006980 and EHI_065330 of *E. histolytica* and EDI_276450 and EDI_244250 of *E. dispar*; and light chain lectin genes EHI_049690, EHI_159870, EHI_058330, EHI_148790, EHI_183400, EHI_035690 and EHI_027800 of *E. histolytica* and EDI_071530, EDI_325130, EDI_131690, EDI_213170, EDI_352500 and EDI_023210 of *E. dispar*.

## Supporting Information

Figure S1 **Divergence across chromosomal regions genes of** ***E. histolytica*** **and** ***E. dispar*** **containing heavy-chain lecin genes.** (A) *hgl* (EHI_012270:EDI_213670); (B) *hgl* (EHI_046650:EDI_123980). Divergence (*d*) for a 200 bp sliding window is shown (black line), and dN (blue bars) and dS (red bars) are plotted for putative coding regions. The grey line shows the proportion of gapped positions in each window, an indication of poor alignment quality. dS is not notably greater for lectin genes than for surrounding genes. In panel B, bases between position 8000–14,000 could not be aligned so were replaced with Ns (hence the apparent sequence identity in the plot).
(PDF)

Figure S2 **Nucleotide alignment of orthologous genome regions of** ***E. histolytica*** **and** ***E. dispar*** **used to estimate inter-specific divergence around the heavy chain lectin orthologues EHI_012270 and EDI_213670.**
(PDF)

Figure S3 **Nucleotide alignment of orthologous genome regions of** ***E. histolytica*** **and** ***E. dispar*** **used to estimate inter-specific divergence around the heavy chain lectin orthologues EHI_046650 and EDI_123980.**
(PDF)

Figure S4 **Nucleotide alignment of orthologous genome regions of** ***E. histolytica*** **and** ***E. dispar*** **used to estimate inter-specific divergence around the intermediate chain lectin orthologues EHI_006980 and EDI_276450.**
(PDF)

Figure S5 **Nucleotide alignment of orthologous genome regions of** ***E. histolytica*** **and** ***E. dispar*** **used to estimate inter-specific divergence around the intermediate chain lectin orthologues EHI_065330 and EDI_244250.**
(PDF)

Figure S6 **Nucleotide alignment of orthologous genome regions of** ***E. histolytica*** **and** ***E. dispar*** **used to estimate inter-specific divergence around the light chain lectin orthologues EHI_049690 and EDI_071530.**
(PDF)

Figure S7 **Nucleotide alignment of orthologous genome regions of** ***E. histolytica*** **and** ***E. dispar*** **used to estimate inter-specific divergence around the light chain lectin orthologues EHI_159870 and EDI_325130.**
(PDF)

Figure S8 **Amino acid multiple alignment of intermediate chain lectin (** ***igl*** **) gene family members from** ***E. histolytica*** **and** ***E. dispar*** **, used to generate a gene phylogeny.**
(PDF)

Figure S9 **Amino acid multiple alignment of light chain lectin (** ***lgl*** **) gene family members from** ***E. histolytica*** **and** ***E. dispar*** **, used to generate a gene phylogeny.**
(PDF)

Figure S10 **Nucleotide multiple alignments of intermediate chain (** ***igl*** **) lectin gene family members from** ***E. histolytica*** **and** ***E. dispar*** **, used for sequence similarity plot.**
(PDF)
