# Supplementary figures and images for: Evidence of Gene Conversion in Genes Encoding the Gal/GalNac Lectin Complex of Entamoeba
Source: PLoS Negl Trop Dis. 2011 Jun 28;5(6):e1209. doi: 10.1371/journal.pntd.0001209 (PMC3125142; doi:10.1371/journal.pntd.0001209)

A

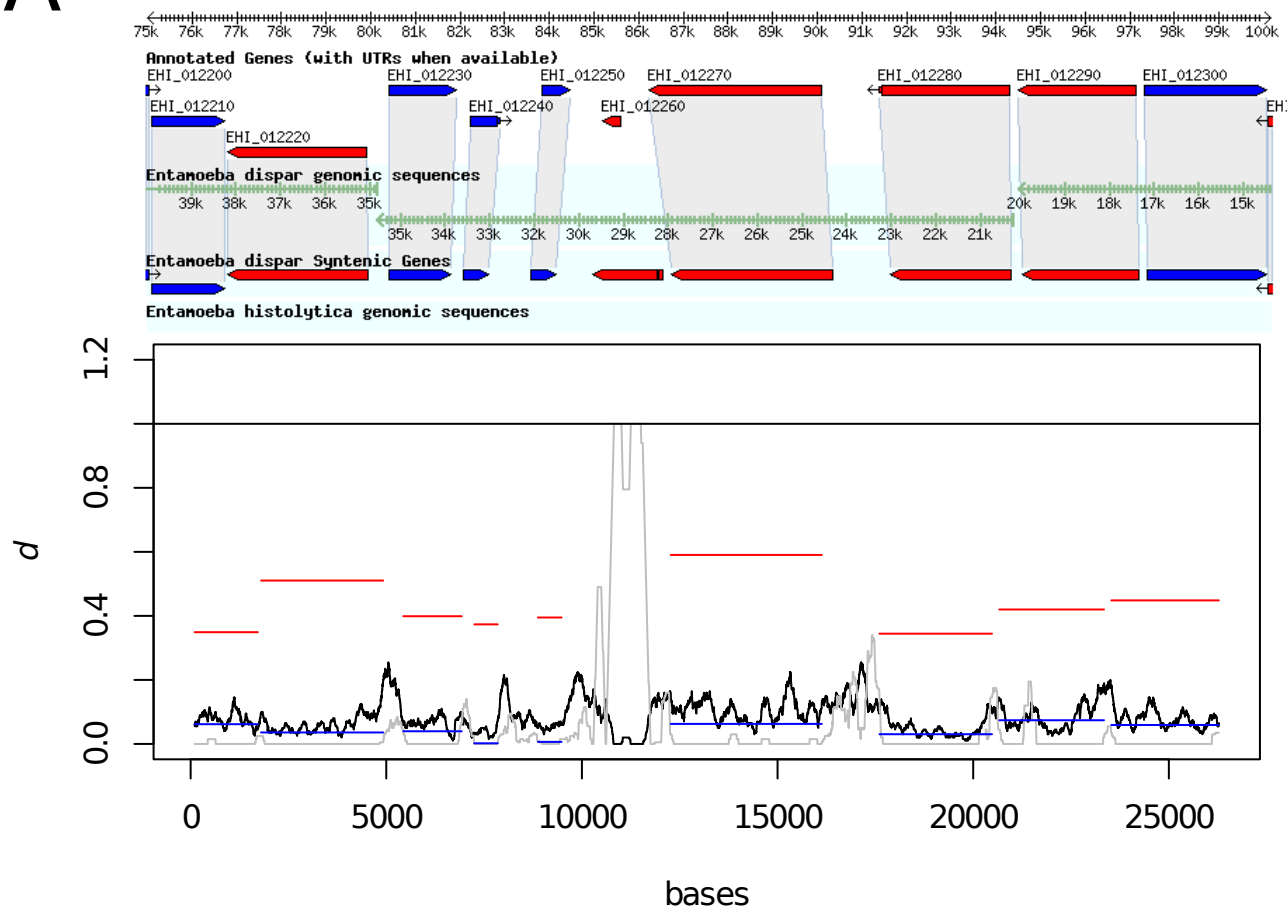

B

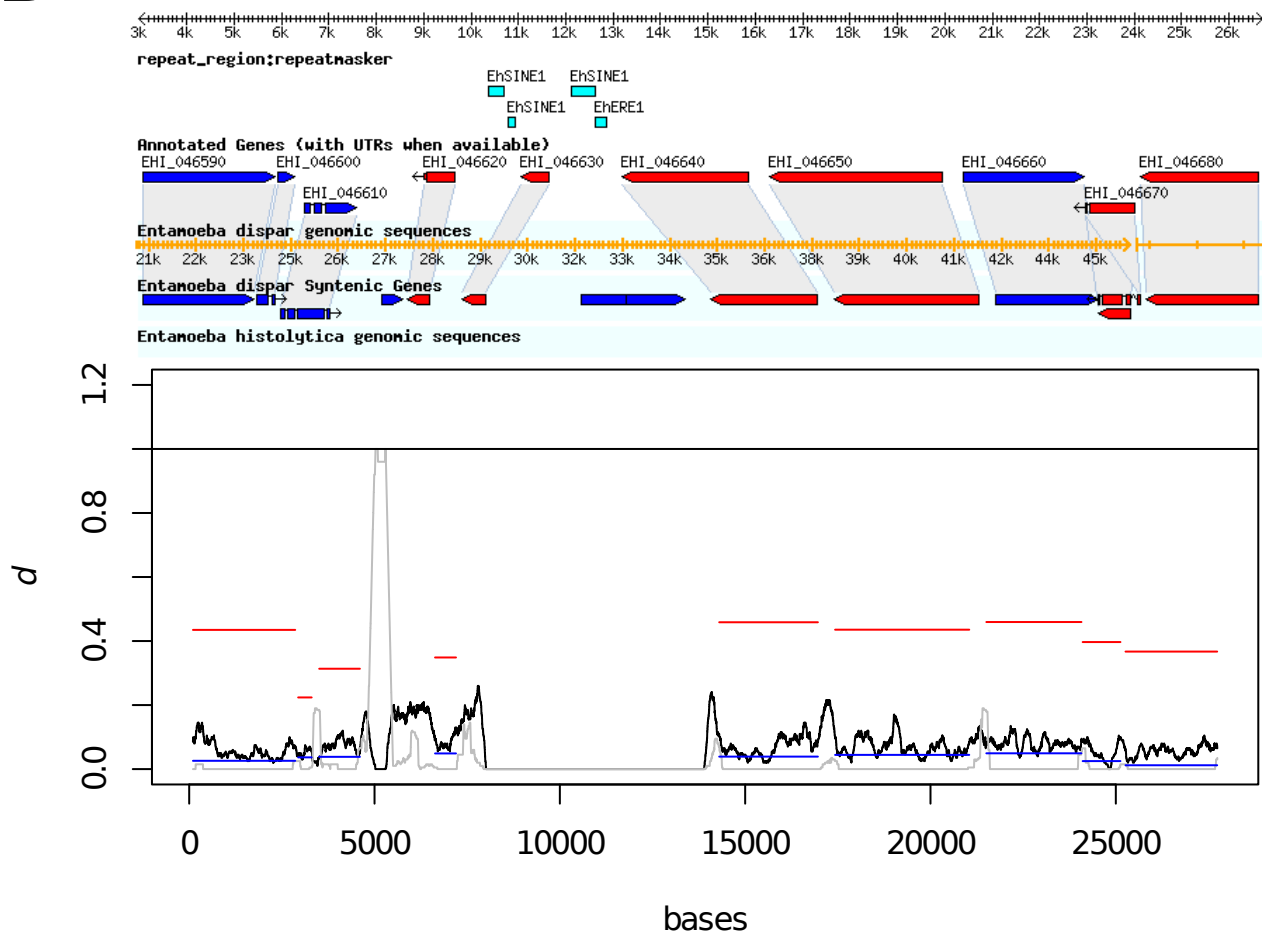

Supplement: Figure S1 — Divergence across chromosomal regions genes of E. histolytica and E. dispar containing heavy-chain lecin genes. (A) hgl (EHI_012270:EDI_213670); (B) hgl (EHI_046650:EDI_123980). Divergence (d) for a 200 bp sliding window is shown (black line), and dN (blue bars) and dS (red bars) are plotted for putative coding regions. The grey line shows the proportion of gapped positions in each window, an indication of poor alignment quality. dS is not notably greater for lectin genes than for surrounding genes. In panel B, bases between position 8000–14,000 could not be aligned so were replaced with Ns (hence the apparent sequence identity in the plot). (PDF) [file pntd.0001209.s001.pdf]
