# Supplementary material for: Evidence of Gene Conversion in Genes Encoding the Gal/GalNac Lectin Complex of Entamoeba
Source: PLoS Negl Trop Dis. 2011 Jun 28;5(6):e1209. doi: 10.1371/journal.pntd.0001209 (PMC3125142; doi:10.1371/journal.pntd.0001209)
Supplement: Figure S2 — Nucleotide alignment of orthologous genome regions of E. histolytica and E. dispar used to estimate inter-specific divergence around the heavy chain lectin orthologues EHI_012270 and EDI_213670. (PDF) [file pntd.0001209.s002.pdf]

```
Alignment: /Users/garethweedall/Desktop/hgl1.fas
Seaview [blocks=70 fontsize=10 A4] on Fri Nov  5 15:20:41 2010

                               1
DS571148_75000-100159          GTTAGGCGGTTATTCAATGTAAtactataatcaataataaagtttattatttaatttattataatt
DS548107_14778-40922_rc        GTTAGGTGGTCATTCAATGTAAtactataaaaaataataaaatttattatttaatttattataaat

                              67
DS571148_75000-100159          tttttaaatagaaaaaatagtgtttctacattcaATGACAGAAGGAGTAAAACGGGTTTTACCTTC
DS548107_14778-40922_rc        tttttaaatagaaaaaatattgttttgacattcaATGACAGAAGGAGTAAAACGGGTTTTACCTTC

                             133
DS571148_75000-100159          ATTTAATATTTTACCAAAAAAAATGAGTCATGACCAAAAACGGTTTCAAATATTTGAAAATTATAA
DS548107_14778-40922_rc        ATTTAATATTTTACCCAAAAAAATGAGTCATGACCAAAAACGGTTTCAAGTATTTGAAAGTTATAA

                             199
DS571148_75000-100159          TGAAAAAAAAAAGGTAATAGAAGCAATTAAACAACCAGTTAATGAATTAATTGAGCAAGTAGAAAA
DS548107_14778-40922_rc        TGAAAAAAAAGTGGTAATAGAAACAACTAAACAACCAGTTAATGAATTAATTGAGCAAATAGAAAA

                             265
DS571148_75000-100159          TAAAGAGTTAAAGGCAAAACTTTCAAGAGAAGTAAAAAGTCTTATTGATGAGTTAATACAACAAAT
DS548107_14778-40922_rc        TAAAGAACTAAAGACAAAACTCTCAAAAGAAGTAAAAAATCTTATTGATGAATTAACACAAGAAAT

                             331
DS571148_75000-100159          CAGTGATCTTAAAAATCAAATAAAAGTATTAGAAAGAAAATATGAAATATATAATATTCAAGAACA
DS548107_14778-40922_rc        CTGTGATCTTAAAAATCAAATAAAAGTATTAGAAAGAAAATGTGAAATATATAACATACAAGAACA

                             397
DS571148_75000-100159          AAATACTGTTATTAGAAGTGAGTCACAAAATGATAACATTCCAAAATTAACAGATACAAAAGATAA
DS548107_14778-40922_rc        AAATAATCTTATTAGAAGTCAGTCACAAAATGATGAAATTACAAAACTAACAGATACAAAAGGTAA

                             463
DS571148_75000-100159          TAAAGAAATTATTCCAATGGAATGTGAAACAAATACAACAGATATTTTATTTGAAGGATATAAAGA
DS548107_14778-40922_rc        TAAAGAAATTATTTCAATGGAATGTGAAACAAATACAACAAATATTTTATTAGAAGGATATAAAGA

                             529
DS571148_75000-100159          TACTCCTCAAGTAGATACAGGTTTAATAGAAATAAAGAATAAAAGAAGAATACTTGCTGATAAACA
DS548107_14778-40922_rc        TACTCCT---GTAGATTCAAATCTATTAGAAATACAAAATAAAAGAAGAGAAATTGCTGATAAACA

                             595
DS571148_75000-100159          AATTAATGAAGTAGCAAAATGTAGATGGATTGAACAAATGTTTGGAAAAGGATGGGATATGATTGA
DS548107_14778-40922_rc        AATTAATGAAGTAGCAAAATGTAAATGGATTGAACAAATGTTTGGAAAAGGGTGGGATATGATTGA

                             661
DS571148_75000-100159          AGTAAAAAAAGATTATCTTTTGACAATACAAAACGAAATAATATCACATTTAGAAATTGAGATATC
DS548107_14778-40922_rc        AGTAAAAGAAGATTATCTGTTGAGAATACAAAACGAAATAATAACACAATTAGAATTTGAAATATC

                             727
DS571148_75000-100159          ATTATGTATTGTTATATTTGATGGTTATGGTAATGTAATTGGATTTGAAATAGAAGGGATGAATAA
DS548107_14778-40922_rc        AATATGTATTGTTATATTTGATGGATACGGTAATGTTATTGGATTTGAAATAGAGGGAATGAATAA

                             793
DS571148_75000-100159          ACTTAATTATTATAATGAAATACATGATGTAGAATGTACTTTATTTTCTCTTTATGATGAAACAAA
DS548107_14778-40922_rc        ACTTAATTATTATAATGAAATACATGATGTACAATGTACATTATTTTCTCTTTATGATGAAACAAA

                             859
DS571148_75000-100159          AGAAGAAATGAATTGTAAAATGTATCGATTAAATGAAGATAATAACATTATTATATTATTTAATAA
DS548107_14778-40922_rc        AGAAGAAATGAATTGTAAAAAGTATCAATTAAATGAAGATAATAATATTATTATATTATTTAATAA

                             925
DS571148_75000-100159          AAGTGGAGAAACTATATGTAAAGTTAATGTGAATGGAAGAGATATTATTAAAATAGGTGTTCCTAT
DS548107_14778-40922_rc        AAGTGGAGAAACTATATGTAAAGTTAATGTAAATGGAAAAGATATTATTAAAATAGGACTTCCTAT

                             991
DS571148_75000-100159          AATACAATTAAGTTCAATTGATATTGAAACAATTAATATTGAGGATGATACTGTTTATAATTCATT
DS548107_14778-40922_rc        AATACAATTAAGTTCAATTGATATTGGAACGATAGATATTAAGGATAATAATGTTTATAAATCATT

                            1057
DS571148_75000-100159          TATAAAATTTATTTCACATCAAGACATAGAAAATGAAATTGATATTCAAGGAATGTTATGTTTTTA
DS548107_14778-40922_rc        TATAAAATTTATTTCACATCAAGATAAAGAAAATGAAATTGATATTCAAGGAATTTTATGCTTTTA

                            1123
DS571148_75000-100159          TAAAAAGAATACATTTAATGGAAAAGAAAATGAAGATTTTATAATAAATACATTTAAGAAAGAAAA
DS548107_14778-40922_rc        TAAAAATAATACATTGAACAAAAAAGAAAATGAGGATTTTATTACAAGTTCTTTTAATAAAGAAAA

                            1189
DS571148_75000-100159          AGATAAACAGAATTGTATTGATGATGAACAACAAGTAGATGATATTTTAATTACTATTATTCAAGA
DS548107_14778-40922_rc        AAATAGACATAATGATATTGATGATGAACAAGAAGTAGATGATATTTTAATTACTATTATTCAAGA
```

```
                            1255
DS571148_75000-100159       AATGTCAGAAAAAAGTGAATGGGAAATGATATTTTCATCAAAAACAATGAAAGAAACAAATAAAAA
DS548107_14778-40922_rc     AATGTCAGAAAAAAATGAATGGGAAATGATATTTTCATCAAAAACAATGAAAGAAACAAATAAAAA

                            1321
DS571148_75000-100159       TTTTAATGAAATAATAATAAATCAAAAGAGTTTGTTAATTAATTTACAAACAAAAGAAAAAGAATG
DS548107_14778-40922_rc     TTTTAATACAAAGATAATAAATCAAAAGAATTTATTAATTAGTTTACAAACAAAAGAAAATGAGTG

                            1387
DS571148_75000-100159       GGTTTGTATTTATATTGAAGATAAAATAATAAAAAAAGAATTTAACCAAATAACAAATCAAACACA
DS548107_14778-40922_rc     GATTTGTATTTATATTGAAGATAAAATAATAAAAAAAGAATTTAATCAAATAACAAATAAAACACA

                            1453
DS571148_75000-100159       TTTTATTTATTCAACAAAAACTAAAAAAAAATATCAATTAAAAGATAACATGATTATTTCATTTTC
DS548107_14778-40922_rc     TTTTATTTATTCAACAAAAATGAAAAAAAAGTATCAATTAAAAGATGATATAATCATTTCATTTTC

                            1519
DS571148_75000-100159       TCTTCATGATAATAATTGTTCATTATTTACTCTTGGAAATAGAATTGGTTTTATTCATATAGCAAA
DS548107_14778-40922_rc     TCTTCATGATAATAATTGTTCTTTATTTACTCTTGGAAATAGAATTGGTTTTATTCATATAACAAA

                            1585
DS571148_75000-100159       AACGAAAAATAAAAGATGTTGTGTTTCAAGATTATCTGAATTATATAAAGAAATGAATGATAATGA
DS548107_14778-40922_rc     AACTAACAATAAAAGGTGTTGTGTTTCAAGATTATCTGAATTATATAAAGAAATGAATGATAATGA

                            1651
DS571148_75000-100159       TTTATTCAATATTTCTTCTTCTATTAATAAATTTGAAATACATCAAATTATAATTCTTAAATTGAG
DS548107_14778-40922_rc     TTTATTCAATACTTCTTCTTCTATTAATAAATTTGAAATACATCAAATTATAATTCTTAAATTGAG

                            1717
DS571148_75000-100159       TTAAttgattttattttattgtaaaaatatt-aattcataagagtaaaaataattctcttcaatat
DS548107_14778-40922_rc     TTAAttgattttattttattgtaaataaattaaattaataataat--aaataattctcttgaatac

                            1783
DS571148_75000-100159       aaatg--aatttaaTTATAAATTTGATTTAAATAATTCAAATTTTTCAGTTCTTTGTTGTATAAAT
DS548107_14778-40922_rc     aaatggaaattaaaTTATAAATTTGATTTAAATGATTCAAATTTTTCATTTCTTTGTTGGATAAAT

                            1849
DS571148_75000-100159       TGTTCTATATCATTCACGTCGATTCCTTCATCTAATTCTCCATGTTCACCAACATTCTCAATAACA
DS548107_14778-40922_rc     TGTTCTATAGTATTCATATCTATTCCTTCATCTAACTCTAAATGTTCACCAACATTTTCAATAACA

                            1915
DS571148_75000-100159       TTTACCATTTTATCACAAACTTCAGAAAATTGAGGACGGTCTTCAAATGAATGTGCCCAACATTCA
DS548107_14778-40922_rc     TTTACCATTTTATCACAAACTTCAGAAAATTGAGGACGATCTTCAAATGAATGTGCCCAACATTCA

                            1981
DS571148_75000-100159       GTAATTAAAATTTTTAATGGTTGTGGCATTTTCATATCAATTTTAAGTCTTTCTCCATTTTCAACA
DS548107_14778-40922_rc     GTAATTAAAGTTTTTAATGGTTGTGGCATTTTATTATCAATTTTAAGTCTTTCTCCATTTTCAACA

                            2047
DS571148_75000-100159       AATCTTTTAATATCAAATAAAGACTTAAATTCTTTGTAAGGTTCATCTTGATAAAATATTTCCCAT
DS548107_14778-40922_rc     AATCTTTTAATATCAAATAAAGATTTAAAATCTTTATAAGGTTCATCTTGATAAAATATTTCCCAT

                            2113
DS571148_75000-100159       GCAGTAATTGCAAATGAATAAACATCTCCTGCAAATGTATAATGATCATGATAACATTCTGGTGCT
DS548107_14778-40922_rc     GCAGTAATTGCAAATGAATAAACATCTCCTGAAAATGTATAATGATCATGATAACATTCTGGAGCA

                            2179
DS571148_75000-100159       AAATAAATTGGTGTACCAAGTCCTTTATCTGTTAAATTATTAGTTTTTTTAGTAAATCTTGAAGTA
DS548107_14778-40922_rc     AGATAAATTGGTGTACCAAGTCCTTTATCTGTTAATTTATTAGTTTTTTTAGTAAATCTTGAAGTA

                            2245
DS571148_75000-100159       CCAAAATCTGTAATTTTAATACAACAACTTGAATCAGTATATAAAGAATTAACTAAAAGATTATCA
DS548107_14778-40922_rc     CCAAAATCAGTAATTTTAATACAACAACTTGAATCAGTATATAAAGAATTAACTAAAAGATTATCA

                            2311
DS571148_75000-100159       GGTTTAAGGTCTAAATGCATAATTCTATTTTCATGTAAGAATTCCATTCCTCTTGCAGTATCAAAT
DS548107_14778-40922_rc     GGTTTAAGATCTAAATGCATAATTCTATTTTCATGTAAAAATTCCATTCCTCTAGCAGTATCAAAT

                            2377
DS571148_75000-100159       AACATTCTAATTTTTAATTTATAAGGTAATCTAAGATAATTTGGTTTATTTTTTCTTAAATATTCA
DS548107_14778-40922_rc     AACATTCTAATTTTTAATTTATATGGTAATCTTAAATAATTTGACTTATTTTTTCTTAAATATTCT

                            2443
DS571148_75000-100159       CCTAATGATCCTAAAATAAAAAATTGTGTAACCATTGATATTTGTGGTATATATGTTATTGATCCT
DS548107_14778-40922_rc     CCTAATGATCCTAAAACAAAAAATTGTGTAACCATTGATATTTGTGGTATATATGTCACTGATCCT

                            2509
DS571148_75000-100159       ATATAATTTGCAATAAATGGATTTCTTAATTTACTCATTACTTCACATTCATTTATTACTTCATTC
DS548107_14778-40922_rc     ATATAATTCGCTATAAATGGATTTCTTAATTTACTCATTACTTCACATTCATTTATTACTTCATTC
```

2575
DS571148_75000-100159 TTTAAATCATTCATTTCTTCTTCTGTTAAGTTTTCCCATCTAAAAACTTTAATTGCTACTGGAACA
DS548107_14778-40922_rc TTTAAATCATTCATTTCTTCTTCTGTTAAATTTTCCCATCTAAAAATTTTAATTGCTACTGGAACA

2641
DS571148_75000-100159 CTACGATATTCTCCAATATATACTTTCCCCATTGCTCCTTCTGCAATTGGTTCTTCTGATATATTT
DS548107_14778-40922_rc CTACGATATTCTCCAATAAATACTCTTCCCATAGCTCCTTCTGCAATTGGTTCTTCTGATATATTT

2707
DS571148_75000-100159 AGTTCATCCATATCAATATGAGTAGAAGATGCAGCTTCAGTAGCAATAACAAAATTACCATGACAA
DS548107_14778-40922_rc AATTCATCCATATCAATATGAGTAGAAGATGCAGCTTCAGTAGCAATAACAAAATTACCATGACAA

2773
DS571148_75000-100159 TGAAGAGGAATATTTTTTAATTCTTTTTCCATTTGTAATTGATCACTTTGACTCCAATCATTAAAT
DS548107_14778-40922_rc TGAAGAGGAATATTTTTTAATTCTTTTTCCATTTGTAATTGATCACTTTGGTTCCAATCATTAAAT

2839
DS571148_75000-100159 GTTTTTTCTTTTAATAACTCAACAATTTTATTAAGATACCTTCTTGATTTACTAAACCAAACAGTA
DS548107_14778-40922_rc GTTTTTTCTTTTAATAAATCAGCAACTTTATGAAGATGTCTTCTTGAATTACTAAACCAAACAGTA

2905
DS571148_75000-100159 TAAGGGATACATACATTTTTTATTTTTGTAGTACAATGAAGAGTCATAAAAACAACAATATCTGTA
DS548107_14778-40922_rc TAAGGAATACATACATTTTTTATTTTTGTGGTGCAATGAAGAGTCATAAAAACAACAATATCTTTA

2971
DS571148_75000-100159 CTTCTTTTTGGACCAAGAATTTTTACTTGAGGATTAAAATGAAAAACATATTTAGGATTATTTGGA
DS548107_14778-40922_rc CTTCTTCTTGGACCAAGAATTTTTACTTGAGGATTAAAATGAAAAACATATTTAGGATTATTTGGT

3037
DS571148_75000-100159 GTATGAAAAATAATCATCATATCTTTCCTTTTTGATAAATTCTTTACATGTATTTCTTGATATCTT
DS548107_14778-40922_rc GTATGAAAAATAATCATCATATCTTTCCTTTTTGATAAATTCTTTACATGTATTTCTTGGTACCTT

3103
DS571148_75000-100159 GTTTCAAATATTTCCGTTGGAATTTCTGAACGACCAAAATCAAGACTTAATGGTGTAATTGAAAAT
DS548107_14778-40922_rc GTTTCAAATATTTCTGTTGGAATTTCTGAACGACCAAAATCAAGACTTAATGGTGAAATTGAAAAT

3169
DS571148_75000-100159 TTAAGACTTTTACTTACTTCAGGTGAATATCTTTTTGATCCACTAATATACATATAATATGGAGGT
DS548107_14778-40922_rc TTAAGATTTTTACTTAATTCAGGTGAATATCTTTTTGATCCACTAATATACATGTAATATGGAGGT

3235
DS571148_75000-100159 TGTTGACTTTTCCATATATCATCATTAAGAAGTAATTGAATAGAATGTGAGAATTTATAAATAATA
DS548107_14778-40922_rc TGTTGATTTTTCCATGTATCATCATTAAGAAGTAATTGAATAGAATGTGAGAATTTATAAATAATA

3301
DS571148_75000-100159 AATATAATGATTATGAGAATTATAATGAATAAAACAGCACAACACCCACAAACTATTCCTGCAATT
DS548107_14778-40922_rc AATCCAATAATTATGAGAATTATAATTAATAAAATAACACAACATCCACAAACTATTCCTGCAATT

3367
DS571148_75000-100159 GTTCCTGATGATATTTTTGGTTGACACGATTGTTGAATATTAGAAGATGGAATATATTTATTACTT
DS548107_14778-40922_rc GTTCCTGATGATAATTTTGGTTGACATGATTGTTGAATACTAGAAGATGGAATATATTTACTACTT

3433
DS571148_75000-100159 TGACATTTACAAAATTCATTACAATATTCTCCTCCATCACAATCTTCTCCTTCATCTCTCATACCA
DS548107_14778-40922_rc TCACATTTACAATATTCATTACAATATTCTCCTCCATCACAATCTTCTCCTTCATCTCTCATACCA

3499
DS571148_75000-100159 TTTCCACATGTTTCAAGAATACATTTTTCATTAAATAATTTATATCCTGATTTACATTTACAATCT
DS548107_14778-40922_rc TTTCCACATGTTTCAAGAATACATTTTTCATTAAATAATTTATACCCAGATTTACATTTACAATCT

3565
DS571148_75000-100159 ATATCACAATTTATGTCACCATTAAAACATTCTATTGTTTCATCTTTAATTCTTGATGTACAATTA
DS548107_14778-40922_rc ATATCACAATTTAAATCACCATCAAAACAATCTATTGTTTCGTCTTTAATTCTTGATGTACAATTA

3631
DS571148_75000-100159 TAAGAAATACATCGATGATTAACTAATACATATCCATCTTCACATAAACAATTTTCAGAACAAGAA
DS548107_14778-40922_rc TAAGAAATACATCTATGATTAACTAATGTGTATCCATCTATACATGAACAATTTTCAGAACAATAA

3697
DS571148_75000-100159 GAACTTCCAAAATAACATTGATTATCTCCTTTATTAGTACTTGGTTGCCATTGATTATTATAATAA
DS548107_14778-40922_rc GAACTTCCAAAATAACATTGATTATCTCCTTTATTAGTACTTGGCTGCCATTGGTCATTATAATAA

3763
DS571148_75000-100159 CATAATAAATGACAATCACTTCCACCATATCCTTCTTTACAAATACATTTTTCTTCAGATATTGAA
DS548107_14778-40922_rc CATAATAAATGACAATCACTTCCACCATATCCTTCTTTACAAATACATTTTTCTTCAGATATTGAA

3829
DS571148_75000-100159 CAATATCCATTTCCAATATCATTATGACAATTATTTGGACAAATAACTCTTAAAGATACATATATA
DS548107_14778-40922_rc CAATATCCATTTCCAATATCATTATGACAATTACTTGGACAAATAACTCTTAATGATACAAATATA

```
                             3895
DS571148_75000-100159        TCCAAATTATGTTCTTGAGTAATAAAAGTAAATTTTCTAACTCTATGTGATGGTTGAACAAGAAGA
DS548107_14778-40922_rc      TCCAAATTATGTTCTTGAGTAATAAAAGTAAATTTTCTAACTCTGTGTGATGGTTGAACAAGAAGA

                             3961
DS571148_75000-100159        AAAATTCCAATATTTCCATTTACGGTAACATAATTCCAAAATTGAACACACTGCTTATCATCATTT
DS548107_14778-40922_rc      TAAATTCCAGTATTTCCATTTACATTAACATAATTCCAAAATTGAACACACTGCTTATCTTCATTT

                             4027
DS571148_75000-100159        TCATCCCATTCATATTCTTCAACATAAAGTATTCTATTTTGTCTTTTAGTTGTCATTAACATGACT
DS548107_14778-40922_rc      TCATCCCAATCATATTCTTCAACATAAAGTACTCTATTATATCGTTTGGTTGTCATTAACATTACA

                             4093
DS571148_75000-100159        GCACTAATATTAAATTCATATTCTAAATCAATCATTTTACCAAAAATATGTGGCATACACACTGGA
DS548107_14778-40922_rc      GCACTAATATTAAATTCATATTCTAAATCAATCATTTTACCAAAAATATGTGGCATACAAACAGGA

                             4159
DS571148_75000-100159        TATCCAAAATATCCTTCTGATGCTAATGGTAATTGAACAGTTCCTGTTCGTTTAACTTCAGCAAGC
DS548107_14778-40922_rc      TATCCAAAATATCCTTCTGATGCTAATGGTAATTGAACAGTTCCTGTACGTTTAACTTCAGCAAGC

                             4225
DS571148_75000-100159        ATTTCAAGATTATCTCCTGAAATATAATATCTTACTCGTTCTGTTTCACTTTCAGTAAAACAAAAA
DS548107_14778-40922_rc      ATTTCAAGAGTATCTCCTGCAATATAATATCTTACTCGTTCTGTTGGGTTTTCAGTAAATGAAAAT

                             4291
DS571148_75000-100159        GAAAATTTTAAATCAACTTCTTCTGTTAAAGAGAAAAAATAATAATATATTGTGTCATCATTCATA
DS548107_14778-40922_rc      GAAAATATTAAATCAAGTTCTTCTGGTAGAGAGAAGAAATAATAATACATTGTATCGTCATTCATA

                             4357
DS571148_75000-100159        AACATTTGTTTTGGCATTTGGAAATGAGGATAAAGTACACTACCTAAACTTGACTGTCCAACACAT
DS548107_14778-40922_rc      AACATTTGTTTTGGCATTTGGAAATGAGGATATGATACACTACCTAAACTAGAATATCCAACACAT

                             4423
DS571148_75000-100159        AATTTATTTCCATGGTGTTGAGAAATAATATTAATAGTCATAGTATAGTCAACTGAATCTTCTTCT
DS548107_14778-40922_rc      AATTTATTTCCAAGATGTTGTGAAATAATATTAATGGTCATAGTATAATCAAAAGAATCTTCTTCT

                             4489
DS571148_75000-100159        CCATTAAGGTCAGTTACTGTAAAATCTAAATAAATAGTATCATTCTCCATTGCCTTAGAATCAAAT
DS548107_14778-40922_rc      CCATTAAGATCAGTTACTGTAAAATTTAAATAAATAGTGTCATTTTCCATTGCTTTAGAATCAAAT

                             4555
DS571148_75000-100159        TTAACCCAAAATCCTTGAACATAACCTCCATTCCCACAAGGAGCTTTTCTATATGGTTTATCTTTA
DS548107_14778-40922_rc      TTAACCCAAAATCCTTGAACATAACCTCCATGACCACAAGGGGTTTTTCTATATGGTTTATCTTTA

                             4621
DS571148_75000-100159        GTAAAAGTATAGGTATAACGTTCATCTAATTTTAACTCTATTACGTCTGTTGGTATATTATCACCA
DS548107_14778-40922_rc      TTAAAAACATAAGTATAACTTTCATCTAATTTTAACTCTATTACGTTTGTTGGTATATTATCACCG

                             4687
DS571148_75000-100159        TCATATACTAACGACCGTTTTTCACATCCATTTACAGTCATTACATTAAGAGTTGTTGATGTTGAA
DS548107_14778-40922_rc      TCATGTACTAACGCTCGTTTTTCACACCCATTTACGGTCATTACATTAAGAGTTGTTGACGTTGAA

                             4753
DS571148_75000-100159        TCAAGATTATCATTATCACAATAAAAACATGACCCATCTTGTTCATATCCTTCTAAGCATAAAGGA
DS548107_14778-40922_rc      TCAAGATTGTCGTTATCACAATAAAGACACGACCCATCTTGTTCATATCCTTCTAAACATATAGGA

                             4819
DS571148_75000-100159        CATGTATATTCTGCAATACAATTTGAAGAACTGCCACATCCAACAGAGCAATTATACTGACTAAAA
DS548107_14778-40922_rc      CATGTATATTCTGCAATACAATTTGAGGCACTACCACATCCAAGAGAACAATTGTACTGACTAAAA

                             4885
DS571148_75000-100159        CCAGTATAAAATAATTGAAGGAGTATCAACACAATCATTggaaatgaatttttatttaatcttaaa
DS548107_14778-40922_rc      ACAATACAGAATAACTGAAATAATATCAGCACAATCATttgaaatgaa-ttctatttaatcttaaa

                             4951
DS571148_75000-100159        atacaagtatatcttaataacacatagaaaaactcctttttattaaaattttattctaataatact
DS548107_14778-40922_rc      atatttatttatcataataacattttgaaataatttcttttattacaatacgattctaaatataat

                             5017
DS571148_75000-100159        attatttttcatattattatttacaacaattcatgtttttcacatcaactacggtttttt-aaaaa
DS548107_14778-40922_rc      atactttt--attcttttattt---aaaattcatgttttttccatcgatcactacttttttaaaaaa

                             5083
DS571148_75000-100159        aaactcgataaat-aagatattaaaaaatatattacaaaaatatttaccacattttgtgtttatat
DS548107_14778-40922_rc      aaattcgatgaataaaaacagtaaaaaatattttacaaaaa-attaaattcattatgcttttaaat

                             5149
DS571148_75000-100159        ttagtggtttttcttttttttttttgaaagtatttttatctaaaaatatatttaatggt-gaagtt
DS548107_14778-40922_rc      tttatgcctcttcctttttttttt--gaatgtattt--atctaaaaatata--taatagtggaagtt
```

```
                           5215
DS571148_75000-100159      tgacgtgtaatgtaaaagtaataattcttctaaaaaaaataaatacattcaaatgacttccaagat
DS548107_14778-40922_rc    tgatatgtaacgtaacacttattattcttcttaaaaaattaaa-attttaaagttacttctaactt

                           5281
DS571148_75000-100159      aattttacaaaagaaggaaatactataagagagcatttaattaaaaaataataat---aaataatt
DS548107_14778-40922_rc    agttt--c-aaacaaggaaataagatga-atactatttaattaaaaaataattattaaaaataatt

                           5347
DS571148_75000-100159      attatcttaataaaacaataatttttcaaagtttttgtttaatggaaccaataagaaaaattatct
DS548107_14778-40922_rc    gttattttaataaaacagt-ttttttcagagttt---tttgatggaaccaataagaagaattatct

                           5413
DS571148_75000-100159      aaaaaatttg-agttt-gagATGAAATTAAGTATTGGATATGATATTGGCAGTTCATCAATAAAAG
DS548107_14778-40922_rc    taaaaatttgtagtttaaagATGAAATTAAGTATTGGATATGATATTGGCAGTTCATCAATAAAAG

                           5479
DS571148_75000-100159      GGTCATTAGTTGATATAGAAAGTGGAACATCTTTAATAACTGATTATTATCCTAAAAAGGAAATGA
DS548107_14778-40922_rc    GGTCATTAGTTGATATAGAAAATGGAATATCTTTAATAAGTGATTATTATCCTAAGAAAGAAATGA

                           5545
DS571148_75000-100159      AAATTTTATCAAAACAAGATGGATTTGCTGAACAAAATGTAGAAGAATGGTGGAAAAATATTCAAC
DS548107_14778-40922_rc    AAATTCTATCAAAAGAAGATGGATTTGCTGAACAAAATGTAGAAGAATGGTGGAAGAATATTCAAC

                           5611
DS571148_75000-100159      AAGTTACTCAACAAATTGTTTATTTTATCAAGGAACATGGACTTTCAATTTCCAATATTGTTTCAA
DS548107_14778-40922_rc    AAATCACTCAACAAATTGTTTCTTTTATCGAAGAACATGGACTTTCAATTTCTAATATTGTTTCAA

                           5677
DS571148_75000-100159      TTGGTATATCTTATCAAATGCATGGAATTGTTTTAATTGATTCTCAAGGAAAAGTATTACGTGATG
DS548107_14778-40922_rc    TTGGTATATCTTATCAAATGCATGGAATTGTTTTAATTGATTGTAATGGAAAGGTATTACGTGATG

                           5743
DS571148_75000-100159      CAATTATTTGGTGTGATTCAAGAGGTGTATCATATGGCTATGATGCAGAACAAAAACTTGGACATG
DS548107_14778-40922_rc    CAATTATTTGGTGTGATTCAAGAGGTGTGTCATATGGTTGTGATGCAGAAAAAAAACTTGGACATG

                           5809
DS571148_75000-100159      ATTTTTGTTTAAAACATTTGTTAAATCTTCCTGGGAACTTTACAGCAACAAAATTAGCATGGATAA
DS548107_14778-40922_rc    ATTTTTGTTTAAAACATTTATTAAACCTTCCTGGGAACTTTACAGCAACAAAACTGGCATGGATAA

                           5875
DS571148_75000-100159      AAGAACATGAACCAGAGATTTTTAATCGTATTTATAAAGTATTACTTCCTGGTGATTATATTGCTT
DS548107_14778-40922_rc    AAGAACATGAACCAGAGGTTTTTAATCACATTTATAAGGTATTACTTCCTGGTGATTATATTGCTT

                           5941
DS571148_75000-100159      TTAGAATGACTGGTAAACCAACTACAACACAATCTGGATTATCAGAATGTATGTTATGGGATTATA
DS548107_14778-40922_rc    TTAGAATGACAGGTAAAGCAACTACAACACAATCTGGATTATCAGAATGTATGTTATGGGATTATG

                           6007
DS571148_75000-100159      AAAGACAAGAAATATCTACTGAAATGATGAATTGGTTAGGATTAAGAAATGACCAACTTGGAGATA
DS548107_14778-40922_rc    AAAGACAAGAAATATCTAGTGAAATGATGAATTGGTTAGGATTAAGAAATGATCAACTTGGAGGTA

                           6073
DS571148_75000-100159      TTGTTCCCACGTTTGGTGTTCAAGGTAAATTAAGAAAAGAAATTGCAAATGAACTTGGATTAGTTG
DS548107_14778-40922_rc    TTGTTCCAACATTTGGAATTCAAGGTAAATTAATAAAAGAAGTTGCAAATGAACTTGGATTGATTG

                           6139
DS571148_75000-100159      AGGATATTCCTATTACTTATAGAGCAGGTGATCAACCAAATAATGCTTTTTCATTAGGTGTATTAA
DS548107_14778-40922_rc    AGGGTATTCCTATTACTTATAGAGCAGGAGATCAACCAAATAATGCTTTTTCATTAGGTGTATTAA

                           6205
DS571148_75000-100159      ATCCAGGGGAAGTTGCATCTACAGCAGGAACATCAGGAGTAGTTTATGGTATAACAGAGTTTCCAT
DS548107_14778-40922_rc    ATCCTGGAGAAGTTGCATCCACAGCAGGAACATCAGGAGTCGTTTATGGTATAACTGAATGTCCAA

                           6271
DS571148_75000-100159      TATATGATGAATTATCAAGAATTAATACATTTATTCATGTAAATAATTCCCAATTTCAACCTAGAA
DS548107_14778-40922_rc    TATATGATGAATTATCAAGAATTAATACATTTATTCATGTAAATAATACTCAACTTAAACCTAAAA

                           6337
DS571148_75000-100159      ATGGAATATTATTATGTATTAATGGAACTGGATGTTTATATTCATTTATTAAGAAATTACTTGATA
DS548107_14778-40922_rc    ATGGAATATTATTATGTATTAATGGAACAGGGTGTTTATATTCATTTGTTAAAAAATTACTTGATA

                           6403
DS571148_75000-100159      GTGATTATAAAGTAATGGATGAATTAGCTGAACAAGTTCCTATTGGAAGTGATGGTCTTGTTATTC
DS548107_14778-40922_rc    GTGATTATGAAACAATGGACGAACTAGCTGAACAAGCCCCTATCGGAAGTGATGGTCTTGTTATTC

                           6469
DS571148_75000-100159      TCCCATTTGGTAATGGGAGTGAAAGAATGTTACAAAATAGTTGTATTGGATGTTCTATTGAACATT
DS548107_14778-40922_rc    TTCCATTTGGTAATGGTAGTGAAAGAATGTTACAAAACATTTCTATTGGATGTTCTATTGAACATT
```

```
                             6535
DS571148_75000-100159       TAAATTTTAATGTTCATACAAAACAACATCTTATCCGAGCATCACAAGAAGGAATTGTATTTGCCT
DS548107_14778-40922_rc     TAAATTTTAATGTTCATAACAAACAACATCTTATCCGAGCAGCACAAGAAGGAATTGTATTTGCTT

                             6601
DS571148_75000-100159       TTGCATATGGAATTGAACAAATGAGAAATATGGGAATGTCAATTACTACAATTAAAGCATGTTATA
DS548107_14778-40922_rc     TTGCATATGGAATTGAACAAATGAGAAATATGGGAATGTCAATTACTACAATTAAAGCATGTTATA

                             6667
DS571148_75000-100159       CAAATATGTTTCAAAGTAAATTATTTGTTAAAACATTATCTGCAGTAACAAATACAACAATTGAAC
DS548107_14778-40922_rc     CAAATATGTTTCAAAGTAAATTATTTGTTAAAACATTATCCGCAGTAACAAATACAACAATTGAAC

                             6733
DS571148_75000-100159       TTTACCAAACTGATGGTTCATTAGGAGCAGCATTAGCAAGTGCAGTTGGTAGTGGTTATTTTACTT
DS548107_14778-40922_rc     TTTATCAAACTGATGGTTCATTAGGAGCAGCATTAGCAAGTGCAGTTGGTAGTGGTTATTTTACTT

                             6799
DS571148_75000-100159       CATTAAACCAAGCATTAAATTCATTAAAAATGGTTGATAAATATACTATCCCAAAAGGAGAATGTA
DS548107_14778-40922_rc     CATTAGACCAAGCATTAAATTCATTAAAAATGGTTAGTAAATATACTTGTTCAATGAGTGAATCCA

                             6865
DS571148_75000-100159       TAGAATATTGTCAAGCTTATTCTCATTGGAAACAAGTTTTGAATGAACATCTGTCACATAATTATT
DS548107_14778-40922_rc     TAGAATATTGTCAAGCTTATTCTCATTGGAAGCAAGTTTTGAATGAACATCTGTCACATAGTTATT

                             6931
DS571148_75000-100159       AAt--atctttcaacttgaatagaatttttttcttttttttttttgataaatcccatttt-taa-a
DS548107_14778-40922_rc     AAtaaatctttaaaattgaatagaattt-----ctttttttttt---ataaattcctttttataaca

                             6997
DS571148_75000-100159       agaaaaaaaatctaaaa-taaaaagaaaaaaaaatgaattgttgaaacaacaaaaaaccaaaaaac
DS548107_14778-40922_rc     acaaaaaaaatctaaaaataaaaagaaaaaaaa-taatttgctg--------aaaaatcagaaaac

                             7063
DS571148_75000-100159       tctcaactaagaaaaaataataataaattataataaaatttattatttatataataaaaagaaaaa
DS548107_14778-40922_rc     tctcaacttggaaaa---aatgaaaaattataataaaatttattatttatataataaaaagaaaaa

                             7129
DS571148_75000-100159       actaaaaagaaaaaaaaaaaaaaacgaaaatgagacaaaaaaatatagtttgataaatagtaaata
DS548107_14778-40922_rc     actaaaaag-----aaaaaaaaaacgaaaataagacaaaaaaatatagtttgataaatagtaaata

                             7195
DS571148_75000-100159       gaattataaaataactagaaattaatgaactaagtttcactcaaaacaaaaagaaATGTCAGCTGC
DS548107_14778-40922_rc     gaattataaaataactaaaaattaatgaactaagtttcactcaaaataaaaagaaATGTCAGCTGC

                             7261
DS571148_75000-100159       ACCAACAGATGCTAAAAGTGTTAAATTAGTTGTTGTAGGAGATGGAGCTGTTGGAAAAACATGTTT
DS548107_14778-40922_rc     ACCAACAGATGCTAAAAGTGTTAAGTTAGTCGTCGTAGGAGATGGGGCTGTTGGAAAAACATGTTT

                             7327
DS571148_75000-100159       ATTAATTTGTTATACAACTAATGAATTTCCAAAAGATTATGTACCAACTGTATTTGATAATTATAT
DS548107_14778-40922_rc     ATTAATTTGTTATACAACTAATGAATTTCCAAAAGATTATGTACCAACTGTATTTGATAATTATAT

                             7393
DS571148_75000-100159       GGCTCCAATGACTGTTGATGGAGAACCTATTAATCTTGGACTATGGGATACTGCAGGACAAGAGGA
DS548107_14778-40922_rc     GGCACCAATGACTGTTGATGGAGAACCTATTAATCTTGGATTATGGGATACTGCAGGACAAGAAGA

                             7459
DS571148_75000-100159       CTATGAACAATTAAGACCATTGTCATATCCAAATACTGATCTTTTCTTATTATGTTTTTCAGTTAT
DS548107_14778-40922_rc     TTATGAACAATTAAGACCATTATCATATCCGAATACTGACCTTTTCTTATTGTGCTTTTCTGTTAT

                             7525
DS571148_75000-100159       ATCAAGAACATCATTTAATAATATTTCATCTAAATGGTTACCAGAAATTAAACATTATGAACCTAA
DS548107_14778-40922_rc     ATCAAGAACATCATTTAATAATATTTCATCTAAATGGTTACCAGAAATTAAACATTATGAACCTAA

                             7591
DS571148_75000-100159       ATGTAAAATGATGGTAGTTGGAACAAAGACTGATTGTCGTAATGATGAAGCTATGATCAGAAAATT
DS548107_14778-40922_rc     ATGTAAAATGATGGTCGTTGGAACAAAGACTGATTGTCGTAATGATGAGGCTATGGTCAGAAAATT

                             7657
DS571148_75000-100159       AGCTGATGAAAACCAAAAACCAATTACAACTGAAGAAGGTGAAAAACTTGCAAAAGATATTAAAGC
DS548107_14778-40922_rc     AGCTGATGAAAACCAAAAACCAATTACAACTGAAGAAGGTGAAAAACTTGCAAAAGATATTAAAGC

                             7723
DS571148_75000-100159       CATTTGTTATATGGAATGTTCAGCTTTAACTCGTTCTGGACTTAATCAAGTATTTGATGAAGCAAT
DS548107_14778-40922_rc     TATTTGTTATATGGAATGTTCAGCTTTAACTCGTTCTGGACTCAATCAAGTATTTGATGAAGCAAT

                             7789
DS571148_75000-100159       TCATATTGTTTTGAATAAAAATCAATCATCAAAGAAGAGTTCTAAGAAATGTGCTTTGTTGTAAGT
DS548107_14778-40922_rc     TCATATTGTTTTGAATAAAAATCAATCATCAAAGAAGAGCTCAAAGAAATGTGCTTTATTGTGAgt
```

```
                         7855
DS571148_75000-100159    TCATTCCAACCGTCTTATCGGTAATTTAATTGTTCTTTGTGAgtaattattattcttatttctaca
DS548107_14778-40922_rc  tcatttcaaccgtcttatcggtaatttaattgttctttgtgagtaattattcttttaatttctatc

                         7921
DS571148_75000-100159    ttttgaagataaaaattttgttattaggaattaattatattatttcttgtttattaaaacaaaat-
DS548107_14778-40922_rc  tttttaatataaatattttgttatta-aaactaatta---tatatcaagtttattaaaacaaaata

                         7987
DS571148_75000-100159    ccttcttataatgtcaatatctttatact-tactgagtttataattgtttttattctattacatta
DS548107_14778-40922_rc  ttttatcataattttgatccatttataataaattgaatttatatttgcttttatcatatttcattg

                         8053
DS571148_75000-100159    ttctgaaatatattctattggttcttcttattatttcaaatatcataatattgcaatgttttattt
DS548107_14778-40922_rc  ttcttgaatatattgtattagttcctctaagtattttaaatatc---atatcacagagttttattt

                         8119
DS571148_75000-100159    atgtttttcttc--aaaatttttggaatggaatgaaaaacataaaaaaaagaatg-ttagaacctt
DS548107_14778-40922_rc  atgtttttctttaaaaaacttttggaatggaataaaaaacaaaaaaaagagaagatttgggacctt

                         8185
DS571148_75000-100159    gattataaaatagaggatgtttgtctattactctataaactacttgagttctaaataaataaaagt
DS548107_14778-40922_rc  gattataaaata---gatgtttgtttat---------aactacttgaattctaaataaataaaagt

                         8251
DS571148_75000-100159    aattaacttcacatatgaaatatgaagataaaactgagatttaaaagattaaatgaaattcattaa
DS548107_14778-40922_rc  aattaacttcaca-atgaaatatgaagataaaatagagatttaaaagattgaatgaaattcattaa

                         8317
DS571148_75000-100159    acaatataagaatgaagggaattaatattcttttaactcctttgaaggttctaacagcatattttt
DS548107_14778-40922_rc  acaatatgatgatgatgggaattaatcttcttttaactactatgaatgttctaaccatatattttt

                         8383
DS571148_75000-100159    tttttaagtttcattcaattt-aaaaaagaaaactaatttcactaaaactccgcagagaaaaacaa
DS548107_14778-40922_rc  ttttaaagtttcattcaatttaaaaaaagaaaactaatttcaataaaactctgtagaaaaaaacaa

                         8449
DS571148_75000-100159    taaaaatattatgtatcttttattcattagttgattaaaggtgcagcgaataaaacgagacaaaaa
DS548107_14778-40922_rc  taaaaatattatgtatcttttattcattaattgattaaaggtgcagcgaataaaattagacaaaaa

                         8515
DS571148_75000-100159    aaaattggagaatgatttgaaatagaaaatgataatcatgaaaatgaagaaaaagaataaaaagga
DS548107_14778-40922_rc  aaa--tggagaatgatttgaaatagaaaatgataatcataaaaatgaagaaaaagaataaaaaaga

                         8581
DS571148_75000-100159    agaatattggtaagattggtagccaatagtttcttgaataaggttggagttatggttttggatagc
DS548107_14778-40922_rc  aagatattggtaagattggtaaccaatagtttcttgattaagattggagttatggttttggctaat

                         8647
DS571148_75000-100159    actcgaaattctaagcaaactaaatgggttaaaaaccaaaa-aaaagagaaggcagcattactttc
DS548107_14778-40922_rc  actcaaaattttaagcaaactaaatgggttaaaaactaaaataagagagaagacggcattattttc

                         8713
DS571148_75000-100159    tatgaaaaagaactaaaaatagtggaagtttaaaact----agtaagaaagtaagggttagtataa
DS548107_14778-40922_rc  tttgaaaaagaactaaaaatagtgaaagttaagaacttgtaagtaagtaagtaagggttagtataa

                         8779
DS571148_75000-100159    tttctttgatggagcctgacaaatcatc-aaactaaaatagaattctaaaaagtatttaaaggtaa
DS548107_14778-40922_rc  tttctttgatggagcctgacaaatcatcgaaactaaaatagaattctccaaagtatttaaagggaa

                         8845
DS571148_75000-100159    taaacttttatttaaaatcactatttcataATGTCAAGAACAACTAAAGTCCAAAAGAAAAGTGAC
DS548107_14778-40922_rc  taaacttttatttataatcactgtttcat-ATGTCAAGAGCAACTAAAGTCCAAAAGAAAAGTGAT

                         8911
DS571148_75000-100159    TCCGAGCGTCTCTGCGAAAAATACTTTGAAATTCGAAATTACTTAATGTGTCAACAAGGAGTTCTT
DS548107_14778-40922_rc  TCTGAACGTCTCTGTGAAAAATACTTTGAAATTCGAAATTACTTAATGTGTCAACAAGGAGTTCTT

                         8977
DS571148_75000-100159    CTACTTCTTTTGAACCAACACTTTGATATTACCATTACAAGACCAACAAAGAAATCATTAGTTACT
DS548107_14778-40922_rc  CTACTTCTTTTAAATCAACACTTTGATATTACTATTACAAGACCAACAAAGAAATCATTAGTTACA

                         9043
DS571148_75000-100159    GGACAAGTTATTAAAGTTAAAACTCTTAGAGACAAATTTGCTGAAGTTGATGTAGTAGACTTTGTT
DS548107_14778-40922_rc  GGACAAGTTATTAAAGTTAAAACTCTTAGAGATAAATTTGCTGAAGTTGATGTAGTAGATTTTGTT

                         9109
DS571148_75000-100159    GAAGAACGTTGTAAAGAGAAAATGAGAGTTGATGTAGCTAATGGAATAAATGAAATAAGTGCATGT
DS548107_14778-40922_rc  GAAGAACGTTGTAAAGAGAAAATGAGGGTTGATATGGCTAATGGAATAAATGAAATAAGTGCATGT
```

```
                             9175
DS571148_75000-100159        AGAAGATACACTAATAATAAAATAGCTGAAAATCATCATCTTCTTCTTGATATGTTAAGAATGTAT
DS548107_14778-40922_rc      AGAAGGTATACTAACAATAAAATAGCTGAAAATCATCATCTTCTTCTTGATATGTTGAGAATGTAT

                             9241
DS571148_75000-100159        GGAATTACTTTTAATACAACATTTTCTTCTGGAAAACACAACTCTACAAAATTAGAAACAATTAGT
DS548107_14778-40922_rc      GGAATTACTTTTAATACAACATTTTCTTCTGGAAAACACAATTCTACAAAATTAGAAACTATTAGT

                             9307
DS571148_75000-100159        GAAATTTTCATTAATTCTAAAGTCCTCTTCACTAAAAATCAAATTAAAGCTATAGGTAAAGAAATT
DS548107_14778-40922_rc      GAAATATTTATCAATTCTAAAGTCCTCTTTACTAAAAATCAAATTAAAGCTATTGGTAAGGAAATT

                             9373
DS571148_75000-100159        AATAACTATTTCTTTAATCTTGTAGATAAAGAAAAAGAAGTTATTTTAGAAAAGAATAACCAATTT
DS548107_14778-40922_rc      AATAATTATTTCTTTAATCTTGTAGATAAAGAAAAAGAGGTTGTTTTAGAAAAAAATAACCAGTTT

                             9439
DS571148_75000-100159        CTTCAATCATTTAGTATATATCACCAGGATTCTTCTTCGTGTTAAtttatgttttgaatatatttt
DS548107_14778-40922_rc      CTTCAATCATTTAGTATATATCACCAGGATTCTTCTTCGTGTTAAtttatgttttgaatatatttc

                             9505
DS571148_75000-100159        ttatgtctattttttttcaatgagaaacattggcatcacaaaatttaagttagtttgataatactt
DS548107_14778-40922_rc      ttaactct-tttttttttaatgagaaaca--agtatcaaaaaatttaagttagtttgataatactt

                             9571
DS571148_75000-100159        tttgagtctaattttttgctaaaaatggtttttcataaataatgaattctaaaaatatacaagtaa
DS548107_14778-40922_rc      tttgattctaattttttgctaaaaatggttttttataaataattatttctaaaaatataaatctaa

                             9637
DS571148_75000-100159        cgacaaggcagttattctataattctataattggttattgtattttttaattaatgaaaactaaaa
DS548107_14778-40922_rc      cgacaaggcagttattctgaaattttataattagatataatattttttaattaat-aaaactaaaa

                             9703
DS571148_75000-100159        gtatagaaacttgatattaaaatcccatttgcaagt-cttttttatcctctttatttgagttaaaa
DS548107_14778-40922_rc      gtatgggaacatgatattacaattccattagaaagtactcttttatcctcttcattttgtttaaaa

                             9769
DS571148_75000-100159        atattcaattaacgcatttttttgtaaaaaaaaaaatttttttaaattaaattatttttaacgact
DS548107_14778-40922_rc      gtatttaattaactcattttttttt---gaaaaaaatttttttaaattaacttattcttaattctt

                             9835
DS571148_75000-100159        tgactctgtgaacaattttttgtttaaagagttattttattgatgcattgtattggatgatcta--
DS548107_14778-40922_rc      tgattctttgaacaacgttttgttcaaagagttgtttcattaatatactgtgttgttcaatctact

                             9901
DS571148_75000-100159        atattaagtggtttatttcataaaaaaaaatattcttattgaataatcaaaaaaaaaaatactttt
DS548107_14778-40922_rc      acattaagtga-tctgcttcagacaaaaaatattcttgttgaatattcattaagaagaacattttt

                             9967
DS571148_75000-100159        attattatcattaaccaataaatctgaaaagctaaaaataaatccataaaaaaaaaataaatttca
DS548107_14778-40922_rc      ataactctcattaactgataaatcagaaaaactaaaa--aaatc--------acaaataaatttca

                            10033
DS571148_75000-100159        acctttt--tgtttgtctcagaatttatttctttgataaattctgagactagatctccgtgattaa
DS548107_14778-40922_rc      gccctttagtgtttgctttagagttgacttctttgataaaatcatctacaaaatctccgagattca

                            10099
DS571148_75000-100159        atgaaatatattttttttgtttttctttttctccccctccttatttaat---gatttatcctttaat
DS548107_14778-40922_rc      gtgaaatg--------tgtttttctttttctcctcctccatatttaattcggatttgtcctttaat

                            10165
DS571148_75000-100159        tttaataactttaacaatttgtattatatcatctaacatgatttggtttttgatttcccaTTATTC
DS548107_14778-40922_rc      tttagtgactttaacaatttgtgttatatcgtctaacatgacttggtctttaatttcccattgttc

                            10231
DS571148_75000-100159        TCTTTCTTGGTTTATTACAAGAATTGTTTCTTTATTAACTAAACAAATAGTCTCTTCTTTGGGTTT
DS548107_14778-40922_rc      tctttcttgagttattacaacaactgcctctttactaactaaacaaatagcctcttctttgctttt

                            10297
DS571148_75000-100159        TCCTTTACATATTATGCACTTAAATTGTGTATTCTCTTTCTTTAGACTTTCACAATACCATTTTAT
DS548107_14778-40922_rc      tcatttacaccttacgcacttaaattgtttattctctttcattacactttcacaacacccatttat

                            10363
DS571148_75000-100159        TGGTTTGGATTTAACAATTGCTGTTTCTTTAA----------------------------------
DS548107_14778-40922_rc      tgatttagctttaacaataaTCATTTTTTTAACTTTTCTTTCTTTTCTAATTCCTTACTTACTTCT

                            10429
DS571148_75000-100159        ------TCTTTTTTTTC-------------------------------------------------
DS548107_14778-40922_rc      TTTGTTTCTTCTTTTTCAATTCTTGTTTCTTGTTCTTTTTTATTTTCTTCTTCTTGGTTTTCTGCT
```

```
                            10495
DS571148_75000-100159       ---------TTCCTCTCTTTTTGACGTTTTCTTTCCTTGTTAAGTTCTTCTGTTAGCCATAGTAGT
DS548107_14778-40922_rc     GTTTCTTCTTTCTTTTCTTCATCACTCTTTTTTTCCTTATTAGGTTCTTCTGTTAGCCATCGTAGT

                            10561
DS571148_75000-100159       GTTTGCCTGAATGAATTTCCCCGTCTTGATTTTGTATATTCTTTTTCTACTTTGATTTTCCCATCT
DS548107_14778-40922_rc     GTTTGCCTGAATGAATTTCCCCATCTTGACTTTGTTTGTTCTTTTTCTACTTTGATTTTCCCATCG

                            10627
DS571148_75000-100159       TCTTCTTTTCCTTCTATCTCTTCTTTATTCTCTTGTTTTTCTTTCTTGTCATCTTCCTTTTTTTCT
DS548107_14778-40922_rc     TCTTCTTTTCCTTCTGTCTCTCCTTTAATCTCTTGTTTTTCTTCTTTGTCGCCTTCCTTTTTTTCT

                            10693
DS571148_75000-100159       GTTTCTTCTTCTGTTTTTCGTTTATCT---------------------------------------
DS548107_14778-40922_rc     TTCTCTCCATCTTTCTTTCGTTTATCTCTAATTCCTTCTGTGTTCTCCTTTACTTCTCCTCTTTTA

                            10759
DS571148_75000-100159       ------------------------------------------------------------------
DS548107_14778-40922_rc     AATCGTGAAAATAATCCTCCTGTGACACCAAGACATGCAGGACATTTCAGTGTATTACGATTTTCT

                            10825
DS571148_75000-100159       ------------------------------------------------------------------
DS548107_14778-40922_rc     ATTTCAAATCCTAAGCTCCCTTCAATAAAGTATTTATGATACATTTTATATCCTCCAACCATAATT

                            10891
DS571148_75000-100159       ------------------------------------------------------------------
DS548107_14778-40922_rc     CCTACTCTCTTATAACCTCTACTGTATAGTTCATAGCACAAGATAGCCATTTCTTTTACTGTGGGA

                            10957
DS571148_75000-100159       ------------------------------------------------------------------
DS548107_14778-40922_rc     TTACCTCCTTCTCCAGATGCGTAGAACACTACAGGTTGTTTCTGTGTCAATTCTGAAAATATTTTT

                            11023
DS571148_75000-100159       ------------------------------------------------------------------
DS548107_14778-40922_rc     TCTATATTTTCTTTCTTTTGATTATCAATCTCTAATGAAATTGCATGTGTTAAATGTCCTTGTTGG

                            11089
DS571148_75000-100159       ---------------------TTTATCATttggagtggttgtttaaatggtattgtgtaatt----
DS548107_14778-40922_rc     TACTGTTCATTTGATCGAACATCTATCATTTGGAGTGGTTGTTTAAATAGTAGGGTGTAATTTACA

                            11155
DS571148_75000-100159       ------------------------------------------------------------------
DS548107_14778-40922_rc     ATATCGGTTGGTGTCAGTGAGACAACAAATGCTTCTGAAATGTTAGCAGTGTACCATTGTTTGAAT

                            11221
DS571148_75000-100159       ------------------------------------------------------------------
DS548107_14778-40922_rc     TGAGGTGTATTATTCAAACACCCATTAATTAATCCATTAAAGGAGGCTGGACTTTTGGCTAAATAA

                            11287
DS571148_75000-100159       ------------------------------------------------------------------
DS548107_14778-40922_rc     CATTTAGCAATTGAGAGAAGAATCTTAATTTCATCAGATGTATAACTTTTTGATGTGATAAAGTTA

                            11353
DS571148_75000-100159       ------------------------------------------------------------------
DS548107_14778-40922_rc     AGAACCTCTTCTTGTGTCTGAAGTGTTTTCAATTGATTTCCCATGTCTTCGTAAATAGCAACTATC

                            11419
DS571148_75000-100159       ------------------------------------------------------------------
DS548107_14778-40922_rc     ATATAGAAATAAAGATTTTTATTTTGGAATGTGAATATTCCATCTAAAAGAGTCAGAATTGCTTTA

                            11485
DS571148_75000-100159       ------------------------------------------------------------------
DS548107_14778-40922_rc     AAGTCTTTACACATTTTAACATTTAAATTAAAAATGAATCGACGAAGTAATTTATCCGTTGTCAAT

                            11551
DS571148_75000-100159       ------------------------------------------------------tgtgttaatgat
DS548107_14778-40922_rc     TTCATTTTATCCAGATGAATAGTAACATCAGGGAAATAATATAACAAGATCATATGTGTTAATGAT

                            11617
DS571148_75000-100159       -------------------------------------aaaatcccatataactaatataaatcctc
DS548107_14778-40922_rc     AATAATCCATGCCATCCTCGTGGAAGTAAATTAATGCAAAATCCCATATAGCTATTATAAATCCTC

                            11683
DS571148_75000-100159       tgtataaaaggtaagtcaattcttcccataattttttccataattaatacaaataatacaatctct
DS548107_14778-40922_rc     TGTATAAAAGGTAAGTCGATTCTTCCCATAATTTTTTCCATAACCAACACAAATAAAACAATCTCT

                            11749
DS571148_75000-100159       tcataatattgacatggatttatatgaataatatataacactgtctcctttgcatcattaatttta
DS548107_14778-40922_rc     TCATAGTATTGACATGGGTTTGTATGAATGAGATATAGTGCTGTCTTCTTTGCGTCATCAATTTTA
```

```
                                11815
DS571148_75000-100159     tcttttgataattcaaactttgtgaggtattcttcaattataataggctttcctatttctcgttgt
DS548107_14778-40922_rc   TCTTTTGATAATTCAAACTTTGTGAGATATTCTTCAATTGTGATAAGTTCTCCCATTTCTCTTTGT

                                11881
DS571148_75000-100159     actttacattcaataccatctgcttttagaattaaatactacgtttgctgtgagtttttattttag
DS548107_14778-40922_rc   ACCTTACACTCAATACCATCTACTTTTGGAATTAAATActaagtttactgtgagtctttgttttag

                                11947
DS571148_75000-100159     ttttacttatctttttttataacttctctaaacaaacttcatacaattcaactctaaactcatcggg
DS548107_14778-40922_rc   ttttatatatc-tttttatactttctctaaacAAACTTCATATAATTCAACTCTGAGCTCATCTGG

                                12013
DS571148_75000-100159     aattggatattcctttgctaaatgtgaaattgtaataaaaccttcatctgctttttcaggttgata
DS548107_14778-40922_rc   AATTTAGTATTCCTTTGCCAGTTGTGAGACTGTAATAAAATCTTCATctgcttttgctcgttgata

                                12079
DS571148_75000-100159     tagttcatttattcttttttctatctcttcttttctatttcaaaaa-ctt----------------
DS548107_14778-40922_rc   taattcatttattcttttttccatcttttcttttctatttcaaagatcttcctagttctattggta

                                12145
DS571148_75000-100159     ---------ttttaactctattagaaaaagcttttataaccaaaaaatatattacttatgactgtt
DS548107_14778-40922_rc   attttcttattttagctctattaaaaagtaatatgataaacaaaaaa-atacacctta--acatct

                                12211
DS571148_75000-100159     ttactaatataacataaaaaataaaaacaatactaaatagtaagataattaaattaaTTATCCATT
DS548107_14778-40922_rc   ttattaatgtagc-taaaaaataaaaataa-a-ttaacaccatgataattaaattaaTTATCCATT

                                12277
DS571148_75000-100159     GTAAGTAGCTGCATTAGTTGCTTCATTATCTGCTCCAACATATTCTGCATTTTCATTAGTTGTTGT
DS548107_14778-40922_rc   ATAAGTAGCAGCATTAGTAGCTTCGTTTTCTGCTCCAACATATTCTGCATTCTCATTGGTTGTTGT

                                12343
DS571148_75000-100159     AATGGCATTCTTCATAGCTGATGAAACAAGTTGATAAGTCTTAAATAATCCAATAGAAACAACAAT
DS548107_14778-40922_rc   GATAGCATTCTTCATAGCTGATGAAACAAGTTGGTATGTCTTAAACAATCCAATAGAAACAACAAT

                                12409
DS571148_75000-100159     TAATGCAACTACGACTGCAACTACAACTACAGCAACAGTAGTTGCAGCTGCAATAGCTCCAACATT
DS548107_14778-40922_rc   TAATGCAACTACAACAGAAACAACAACTGCTGCTACAGTAGTTGCTGCTGCGATGGCTCCAACATT

                                12475
DS571148_75000-100159     ATTAATTTCTCCTGCGAGAATACACTCGGTGTGATTATTAAGTTGTTGTGTTCTATCTGTTTGATT
DS548107_14778-40922_rc   ATTAATTTCTCCTGCGAGAATACATTCAGTATAGTTATTGAGTTGTTGTGTTCTATCTGATTGATT

                                12541
DS571148_75000-100159     CCAAACACGACAATTACCACATGCATCAAGATCATAATATTCTTTATCTGTTTCTCCATACATAGT
DS548107_14778-40922_rc   CCAGACACGGCAATTACCACATGCATCAAGATCATAATATTCTGTATCAGTTTCTTCATACATAGT

                                12607
DS571148_75000-100159     AAGAACTGATGAACCGTCAAGATATGAACATTGACAATAGAAACATCTTCCTTGATTTGGTGTGAT
DS548107_14778-40922_rc   AAGAACTGCTGATCCATCAAGATATGAGCATTGACAATAGAAACATCTTCCTTGATTTGGTGTAAT

                                12673
DS571148_75000-100159     TGTTGGGAATTCTTCTGAAACAGTACATGTTTTCTTTGTGTAAATAGTTTCTCCAGTTGTTTGATC
DS548107_14778-40922_rc   TGTTGGGAATTCTTCTGAGACTGTACATGTTTTCTTGTTATATACTGTTTCACCTGTAGCTGGATC

                                12739
DS571148_75000-100159     ACATGTTGCAGTTTCGCATGGATTTCTTGGTTTTGGATCAATATTTGATCGTTCTGTATCAATTTC
DS548107_14778-40922_rc   ACATGTTGCTGTTTCACATGGATTTCTTGCTTTTGGATCAATATTTGATTTTTCGGTATCAATCTC

                                12805
DS571148_75000-100159     CCATTTATGTGTGATTTTTCCATCACTTCCTTTACTCTCAACACATTTATATTTCAAACATGCATT
DS548107_14778-40922_rc   CCATGTGTGTGTTATTGTTCCATCACTTCCTTTATTCTCAACACATTTATATTTCTTACAAGCATT

                                12871
DS571148_75000-100159     GTTAAGTTCAACTCCTGATTTAGTTCTAAAATGTTCACTATCTAATGATGGATCATCAGTAAGTTG
DS548107_14778-40922_rc   GTTAAGTTCTACTCCTGATTTAATTCTGAAATAAGCAATATCTAATGATGGATCATCAGTAAGTGG

                                12937
DS571148_75000-100159     TACAAATTTACAAAGATATGTAGTACCATCACATTCTCCTGTCATTGCTTTACATCCATAATCTAC
DS548107_14778-40922_rc   TACAAACTTACAAAAGTATGTTGTACCATCACATTCTCCTGTCATTACTCTACATCCATAATCTAC

                                13003
DS571148_75000-100159     TTCTGTGTTTTGATTACATTTTTCTACATCAGCAATAATTGGACAACTATTATCATTTTGACAAAC
DS548107_14778-40922_rc   TTCTGTATTTTGATTACATTTTGTTGCATTAATAACAATTGGACAGCTATTATCATTTTGACAAAC

                                13069
DS571148_75000-100159     AGTACTTGCACATTCTACTTGCTCAATTCCTTCTTTTCCTTCAATGAATGCTTTACGTGCATCTTC
DS548107_14778-40922_rc   AGTACCTGCACATTCTACATTTTCTACTCCTTCTTTTCCATTAAGGAATGCTTTACGTGCTTCTTC
```

```
                             13135
DS571148_75000-100159     TATTCTCTTATTGTTAGTGTCACATGTTGTTTTTCCAGTATCTTTACATGTTGTTGTTTTTATTCC
DS548107_14778-40922_rc   AATTTTCTTGTTGTTAGTGTCACATGTTGTTTTTGAAGTACTCTTACAGGTTGTTGTTTTTACTCC

                             13201
DS571148_75000-100159     ATCTTTTTCTGTAATAACAATCTCTTGAATCTTACATTCAATAGGTTGACAATCAAAGTTAGGATC
DS548107_14778-40922_rc   ATCTTTTTCCTCAATAATAATCTCTTGAATTGTACATTCAACTGGTTGACAATCAAAGTTAGGATC

                             13267
DS571148_75000-100159     AATAATGCAATTGTTATTCTTACATTTGAATGGAACTCCTCCATTCTTAGTCTCTTCTGCACACAA
DS548107_14778-40922_rc   AATAATACATTCATTGTTCTTGCATTTATATGGAACTCCTCCATTATTAGTTTCTTCTGCACATAA

                             13333
DS571148_75000-100159     ATCAATTTCTTCTACTTTTTCTTTAGTTTCAGTTAATGAACATGTTTTACATTCTCTTGTTGTTAT
DS548107_14778-40922_rc   ATCAACTTCTTGTACTTTATCCTTACTTTCAACTAATGAGCATGTTTTACATTCTCTTGTTGTTAT

                             13399
DS571148_75000-100159     TTCGTATGTACAATATGCTTCTTTGTCTGCCTCTTTCATAAATTCTTCACTTTGTGGTAAATGAAG
DS548107_14778-40922_rc   TTTGTATGTACAATATGCTTCTTTGTCTGCCTCTTTCATAAATTCTTCACTTTGTGGTAAATGAAG

                             13465
DS571148_75000-100159     TTTATAACTAGTTAAATTGAATAATGAACTGTAATGATAGGTAGCTGTAACATATGCTTGACATGG
DS548107_14778-40922_rc   TTTGTATTTAGTTAAATTGAATAATGAACTGTAATGATAAGTAGCTGTAACATATGCTTGACATGG

                             13531
DS571148_75000-100159     TAAATAGAATTTATAAATTTCTCCAAAATTATCATCATTAGCTAAACAATCAACATAGGTTCTTGG
DS548107_14778-40922_rc   TAAATAGAAGTTATAAATCTTTCCAAAGTTATCGTCATTAGCTAAACAATCAACATAGGTTCTTGG

                             13597
DS571148_75000-100159     AATGCATTGTTCATTGACACAATCTTTTGGAATATTGTATTGATATTCTTTTCCATCTTCTAAAGT
DS548107_14778-40922_rc   AATACATTGTTCATTAACACAATCTTTTGGAATAGTATAATCATACTCTTTTCCATCTTCTAAAGT

                             13663
DS571148_75000-100159     GACTTTACGTTCTAATTTTTGATCTTTGCATTTCTCTACAAATTTACCTTTTTCGCATACTGAACA
DS548107_14778-40922_rc   AACTTTACGTTCTAATTTTTGATCTTTGCATTTCTCTGTAAATTTACCTCCTTCACATTCTGAACA

                             13729
DS571148_75000-100159     TGATGGTGTTGGCTTAACAGTTGCTTTACATGTTCCTGTTTTTGAGTCATATGTATATGTTCTGTA
DS548107_14778-40922_rc   TGCTGGTGTTGGTTTAACAGTTACTTTACATGTTCCTGTTTTTGCATCATAAGTGTATGTTCTGTA

                             13795
DS571148_75000-100159     TGTAGTATCAGCTGGACATGTTAAACTAGAACATTCAGCACAAATATCATTATCTGATTTAGAAGA
DS548107_14778-40922_rc   TGTGGCATCAGCTGGACATGTTAAACTAGAACACTTAGCACAAATGTCATTGTCAGATTT---AGA

                             13861
DS571148_75000-100159     AGTTTTATCTTTTTCAAATACGCAAGAATAAGAATTATC---TCCATTTGGTTGACACTTACCATT
DS548107_14778-40922_rc   AGTGTCATCTTTATCAAATTCACATGAATAAGATTTGTCTTTTTCATTTTGTTTACATTTACCATT

                             13927
DS571148_75000-100159     AAAGTTTCCACATTGTGATTTACGTGAATCACAATCATCATGATATGTAATAACATTATCTGTTCC
DS548107_14778-40922_rc   AAAGTTTCCACATTGTGATTTACGTGAACTACAATCATCATGATATCTAATAACATTGTCTGTTCC

                             13993
DS571148_75000-100159     CATTGAACAATATCCACATCCTACATTAGAAACATCACCAACTGCTTGAACAACACATTGTCCATT
DS548107_14778-40922_rc   CATTGAACAATATCCACATCCTACATTAGAAACATCACCAACTGCTTGAACAACACATTCTCCATT

                             14059
DS571148_75000-100159     CAAACATGTAGCTGAGTAATATGGTTTACACATAGTTTCAGATTTACCATCTTGAGCAAATTCAAT
DS548107_14778-40922_rc   CAAACATGTAGCTGAGTAATGATCTTTGCACATAGTTTCAGATTTACCATCATTAGCAAATGTAAT

                             14125
DS571148_75000-100159     CTTAGTGTTTGAACAATCAACATTCTCCCAATATGTTGTTTTACTTGTCTTATTACATCTCTTAAT
DS548107_14778-40922_rc   CTTAGTATTAGAACAATCAACATTCTCCCAATATGTAGTCTTACTTGTCTTATTACATCTCTTAAT

                             14191
DS571148_75000-100159     AAGACATCCATCTCTAACAAGATCTTCTGTACATTCAGAGACAATACATTTTGGTTTATCTCCAGT
DS548107_14778-40922_rc   AAGACATCCATCTCTAACAAGATCTTCTGTACATTCAGAGACAACACATTGTGGCTTGTCTCCAGT

                             14257
DS571148_75000-100159     ATAATCACAAATTTCATGTTTACTAGAATCACAATAATTACCATTTTCTACCTTATTACATTTACA
DS548107_14778-40922_rc   ATAATCACAAATTTCGTGTTTATTAGAATCACAATAATTACCATTCTTTACTTTATTACATTTACA

                             14323
DS571148_75000-100159     TTGATTACCAGTTGATGCATCACATTTACATACTGATCCTGTAGAATCTCCACCACATGATGAGTA
DS548107_14778-40922_rc   TTGATTACCAGTTGATGCATCACACTTACATGCTGATCCGGTAGAATCTCCACCACATGATGAATA

                             14389
DS571148_75000-100159     TGTATCACATGATAATCTGTTCTCACACATACCTGTATTGAGATTACACTTAGCTACATAACATGA
DS548107_14778-40922_rc   TGTATCACATGATAATCTGTTCTCACACATACCTGTATTGAGATTACATTTAGCTACATAACATGA
```

```
                          14455
DS571148_75000-100159     CATTTCAATGTATGGTGATGTTTTTACACATACCTTTTCTTGAACAGTACATCTTTGTTCTGAATC
DS548107_14778-40922_rc   CATTTCAATATATGGTGATGTTTTTACACATACCTTTTCTTGAACAGTACATCTCTGTTGTGAATC

                          14521
DS571148_75000-100159     ACAAACATATTCTTGACAGTTAGTAGTTTTTCCTGTACACAAATTTCCGTGTTTATATTTCTTACA
DS548107_14778-40922_rc   ACAAACATATTCTTGACAGTTAGTAGTCTTTCCTGTGCATTTGTCTCCATGTTTATCTTTCTTACA

                          14587
DS571148_75000-100159     AAAACCAGTAGTTTCATCACATGTATAACTCCAACAATATCCATCAGAACTTGGTTTTTGATCTGC
DS548107_14778-40922_rc   AAAACCAGTAGTTTCATCACATGTATAACTCCAACAATATCCATCTGAACTTGGTTTTTGATCTGC

                          14653
DS571148_75000-100159     TTCACAATTAGCTTTTTGTTTACATACTTCCTTTAGATTATTATCGTCATCACAATATGGTACCAT
DS548107_14778-40922_rc   TTCACAGTTAGGCTTTTGTTTACATGTTTCAGTAAC---ACTATCTCCCTCACAATATGGTACCAT

                          14719
DS571148_75000-100159     GCATTTCACTTTTGGTTCACAAGATGCGTTATATTTTGTAACAACACATCCTTCAAGATCTTTACA
DS548107_14778-40922_rc   ACATTTTACTTTTGGTTCACAAGATTTGTTGTATTGTTTGATAACACATCCTTCAAGATCTCTACA

                          14785
DS571148_75000-100159     TTCATATAAACCTACACATGTTGCTGGACATTCTTTCTTTGCTTTTGGTTTAGCTTTAGGCCATGG
DS548107_14778-40922_rc   TTCATATGAACCTACACAGGTATCTGGACATTCTTTCTTTGCCTTTGGTTTAGCTTTAGGCCATGG

                          14851
DS571148_75000-100159     AATACATGATCCTGTTTCTACATCACAGGATGTATAGAAACAATCATTCTCACAACACATTGGACA
DS548107_14778-40922_rc   AATACATGATCCTGTTTCTACATCACATGATGTGTAGAAGCAATCATTCTCACAACACATTGGACA

                          14917
DS571148_75000-100159     TGTGCATCTATTCATTGCATCACAAAATCCTGAACATTTATCTCTATCACATCTTTCTGTTAAATT
DS548107_14778-40922_rc   TGTACATCTGTTCATTGCATCACAAAATCCTGAACATTTATTTCTATCACATCTTTCTGTTAAATT

                          14983
DS571148_75000-100159     AGCTCTAATTCCTTTGAAAAGTTCATCAATAGTAGTTGATACTTCACTATTACTTGTGAATTCATG
DS548107_14778-40922_rc   AGCTCTAATTCCCTTGAACAATGCATTGATAGTAGTTTCTACTTCACTATTACCTGTAAATTCATG

                          15049
DS571148_75000-100159     ATAATGTGGTCCAAGTGAAACAAGTTTCAAAGCACTATATGTAATCTTTGAGTCTTTATTTCTATT
DS548107_14778-40922_rc   ATAATGTGGTCCAAGTGAAACAAGTTTCAAAGCACTACGTGTAATCTTTTGGTCTTTATTTCTATT

                          15115
DS571148_75000-100159     AAGAACAACAGTATGAACTGAAATATTCTTTCTATCAAATTCTGTTTCTGCATTAAATTCTGATCC
DS548107_14778-40922_rc   AAGAACAACAGTGTGGACAGTAATATTATTTCTATCAAATGCAGTTTCTGCATCAAATTCTGATCC

                          15181
DS571148_75000-100159     AAGTGCATTGTCCATTAACACAACCATAGTTCCTCTTTTGATTTTCTTGTCAGAAATCATTTTGAA
DS548107_14778-40922_rc   AAGTGCCTTGTCCATTAATACAATCATAGTTCCTCTTTTAATTGTCTTCTTTGAAATCTTATCAGC

                          15247
DS571148_75000-100159     TAAGTCATTCTTAGCAATATTAACAAGTGGATCAACTTGTCCTTGTCCTGCTGTGTATTTGACAAG
DS548107_14778-40922_rc   TAAATCTCTCTTAGCGATATTAACAAGTGGATCAACTTGTCCTGATCCTGCTGTGTATTGAGTAAG

                          15313
DS571148_75000-100159     TGTTTCATATTTCAAATCTTTTAATTTAATATTATCAGTTGCATATGAATCTCCTTTAAGATAATA
DS548107_14778-40922_rc   AGTATTATAATTTAAATTCTTCAAATCAATATTGGCAGTTGCATATGAATCTCCTTTAAGATAGTA

                          15379
DS571148_75000-100159     GATTGTTACTCCATCGACTGTTGTTAATTGACTTAAAACGGATAATTTTTCTTGAAGTGATTTAAT
DS548107_14778-40922_rc   CAATGTTACTCCTTTTACTGTAGTTAATTGGTTTAAAGTAGATAACTTCTCTTGAAGAGATTTAAT

                          15445
DS571148_75000-100159     GACTGTAGAATCAGTAATACCAAGAATAATTGCAAGATGTGTATTAACATTATTCTTATGAACATC
DS548107_14778-40922_rc   AACTGTAGAATCAGTAATACCAAGAATAATTGCAAGATGTGTATCAACATTATTCTTGTTAACATC

                          15511
DS571148_75000-100159     AATGATACATTTTGATGTAGTGTTTGTATTATATTGATCAATAAAGTCTTTATTCTCATTGTTAAA
DS548107_14778-40922_rc   AAGAATACACTTTGATGTAGTGTTTGTATTATATTGATCAATGAATGCTTTGTTTTCGTTGTTAAA

                          15577
DS571148_75000-100159     TGATCTGAATGTAATTGATCTTCCTCCAATGAAATCCATTCTACAAACATCATATGCTTCAGTTTT
DS548107_14778-40922_rc   TGATCTGAAGGTAATTGATCTTCCTCCAATGAAGTCCATTCTACAAACATCATAAGCTTCTGTTTT

                          15643
DS571148_75000-100159     TGTTGCATATGTCATTTTTGTATTTAAATAAACATAAGCCAATGGTCTTTTAGCTGCATATTTAAA
DS548107_14778-40922_rc   TGTTGCATAAGTCATTTTTGTATTTAAATAGACATAAGCCAATGGTCTTTTAGCTGCATATTTGAA

                          15709
DS571148_75000-100159     ACAAGTTTTTCCATTAATATCTACACTTTCAATTTGTTGTTCATCAACTGGAACATTGTCCCAATC
DS548107_14778-40922_rc   GCATGTTTTTCCGTTAATGTCTGCACTCTCAATTTGTTGTTCATCAACTGGAACATTATCCCAATC
```

```
                          15775
DS571148_75000-100159     AACTTCATATTTTTCAATTGGATATGCATATTCTTTTTGACAAAAATCTTGTTTATCAAGGTTTTT
DS548107_14778-40922_rc   AATTTCATATTTTTCAATTGGGTATGCATATTCTTTTTGACAAAAATCTTGTTTATCAAGAGTCTT

                          15841
DS571148_75000-100159     AATGTTATTCAATTGTTGAGCGTTAAGATTATGATCTTCATTGATAATATATTTATAACATTCTTT
DS548107_14778-40922_rc   AATATTAGCCAATTGTTGACTGTTAAGATTGTGATCTTCATTAATAGTGTATTTATAACAGTCAGT

                          15907
DS571148_75000-100159     ATTACCCTTGGTTGTTTCACAAGTAGTCCATACAAAATGTCTCCATGGTTGCATAGCTAAATAATA
DS548107_14778-40922_rc   ATTACCATT---TCCACTACAAGTAGTCCACACAAAATGTCTCCATGGTTGCATAGCTAAATAATA

                          15973
DS571148_75000-100159     GAAAACATCATATTGGTGAGTGTAAGAATGATACCATGAACCTGCATTCTTTCCACGAGACATAAT
DS548107_14778-40922_rc   GAAGACATCATATTGGTTGGTGTAAGAATGATACCATGAACCTGCATTCTTTCCACGAGACATAAT

                          16039
DS571148_75000-100159     ACCAAGGTCATAATAATCAATATCTGCTGAAAATTCATTAAGTTTATCTGCAAGACAACATAATAA
DS548107_14778-40922_rc   ACCAAGATCATAATAGTCAATGTCTGCTGAGAATTCGTTAAGTTTGTCAGCAAGACAACATAATAA

                          16105
DS571148_75000-100159     TAAGATATTTAATAATAATAATTTCATatcctgaatttttaaagttcattgagttgtttaaattat
DS548107_14778-40922_rc   TAAGATATTTAATAATAATAATTTCATatcctgaaaatttgaagttctatgagttgtttaaatttg

                          16171
DS571148_75000-100159     cttatttttccttttgtttttcagaacttgttctattctcttattttcttttc-aagagagtttat
DS548107_14778-40922_rc   ctaaactttcttttttttttttcagaacttgtcctattcccttcttttcttttcgaatgaaatatat

                          16237
DS571148_75000-100159     ttttggattat-taacacctaaaaacgaaattac-ttttttcaacttcttcctttttactcttttt
DS548107_14778-40922_rc   ttttggataataaaacaactaaaaacgaatttactttttttcaacttcttctttttttctcttttt

                          16303
DS571148_75000-100159     ttt--aaattatttcaaataataataaattatttcgaacttattttaaaaataaggaaattaaagt
DS548107_14778-40922_rc   ttttagagttatttgaaataattataaattatttcgaaattaaattaaaaataaggaaattagagt

                          16369
DS571148_75000-100159     aaaagtcatcaacgtttttcaaactttttttttgatttaaaaaaagaaaatattttaaa-ttaaaa
DS548107_14778-40922_rc   aaaagtcatcaacatttttcaaac-tttgttttgatgtgaaaaaagaaaatatttttagattaaca

                          16435
DS571148_75000-100159     aaaatacctttttcatattttattactttttgtttatttaaattaaaaattttaacatttttcaaa
DS548107_14778-40922_rc   aaaa-acctttttcttattttatcatttt---attgtttaagttaaaa--tgatgcatttttcaa-

                          16501
DS571148_75000-100159     taaaccaagttgttataaaatagaatttctttaatta-tattcgtcaccaacaaacattgttgagt
DS548107_14778-40922_rc   t---ccaa----------aaaacaatttccttaaatattattcttcaccaacaagcacagttgagt

                          16567
DS571148_75000-100159     tgagactaattcatgtcaactataattattacatagaaatttaatta-aattaaacaggatatcct
DS548107_14778-40922_rc   tgagattgattcattctaactatgctaattacaaagaaattaaattacaatt-------atatcct

                          16633
DS571148_75000-100159     aactaaaaataaatagtttcataatttaaatattttcgatgatgtgttcttcctttttgtaattta
DS548107_14778-40922_rc   gactaaaaataaataggttcataactt-aatattttctatgatgtgttgtt-cttttagtaattaa

                          16699
DS571148_75000-100159     ttacttcaatactccatttttttaatccttttcattttattttttattttttaaaaaaagtgtttt
DS548107_14778-40922_rc   t-----------tccattatttttatccttttcattttattttttcatttacaaaaactgttatta

                          16765
DS571148_75000-100159     aatattccttagacttattttttctaaaataacatta-ttttttcttttatctcttttcaactaa
DS548107_14778-40922_rc   aatattctatgaatttgttctctgtaaaataacaacatttttttctctttatctcttttctgttaa

                          16831
DS571148_75000-100159     ctctttctttaaaaaattatgaaaaatacatgtctgacattctattat-ttaataccctccccttt
DS548107_14778-40922_rc   gactttcttt---gagttatgaaaaatacaactc-aacattcaattatactgataccctc----tt

                          16897
DS571148_75000-100159     ttatttacaacaaagttaaataaact--------ggggatagtt-----------t---------c
DS548107_14778-40922_rc   ttatttacaacaaaattaaataaactcattgttgtggaatagttcctaatttatttgaataacacc

                          16963
DS571148_75000-100159     aatttatgacttaaagaaataatttttgttggtgttattaccatgttataatgtgatgacttatat
DS548107_14778-40922_rc   aagttatcagtcaaagaaatagtttttattggtattattaccatgttataat--------ttatat

                          17029
DS571148_75000-100159     atacttcaatggtataatatatgatttaatcttcctctcaaataagatatgatacatcatttgttt
DS548107_14778-40922_rc   atacttcaataatacagtacataatttaactatcctctaaaataggatttgataagtt-tgtgttt
```

```
                         17095
DS571148_75000-100159    attttatcttctccacagaataaataatggaataactgacttaatgttgaattaatatgtaggcac
DS548107_14778-40922_rc  atatcatcttttcaaaagaataaatgatggagtagctggattaatgtttagttaatatgtaggcac

                         17161
DS571148_75000-100159    ttatcccaaattcttttattgcattgaaagtacaaatgataatcgttccatttatcttaaagtact
DS548107_14778-40922_rc  tggaccaaggctcttgtgatataatgaaaaca-aactaataaccattccacttacctcaaa-----

                         17227
DS571148_75000-100159    tggtaaaggaagaagagttatttcttctcacaaaaacaagttaactcattgaattatttctaattc
DS548107_14778-40922_rc  ---tgaaggaaggggacttatttcttctaacaaagacatgtcagttcattgaattatttctgattc

                         17293
DS571148_75000-100159    tcattaaaacgaataat-aaatacatttgaa------------------atttttaacaaatgaaa
DS548107_14778-40922_rc  tcattaaaacaactaataaaataagttttaaagaataaactccatttgtatttttaataaataaaa

                         17359
DS571148_75000-100159    aa-ttattaaaaaa-aa-----------------------------ttgataaataaataataatt
DS548107_14778-40922_rc  aagttattgaaaaagaaagggaaaaattacattaatacaatgaaatttgataaataagtagtaagt

                         17425
DS571148_75000-100159    tgtgataaataagaataaagaagtgttaagataaaagacaaaaatgattgaa-taaatttcttcaa
DS548107_14778-40922_rc  tgtg---aataaaatcaaagac-ttttaaagtaaaagatgataatgattgaattaaatgtatttaa

                         17491
DS571148_75000-100159    aacaaaaag--------------ataaaaaaggaa-------------aa--gaag-aaatatatt
DS548107_14778-40922_rc  aacaaaaagaaataaagaataaaataaataagtaaataaaataaagataataaaagaaaaaacaat

                         17557
DS571148_75000-100159    ttaaaatgATTGAAAAATTGATTCAAATTAAG----AAAATAAATCAAAACTCATTTGCTTCTTTT
DS548107_14778-40922_rc  ttaaaatgattgaaaaaatgaatcaagttaagaaaaaaaataaaTCAAAACTCTTTAGTTTCATTT

                         17623
DS571148_75000-100159    ATTTTATTAAGAGAAGAAGTAGTAGTAGGATTTCGTTTGATGAACATTGATTGAGACTTAGTAAAT
DS548107_14778-40922_rc  GGTTTATTAAGAGAAGAAGCAGTAGTAATATTTCGTTTGATGAACATTGATTGAGATTTAGTAAAT

                         17689
DS571148_75000-100159    GAAGCTGACATAGGCATAACTTTCATTGATTGAGTTTCTCTTTTGGGTAATGGATGTAAAGGTTTT
DS548107_14778-40922_rc  GAAGCTGAAGTAGGGATATTTCTCATTGACTGAGTTTCTCTATTAGGTAATGGACGGAATGGTTTT

                         17755
DS571148_75000-100159    CTAATAGGTTTTTCATCTTGACTGACTAATTTAAAAGCATCATCAAAAGAATTTAATGAGTCAATG
DS548107_14778-40922_rc  CTAATAGGTTTTTCATCATGACCGACCAATTTAAAAGCATCATCAAAAGAATTTAAAGAATCAATG

                         17821
DS571148_75000-100159    TTATCCATTGTAACATCACCCATAACAGAAGGTTGTTTTTTATGAGTTAAAAGAATAGAGGATTTA
DS548107_14778-40922_rc  TTATCAATTGTAACATCACCCATAACAGAAGGAAGTTTTTTATGAGTTACAAGAAGAGGGGATTTA

                         17887
DS571148_75000-100159    GGTTTATCGTCTTGAGTTTTCTTTTGAGGTCTTTTTCTTTTTGGAGCTTCAGGTTTAGGTAAGAGA
DS548107_14778-40922_rc  GGTTTATCATCTTGAATTTTCTTTTGAGGTCTTTTTCTTTTTGGAGCTTCAGGTTTAGGTAAGAGA

                         17953
DS571148_75000-100159    TTTTTAAATAATCCAAGGTCTTGAATTAGATAAAGGACACTTGACCAAGAAATACTAAGAATATAA
DS548107_14778-40922_rc  TTTTTAAATAATCCAAGGTCTTGAATTAAATAAAGAACACTTGACCAAGAAATACTAAGAATGAAA

                         18019
DS571148_75000-100159    GAAATAACAGAGTCAATCAAATAAGCATAACCTATTTTATTTAATGAATAAATGATACCAATAATA
DS548107_14778-40922_rc  GAAATAACAGAATCAATCAAATAAGCATAACCGAATTTATTTAATGAATAAATAATAGCAATAATA

                         18085
DS571148_75000-100159    ATTAAAGTTAATGCAAGTAATGTAGGAGATTTAAATACATATACTATATTATCAAATGATAAAAAT
DS548107_14778-40922_rc  ATTAAAGTTAATGCAAGTAAAGTAGGAGATTTTAATACATATACTATATTATCAAAAGATAAAAAT

                         18151
DS571148_75000-100159    GCAATTAGAAGAATTAACCACATTGGAGTTTTTGAATGAACAGTAATAGCAGCAAGTTCTTGTTGT
DS548107_14778-40922_rc  GCAATTAAAAGAATTAACCACATTGGAGTTTTAGAATGAACAGTAATAGCAGCAAGTTCTTGTTGT

                         18217
DS571148_75000-100159    GCTCTGATAAAATTAGATTCAGCCATATTTTGAAATCCTTCATAAATACTAAGTCTTTCACTTAAA
DS548107_14778-40922_rc  GCTCTCATAAAATTAGCTTCAGCCATATTTTGAAATCCTTCATAAATACTAAGTCTTTCACTTAAA

                         18283
DS571148_75000-100159    GAAAAAAGAACTTTAGTTTGATCAATAGTTCCATTTTCTGGTTCTTCAATGATCATTTTTTTACCA
DS548107_14778-40922_rc  GAAAAAAGAACTTTAGTTTGATCAATAACACCATTTTCAGGTTCTTCAATAATCATTTTTTTACCA

                         18349
DS571148_75000-100159    GTTGCAGGGTTAATAGAAATAAAAGATAATTCATCATCTTTAGGATCCATTCTAATATAAGAAAAT
DS548107_14778-40922_rc  GTTGCAGGATTAATAGAAATAAAAGAGAAATTATCATCTTTAGGATCCATTCTAATATAAGAAAAT
```

```
                      18415
DS571148_75000-100159   AAGTCAAGAATTTTTTCAGCTTCTTCTTTAGCTTTAAAGAATGGTTTACTAAGATCTTCATTTTGT
DS548107_14778-40922_rc AAATCAAGAATTTTTTCAGCTTCAGCTTTAGCTTTAAAGTAAGGTTTACTAAGATCTTCATTTTGT

                      18481
DS571148_75000-100159   TTCCATTTTTTTGGTAAACCTTCATCGTCTAATCTAAAGATGGAAATAAATTTATTTTCCATTAAA
DS548107_14778-40922_rc TTCCATTTTTTTGGTAAACCTTCATTATCTAATCTAAAGATAGAAATAAATTTATTTTCCATTAAA

                      18547
DS571148_75000-100159   TTACTAACAAATCCTGGTCTTTCTAATATTGTAGATCTTACAGCATCAATAATATAAACTTGAAGT
DS548107_14778-40922_rc TTACTAACAAATCCAGGTCTTTCTAATATTGTAGATCTTACAGCATCAATAATATAAACTTGAAGT

                      18613
DS571148_75000-100159   TCATTTAATTTTTTTTCAACATCATCTTTATTCATTTTTAATGAATTAATCATTCCATTTTCTAAT
DS548107_14778-40922_rc TCATTTAATTTTTTTTCAACATCATCTTTATTCATTTTTAATGAATTAATCATTCCATTTTCTAAT

                      18679
DS571148_75000-100159   TCTTGAAGGTTTTGACTTGTAGTTTCAATAACAACTTTTCTAACTTGTTCCCACATATTTTGATTT
DS548107_14778-40922_rc TCTTGAAGATTTTGACTTGTAGTTTCAAGAACAATTTTTCTAACTTGTTCCCACATATTTTTGTTT

                      18745
DS571148_75000-100159   GATTGTTCAAATAAAGGAAGAAGAAGTGGTTTCATAATGTTTTGTAAATGCTTTTTAAAATGAGTC
DS548107_14778-40922_rc GATTGTTCAAATAAAGGAAGAAGAAGTGGTTTCATGATATTTTGTAAATGTTTTTTAAAATGAGTC

                      18811
DS571148_75000-100159   ATTAAAGCTTCAATAAGGTCTCTTCTTCCAATTTCATATAATTTATTAATAAATCTATCTAATGTA
DS548107_14778-40922_rc ATTAAAGCTTCAATAAGATCTCTTCTTCCAATTTCATATAATTTATTAATAAATCTATCTAATGTA

                      18877
DS571148_75000-100159   TTAATTTCTATTTCAAAATTATTTTCAATATTAGAAGGAACGCTTTGAGTTGATATTTTTTCCCAT
DS548107_14778-40922_rc TTAATTTCTATTTCAAAATTATTTTCAATATTAGAAGGAACACTTTGAGTTGATATTTTTTCCCAT

                      18943
DS571148_75000-100159   TCATTTTTAATTGTACTATTAAAATCATCCATTTGTTCTGCATATCCAGCATATTTTGTTGGATCA
DS548107_14778-40922_rc TCATTTTTAATTGTAATATTAAAATCATCCATTTGTTCAGCATATCCAGCATATTTTGTTGGATCA

                      19009
DS571148_75000-100159   AAAAGAGAAGATTGTTCTGTGATATATTGATTTTTTAAAGTGAAATAACTTGTAGTAAGCATTGTT
DS548107_14778-40922_rc AAAAGAGAAGATTGTTCTGTAATATATTGATTTTTTAAAGTAACATAACTTGTAGTAAGCATTGTT

                      19075
DS571148_75000-100159   TTAATATAGTTAATAGCAAGAGTCATTTGAGTTTGAAACAAATATGATATTTCATTTAACATTTTT
DS548107_14778-40922_rc TTAATATAATTAATAGCAAGTGTCATTTGAGTTTGAAATAAATATGAAATTTCATTTAACATTTTT

                      19141
DS571148_75000-100159   TCACTAAGAAGATCAGCTTTTTCTTTAACAACTCTATCGAGATATTTAGATGCAACATTCATAAAT
DS548107_14778-40922_rc TCACTAAGAAGATCAGCTTTTTCTTTAACAACTCTATCTAAATATTTAGAAGCAATATTCATAAAT

                      19207
DS571148_75000-100159   TCTTTCATTTTTTTATCATATAAACAATCACAAAATATCTTAAAATTATTAAAAATATGTTTTTCA
DS548107_14778-40922_rc TCTTTCATTTTTTTATCATATAAACAATCACAAAATATTTTAAAATTATTAAAAATATGTTTTTCA

                      19273
DS571148_75000-100159   GAATGTTGTAGTTTAATTTCTTCTATTTCTTTTTCAAATTCATTAAAAATATTTTCAATCAATTCA
DS548107_14778-40922_rc GAATGTTTTATTTTAATTTCTTCTATTTCTTTTTCAAATTCATTAAAAATATTTTCAATCAATTCA

                      19339
DS571148_75000-100159   TCACATCTAAATCTTGCTAACATTTCTTTTTGACTTGGAAGGTCAAGATCTTTATTAGATTTAATT
DS548107_14778-40922_rc TCACATCTAAATCTTGCTAACATTTCTTTTTGACTTGGAAGATCAAGATCTTTATTAGATTTAATT

                      19405
DS571148_75000-100159   GCACTCCATACTTGTTTTGTAAATAATGCTAATCCATCTGCTGGAATTTCTTTATTATAAATAGGT
DS548107_14778-40922_rc GAACTCCATACTTGTTTTGTAAATAATGCTAATCCATCTGCTGGAATTTCTTTATTATAAATACTT

                      19471
DS571148_75000-100159   CTAAAATAAGTATTTTGATTTTTACAATCAAATCTTTTTTTTAATTCTTTTGCTTCTTGAATAAAT
DS548107_14778-40922_rc CTAAAATAAGTATTTTGATTTTTACAATCAAATCTTTTTTTTAATTCTTTTACTTCTTGAATAAAT

                      19537
DS571148_75000-100159   AATTCTTTATCATGTTTAAAATGAGGTAATGATGTAAATTCTAAATCAAAAAACTTATCAATAGGT
DS548107_14778-40922_rc AATTCTTTATCATGTTTAAAATGAGGTAATGATGTAAATTCTAAATCAAAAAATTTATCAATAGGT

                      19603
DS571148_75000-100159   GCACGTTTAAAACATTCTGGGCGTGCAACACTTTCCCAAATTTTCATAATATCTTCTAATAATACA
DS548107_14778-40922_rc GCACGTTTAAAACATTCTGGACGTGCAACATTATCCCAAATTTTCATAATATCTTCTAATAATACA

                      19669
DS571148_75000-100159   CTTTTAGTATTTTCAAATGGTTTTCTTTGATCTCTATCACGAATAACAAAAAGAATTAATGTTTTA
DS548107_14778-40922_rc CTTTTAGTATTTTCAAATGGTTTTTTTTGATCTCTATCACGAATAACAAAAAGAATTAATGTTTTA
```

```
                           19735
DS571148_75000-100159      GGTGAATCTTCTTTTTGAAATAGTTGTAAATTAAGTTCAAATATATTCTTTAATAATGACATATTT
DS548107_14778-40922_rc    GGTGAATCTTCTTTTTGAAATAATTGTAAATTAAGTTCAAATATATTTTTTAATAATGACATATTT

                           19801
DS571148_75000-100159      GATGCTCCATATCTACCAACATCATGTGACCATATATTAACCATTAAAACACTACAAACTGATAAA
DS548107_14778-40922_rc    GATGCTCCATATCTACCAACATCATGTGACCATATATTAACCATTAAAACACTACAAACTGATAAA

                           19867
DS571148_75000-100159      CTAAATAATGATATTTTTCTTTCAAATGATAAATCATCTTCTCTTATTGATCCATCTGACCCTTCT
DS548107_14778-40922_rc    CTAAATAATGATATTTTTCTTTCAAATGATAAATCATCTTCTCTTATTGATCCATCTGATCCTTCT

                           19933
DS571148_75000-100159      AAATCCATAATTATAATATCTTTTCTATCCCCAACTAGTCCTAACCAAACTCCTCTTGTTGTTCTT
DS548107_14778-40922_rc    AAATCCATAATTATAATATCTTTTCTATCTCCAACTAATCCTAACCAAACTCCTCTTGTTGTTCTT

                           19999
DS571148_75000-100159      TGTCTTCCATTCTTTTCATTTAATACTGCAAAGTCTGTATCAAATAAATAATTTAATAATGTTGAT
DS548107_14778-40922_rc    TGTCTTCCATTTTTTTCATTTAATACTGTAAAATCTGTATCAAATAAATAATTTAATAATGTTGAT

                           20065
DS571148_75000-100159      TTTCCACTATTTTGTGGTCCAAGAATGCTTAACATATTATAATTAAATCCTAATTCTTTAAATTTT
DS548107_14778-40922_rc    TTTCCACTATTTTGTGGTCCAAGAATACTTAACATATTATAATTAAATCCTAATTCTTTAAATTTT

                           20131
DS571148_75000-100159      GTATTTTCTTGAATAAACTCTTCAAATGTGATTCTATCTTTTTGGTTTTCATCTGCAAAAATCCCT
DS548107_14778-40922_rc    GTATTTTCTTGAATAAATTCTTCAAATGTAATTCTTTCTTTTTGATTTTCATCTGCAAAAATTCCT

                           20197
DS571148_75000-100159      TCTTGATCAATAATTTGCATACATGGATAATTTTCTTTTTCTTTCTTTTCTTCTTCTTCTTTTATT
DS548107_14778-40922_rc    TCTTGATCAATAATTTGCATACATGGATAGTTTTCTTTTTCTTTCTT------TTCTTCTTTTATT

                           20263
DS571148_75000-100159      TCTTCTTCTTCAACTTTCATATTTTCTTTTTCTTCTTCTTTAAGTTCTGCTTTAATATTTTCTGCA
DS548107_14778-40922_rc    TCTTTTTCTTTAACTTTTATATTTTCTCTTTCTTCTTCTTTAAGTTCTCCTTGGATATTTTCTGCA

                           20329
DS571148_75000-100159      ATAATATCTTCATCTCTTGTTTTTATTAAATTAACTTTATTCATATCAATCCCTGGTGCTTTTTTC
DS548107_14778-40922_rc    ATAATATCTTCATCTCTTTTCTTTATTAAATTAACTCTTTTCATATCAATTCCTGGTGTTTTTTTC

                           20395
DS571148_75000-100159      TTTTCTGAATTATTTTCTACTTCGTCTTCTTCTTTTTTTAACATTTCTACTCTCAATTGGTTGTGA
DS548107_14778-40922_rc    TTTTCTAAATTATTTTCTATTTC---TTCCTCTTTTTTTAATTCTTCTACTTTTAATTGGTTATGA

                           20461
DS571148_75000-100159      AATACATCTGACTCTTGCATattctcc--ttattctttttaagtttctttcgttttgttttatact
DS548107_14778-40922_rc    AGTAATTCTGATTCTTGCATcttttccttttattctttttaagttttttt----------------

                           20527
DS571148_75000-100159      aaaaattatttttttttctttatttttttattaaaaaataaaaataattgaaattttc--tttttta
DS548107_14778-40922_rc    -----tta---tttattttttatttttattaaaaactaaaaataattgaaattttcttttttttt

                           20593
DS571148_75000-100159      gaattaggaata----ttcgtcattatt-gtcatcattatttattttctccttcccttcttcctct
DS548107_14778-40922_rc    gaattagggatatattttcttcagtattctttctcattatttattttatttttcccttcttcttct

                           20659
DS571148_75000-100159      tctcttTTAAATTTCTTTCATTAAGCCATTATCATTTTCTGGTGTTAATTCTTCTTGAACTTCATT
DS548107_14778-40922_rc    --tcttTTAAATTTCTTTCATTAATCCATTACCCCTTTCTTGTATTACTTCTTTTTGAATTTCATT

                           20725
DS571148_75000-100159      TCCTTCTTCTTTGGTAAATTCTTCTGTATTGATATTTGTTTGTTGGTCAATATCTTTTCTAATGTA
DS548107_14778-40922_rc    ATTTTCGTCTTTTATAAATTCTTCTGTATTGATACTTATTTGTTGGTCAATATCTTTTCTAATGTA

                           20791
DS571148_75000-100159      ATACAACACATAAGCATTCTTTGAAACAATATTTTCTTTTGTTCCTTCAATCTTTGAAGTGGTTGA
DS548107_14778-40922_rc    ATACAATACATAAGCATTCTTTGAAACAATATTTTCTTTTGTCCCCTCAATCTTTGAAGTACTTGA

                           20857
DS571148_75000-100159      GTCATTAAAATCATACCAATCATTTCCAACACGAGCAGATGCAACATAATGTCCACCACCAAGAGA
DS548107_14778-40922_rc    ATCATTAAAATCATACCAATCCTTTCCAACACGAGCAGATGCGACATAATGTCCACCACCAAGAGA

                           20923
DS571148_75000-100159      ACCCATATGATTAGTAACACCATATAAATTATAAATTGGAGGTGTTGTTGGGTCATAGTGTTTAAC
DS548107_14778-40922_rc    ACCCATATGATTAGTAACACCATATAAATTATAAATTGGAGGTGTTGTTGGATCATAATGTTTAAC

                           20989
DS571148_75000-100159      AAAACGAGTAAGATCTAATGACTCAATTGGGAAATCAACAAATGTAGTAATTTTATCACGAGAATA
DS548107_14778-40922_rc    AAATCTAGTAAGATCTAATGATTCAATTGGAAAATCAACAAAGGTAGTAATTTTATCACGAGAATA
```

```
                          21055
DS571148_75000-100159     TCCATTGCTATTACCAAATCTTTTTAAATGAATAATAAGTATTTGATTAGTACTCCAAAGATCAAC
DS548107_14778-40922_rc   ACCACAACTATTACCAAATCTTTTTAAATGAATAATAAGTATTTGATTAGTACTCCAAAGATCAAC

                          21121
DS571148_75000-100159     TTTTTTATGTGATGGTTGGTGTTCTTTACATTTAGAACAATACACTTTATTATTTTCATCCATTAA
DS548107_14778-40922_rc   TTTTTTATGTGATAATTGGTGTTCTTTACATTTAGAACAATAAACTTTATTATTTTCATCCATTAA

                          21187
DS571148_75000-100159     TTCTTCTACTTCAAATGCTTCAAAACATTCTTCAAGAGTAACAGGATCACCATCAATTTGATTATA
DS548107_14778-40922_rc   TTCTTCTGCTTCAAATGTTTCAAAACATTCTTCAAGAGTAACAGGATCACCATCTGTTTGATTATA

                          21253
DS571148_75000-100159     ACGATATGACAATCCTCTGTCATATAAATATTTAAATGTTCCACTATCAATACTTTGACATTGAAT
DS548107_14778-40922_rc   ATGATATGACATTCCTCTATCATATAAATATTTAAATACACCATTATCAATACTTTGACATTGAAT

                          21319
DS571148_75000-100159     AATATTTTCATGTTCTTCGTTAAATTCAAAATACCATTTTTTTTCTTCAATTTCTTGTTCTTTTTC
DS548107_14778-40922_rc   AATATTTTCATTTTCTTCTTTAAATTCAAAATTCCATTTTTTTTCTTTAATTTC---TTCTTTTTC

                          21385
DS571148_75000-100159     ---TAA------------------TTGTTCTTCTTGTTCTTTTTCTAATTGTTTATTATTAAAAAT
DS548107_14778-40922_rc   TTTAAGTTCATCAATTTCAATTTTTTGTTCTTCATGTTGATTTTCTAATTTTTTATTATTAAAAAT

                          21451
DS571148_75000-100159     TAAAGTACCATTATTTTGATTTTCTTTAATATTATTATTCATATTATCTTC---------------
DS548107_14778-40922_rc   TAATATACTATTATTTTGATTTTCTTTAATATTATTATTCATATCATCTTCATTATCATCATCATC

                          21517
DS571148_75000-100159     TTCATCTTCTTCATCTTCATGAATTTTTTTATTAGAAAGAATAGTTTTTGAGTCCCAAGCATTTAC
DS548107_14778-40922_rc   ATTATCTTCTTCTTCTTCATGAACTTTTTTATTAGAAATATTTATTTTTGGTTCCCATTCATTTAA

                          21583
DS571148_75000-100159     GTTGTTTTTATAAGTATCTTTAAATAATTCAAATATTTTTTCATCAACAATTTCTTTATTAATTGA
DS548107_14778-40922_rc   TTTACTTTTATAAGTATCTTTAAATAATTCAAATAATTTTTCATCAACAATTTCTTTATTAATTGA

                          21649
DS571148_75000-100159     TTTACCAAACACAATTGGTATTCTATATTGTCTATATGAAGCATATTTTATTAAACAATCATAAAC
DS548107_14778-40922_rc   TTTTCCAAATACAACTGGTATTCTATATTGTTTATAAGAAGCATATTTTATTAAACAATCATAAAC

                          21715
DS571148_75000-100159     AATAAAATCAGATTCATTGTCTTGTTTTTCAATAATACCATATTCCATGTCTTTTTCTAATTTACC
DS548107_14778-40922_rc   AATAAAATCTGATTCATCATCTTGTTTTTCAATAATACCATATTCCATATCTTTTTCTAATTTACC

                          21781
DS571148_75000-100159     ATTTGTTATTATTTTTTCAACCTTTTTTTCATATCCAAATTGTACAATAACAAAATTATTTGGATT
DS548107_14778-40922_rc   ATCTTTTATTATTCTTTCAATTTTTTTTTCATATCCAAATTGTACAATAACAAAATTATTTGGATT

                          21847
DS571148_75000-100159     TGTATTTAAACAAATATCTTTTGTTGATTCTTCAATAATTTCTTCAATGTTTTTTCCTTTAATATT
DS548107_14778-40922_rc   TGTATTTAAACAAATATCTTTTGTTGATTCTTTAATAATTTCTTCAATATTTTTTCCTTTAATATT

                          21913
DS571148_75000-100159     TATTTTAATTGTTTTAGTATTTTGATAATTCTTATCACAAACATAAATTGATACTGAGTTTTGTTT
DS548107_14778-40922_rc   AAGTTTAATTGTTTTAGTATTTTGATATTTCTTATCACAAACATAAATTGATACTGAATTTTGTTT

                          21979
DS571148_75000-100159     ATTTGGAATTGCAAGTGATAAGAATACATATGGATCAAATCTCATATTGGTATTATGACAAACATC
DS548107_14778-40922_rc   ATTTGGAATTGCAAGTGATAAGAATACATATGGATCAAATCTCATATTTATATTATGACAAACATC

                          22045
DS571148_75000-100159     ACAAATAACTTTACTTTTAAGTTGTGCTGCAAATAAGTCTGTAATAATTGAATCATTTCTTTGTTT
DS548107_14778-40922_rc   ACAAATAACTTTACTTTTAAGTTGTGCTGCAAATAAATCTGTAATAATTGAATCATTTCTTTGTTT

                          22111
DS571148_75000-100159     ATATCTATTCCATTCAGCCACAGCCCATTCATCATCTGGTTCATTATTATAATCTGGTGTTTCAAC
DS548107_14778-40922_rc   ATATCTATTCCATTCAGCTATAGCCCATTCATTATCTGGTTCATTATTATAATCAGGTGTTTCAAT

                          22177
DS571148_75000-100159     ATATGGTTTCTTTAAAACACGATTTAAATCTTCATGTATTCCATCAATTAAATATGCTACTAATTC
DS548107_14778-40922_rc   ATATGGTTTTTTTAAAACACGATTTAAATCTTCATGTATTCCATCAATTAAATATGCTACTAATTC

                          22243
DS571148_75000-100159     TTGTGAATCATGTTGTTGATATCCACTAAATTGTGGTGCAAATTCTCCAATAGCAAATTTAAATTC
DS548107_14778-40922_rc   TTGTGAATCATGTTGTTGATATCCACTAAATTGTGGTGCAAATTCTCCAATAGCATATTTAAATTC

                          22309
DS571148_75000-100159     TTTAGGAGAAATAGTAGAATATCCTGACCAATATTTTCTTAATAATTGACTCCATTGTTCAACTAA
DS548107_14778-40922_rc   TCTAGGAGTAATAGTAGAATAACCTGACCAATATTTTATTAATAATTGATACCATTGTTCAACTAA
```

22375
DS571148_75000-100159 TTTACCTGATGTTCCTAATGGATTATTTAAATTAATATCTTTTTTCCAATCATCTTTTTGAAAGAA
DS548107_14778-40922_rc TTTACCAGATGTTCCTAATGGATTATTTATATTAATTTCTTTTTTCCAATCTTCTTTTTGAAAAAA

22441
DS571148_75000-100159 TTGAACTAATGGAGTTGTATGAATTAAACATTGAATAGCAGAATTCATATAACATGTATTTCCTAA
DS548107_14778-40922_rc TTGAACTAATGGAATTGTATGAATTAAACATTGAATAGCAGAATTCATATAACATGTATTTCCTAA

22507
DS571148_75000-100159 ATTATGTAATCCACAAACACCTTTTATTCCATTTGGTTCTTCTTCATTTCTAATTCCATAATATCC
DS548107_14778-40922_rc ATTATGTAATCCACAAACACCTTTTATTCCATTTGGTTCTTCTTCATTTCTAATTCCATAATATCC

22573
DS571148_75000-100159 AGAATATTTTGAACTAACATTTGATTCATATGGATAATTTATATTTGGTATTGTTGGTTGTTTTTC
DS548107_14778-40922_rc AGAATATCTTGAACTAATATTTGATGCATATGAATAAATTGAATTTATCATTGGTGTTTGTTTTTC

22639
DS571148_75000-100159 AATAATTTGAACATGAACTTTATTTAATTCATTTGAATCAATAAGAATTGATGATATAATATTTTC
DS548107_14778-40922_rc AATAATTTGAATATTAACTTTATTTAATTCATTTGAATTAATAATAATTGTTGATACAATATTTTC

22705
DS571148_75000-100159 TAAATCTTCTACTTCTTCTTCTTCATTTTTCATTACTTTAACTTTATAATTTGTAAAGTTAACATC
DS548107_14778-40922_rc TAAATTTTCTACTTCTTCTTCTTCATTTTTCATTACTTTAACTTCATAATTTATAAATTTAATATC

22771
DS571148_75000-100159 AATTTGAAAGTCTTTTTGTAATTTCTCAATTGCTTTTGAAATAGTAATATTTTTATATATTTCTCC
DS548107_14778-40922_rc ATTTTGAAATTCTTTTTGTAAATTCTTAATTGCTTTTGAAATTGTAATATTTTTATATACTTCTTT

22837
DS571148_75000-100159 TATATATTCACTTTTATTTTCTTTATGATCAAGTCCAATAAAATGAAAAACAACAGTTAAATGTAT
DS548107_14778-40922_rc TGTACGTTCAATTATACTTTCTGTTTGGTTAATTCCAATAAAATGAAGAGTAATAGTTAAATGTAT

22903
DS571148_75000-100159 TGGAATACTTTCTTTAATAATATGTTCTTGTGTTTTATAAATATAAACTTTAGGTAATTTAGGAAA
DS548107_14778-40922_rc TGGAATACTTTCTTTGATAACATATTTTTGTGTTTTATAAATATAAACTTTAGGTAATTTAGGAAA

22969
DS571148_75000-100159 TTTGATATCTTCATCAAAAAATCCATAGAGTGTTTTTATCATTTTCTTATTCTTTTTACTAATTAA
DS548107_14778-40922_rc TATTATATCTTCATCAAAAAATCCATATAATCTTTTTATCATTTTCTTATTCTTTTTACTAATTAA

23035
DS571148_75000-100159 TAAATAATCTATGTTCTCTTTTACTTCTCCTTTGAGATATCCATTTTCATCACTTAATCCTAATGC
DS548107_14778-40922_rc TAAATAATCTATTCCTTCTTTTATTTCTCCTTTAAGATATCCATTTTCATCACTTAATCCCATTAT

23101
DS571148_75000-100159 TGTATCTATTTTAGGTAATTTATATAATTTTTCCATTTCTTGTTTATCTTGTTCATTTAATGAACT
DS548107_14778-40922_rc TGTATCTAATTTAGGTAATTTGTATAATTTTTCAATTTCTATTTTATCTTCTTCATGAAATGAACT

23167
DS571148_75000-100159 ATAAGCAACTGCTGCATCATGAATATTCTTTACAAATGTTATTGGTACAGCAAAATACTTTTTTGT
DS548107_14778-40922_rc ATATGCAACTGCAGCATCATGAATACTCTTTACAAATGGCACTGGTACTGCAAAATACTTTTTTGT

23233
DS571148_75000-100159 TTTACCATCATGGATACTATTCATATAATTTGTTGGAAATTTGTTCTCCCTTTTAAGATGAGTAAT
DS548107_14778-40922_rc TGTACCATCTTGTATCTCATCCATATAGTTTGATGGAAATTTTCCTTCTTTTTGAAGGTGACTAAT

23299
DS571148_75000-100159 TGCAACATAGAAGTCTGTGAAAGGATTTGTATTGAACCAATTCATacttatctaataatatttcta
DS548107_14778-40922_rc TGCAACATAAAATTCTGTGAAAGGATTTGTACTGTACAAATTCATactaatctaataataaatcag

23365
DS571148_75000-100159 tttcaagttcttctgttttaatctttttctttgaggattaactttaccattagaaaaaaagaaacg
DS548107_14778-40922_rc tttcaagttcttccgttttaatcttttcctttgaggattaactcttacttttg----aaagaaatg

23431
DS571148_75000-100159 agataaaaaaaatga--aaaaatgaaaaaataaaaaaaaataaaaaaaaataaaaatgaaaaatgaga
DS548107_14778-40922_rc aaataaaaaagaaaatctaaaatcaaaaaaaaaataaaaataaaaatca-aaaaaaataaaataaaa

23497
DS571148_75000-100159 agaaaagataatcagaaatactctttttcttttATGAATAATAATTTACCTAATAGTTATGAAATA
DS548107_14778-40922_rc tgaaaagataaacaaaattaa-ctttttctttgatg---aattatctatctaattgtcaagaaATG

23563
DS571148_75000-100159 AACAAAGAAATTGAAATGAAAGAAGAGGTTTTGTCTCCTAATATTCTTATTGAAAATAATAGTGAT
DS548107_14778-40922_rc AACAAAGAAATTGAAATGAATGAAGAGACATTATCTCCTAGTATTCTTATTGAAAATAATAATGAA

23629
DS571148_75000-100159 AATAATGAAGTATCATATTCTCAAATAACAACAAAACAACATAATTGGTATAAACGATTAACTGTA
DS548107_14778-40922_rc AATAATGAAATATCATATTCTCAAATAACAACAAAACAACATAATTGGTATAAACGATTAACTATA

```
                          23695
DS571148_75000-100159     ACAATTGTCTGTGTAATATTATTATTTAGTTATATATTTACTTATGTATTATTGAAAACAACACCA
DS548107_14778-40922_rc   ACAATTGTTTGTGTAATATTGTTATTTAGTTATATATTTACTTATGTATTATTAAAAACAACACCA

                          23761
DS571148_75000-100159     GATACAAAAGAAATTAAGAAAATATTGATAATAGGAGTAAGTGAAGTATTATTATTGGTAATTATC
DS548107_14778-40922_rc   GATACAAAAGAAATTAAAAAAATATTAATAATAGGTGTAAGTGAAGTATTATTATTAATAATTGTC

                          23827
DS571148_75000-100159     TTTAGTATTCAAACAATTTTATTATTTCCATTTGTTCAACAACGATTACATGAATCTTCAATTATA
DS548107_14778-40922_rc   TTTGGTATTCAAACGATTTTATTATTTCCTTTTGTTCAAGAAAGATTACATAAATCATCAATTATA

                          23893
DS571148_75000-100159     ACAAAAATAATGGGAAAAGTTTATTCAAAGTATAATTCAACAACATTTGTATTTAAATTACCCAAA
DS548107_14778-40922_rc   ACAAAAATAATGGAAAAACTTTATTCAAAATATAATTCAAAAAAATTTGTATTTAAATTACCTAAT

                          23959
DS571148_75000-100159     ATCAATAAAATGATTGAAAAAATATATCCACCAGTTCCAAATGAAAAAGTAAATAAAACGATATTA
DS548107_14778-40922_rc   ATTAATAAAATGGTTGAAAAATTATATCCACCAATTCCAAATGAAAAAGTAAATAAAACAATATTA

                          24025
DS571148_75000-100159     AATAGAATGATAGAAAAATATAGAAAATTTATTGACCAACATTTAGTTTCAATAAAAGTATTTTCA
DS548107_14778-40922_rc   AATAGAATGATAGAAAAGTATAGAAAATTTATTGAACAACATTTAGTTTCAATAAAAATATTTTCA

                          24091
DS571148_75000-100159     TTAATATTTTGTTGTATTTCATCAATTATAACATTAATATTAATTTATTTACTTATTCAACAAAAA
DS548107_14778-40922_rc   TTAATATTTTGTTGTATTTCATCAATTATAACATTAATATTAATTTATTTACTTATTCAACAAAAA

                          24157
DS571148_75000-100159     AATACTTTTAGGATTCTTTATATTCAAACATTTGTTATTTCTGTTTTAATTCTTTCAGTATTAATT
DS548107_14778-40922_rc   GATACATTTAGAATTCTTTATATTCAAACATTTGTTATTTCTATTTTAATTATTTCAATATTAATT

                          24223
DS571148_75000-100159     ATTGGATGTATTATAATAACAAAAATATTAGAAATAACATTGGTTTTATTAAATGCATTAATGTAT
DS548107_14778-40922_rc   ATTGGATGTATGATAATATCAAAAATAATAGGAATAATAATTATTATATTAAATGTATTAATGTAT

                          24289
DS571148_75000-100159     GTTTTATACAAAAGAATCATATTTTCTGCTTCATTATTTATTTCAATGAGTCTTATTGGAATAGTA
DS548107_14778-40922_rc   GTTTTATACAAAAGAATTATTTTTTCTGCTTCATTATTTATTTCAATGAGTCTTATTGGAATAGTA

                          24355
DS571148_75000-100159     ACATCTTTAATTGTTTCAGTAATTCCTAATTTAAAACCAAATTCAAGTAGTTTTTATTTGGTGAGT
DS548107_14778-40922_rc   ACATCTTTAATTATTTCAATAATTCCTAATTTAAAAACAAATTTAAGTAGTTTTTATTTAGTGAAT

                          24421
DS571148_75000-100159     TATTCAACAATAACATTCCTTGCTTTTCTTCTTTTTTTATTATCAGCACTTATTCATACAATTTTC
DS548107_14778-40922_rc   TATTCAACAACAATATTCTTTACTTTTCTTCTTTTTCTATTATCAATATTTATTTATACAATTTTC

                          24487
DS571148_75000-100159     TTATGTTGTAAAAATCCAAAAGAATGGATGAACGTATTAAAGAAATGTATAACATCATTATTTAAA
DS548107_14778-40922_rc   TTATGTTGTAAAAATTTAAAAGAATGGAAGAAAATATTAAAGAAATATATATTATCAATATTTAAA

                          24553
DS571148_75000-100159     CATCATTATATTGCTACATTAATCTTTTTCCTTATTATGTTAACATCAGGAATTACTACAATATTA
DS548107_14778-40922_rc   TATCATTATATTGCTACAACAATTTTTCTGTTTATTATGATAACATCAGGAATTATTACAATATTA

                          24619
DS571148_75000-100159     AGTTGGAGAGGAAGTATTCTCTATTTCAAATCAATTGTTGTTCCAGATTCTTTTAATTCAGTTGAG
DS548107_14778-40922_rc   AGTTGGAGAGGAACTATTCTTTATTTTAAATCAATTATTATTCCAGATTCTTTTAATGCAGTTGAA

                          24685
DS571148_75000-100159     AATAACTCTTCAATAAAAATTACAAATATTCCAATAATAACACGAGATATTGAAGTAATAACATTT
DS548107_14778-40922_rc   AAGAATTCTCCCATAAAAATTTCAAATATTCCAATAATAACAAGAGATATTGAAGTAATAACATTT

                          24751
DS571148_75000-100159     GGATGTGGAAAGAAGAATTATTATAGAGAATGTTATGCTAACCCAACTATCAAAACACCAACAATT
DS548107_14778-40922_rc   GGATGTGGAAAAGAGAATTATTATAGAGGGTGTTATGCTAATCCAACTATTAAAACACCAACAATT

                          24817
DS571148_75000-100159     GATATGTCTAGATTTATTGAATTATCTATTACATCTCAAGATTATTATCATTTGAATAGTAGAATA
DS548107_14778-40922_rc   GATATGTCTAAATTTATTGAATTATCTATTACATCTCAAGATTATTATCATTTAAATAGTAGAATA

                          24883
DS571148_75000-100159     ATTCCTTTAAATGGTCAAGTTTATTTCCCTAAAGATATTCTTAGATTAAACAAAACATCACATTTT
DS548107_14778-40922_rc   ATTCCTTTAAATGGTAAAGTTTATTTTCCTAAAGATATTCTTCAATTAAATAAAACATCACATTTT

                          24949
DS571148_75000-100159     AAATCAGTAATTATTCTACCACATCAACTTGGAAATCTTATTAATAGTGATGATGGATATGATTAT
DS548107_14778-40922_rc   AAATCAGTAATTATTTTACCACATCAACTTGGAAATCTTATTAATAGTGATGATGGATATGATTAT
```

```
                      25015
DS571148_75000-100159   CTTCAAAAGACATTTGCACAAAATGATTTGATTGGCATTGTCATTGATTCATCATTTTTTGATAAA
DS548107_14778-40922_rc CTTCAAAAAACATTTGCACAAAATGATTTAATTGGAATTGTTATTGATTCATCATTTTTTGATAAA

                      25081
DS571148_75000-100159   GATTTTAACGATAAAACCATTATTAGTAAAAATTATAATCTTACTACACAAGAAGCTTATATTGAA
DS548107_14778-40922_rc GATTCTAATGATAAAACAATTATTAGTAAAAATTCTAATCTAACAATACAAGAAGCTTATATTGAA

                      25147
DS571148_75000-100159   GCAAGAAGTATTTTAACATATAATATTCTTCAATATCTTAATGTTGCATTAAATAATTATTTTAAT
DS548107_14778-40922_rc GCAAGAAGTATTTTAACATATAATATTCTTCAATATCTTAATATAGCATTAAATAATTATTTTAAT

                      25213
DS571148_75000-100159   TTTACTGTTGACATGTCAGAAATTGGTATTGTTGGAGATAGAGAAGGTGGTGCAGTTGGGATACGA
DS548107_14778-40922_rc TTTACTATTGATATGTCAGAAATTGGTATTGTTGGAGATAGAGATGGTGGTACAGTTGGAATAAGA

                      25279
DS571148_75000-100159   CTAATAGAAGTAATGAATAGAAATAAGACGTTTCCAATATTATTAAAAGATTGGAACATTATAACT
DS548107_14778-40922_rc TTAATAGAAGTAATGAATAGAAATAATAAATTTCCAATATTATTAAAAGATTGGAATATTATAAAT

                      25345
DS571148_75000-100159   ATTAAAGTTATTTCATTTAGTGGATTGTCATGTAATCAAATAATAAAACATAATATAATGAATGAT
DS548107_14778-40922_rc ATTAAAATTACTTCATTTAGTGGATTATCATGTAATCAAACAATAAAAAATAATATATTAAATGAA

                      25411
DS571148_75000-100159   ATACAATCTTATTTTATTGAAACAATCCCATCTATCAGTACATATCCTAATCCTTTTTTATCAAGT
DS548107_14778-40922_rc ATGCAATCTTATTTTATTGAAACAATTCCATCTATTAGTATTTATCCTAATCCATTTTTATCAAGT

                      25477
DS571148_75000-100159   TATGCAATTTTTGAATCACCTGATTTAATATTTATTAATCAAACAAATATTTTAAATTCTAATCGT
DS548107_14778-40922_rc TATGTAATTTTTGAATCACCTGATTTAATATTTATAAATCAAACAAATATTTTAAATTATAATAAT

                      25543
DS571148_75000-100159   CCATTTTATTATACAGGTAGTTTATTTATTCAACGTGGAAATCCTAATGATTTTAATATAATTTAT
DS548107_14778-40922_rc TCATTGTATTATACAGGTAGTTTATTTATTCAACGTGCAAATCCTAATGATTTAAATATAATTTAT

                      25609
DS571148_75000-100159   GAAGAACCAAATAATCATATTATTCTTGATAATATTTTTCCAATACAATCAAATTATATTTCAAAA
DS548107_14778-40922_rc GAAGAACCAAATAATCATATTATTCTTGATAAAATTTTTCCAATTCAATCTAATTATATTTCAAAA

                      25675
DS571148_75000-100159   ACCCAACTTGATTATATTGTTTCTCTTTTCATTGGAAGTCATTTTATGTGTAGTTTAAATTCTAAT
DS548107_14778-40922_rc AATCAACTTAATTATATTGTTTCTCTTTTTATTGGAAGTCATTTTATGTGTAGTTTAAATTTTAAT

                      25741
DS571148_75000-100159   TGTATTAATTATAAGATGTTACAAGATTTTAAAATTGCTGATGAAGTTCTTCCTATGAATTCTGGT
DS548107_14778-40922_rc TGTATTAATTATAAAATGTTACAAGATTTTAAAATTGCTGATGAAGTTCTTCCTATGAATTCTGGT

                      25807
DS571148_75000-100159   TATTTCAATACATTTATAAGTAACAATGATATATTAATAGATTCTAATGGAAAGATTTCTCCTAAT
DS548107_14778-40922_rc TATTTTAATACATTTATAAGTAATAATGATATATTAATAGATTCTAATGGAAAGATTTATTCTAAT

                      25873
DS571148_75000-100159   ATTACTATTTCTACTAATTCTTCATATGTTGGACTTTATTATCAAAATGCTTATCAACGATTTCAT
DS548107_14778-40922_rc ATTACTATTTCTACTAATTCTTCATATATTGGACTTTATTATCAAAATGCTTATCAAAGATTTCAT

                      25939
DS571148_75000-100159   AGAAATCTTCAATGTCATAAGAAATGTTCTATTAAATATTTATTTAATGTACCAATCTATGCAAAA
DS548107_14778-40922_rc AGAAATCTTCAATGTCATAAAAAATGTTCTATTAAATATTTATTTAATATTCCAATTTATGCAAAA

                      26005
DS571148_75000-100159   GGAATAGAATTTAATTTTTATCAAGTTGGTAATTCATCAAATTATAATAATGCAATACTCATTCAT
DS548107_14778-40922_rc GGAATAAAATTTAATTTTTATCAAGTTGGTAGTTCATCAAATTATAATAATGCAGTATTTATTCAT

                      26071
DS571148_75000-100159   TCAAATAAAAATGTAAAAGAAATTGCTAAATTTAAATATGCAGGTATTCCACAATCTAAAACACCA
DS548107_14778-40922_rc TCAAATAAAAATATAAAAGAAATAGCTAAATTTAAATATGCAGGTATTCCACATTCACAATCATCA

                      26137
DS571148_75000-100159   TTTACATTGAGTAAAGTGTATTTTTTACAATTATTTGAATTTGTTTTAATACACAACAACAA---
DS548107_14778-40922_rc TTTACATTAAGTAAAGTATATTTTTTACAATTATTTGAATTTGTATTTAATACACAACAACAACAA

                      26203
DS571148_75000-100159   ---CCAATAGAAATTGATGAATTAGAGATTCTTTTGATGGTGAAATTTTATTAGATAATGTTGCT
DS548107_14778-40922_rc CAATCATTAGAAATTGATGAATTAGAAATTCTGTTTGATGGAGAAATATTATTAGATAATGTTGCT

                      26269
DS571148_75000-100159   GTTTTTAATTAAaacatttc-aagaaataaacaaataattaaaTTATTGACTATTTTTTAATGCAT
DS548107_14778-40922_rc ATTTTTAATTAAaagattttaaagaaataaacaaataattaaaTTATTGACTATTTTTTAATGCAT
```

```
                               26335
DS571148_75000-100159     TATGTAATATTTGTTTTGTATTTAATGAACGTAATTCAGCAATTCCT
DS548107_14778-40922_rc   TATGTAATATTTGTTTAGTATTTAATGAACGTAATTCTGCTATTCC-
```
