# Supplementary material for: Evidence of Gene Conversion in Genes Encoding the Gal/GalNac Lectin Complex of Entamoeba
Source: PLoS Negl Trop Dis. 2011 Jun 28;5(6):e1209. doi: 10.1371/journal.pntd.0001209 (PMC3125142; doi:10.1371/journal.pntd.0001209)
Supplement: Figure S3 — Nucleotide alignment of orthologous genome regions of E. histolytica and E. dispar used to estimate inter-specific divergence around the heavy chain lectin orthologues EHI_046650 and EDI_123980. (PDF) [file pntd.0001209.s003.pdf]

```
Alignment: /Users/garethweedall/Desktop/hgl2.fas
Seaview [blocks=70 fontsize=10 A4] on Fri Nov  5 15:22:27 2010

                         1
DS571321_2985-26705      aatgaagattctatgagacgaaataaaaatacaaaaagtagtttttattttcacaaaaaaagaagaaaa
DS550441_21198-48907     aatgaagattccatgagatgaaataaaaatgaaaaaagtagtttttattttcagaaaaaaagatgaaaa

                        70
DS571321_2985-26705      agaactacaaaagaatgattcacatcagaggATGACTGATGAACTTTCTCCATTATTCTTAATTAACAA
DS550441_21198-48907     ggaactacaaaagaatgattcacatcagaggATGACTGATGAACTTTCTCCATTATTCTTAATTAACAA

                       139
DS571321_2985-26705      AAATGAAAATAAAAAGAAAACAGTAAATAAAGAAGATATTGAGAATGGACAAAACCAAATTTCTAAAGC
DS550441_21198-48907     AAGTGAAAATAAAAAAAAGACCCTAAACAAAGAAGACATTGAGAGTGGACAGAACCAAAATTTTAAAGG

                       208
DS571321_2985-26705      GATTTTTCAACAAAAACGAAAAGTATTATTTGGTCAAAAAATAGAAGAAGAAAAAAGAAAAGAAGAACA
DS550441_21198-48907     AATTTTTCAACAAAAACGAAAAGTATTATTTGGTCAAAAAATTAAAGAGGTAAAAGGAAAGGAA---GA

                       277
DS571321_2985-26705      ACGTACTAAGTTTCAATCAAATAATTTTGCAAGAAAACTTTGTGATGATGAGTTTATTAGTCATGATTT
DS550441_21198-48907     ATGGACTAAATCTCAATCAAATAGCTTTTCAAGAAAACTTTGTAATGATGAATTTATTAGTCATGACTT

                       346
DS571321_2985-26705      AAAAGTGAGTACATTTAAACGATCTCAATCAACTCAATCAACCCCTCAAAAAAGAGTCTATTCATCAAG
DS550441_21198-48907     AAAAGTGAGTACATTTAAACGATCTCAATCAATTCAACCAACACCTCAAAAAAGACTAAACCCATCAAG

                       415
DS571321_2985-26705      AAGTCCTTGTTTAGTCGATTCAGTCTATTTTCAACAACAATCAATCAATTTAAAGATGAAACAATTAAA
DS550441_21198-48907     GAATCCTTGTTTAGTTGATTCAGTCTACTTTCAACAACAATCAGTTAATTTAAAGATGAAACAATTAAA

                       484
DS571321_2985-26705      ACAAAAAGAACAAAGTAAACAAATTTATTTAAATAGAGACGCAAATATTGGGACAACTGGTATTAAACA
DS550441_21198-48907     ACAAAAAGAACAAAGTAAACAAATTTATTTAAATAGAGATGCAAATATTGGTACAACTGGCATTAAACA

                       553
DS571321_2985-26705      ACCAATTAAAAGAACACCAGTCGATATTCAATCAACTCAATTTGAAGATACCATAGAGTCTATTGGATT
DS550441_21198-48907     ACCAATTAAAAGAACTCTACTTGATATTCAATCAACTCAATTTGAAAATACTATGGAATCTATTGGTTT

                       622
DS571321_2985-26705      AGAAGATTTAGAATGGGAAGAGCAACATACTTTAACAGAAGAAAGTTCTCCTATGAAAAGAAGAAGAAG
DS550441_21198-48907     AGAAGAATTAGAATGGGAAGACCAACATATTTTAATAGAAGAAACTTCTCCAATGAAAAGAAGAAGAAG

                       691
DS571321_2985-26705      TCCAATAGTCCCAATGTTTAAAAGTCAAATGAAAATAACAAAACTCCCTTTTACAAAATTTACACCTAA
DS550441_21198-48907     TCCAATAGTTCCCATGTTTAAAAGTCAAATGAAAATAACTAAACTTCCTTTTACAAAATTTACACCTAA

                       760
DS571321_2985-26705      AACAACTGAATTTCAACCAATTGCACCAATGAGTTGGATTCTTGAAGGAAAAGAACCAACAAGAGAAGA
DS550441_21198-48907     AACTAATGAGTTTCAACCAATTGCACCAATGAGTTGGATTCTTGAAGGAAAAGAACCAACAAGAGAAGA

                       829
DS571321_2985-26705      GATATCTTCTAATATTGAACTTTATTCAGATATGACTACTTTTATTTCAAACAAAGATTTATGGAGGAA
DS550441_21198-48907     AATATCTTCTAATATTGAACTTTATTCAGATATGACTACTTTTATTTCAAACAAAGATCTATGGAGAAA

                       898
DS571321_2985-26705      AAACAATTTATTAGATCATCTTTCATATTATGCTGATTCAGAAAGAATATCTGTATTAGCATGTACATG
DS550441_21198-48907     AAACAATTTATTAGACCATCTTTCATATTATGTTGATCCAGAAAGAATTTCTGTATTAGCATGTACATG

                       967
DS571321_2985-26705      GAATGTTAATCAATGCGTTTTTAGTCGAGGAGAAATTGATAGACTAACAAGTGGAATTAAAAACAAACC
DS550441_21198-48907     GAATGTTAATCAATGCGTTTTTAATCGAGGAGAAATTGATAGATTAACAAGTGGAATTAAAAACAAACC

                      1036
DS571321_2985-26705      AGATATTATTGTGATTGGGTTAGAAGAATTAGAAATGAGTTTTGATGCCATTATTACAGGAAAAAAATT
DS550441_21198-48907     AGACATTATTGTGATTGGATTAGAAGAATTAGAAATGAGTTTTGATGCTATTATTACAGGAAAAAAATT

                      1105
DS571321_2985-26705      TAGTGATAAATCAATTCAATGGGAAGCTTTAATACAAGAATCAATTAATAGAGGACAAAATACTTATAT
DS550441_21198-48907     TAGTGATAAATCAATTCAATGGGAAGCCTTACTACAAGAGTCAATTAACAGGGGACAAAATACTTATAT

                      1174
DS571321_2985-26705      TGAATTAGGATATTATCAACTTTGTGGAGTTGTGTTATATGTATTTTTTGATGAAAGATTAAAAAATCA
DS550441_21198-48907     TGAACTAGGATATTATCAACTTTGTGGAGTTGTATTATATGTATTTTTTGATGAAAGATTAAAGAGTCA

                      1243
DS571321_2985-26705      TATTACAGATGTTGGATATGGTGATATAAGAGTTGGTGCTATGAGTGGAAAATTAGCAAATAAAGGAGG
DS550441_21198-48907     TATTACAGATGTTGGATATGGAGATATGAGAGTTGGTGCTATGAGTGGAAAATTAGCAAATAAAGGAGG
```

```
                 1312
DS571321_2985-26705   TGTTGCTTATAGAATGAGAATATATAACTCAACAATATGTTTTGTTGTAAGTCATCTTGCAGCTCATCA
DS550441_21198-48907  TGTTGCTTATAGAATGAGAATATATGATTCAACAATATGTTTTGTTGTAAGTCATCTTGCAGCTCATCA

                 1381
DS571321_2985-26705   AAATTTTTGTGACAAGAGAAATGAAGATTGGAATGAGATAAGCAAAATGAAAATAAGATATTTTGATGT
DS550441_21198-48907  AAATTTTTGTGAAAAGAGAAACGAAGATTGGAATGAGATAAGTAAAATGAAAATAAGATATTTTGATGT

                 1450
DS571321_2985-26705   TGGTAGTGGTTGTAGAAAAGTAGTTGAACTTTTACAACATGATGTTGTTATTTGGATGGGAGACTTAAA
DS550441_21198-48907  TGGTAGTGGATGTAGAAAAGTTGTTGAACTCTTACAACATGATGTTGTTATTTGGATGGGAGATTTAAA

                 1519
DS571321_2985-26705   TTATCGTATTGATATGGATGATGTTGAAGTTAGAAAATGTATGAAAATGAAAAATTATCTTGAACTTAT
DS550441_21198-48907  TTATCGTATTGATATGGATGATGTTGAAGTTAGAAAATGTATGAAAATGAAAAATTATCTTGGACTTAT

                 1588
DS571321_2985-26705   AAAACATGATCAATTATTATATTGTATGCAATCAAATAAAGTTTTTAATCATTTTTGTGAAGCAGCAAT
DS550441_21198-48907  AAAACATGATCAGTTATTATATTGTATGCAGTCAAATAAAGTTTTTAATCATTTTTGTGAAGCAGCAAT

                 1657
DS571321_2985-26705   CAAATTTGCACCAACATTTAAGATTAAAATTGGTAAACAAGGAATGTATGAAGAGAATAGAATTCCATC
DS550441_21198-48907  CAAATTTGCACCAACATTTAAAATTAAAATTGGTAAACAAGGAATATATGAAGAGAATAGAATTCCTTC

                 1726
DS571321_2985-26705   TTGGTGTGACAGAGTATTATGGAAAACAGAAAATAGACATAATGTTGAAGTAAAAGAATATATGAGTCA
DS550441_21198-48907  TTGGTGTGATAGAGTTTTATGGAAAACAGAAAATAGACATTATGTAGAAGTAAAAGAATATATGAGTCA

                 1795
DS571321_2985-26705   TGAATTATATTGTTCTGATCATAAACCAGTAACGTGTTTTATGAGTATAGATTTACAAAGAATTAACAT
DS550441_21198-48907  TGAGTTATATTGTTCTGATCATAAACCAGTAACATGTTTTATGAATATTGATTTACAAAAGATTGATAT

                 1864
DS571321_2985-26705   TAAATTACAACAAACAGTAATAAATTATTTAGACAAAGTAGAAAAAATATATAGTAAAGTAGTTTGTCC
DS550441_21198-48907  TAAATTACAACAAACAGTAATAAATTATTTAGATAAAGTAGAAAAAATATATAGTAAAGTAGTTTGTCC

                 1933
DS571321_2985-26705   AAAAATTATAATTGAACCTTCTATTATTACTATAGACCAAATTAAATTATTTGAAAAATATCAATTTAA
DS550441_21198-48907  AAAGATTATAATTGAACCTTCTATTATTACTATAGACCAAGTTAAATTATTTGAAAAATATCAATTTAA

                 2002
DS571321_2985-26705   AGTACAATTAAAAAACATTGGAAAGTTTGGAACATGTTATGAAATAGAAAATGGTGATGGAAGAATTTT
DS550441_21198-48907  AATACAGTTAAAAAATATTGGAAAGTTTGGAACATGTTATGAAATAGAAAATGGGGATGGAATATTTTT

                 2071
DS571321_2985-26705   TAAAGATGAATGGCTTTCAATTAAGAAATGTGAAGGATTTATAGATATTTTAGAAGGAAAGGATGTTGT
DS550441_21198-48907  TAAAGATGAATGGCTTTCAATTAAGAAATGTGAAGGATTTATAGATATTTTAGAAGGAAAAGATGTTGC

                 2140
DS571321_2985-26705   TAGTATTGATTGTGAAATATGTATTGATGAAAAATATGTTTGGATGTATCAAGAACAAAATTCATTAAT
DS550441_21198-48907  TAGTATTGATTGTGAAATATACATTGATGAGAAATATGTTTGGATGTATCAAGAACAAAATTCATTAAT

                 2209
DS571321_2985-26705   GAAAGTTATTAAAATAAAATTAGGTGAAGGAAATGAATTTATTACTTTTAGTATTGTTTTGAAAACTAC
DS550441_21198-48907  GAAAGTTATTAAAATAAAATTAGGTGAAGGAAATGAATTTATTACTTTTAGTATTGTTTTGAAAACTAC

                 2278
DS571321_2985-26705   ACCAAATATCATTGGAATGAATTTAGAAACATTAAATAGACTTAATAAACCATTAATTTCTAATGATAA
DS550441_21198-48907  ACCAAATATCATTGGAATGAACTTAGAAACATTAAATAGACTTAATAAGCCATTAATTTCAAATGATAA

                 2347
DS571321_2985-26705   AATTCAAAAGAAATATCAACCAATTCCATTTTTTATTCCAAAAGAAATATATCGATTGATTGATTATAT
DS550441_21198-48907  AATTCAAAAGAAATATCAACCCATTCCTTTTTTTATTCCAAAAGAAATATATCGATTGATTGATTATAT

                 2416
DS571321_2985-26705   TATTCAACATTATGAACCTAATTGTTTTGTTAAAAAAGATCCGTGTAATTATCTTAAAGAACAACTTAT
DS550441_21198-48907  TATTCAACATTATGAACCTAATTGTTTTATTAAAAAAGATCCTTGTAATTATCTTAAAGAACAACTTAT

                 2485
DS571321_2985-26705   TGAAGTTATTCAATGTTTAAATAAAGAAAAAGAATTTATTTCTGGACCTATTCAATTGTATTGTGATGC
DS550441_21198-48907  TGAAGTTATTCAATGTTTAAATAAAGAAAAAGAATTTATTTCAGGACCTATTCAATTATATTGTGATGC

                 2554
DS571321_2985-26705   TTTATTATTAGTATTATCAGGACTTCATAAATGTGTTATTTTTTATTCTTTTACTGACTCTGACTTAGT
DS550441_21198-48907  TTTATTACTAGTATTATCAGGTCTTCATAAATGTGTTATTTTTTATTCTTTTACTGATTCAGACTTAGT

                 2623
DS571321_2985-26705   TTTAAATGACCAAACTCTGTTACAAGTAGTTAATTTCATTATCCCAGACGAATATAACCATCTATTTGT
DS550441_21198-48907  ATTAAATGACCAAACCCTTTTACAAGTAGTTAATTTCATTATCCCAGATGAATATAACCACCTTTTTGT
```

```
                          2692
DS571321_2985-26705   ATATTTAATCACTTTCTTAAAAAAACTGATTTGTCTTGGTGAAAATGAACTTCTTTTAATTTCTAGATT
DS550441_21198-48907  ATATTTAATTTCTTTCTTAAAAAAGATGATTTGTCTTGGTGAAAATGAAGTTCTTTTAATTACTAGATT

                          2761
DS571321_2985-26705   TACTCCTCCTATATTTCGGTGTTTTGATCCAAATAAGTTACTACCCTTTACTCACTTTCTTGAATATGT
DS550441_21198-48907  TACTCCTCCTATATTTCGATGTTATGATCCAACTAAATTATTACCTTTTACTCACTTTCTTGAATATGT

                          2830
DS571321_2985-26705   TCTTCATTCTCCTCTTTCTTTATTTTATCAATGActaatgtattattatttattacaaacttt---tct
DS550441_21198-48907  CCTTCATTCTCCTCTTTCTTTATTTTATCAATAAttaatgtattattatttattacaaactttctttct

                          2899
DS571321_2985-26705   tttttttt-ctttt-attcatcttgaaattatttgattttATGACAACTACTAAACCTCATGTTCAATT
DS550441_21198-48907  tttttttttcttttttttatctttaaattatttgaatttATGACAACTACTAAACCTCATATTCAATT

                          2968
DS571321_2985-26705   ACGGTTAGTTCGTCCTGAAGCTCTTAATCCTTCTCAACACTTATTTGCAGGAACAGCATCTTCATGGTT
DS550441_21198-48907  ACGGTTAGTTCGTCCTGAAGCTCTTAATCCTTCTCAACATTTATTTGCAGGAACAGCATCTTCTTGGTT

                          3037
DS571321_2985-26705   TGTTGAAGAGTGTTATTTATGTGCAGTTTTTAATTTTCCACATTGTCACTTTGTTTGTCGAACAGTCAA
DS550441_21198-48907  TGTTGAAGAGTGTTATTTGTGTGCAGTTTTTAATTTTCCAACTTGTCACTTTGTTTGTCGAACAGTCAA

                          3106
DS571321_2985-26705   AAATTTAGAAATGGTTGCTCCTGTTAATGTTGGTGATGTATTATGTTATTCTGGTACTTGTGAAATTGG
DS550441_21198-48907  AAATTTAGAAATGGTTGCTCCTGTTAATGTTGGTGATGTGTTATGTTATTCTGGTGTTTGTGAAATTGG

                          3175
DS571321_2985-26705   AAAAACTTCGATAATTGTTGATATGGTTGTTTCTCGTCAGAATGACAACAAGGTTGTAACCAAAGGACA
DS550441_21198-48907  AAAAACTTCAATAACTGTTGATATGGTTGTTTCTCGTAATTGTGACAATAAAGTTGTAACCAAANNNNn

                          3244
DS571321_2985-26705   AGTTGTCTTTGTCAATATCAATTCTGAAGGAAAAGCAACTCCACACAATATTGTTTTGCAATAATcaaa
DS550441_21198-48907  nnnnnnnnnnnnnnnnnnnnnnnnnnnAGGAAAAGCAACTCCACATAATATTGTTTTGCAATAATCAAA

                          3313
DS571321_2985-26705   ttgtcatttttattttttgacttcttta-atgtttaaaataaggtaaaaagatagtaaaataaattattt
DS550441_21198-48907  TTGTCATTTTATTTTTTGACTTCTTTACAAATTTAAAATAAtgtaaaaagatagtaaaataaattattt

                          3382
DS571321_2985-26705   gtttacgatttaaccataaaaactagaaagatctatgaaaggaaaaaagaaaaaattactttttgaat
DS550441_21198-48907  atttacgatttaaccataaaaactagaaa-atctatgaaaggaa-------------------------

                          3451
DS571321_2985-26705   atttttagaaaagaaaaaattaataagaaacttcaaaaatttttttttaagtctcaATGAGCGAAACAA
DS550441_21198-48907  --------aaaagaaaaaattaataagaaacttcaaaaa---ttttttaagtctcaATGAGCGAAACAA

                          3520
DS571321_2985-26705   GTGAAGACCATTCAACATTTTATGATTTTCCATTACAAAAAATAGTTATATCTATTGAATACCTTGTAA
DS550441_21198-48907  GTGAAGACCATTTAACATTTTATGATTTTCCATTACAAAAAATAATTATGTCAATTGAGTATCTTGCAA

                          3589
DS571321_2985-26705   TTGCAGTGTTATGTTTTATTCAAAGTCTGAGAATTGTTAAAAATCAgtttgtcattattattttttttt
DS550441_21198-48907  TTGCAGTGTTATGTTTTATTCAAAGTATGAGAATTGTTAAAAATCAgtttgtcattattattttttttt

                          3658
DS571321_2985-26705   tttgttatt-tttaaataaaaaaacgaaagagaaaaaaaaag-ta-tcttatcacatagTCATAAATGTT
DS550441_21198-48907  gttgttttatttaagtaaaaaatgaaagagaaaaaaagtgatattcttatcatatagTCATAAATGTT

                          3727
DS571321_2985-26705   GTGGATTTCAAAACCTTCTTATCATATTTTTAATCTGTTGGTGTTTATTTAGAATATCAGATTTGATGA
DS550441_21198-48907  GTGGATTTCAAAATCTTCTTATTATATTTTTAATCTGTTGGTGTTTATTTAGAATATTAGACTCAATGA

                          3796
DS571321_2985-26705   TAAGATTCTTTCTGATATTATTTACTCACCAACCAGATGATTTTATTGACCAAACAACTTCTGTTGAAT
DS550441_21198-48907  TAAGATTCTTTCTGATATTATTTACTCCTCAACCAGATGATTTTATTGACCAAACAACCTCTGTTGAAT

                          3865
DS571321_2985-26705   GGTTGATTTCTGAgtttgttctatttctttttcaaataaaac---ataacaccttatttgtaaataatt
DS550441_21198-48907  GGTTGATTTCTGAgtttgttgtatttctttttttaaataaaataaaataacaccctttttataaataatt

                          3934
DS571321_2985-26705   atttattgaattagCATTCCTAGTTTTGTAATGGTTGCTCTTCTTTCATTCTTCATTTATTATTATTTA
DS550441_21198-48907  atttattgcattagTATTCCTAGTTTTGTAATGGTTGCTCTTCTCTCATTTTTCATTTATTATTATTTA

                          4003
DS571321_2985-26705   TTCCTTATTCATACTTCTCATTGGCAATATGCCAAATTAGTTTCAATTGTTATCATTATAACAATCAAT
DS550441_21198-48907  TTCCTTATTCATAGCTCTCATTGGAAATATGCCAAATTAGTTTCAGTTATCATTCTTATAACAATAAAT
```

```
                          4072
DS571321_2985-26705    CTCTTTCATATCTTTTCATTAATATCAATTGATATTTCATTAATGATAAAGCCAACTACAGCAGCAATT
DS550441_21198-48907   CTCTTTCATATCATTTCATTAACATCAATCGACATTTCATTAATGGTTAAACCAACCACAGCAGCAATT

                          4141
DS571321_2985-26705    CATTTAACTTTTTGCTGTAGTTCATTTGTATATTTATTAGTTGTAGGAGGGTTATTGTATGTATCTGTT
DS550441_21198-48907   CATTTAACTTTTTGTTGTAGCTCATTTGTATATTTGTTTGTTGTAGGAGGGTTGTTGTATGTATCTATT

                          4210
DS571321_2985-26705    CGTATTTTTTTCATTAATCCACATGTTTTAGAACAATCTCATCCAAAACCATCAGCAATGGGAGTTTGT
DS550441_21198-48907   CGTATTTTTTTCATCAACCCACATACTTTAGAACAATCTCATCCAAAACCATCAGCAATGGGAGTTTGT

                          4279
DS571321_2985-26705    GGTTTAGTTATTTCTTTAGCAATCTCAACCCGTTTAATATATGATTTTTCAAGTGCTATTGTAGGTCAA
DS550441_21198-48907   GGTTTAGTTATTTCTTTAGCAATTTCAACTCGTTTAATATATGACCTTTCAAGTGCAATTGTAGGTCAA

                          4348
DS571321_2985-26705    TCCATTCTGTCACTCCTTTGTTCAAACGAAAGTTCATTTTGTTCTGAACATTTTGGAATAGTTGTTTTC
DS550441_21198-48907   TCTATTTTATCACTCCTTTGTTCTCAAGAAAGTCCATTTTGTTCTGAACATTTTGGACTTGTTGTGTTC

                          4417
DS571321_2985-26705    TTTTCTCAAATTATTGAGGTTGTTTTAATGTCATTATGGGAAATTGTTCCATTAACAGCATTAGTGATT
DS550441_21198-48907   TTTTCTCAAATTATTGAGGTTGTTTTAATGTCATTTTGGGAAATTGTTCCATTAACAACGTTAGTAATT

                          4486
DS571321_2985-26705    TTATTCTGGAATATCCCTTCGAATAAGTCATATGACCCATTCCCTCACTTCCAATATCTTGTTGAAGAA
DS550441_21198-48907   TTATTCTGGAACATCCCTTCGAATAAATCATATGACCCATTCCCTCACTTTCAATATCTTGTTGAAGAA

                          4555
DS571321_2985-26705    ACAGATGATTCATCATACGTTACATATGATGCAACTACTCCTTTG---ATTTAAaaacatcaataagtg
DS550441_21198-48907   ACAGATGATTCTTCATACGTCACATATGATGCAACTACTCCTTTGATGATTTAAAAACATCAATAAGTG

                          4624
DS571321_2985-26705    tattg-tttttttttgtttatttt------gaatattatacttgaatgatatttagttttactgaatat
DS550441_21198-48907   TATTGTTTTTTTTTTGTTTATTTTAAttttgaat--taaaaattgaatgatattt-tttgtactgaatct

                          4693
DS571321_2985-26705    ttttagtgaaataatactcaaaacaaaaaaagttatataacatttgttttttttttttcactatctttc
DS550441_21198-48907   tttaagtaaaataatacttcgtacaacaaaatctatatactatttattttgtctt------tatcgttc

                          4762
DS571321_2985-26705    ttggtattacaataaatttacaccgtgactttctaactctttaaatattataaaagaagaaacaagaaa
DS550441_21198-48907   tcaatattccaataaatttatattgcta-ttt-taactctttaaatcttattaaagaagaacctagaaa

                          4831
DS571321_2985-26705    aattataagattatagagattctaaataaatactt-aaaagattaaatattgtctaaaaac--------
DS550441_21198-48907   tattataagattataaagattct--ataaacacttaaaaagattgaaca-tatctaaaaactatgttta

                          4900
DS571321_2985-26705    -----------tatttgttataattta------------------------------------------
DS550441_21198-48907   attataaaacatatttattgaaatttaacaaaaaattagtattaaataggtcattcattatattatatg

                          4969
DS571321_2985-26705    ---------------------------------------------------------------------
DS550441_21198-48907   aatataagcatagttaaagaattgataaaaatatttattactgtttttttgagataaagaaaggagtta

                          5038
DS571321_2985-26705    ---------------------------------------------------------------------
DS550441_21198-48907   ttgtagatatagtatgacgtaaattgcaaattatgacttacaaaaaaagaagatttttgattagttatt

                          5107
DS571321_2985-26705    ---------------------------------------------------------------------
DS550441_21198-48907   aaaattaaacaaatagtgtaatgaagttatttgatgaaagaaaataaaagtaaaagtattaacagtcta

                          5176
DS571321_2985-26705    ----------gttaaaat---------------------------------------------------
DS550441_21198-48907   aatggtatgtgttaaaataagaattacttctacctacgtgtttactgctgtaacaataaaacttatata

                          5245
DS571321_2985-26705    ---------------------------------------------------------------------
DS550441_21198-48907   tttataaattgtcattgatataatcaaataattttcttaatataatgataattaaacaattattctgaa

                          5314
DS571321_2985-26705    ---------------------------------------------------------------------
DS550441_21198-48907   tatattacttgtaaggatttgaaataaaataaatttaaataattcattttttttttatgatttttaaat

                          5383
DS571321_2985-26705    -----------------atattctaatagaatgagaaatgttataattaaacaaatcaactcattaaca
DS550441_21198-48907   caacttcttttgatgagacattctaatagaataagaattgttatttttagataaacagaacaattaaaa
```

```
                    5452
DS571321_2985-26705   ttgagataagttatttttaaataaataaacaaattttattatccatttataaaatcaataggcattatt
DS550441_21198-48907  taaagataagttatttttaaataactacaacagtttttttatccatttataaaatcaatgggtgttatt

                    5521
DS571321_2985-26705   tttatatttataatactcattctcctttct-cataataaaataaaatataactaattcaattcacata
DS550441_21198-48907  tttatatttagaatactcatccccttttcttacaaaaaaaaatgaaatataaataatactattcacata

                    5590
DS571321_2985-26705   ttataatgctttaaataattcaatttaaattaaaatattcataattcattgaattagatgaatatttttt
DS550441_21198-48907  ttataa-acattaaaaaactcaatttaaatcaaattattcataatttattgaattaggtaaatacttttt

                    5659
DS571321_2985-26705   gggttttgagaatcaaaaaac-aaatactatttgtatagtttgaaaactattaactttaattaaaacaa
DS550441_21198-48907  gggttttgtgaatcaataagtaaaatattatttttagcctttgtaaactacta--tttcattaaaatct

                    5728
DS571321_2985-26705   cttatatatttatagaattattctatttgaacaattgattagaatgatctattttgttttaataaataa
DS550441_21198-48907  catatgtgtttatagaattattctatttatataattgattagaatgatctatttcgttttattaaataa

                    5797
DS571321_2985-26705   aaatttcaaaat-aaaaaattataataaaaatatattattaaaaaactacttttttatattaaaagaaaa
DS550441_21198-48907  taacctcaaaacgaaaaagttataataaaaaaattactattgaaaaacta-gttttatattaaaggaaaa

                    5866
DS571321_2985-26705   atcaaaacttttt-gattttcacttcaaattattattaatttcattattcacatcattctt-a-atttaa
DS550441_21198-48907  gtcaaga-ttttcgattttcgtttcaaagaattattgacctcattgtccacgtcattcttgatatttaa

                    5935
DS571321_2985-26705   acgatacgttatctaaaaaatcgtattattataatatttgctatttcatttacgtgtataatatatatca
DS550441_21198-48907  atgattcgttatctaaaaaaacatattaccataATGCCTGCTATTTCATTTACACGGATA--ACACGTTC

                    6004
DS571321_2985-26705   aataaacactctccttccaaaacttaat-caattacctcaaaactcttttgggaatagttgtgttag--
DS550441_21198-48907  AACAGACACTTTACTTCCAAAA-TTAATTCAATTACTTCGAACTTCTTTTGGGGATAATCTCGTTAGAG

                    6073
DS571321_2985-26705   ---------------aacaagttattaaaacacagaaaaaatcatggttttattg-agtaaaatatatt
DS550441_21198-48907  GTACTTCTCAACTTAAACAAGTTATTGACACACA-ACCAAATCATTATTTTATTTCAGTAAACTATAAT

                    6142
DS571321_2985-26705   ggaaaagttgcaagacttgttatttattggcatttgatgaaatagttccatactatagaaaatattgca
DS550441_21198-48907  GGAAAAATTGTAGGAGTTGTTGTTTATTGGTATTTGATGAAACAGTTTCATTTTATAGAGCATATTGCA

                    6211
DS571321_2985-26705   ttccttccagagttgagaaacgaaaaagaagaataacagataattgaacatttattcaaaacatttggt
DS550441_21198-48907  ATCTCTCCAGAGTTCAGAGGTGAAAAGATAGGACAACAAGTAATTGAACATTTATTCAAAACAATTGGT

                    6280
DS571321_2985-26705   agtttgtgtgttttttgaagttgaatcaactgaagatgagactcatcatctattaagaaaatggtatatt
DS550441_21198-48907  GGTTTGTGGATTCTTGAAGTTGAACCAACTGAAGATGAAGTTCATCATCGGTTAAGAAAATGGTATTAT

                    6349
DS571321_2985-26705   caaaatgg-tttttggttacagacaaaaattatagacaacaatcatactcattaaaggaacattcaatc
DS550441_21198-48907  CGCAATGGATTTTCAATTATAGATAAAAATTATAAACAACCATCATACTCATTTGGAGGACAATCAATC

                    6418
DS571321_2985-26705   cctttatgaactatggcaacacacagctttcaaac-aggttctgttaacctttatttttgtactcaaa
DS550441_21198-48907  CCCCTATGGATTATGGCAACACAACCCCTTTCAAACAAGGTTTTGTCAACCTTTATTTCTGTACTCAAA

                    6487
DS571321_2985-26705   catag-gtatatgagtcttattattgtttgaataatttctagttttattttgtttatattaatttttaa
DS550441_21198-48907  CATAATGTATATGAAGCTCATTATAGCTTGAACCGTTTCTAGttttattttatttataatattttttaa

                    6556
DS571321_2985-26705   gtgaTTTCTTAATGGAAGTATTCAATTGTTTTATAAAAG-CTAATAAATGGTTGAACTCTTATGAAAAG
DS550441_21198-48907  ttgaattctaaattgaagtattcaattgttttataaaaatttaataaatgactgaactcttttgaaaag

                    6625
DS571321_2985-26705   ACGTCTACAAAGCAGAGAGTTCTAATGCATGTTGTTTAGCAGATTCTTTCATATTTTTTCTCATTTCCT
DS550441_21198-48907  acgtCTACAAAGCAGCGAGTTCTAATGCGTGTTGTTTAGCAGATTCTTTCATATTTTTTCTCATTTCTT

                    6694
DS571321_2985-26705   TTTCAAGCTTTTTTTGTCGTGCTTTTGCTTTGTTACACAATATATCGGTAAAGCAATCAACAAATCTAC
DS550441_21198-48907  TTTCAACCCTTTTTTGTCGTGCCTTTGCTTTGTTACACAAGATATCAGTAAAACAATCAACAAATCGAC

                    6763
DS571321_2985-26705   ATGTCATTGTACAAACCATACAAACACTTACAACAACTAACACAGAAAAGATGATGAAAATAAGCAATG
DS550441_21198-48907  ATGTCATTGTACAAACCATACAAACACTTACAACAACTAACACAGCAAAGATAATGAAAATAAACAATG
```

```
                  6832
DS571321_2985-26705    ATTTTGTAGGCATTAATAAATCAAGAAATGAATCAAATGTAAATGTCCCACGTTTTAATGTCCCTATCA
DS550441_21198-48907   ATTTTGTAGGCATTAACAAATCAAGAAATGAGTTAAATGTAAATGTACCACTTTTTAATGTTCCTACCA

                  6901
DS571321_2985-26705    TACATTCTTTAGTTTCTCTATTACAGTCATACTGACATTTCTTACAAATACATTCTTCTTTTGATTGAA
DS550441_21198-48907   TACATTCTTTAGTTTCTCTATTACAATCATATTGACATTTCTTACAAACACATTCTTCTTTTGATTGAA

                  6970
DS571321_2985-26705    TTATGGAATATTCTGCTTTTAATGTTATTGGTGATTCTATGTATATTTCATGAGTTGTTTTATATACTT
DS550441_21198-48907   TTATAGAATATTCTGCGTTTAGTGTTATTGGTGATTCTATGTATATTTCATGCGTTGTTTTATATACTT

                  7039
DS571321_2985-26705    TAATCTCTTCATATAACCGTTCTGTATGGAAAATTTGTATTGTTTCATTATCTTGTAATTGAATAGGAT
DS550441_21198-48907   TAATCTCTTCATAAATTCGTTCTGTATGGAAAATTTGTATTGTTTCATTACCTTTTAATTGAATAGGAT

                  7108
DS571321_2985-26705    ATAAGTGCTTTCCTTTATTAATGACAAATTGATGTATTTCCATTGCATTGCAAACAATAAATAAAAGAC
DS550441_21198-48907   ATAAATGCTTTCCTTTATTAATGACAAACGGGTGTAGTTCAAATGCATGACAAACAACAAATAAAAGAC

                  7177
DS571321_2985-26705    TAACACTAATAAACCAAGTCATtatacttgttattcagatgattcttaaaacaaaactcgtgtcaaatt
DS550441_21198-48907   TAACACAGATAAAACAATTCATtatacttgttattgagatgagtcttaaaacaaaattcgtgtcaattt

                  7246
DS571321_2985-26705    ctctttactatttcatagtatttatttcttctattaaactatacttcaataaaacaaaaattgaacatt
DS550441_21198-48907   ctctttagtatatcatataatttatttcctatattaaactatacttcaataaaacaaaaattaaaaatt

                  7315
DS571321_2985-26705    aaataa-----ttttttgttgtttaaattagaatgaaaaaaaaaattttaaacgttttatttatatgat
DS550441_21198-48907   aaataattacttttttttgttgtttaagtt----tattaaaaagtattttaaatattatgtttatgtgat

                  7384
DS571321_2985-26705    atgtttggtagttataattttcttgctatcatttactattgaattccttcttattaaattaccaatcat
DS550441_21198-48907   atatttgttagttataattttctttctatcatttactgttgtactagtccttattatattatcattcat

                  7453
DS571321_2985-26705    tttatttcatttaacataagtatgttaattat-------------------tctttctttatggattga
DS550441_21198-48907   ttaatttcatgtaacataaacatgttatttattatttattatttccttttgtcttattttgcgtatagg

                  7522
DS571321_2985-26705    tttaaattgattaaagcatttgttgaataacaaaaaatattcagaaattaaaaaacttattacgtaatt
DS550441_21198-48907   tttaagctgatataaacatttgttgaagaacaagaaatgttcagaaagtacaaaacttattatctact-

                  7591
DS571321_2985-26705    ttcaaaagtacaaatgactgtttttgataaaattctctggtac--aaataaaatgtattccatttcaca
DS550441_21198-48907   -tc-aaagaacaaa--attg-ttatgataaaattctttggtacataaataaaaagcaccccatttcaca

                  7660
DS571321_2985-26705    acaacatatttgttaagagaaaactaatagttttaaacaaacagagtgatattccaaatactcaaaaag
DS550441_21198-48907   ata--ttatttgtgaagagaaaaataacatttttcaataaagtgagtgatgttccaattacctaaaagg

                  7729
DS571321_2985-26705    caattggttcaacaagg-tgaattttcttattttctttacaaaaacaaataacacattcaaatatgaac
DS550441_21198-48907   taattagttaaaca-ggatgtattttattattttctttataaacacaaacagtgtattcgaatataaac

                  7798
DS571321_2985-26705    aattagttacttgactcaataaatatttatttatatttttatttctttgatacatagtaaaaagaagaa
DS550441_21198-48907   agtctatcacccgattcaataaat-tttatgtgtgttattattt-tttaatacaaattaaaaagaaata

                  7867
DS571321_2985-26705    acttcaacacaaacaactgcaatttattttatttttttaagnnnnnnnnnnnnnnnnnnnnnnnnnnnn
DS550441_21198-48907   gttttaaaatgaataagtaagatttattttgtttaactaggnnnnnnnnnnnnnnnnnnnnnnnnnnnn

                  7936
DS571321_2985-26705    nnnnnnnnnnnnnnnnnnnnnnnnnnnnnnnnnnnnnnnnnnnnnnnnnnnnnnnnnnnnnnnnnnnnn
DS550441_21198-48907   nnnnnnnnnnnnnnnnnnnnnnnnnnnnnnnnnnnnnnnnnnnnnnnnnnnnnnnnnnnnnnnnnnnnn

                  8005
DS571321_2985-26705    nnnnnnnnnnnnnnnnnnnnnnnnnnnnnnnnnnnnnnnnnnnnnnnnnnnnnnnnnnnnnnnnnnnnn
DS550441_21198-48907   nnnnnnnnnnnnnnnnnnnnnnnnnnnnnnnnnnnnnnnnnnnnnnnnnnnnnnnnnnnnnnnnnnnnn

                  8074
DS571321_2985-26705    nnnnnnnnnnnnnnnnnnnnnnnnnnnnnnnnnnnnnnnnnnnnnnnnnnnnnnnnnnnnnnnnnnnnn
DS550441_21198-48907   nnnnnnnnnnnnnnnnnnnnnnnnnnnnnnnnnnnnnnnnnnnnnnnnnnnnnnnnnnnnnnnnnnnnn

                  8143
DS571321_2985-26705    nnnnnnnnnnnnnnnnnnnnnnnnnnnnnnnnnnnnnnnnnnnnnnnnnnnnnnnnnnnnnnnnnnnnn
DS550441_21198-48907   nnnnnnnnnnnnnnnnnnnnnnnnnnnnnnnnnnnnnnnnnnnnnnnnnnnnnnnnnnnnnnnnnnnnn
```

```
                      8212
DS571321_2985-26705    nnnnnnnnnnnnnnnnnnnnnnnnnnnnnnnnnnnnnnnnnnnnnnnnnnnnnnnnnnnnnnnnnnnnn
DS550441_21198-48907   nnnnnnnnnnnnnnnnnnnnnnnnnnnnnnnnnnnnnnnnnnnnnnnnnnnnnnnnnnnnnnnnnnnnn

                      8281
DS571321_2985-26705    nnnnnnnnnnnnnnnnnnnnnnnnnnnnnnnnnnnnnnnnnnnnnnnnnnnnnnnnnnnnnnnnnnnnn
DS550441_21198-48907   nnnnnnnnnnnnnnnnnnnnnnnnnnnnnnnnnnnnnnnnnnnnnnnnnnnnnnnnnnnnnnnnnnnnn

                      8350
DS571321_2985-26705    nnnnnnnnnnnnnnnnnnnnnnnnnnnnnnnnnnnnnnnnnnnnnnnnnnnnnnnnnnnnnnnnnnnnn
DS550441_21198-48907   nnnnnnnnnnnnnnnnnnnnnnnnnnnnnnnnnnnnnnnnnnnnnnnnnnnnnnnnnnnnnnnnnnnnn

                      8419
DS571321_2985-26705    nnnnnnnnnnnnnnnnnnnnnnnnnnnnnnnnnnnnnnnnnnnnnnnnnnnnnnnnnnnnnnnnnnnnn
DS550441_21198-48907   nnnnnnnnnnnnnnnnnnnnnnnnnnnnnnnnnnnnnnnnnnnnnnnnnnnnnnnnnnnnnnnnnnnnn

                      8488
DS571321_2985-26705    nnnnnnnnnnnnnnnnnnnnnnnnnnnnnnnnnnnnnnnnnnnnnnnnnnnnnnnnnnnnnnnnnnnnn
DS550441_21198-48907   nnnnnnnnnnnnnnnnnnnnnnnnnnnnnnnnnnnnnnnnnnnnnnnnnnnnnnnnnnnnnnnnnnnnn

                      8557
DS571321_2985-26705    nnnnnnnnnnnnnnnnnnnnnnnnnnnnnnnnnnnnnnnnnnnnnnnnnnnnnnnnnnnnnnnnnnnnn
DS550441_21198-48907   nnnnnnnnnnnnnnnnnnnnnnnnnnnnnnnnnnnnnnnnnnnnnnnnnnnnnnnnnnnnnnnnnnnnn

                      8626
DS571321_2985-26705    nnnnnnnnnnnnnnnnnnnnnnnnnnnnnnnnnnnnnnnnnnnnnnnnnnnnnnnnnnnnnnnnnnnnn
DS550441_21198-48907   nnnnnnnnnnnnnnnnnnnnnnnnnnnnnnnnnnnnnnnnnnnnnnnnnnnnnnnnnnnnnnnnnnnnn

                      8695
DS571321_2985-26705    nnnnnnnnnnnnnnnnnnnnnnnnnnnnnnnnnnnnnnnnnnnnnnnnnnnnnnnnnnnnnnnnnnnnn
DS550441_21198-48907   nnnnnnnnnnnnnnnnnnnnnnnnnnnnnnnnnnnnnnnnnnnnnnnnnnnnnnnnnnnnnnnnnnnnn

                      8764
DS571321_2985-26705    nnnnnnnnnnnnnnnnnnnnnnnnnnnnnnnnnnnnnnnnnnnnnnnnnnnnnnnnnnnnnnnnnnnnn
DS550441_21198-48907   nnnnnnnnnnnnnnnnnnnnnnnnnnnnnnnnnnnnnnnnnnnnnnnnnnnnnnnnnnnnnnnnnnnnn

                      8833
DS571321_2985-26705    nnnnnnnnnnnnnnnnnnnnnnnnnnnnnnnnnnnnnnnnnnnnnnnnnnnnnnnnnnnnnnnnnnnnn
DS550441_21198-48907   nnnnnnnnnnnnnnnnnnnnnnnnnnnnnnnnnnnnnnnnnnnnnnnnnnnnnnnnnnnnnnnnnnnnn

                      8902
DS571321_2985-26705    nnnnnnnnnnnnnnnnnnnnnnnnnnnnnnnnnnnnnnnnnnnnnnnnnnnnnnnnnnnnnnnnnnnnn
DS550441_21198-48907   nnnnnnnnnnnnnnnnnnnnnnnnnnnnnnnnnnnnnnnnnnnnnnnnnnnnnnnnnnnnnnnnnnnnn

                      8971
DS571321_2985-26705    nnnnnnnnnnnnnnnnnnnnnnnnnnnnnnnnnnnnnnnnnnnnnnnnnnnnnnnnnnnnnnnnnnnnn
DS550441_21198-48907   nnnnnnnnnnnnnnnnnnnnnnnnnnnnnnnnnnnnnnnnnnnnnnnnnnnnnnnnnnnnnnnnnnnnn

                      9040
DS571321_2985-26705    nnnnnnnnnnnnnnnnnnnnnnnnnnnnnnnnnnnnnnnnnnnnnnnnnnnnnnnnnnnnnnnnnnnnn
DS550441_21198-48907   nnnnnnnnnnnnnnnnnnnnnnnnnnnnnnnnnnnnnnnnnnnnnnnnnnnnnnnnnnnnnnnnnnnnn

                      9109
DS571321_2985-26705    nnnnnnnnnnnnnnnnnnnnnnnnnnnnnnnnnnnnnnnnnnnnnnnnnnnnnnnnnnnnnnnnnnnnn
DS550441_21198-48907   nnnnnnnnnnnnnnnnnnnnnnnnnnnnnnnnnnnnnnnnnnnnnnnnnnnnnnnnnnnnnnnnnnnnn

                      9178
DS571321_2985-26705    nnnnnnnnnnnnnnnnnnnnnnnnnnnnnnnnnnnnnnnnnnnnnnnnnnnnnnnnnnnnnnnnnnnnn
DS550441_21198-48907   nnnnnnnnnnnnnnnnnnnnnnnnnnnnnnnnnnnnnnnnnnnnnnnnnnnnnnnnnnnnnnnnnnnnn

                      9247
DS571321_2985-26705    nnnnnnnnnnnnnnnnnnnnnnnnnnnnnnnnnnnnnnnnnnnnnnnnnnnnnnnnnnnnnnnnnnnnn
DS550441_21198-48907   nnnnnnnnnnnnnnnnnnnnnnnnnnnnnnnnnnnnnnnnnnnnnnnnnnnnnnnnnnnnnnnnnnnnn

                      9316
DS571321_2985-26705    nnnnnnnnnnnnnnnnnnnnnnnnnnnnnnnnnnnnnnnnnnnnnnnnnnnnnnnnnnnnnnnnnnnnn
DS550441_21198-48907   nnnnnnnnnnnnnnnnnnnnnnnnnnnnnnnnnnnnnnnnnnnnnnnnnnnnnnnnnnnnnnnnnnnnn

                      9385
DS571321_2985-26705    nnnnnnnnnnnnnnnnnnnnnnnnnnnnnnnnnnnnnnnnnnnnnnnnnnnnnnnnnnnnnnnnnnnnn
DS550441_21198-48907   nnnnnnnnnnnnnnnnnnnnnnnnnnnnnnnnnnnnnnnnnnnnnnnnnnnnnnnnnnnnnnnnnnnnn

                      9454
DS571321_2985-26705    nnnnnnnnnnnnnnnnnnnnnnnnnnnnnnnnnnnnnnnnnnnnnnnnnnnnnnnnnnnnnnnnnnnnn
DS550441_21198-48907   nnnnnnnnnnnnnnnnnnnnnnnnnnnnnnnnnnnnnnnnnnnnnnnnnnnnnnnnnnnnnnnnnnnnn

                      9523
DS571321_2985-26705    nnnnnnnnnnnnnnnnnnnnnnnnnnnnnnnnnnnnnnnnnnnnnnnnnnnnnnnnnnnnnnnnnnnnn
DS550441_21198-48907   nnnnnnnnnnnnnnnnnnnnnnnnnnnnnnnnnnnnnnnnnnnnnnnnnnnnnnnnnnnnnnnnnnnnn
```

```
                    9592
DS571321_2985-26705    nnnnnnnnnnnnnnnnnnnnnnnnnnnnnnnnnnnnnnnnnnnnnnnnnnnnnnnnnnnnnnnnnnnnn
DS550441_21198-48907   nnnnnnnnnnnnnnnnnnnnnnnnnnnnnnnnnnnnnnnnnnnnnnnnnnnnnnnnnnnnnnnnnnnnn

                    9661
DS571321_2985-26705    nnnnnnnnnnnnnnnnnnnnnnnnnnnnnnnnnnnnnnnnnnnnnnnnnnnnnnnnnnnnnnnnnnnnn
DS550441_21198-48907   nnnnnnnnnnnnnnnnnnnnnnnnnnnnnnnnnnnnnnnnnnnnnnnnnnnnnnnnnnnnnnnnnnnnn

                    9730
DS571321_2985-26705    nnnnnnnnnnnnnnnnnnnnnnnnnnnnnnnnnnnnnnnnnnnnnnnnnnnnnnnnnnnnnnnnnnnnn
DS550441_21198-48907   nnnnnnnnnnnnnnnnnnnnnnnnnnnnnnnnnnnnnnnnnnnnnnnnnnnnnnnnnnnnnnnnnnnnn

                    9799
DS571321_2985-26705    nnnnnnnnnnnnnnnnnnnnnnnnnnnnnnnnnnnnnnnnnnnnnnnnnnnnnnnnnnnnnnnnnnnnn
DS550441_21198-48907   nnnnnnnnnnnnnnnnnnnnnnnnnnnnnnnnnnnnnnnnnnnnnnnnnnnnnnnnnnnnnnnnnnnnn

                    9868
DS571321_2985-26705    nnnnnnnnnnnnnnnnnnnnnnnnnnnnnnnnnnnnnnnnnnnnnnnnnnnnnnnnnnnnnnnnnnnnn
DS550441_21198-48907   nnnnnnnnnnnnnnnnnnnnnnnnnnnnnnnnnnnnnnnnnnnnnnnnnnnnnnnnnnnnnnnnnnnnn

                    9937
DS571321_2985-26705    nnnnnnnnnnnnnnnnnnnnnnnnnnnnnnnnnnnnnnnnnnnnnnnnnnnnnnnnnnnnnnnnnnnnn
DS550441_21198-48907   nnnnnnnnnnnnnnnnnnnnnnnnnnnnnnnnnnnnnnnnnnnnnnnnnnnnnnnnnnnnnnnnnnnnn

                   10006
DS571321_2985-26705    nnnnnnnnnnnnnnnnnnnnnnnnnnnnnnnnnnnnnnnnnnnnnnnnnnnnnnnnnnnnnnnnnnnn
DS550441_21198-48907   nnnnnnnnnnnnnnnnnnnnnnnnnnnnnnnnnnnnnnnnnnnnnnnnnnnnnnnnnnnnnnnnnnnnn

                   10075
DS571321_2985-26705    nnnnnnnnnnnnnnnnnnnnnnnnnnnnnnnnnnnnnnnnnnnnnnnnnnnnnnnnnnnnnnnnnnnnn
DS550441_21198-48907   nnnnnnnnnnnnnnnnnnnnnnnnnnnnnnnnnnnnnnnnnnnnnnnnnnnnnnnnnnnnnnnnnnnnn

                   10144
DS571321_2985-26705    nnnnnnnnnnnnnnnnnnnnnnnnnnnnnnnnnnnnnnnnnnnnnnnnnnnnnnnnnnnnnnnnnnnnn
DS550441_21198-48907   nnnnnnnnnnnnnnnnnnnnnnnnnnnnnnnnnnnnnnnnnnnnnnnnnnnnnnnnnnnnnnnnnnnnn

                   10213
DS571321_2985-26705    nnnnnnnnnnnnnnnnnnnnnnnnnnnnnnnnnnnnnnnnnnnnnnnnnnnnnnnnnnnnnnnnnnnnn
DS550441_21198-48907   nnnnnnnnnnnnnnnnnnnnnnnnnnnnnnnnnnnnnnnnnnnnnnnnnnnnnnnnnnnnnnnnnnnnn

                   10282
DS571321_2985-26705    nnnnnnnnnnnnnnnnnnnnnnnnnnnnnnnnnnnnnnnnnnnnnnnnnnnnnnnnnnnnnnnnnnnnn
DS550441_21198-48907   nnnnnnnnnnnnnnnnnnnnnnnnnnnnnnnnnnnnnnnnnnnnnnnnnnnnnnnnnnnnnnnnnnnnn

                   10351
DS571321_2985-26705    nnnnnnnnnnnnnnnnnnnnnnnnnnnnnnnnnnnnnnnnnnnnnnnnnnnnnnnnnnnnnnnnnnnnn
DS550441_21198-48907   nnnnnnnnnnnnnnnnnnnnnnnnnnnnnnnnnnnnnnnnnnnnnnnnnnnnnnnnnnnnnnnnnnnnn

                   10420
DS571321_2985-26705    nnnnnnnnnnnnnnnnnnnnnnnnnnnnnnnnnnnnnnnnnnnnnnnnnnnnnnnnnnnnnnnnnnnnn
DS550441_21198-48907   nnnnnnnnnnnnnnnnnnnnnnnnnnnnnnnnnnnnnnnnnnnnnnnnnnnnnnnnnnnnnnnnnnnnn

                   10489
DS571321_2985-26705    nnnnnnnnnnnnnnnnnnnnnnnnnnnnnnnnnnnnnnnnnnnnnnnnnnnnnnnnnnnnnnnnnnnnn
DS550441_21198-48907   nnnnnnnnnnnnnnnnnnnnnnnnnnnnnnnnnnnnnnnnnnnnnnnnnnnnnnnnnnnnnnnnnnnnn

                   10558
DS571321_2985-26705    nnnnnnnnnnnnnnnnnnnnnnnnnnnnnnnnnnnnnnnnnnnnnnnnnnnnnnnnnnnnnnnnnnnnn
DS550441_21198-48907   nnnnnnnnnnnnnnnnnnnnnnnnnnnnnnnnnnnnnnnnnnnnnnnnnnnnnnnnnnnnnnnnnnnnn

                   10627
DS571321_2985-26705    nnnnnnnnnnnnnnnnnnnnnnnnnnnnnnnnnnnnnnnnnnnnnnnnnnnnnnnnnnnnnnnnnnnnn
DS550441_21198-48907   nnnnnnnnnnnnnnnnnnnnnnnnnnnnnnnnnnnnnnnnnnnnnnnnnnnnnnnnnnnnnnnnnnnnn

                   10696
DS571321_2985-26705    nnnnnnnnnnnnnnnnnnnnnnnnnnnnnnnnnnnnnnnnnnnnnnnnnnnnnnnnnnnnnnnnnnnnn
DS550441_21198-48907   nnnnnnnnnnnnnnnnnnnnnnnnnnnnnnnnnnnnnnnnnnnnnnnnnnnnnnnnnnnnnnnnnnnnn

                   10765
DS571321_2985-26705    nnnnnnnnnnnnnnnnnnnnnnnnnnnnnnnnnnnnnnnnnnnnnnnnnnnnnnnnnnnnnnnnnnnnn
DS550441_21198-48907   nnnnnnnnnnnnnnnnnnnnnnnnnnnnnnnnnnnnnnnnnnnnnnnnnnnnnnnnnnnnnnnnnnnnn

                   10834
DS571321_2985-26705    nnnnnnnnnnnnnnnnnnnnnnnnnnnnnnnnnnnnnnnnnnnnnnnnnnnnnnnnnnnnnnnnnnnnn
DS550441_21198-48907   nnnnnnnnnnnnnnnnnnnnnnnnnnnnnnnnnnnnnnnnnnnnnnnnnnnnnnnnnnnnnnnnnnnnn

                   10903
DS571321_2985-26705    nnnnnnnnnnnnnnnnnnnnnnnnnnnnnnnnnnnnnnnnnnnnnnnnnnnnnnnnnnnnnnnnnnnnn
DS550441_21198-48907   nnnnnnnnnnnnnnnnnnnnnnnnnnnnnnnnnnnnnnnnnnnnnnnnnnnnnnnnnnnnnnnnnnnnn
```

```
                   10972
DS571321_2985-26705    nnnnnnnnnnnnnnnnnnnnnnnnnnnnnnnnnnnnnnnnnnnnnnnnnnnnnnnnnnnnnnnnnnnnn
DS550441_21198-48907   nnnnnnnnnnnnnnnnnnnnnnnnnnnnnnnnnnnnnnnnnnnnnnnnnnnnnnnnnnnnnnnnnnnnn

                   11041
DS571321_2985-26705    nnnnnnnnnnnnnnnnnnnnnnnnnnnnnnnnnnnnnnnnnnnnnnnnnnnnnnnnnnnnnnnnnnnnn
DS550441_21198-48907   nnnnnnnnnnnnnnnnnnnnnnnnnnnnnnnnnnnnnnnnnnnnnnnnnnnnnnnnnnnnnnnnnnnnn

                   11110
DS571321_2985-26705    nnnnnnnnnnnnnnnnnnnnnnnnnnnnnnnnnnnnnnnnnnnnnnnnnnnnnnnnnnnnnnnnnnnnn
DS550441_21198-48907   nnnnnnnnnnnnnnnnnnnnnnnnnnnnnnnnnnnnnnnnnnnnnnnnnnnnnnnnnnnnnnnnnnnnn

                   11179
DS571321_2985-26705    nnnnnnnnnnnnnnnnnnnnnnnnnnnnnnnnnnnnnnnnnnnnnnnnnnnnnnnnnnnnnnnnnnnnn
DS550441_21198-48907   nnnnnnnnnnnnnnnnnnnnnnnnnnnnnnnnnnnnnnnnnnnnnnnnnnnnnnnnnnnnnnnnnnnnn

                   11248
DS571321_2985-26705    nnnnnnnnnnnnnnnnnnnnnnnnnnnnnnnnnnnnnnnnnnnnnnnnnnnnnnnnnnnnnnnnnnnnn
DS550441_21198-48907   nnnnnnnnnnnnnnnnnnnnnnnnnnnnnnnnnnnnnnnnnnnnnnnnnnnnnnnnnnnnnnnnnnnnn

                   11317
DS571321_2985-26705    nnnnnnnnnnnnnnnnnnnnnnnnnnnnnnnnnnnnnnnnnnnnnnnnnnnnnnnnnnnnnnnnnnnnn
DS550441_21198-48907   nnnnnnnnnnnnnnnnnnnnnnnnnnnnnnnnnnnnnnnnnnnnnnnnnnnnnnnnnnnnnnnnnnnnn

                   11386
DS571321_2985-26705    nnnnnnnnnnnnnnnnnnnnnnnnnnnnnnnnnnnnnnnnnnnnnnnnnnnnnnnnnnnnnnnnnnnnn
DS550441_21198-48907   nnnnnnnnnnnnnnnnnnnnnnnnnnnnnnnnnnnnnnnnnnnnnnnnnnnnnnnnnnnnnnnnnnnnn

                   11455
DS571321_2985-26705    nnnnnnnnnnnnnnnnnnnnnnnnnnnnnnnnnnnnnnnnnnnnnnnnnnnnnnnnnnnnnnnnnnnnn
DS550441_21198-48907   nnnnnnnnnnnnnnnnnnnnnnnnnnnnnnnnnnnnnnnnnnnnnnnnnnnnnnnnnnnnnnnnnnnnn

                   11524
DS571321_2985-26705    nnnnnnnnnnnnnnnnnnnnnnnnnnnnnnnnnnnnnnnnnnnnnnnnnnnnnnnnnnnnnnnnnnnnn
DS550441_21198-48907   nnnnnnnnnnnnnnnnnnnnnnnnnnnnnnnnnnnnnnnnnnnnnnnnnnnnnnnnnnnnnnnnnnnnn

                   11593
DS571321_2985-26705    nnnnnnnnnnnnnnnnnnnnnnnnnnnnnnnnnnnnnnnnnnnnnnnnnnnnnnnnnnnnnnnnnnnnn
DS550441_21198-48907   nnnnnnnnnnnnnnnnnnnnnnnnnnnnnnnnnnnnnnnnnnnnnnnnnnnnnnnnnnnnnnnnnnnnn

                   11662
DS571321_2985-26705    nnnnnnnnnnnnnnnnnnnnnnnnnnnnnnnnnnnnnnnnnnnnnnnnnnnnnnnnnnnnnnnnnnnnn
DS550441_21198-48907   nnnnnnnnnnnnnnnnnnnnnnnnnnnnnnnnnnnnnnnnnnnnnnnnnnnnnnnnnnnnnnnnnnnnn

                   11731
DS571321_2985-26705    nnnnnnnnnnnnnnnnnnnnnnnnnnnnnnnnnnnnnnnnnnnnnnnnnnnnnnnnnnnnnnnnnnnnn
DS550441_21198-48907   nnnnnnnnnnnnnnnnnnnnnnnnnnnnnnnnnnnnnnnnnnnnnnnnnnnnnnnnnnnnnnnnnnnnn

                   11800
DS571321_2985-26705    nnnnnnnnnnnnnnnnnnnnnnnnnnnnnnnnnnnnnnnnnnnnnnnnnnnnnnnnnnnnnnnnnnnnn
DS550441_21198-48907   nnnnnnnnnnnnnnnnnnnnnnnnnnnnnnnnnnnnnnnnnnnnnnnnnnnnnnnnnnnnnnnnnnnnn

                   11869
DS571321_2985-26705    nnnnnnnnnnnnnnnnnnnnnnnnnnnnnnnnnnnnnnnnnnnnnnnnnnnnnnnnnnnnnnnnnnnnn
DS550441_21198-48907   nnnnnnnnnnnnnnnnnnnnnnnnnnnnnnnnnnnnnnnnnnnnnnnnnnnnnnnnnnnnnnnnnnnnn

                   11938
DS571321_2985-26705    nnnnnnnnnnnnnnnnnnnnnnnnnnnnnnnnnnnnnnnnnnnnnnnnnnnnnnnnnnnnnnnnnnnnn
DS550441_21198-48907   nnnnnnnnnnnnnnnnnnnnnnnnnnnnnnnnnnnnnnnnnnnnnnnnnnnnnnnnnnnnnnnnnnnnn

                   12007
DS571321_2985-26705    nnnnnnnnnnnnnnnnnnnnnnnnnnnnnnnnnnnnnnnnnnnnnnnnnnnnnnnnnnnnnnnnnnnnn
DS550441_21198-48907   nnnnnnnnnnnnnnnnnnnnnnnnnnnnnnnnnnnnnnnnnnnnnnnnnnnnnnnnnnnnnnnnnnnnn

                   12076
DS571321_2985-26705    nnnnnnnnnnnnnnnnnnnnnnnnnnnnnnnnnnnnnnnnnnnnnnnnnnnnnnnnnnnnnnnnnnnnn
DS550441_21198-48907   nnnnnnnnnnnnnnnnnnnnnnnnnnnnnnnnnnnnnnnnnnnnnnnnnnnnnnnnnnnnnnnnnnnnn

                   12145
DS571321_2985-26705    nnnnnnnnnnnnnnnnnnnnnnnnnnnnnnnnnnnnnnnnnnnnnnnnnnnnnnnnnnnnnnnnnnnnn
DS550441_21198-48907   nnnnnnnnnnnnnnnnnnnnnnnnnnnnnnnnnnnnnnnnnnnnnnnnnnnnnnnnnnnnnnnnnnnnn

                   12214
DS571321_2985-26705    nnnnnnnnnnnnnnnnnnnnnnnnnnnnnnnnnnnnnnnnnnnnnnnnnnnnnnnnnnnnnnnnnnnnn
DS550441_21198-48907   nnnnnnnnnnnnnnnnnnnnnnnnnnnnnnnnnnnnnnnnnnnnnnnnnnnnnnnnnnnnnnnnnnnnn

                   12283
DS571321_2985-26705    nnnnnnnnnnnnnnnnnnnnnnnnnnnnnnnnnnnnnnnnnnnnnnnnnnnnnnnnnnnnnnnnnnnnn
DS550441_21198-48907   nnnnnnnnnnnnnnnnnnnnnnnnnnnnnnnnnnnnnnnnnnnnnnnnnnnnnnnnnnnnnnnnnnnnn
```

```
                      12352
DS571321_2985-26705    nnnnnnnnnnnnnnnnnnnnnnnnnnnnnnnnnnnnnnnnnnnnnnnnnnnnnnnnnnnnnnnnnnnnn
DS550441_21198-48907   nnnnnnnnnnnnnnnnnnnnnnnnnnnnnnnnnnnnnnnnnnnnnnnnnnnnnnnnnnnnnnnnnnnnn

                      12421
DS571321_2985-26705    nnnnnnnnnnnnnnnnnnnnnnnnnnnnnnnnnnnnnnnnnnnnnnnnnnnnnnnnnnnnnnnnnnnnn
DS550441_21198-48907   nnnnnnnnnnnnnnnnnnnnnnnnnnnnnnnnnnnnnnnnnnnnnnnnnnnnnnnnnnnnnnnnnnnnn

                      12490
DS571321_2985-26705    nnnnnnnnnnnnnnnnnnnnnnnnnnnnnnnnnnnnnnnnnnnnnnnnnnnnnnnnnnnnnnnnnnnnn
DS550441_21198-48907   nnnnnnnnnnnnnnnnnnnnnnnnnnnnnnnnnnnnnnnnnnnnnnnnnnnnnnnnnnnnnnnnnnnnn

                      12559
DS571321_2985-26705    nnnnnnnnnnnnnnnnnnnnnnnnnnnnnnnnnnnnnnnnnnnnnnnnnnnnnnnnnnnnnnnnnnnnn
DS550441_21198-48907   nnnnnnnnnnnnnnnnnnnnnnnnnnnnnnnnnnnnnnnnnnnnnnnnnnnnnnnnnnnnnnnnnnnnn

                      12628
DS571321_2985-26705    nnnnnnnnnnnnnnnnnnnnnnnnnnnnnnnnnnnnnnnnnnnnnnnnnnnnnnnnnnnnnnnnnnnnn
DS550441_21198-48907   nnnnnnnnnnnnnnnnnnnnnnnnnnnnnnnnnnnnnnnnnnnnnnnnnnnnnnnnnnnnnnnnnnnnn

                      12697
DS571321_2985-26705    nnnnnnnnnnnnnnnnnnnnnnnnnnnnnnnnnnnnnnnnnnnnnnnnnnnnnnnnnnnnnnnnnnnnn
DS550441_21198-48907   nnnnnnnnnnnnnnnnnnnnnnnnnnnnnnnnnnnnnnnnnnnnnnnnnnnnnnnnnnnnnnnnnnnnn

                      12766
DS571321_2985-26705    nnnnnnnnnnnnnnnnnnnnnnnnnnnnnnnnnnnnnnnnnnnnnnnnnnnnnnnnnnnnnnnnnnnnn
DS550441_21198-48907   nnnnnnnnnnnnnnnnnnnnnnnnnnnnnnnnnnnnnnnnnnnnnnnnnnnnnnnnnnnnnnnnnnnnn

                      12835
DS571321_2985-26705    nnnnnnnnnnnnnnnnnnnnnnnnnnnnnnnnnnnnnnnnnnnnnnnnnnnnnnnnnnnnnnnnnnnnn
DS550441_21198-48907   nnnnnnnnnnnnnnnnnnnnnnnnnnnnnnnnnnnnnnnnnnnnnnnnnnnnnnnnnnnnnnnnnnnnn

                      12904
DS571321_2985-26705    nnnnnnnnnnnnnnnnnnnnnnnnnnnnnnnnnnnnnnnnnnnnnnnnnnnnnnnnnnnnnnnnnnnnn
DS550441_21198-48907   nnnnnnnnnnnnnnnnnnnnnnnnnnnnnnnnnnnnnnnnnnnnnnnnnnnnnnnnnnnnnnnnnnnnn

                      12973
DS571321_2985-26705    nnnnnnnnnnnnnnnnnnnnnnnnnnnnnnnnnnnnnnnnnnnnnnnnnnnnnnnnnnnnnnnnnnnnn
DS550441_21198-48907   nnnnnnnnnnnnnnnnnnnnnnnnnnnnnnnnnnnnnnnnnnnnnnnnnnnnnnnnnnnnnnnnnnnnn

                      13042
DS571321_2985-26705    nnnnnnnnnnnnnnnnnnnnnnnnnnnnnnnnnnnnnnnnnnnnnnnnnnnnnnnnnnnnnnnnnnnnn
DS550441_21198-48907   nnnnnnnnnnnnnnnnnnnnnnnnnnnnnnnnnnnnnnnnnnnnnnnnnnnnnnnnnnnnnnnnnnnnn

                      13111
DS571321_2985-26705    nnnnnnnnnnnnnnnnnnnnnnnnnnnnnnnnnnnnnnnnnnnnnnnnnnnnnnnnnnnnnnnnnnnnn
DS550441_21198-48907   nnnnnnnnnnnnnnnnnnnnnnnnnnnnnnnnnnnnnnnnnnnnnnnnnnnnnnnnnnnnnnnnnnnnn

                      13180
DS571321_2985-26705    nnnnnnnnnnnnnnnnnnnnnnnnnnnnnnnnnnnnnnnnnnnnnnnnnnnnnnnnnnnnnnnnnnnnn
DS550441_21198-48907   nnnnnnnnnnnnnnnnnnnnnnnnnnnnnnnnnnnnnnnnnnnnnnnnnnnnnnnnnnnnnnnnnnnnn

                      13249
DS571321_2985-26705    nnnnnnnnnnnnnnnnnnnnnnnnnnnnnnnnnnnnnnnnnnnnnnnnnnnnnnnnnnnnnnnnnnnnn
DS550441_21198-48907   nnnnnnnnnnnnnnnnnnnnnnnnnnnnnnnnnnnnnnnnnnnnnnnnnnnnnnnnnnnnnnnnnnnnn

                      13318
DS571321_2985-26705    nnnnnnnnnnnnnnnnnnnnnnnnnnnnnnnnnnnnnnnnnnnnnnnnnnnnnnnnnnnnnnnnnnnnn
DS550441_21198-48907   nnnnnnnnnnnnnnnnnnnnnnnnnnnnnnnnnnnnnnnnnnnnnnnnnnnnnnnnnnnnnnnnnnnnn

                      13387
DS571321_2985-26705    nnnnnnnnnnnnnnnnnnnnnnnnnnnnnnnnnnnnnnnnnnnnnnnnnnnnnnnnnnnnnnnnnnnnn
DS550441_21198-48907   nnnnnnnnnnnnnnnnnnnnnnnnnnnnnnnnnnnnnnnnnnnnnnnnnnnnnnnnnnnnnnnnnnnnn

                      13456
DS571321_2985-26705    nnnnnnnnnnnnnnnnnnnnnnnnnnnnnnnnnnnnnnnnnnnnnnnnnnnnnnnnnnnnnnnnnnnnn
DS550441_21198-48907   nnnnnnnnnnnnnnnnnnnnnnnnnnnnnnnnnnnnnnnnnnnnnnnnnnnnnnnnnnnnnnnnnnnnn

                      13525
DS571321_2985-26705    nnnnnnnnnnnnnnnnnnnnnnnnnnnnnnnnnnnnnnnnnnnnnnnnnnnnnnnnnnnnnnnnnnnnn
DS550441_21198-48907   nnnnnnnnnnnnnnnnnnnnnnnnnnnnnnnnnnnnnnnnnnnnnnnnnnnnnnnnnnnnnnnnnnnnn

                      13594
DS571321_2985-26705    nnnnnnnnnnnnnnnnnnnnnnnnnnnnnnnnnnnnnnnnnnnnnnnnnnnnnnnnnnnnnnnnnnnnn
DS550441_21198-48907   nnnnnnnnnnnnnnnnnnnnnnnnnnnnnnnnnnnnnnnnnnnnnnnnnnnnnnnnnnnnnnnnnnnnn

                      13663
DS571321_2985-26705    nnnnnnnnnnnnnnnnnnnnnnnnnnnnnnnnnnnnnnnnnnnnnnnnnnnnnnnnnnnnnnnnnnnnn
DS550441_21198-48907   nnnnnnnnnnnnnnnnnnnnnnnnnnnnnnnnnnnnnnnnnnnnnnnnnnnnnnnnnnnnnnnnnnnnn
```

```
                    13732
DS571321_2985-26705   nnnnnnnnnnnnnnnnnnnnnnnnnnnnnnnnnnnnnnnnnnnnnnnnnnnnnnnnnnnnnnnnnnnnn
DS550441_21198-48907  nnnnnnnnnnnnnnnnnnnnnnnnnnnnnnnnnnnnnnnnnnnnnnnnnnnnnnnnnnnnnnnnnnnnn

                    13801
DS571321_2985-26705   nnnnnnnnnnnnnnnnnnnnnnnnnnnnnnnnnnnnnnnnnnnnnnnnnnnnnnnnnnnnnnnnnnnnn
DS550441_21198-48907  nnnnnnnnnnnnnnnnnnnnnnnnnnnnnnnnnnnnnnnnnnnnnnnnnnnnnnnnnnnnnnnnnnnnn

                    13870
DS571321_2985-26705   nnnnnnnnnnnnnnnnnnnnnnnnnnnnnnnnnnnnnnnnnnnnnnnnnnnnnnnnnnnnnnnnnnnnn
DS550441_21198-48907  nnnnnnnnnnnnnnnnnnnnnnnnnnnnnnnnnnnnnnnnnnnnnnnnnnnnnnnnnnnnnnnnnnnnn

                    13939
DS571321_2985-26705   nnnnnnnnnnnnnnnnnnnnnnnnnnnnnnnnnnnnnnnnnnnnnnnnnnnnagaaataatgcaaagtt
DS550441_21198-48907  nnnnnnnnnnnnnnnnnnnnnnnnnnnnnnnnnnnnnnnnnnnnnnnnnnnnagaaattaagcaaaatg

                    14008
DS571321_2985-26705   ataatttatattactcgttactttgtttattaatttattccttcaaaaataacaa-acactgaaattga
DS550441_21198-48907  attatttatattaagagttaattt-tatattaagttgttactttaacaacaataacacaataaaaagat

                    14077
DS571321_2985-26705   tgaaattaaaaagggaataaacaatagaattaaagtaaattatctgaaattgttataacaaagtatta-
DS550441_21198-48907  taaattcagaaatagaaaaaataatagagttaaaataatttatttaaaaataatgtattaaagtaatac

                    14146
DS571321_2985-26705   --ttaagtgtaaatgttcatttgttgaatagaaata-atatatatttttaattaatcaatctttttgta
DS550441_21198-48907  tgttaattgtagatattcatttgtttaatagaaatatatat-tttttttaattaacta--cttttt-ta

                    14215
DS571321_2985-26705   taaaagaaagaggtttttc--atgtcaattataaattaattgctttttattta-----taa-tacataa
DS550441_21198-48907  taaaaggaagtagtttttcatatattacttaaaaattaatt--ttcttatataataaataaataaacaa

                    14284
DS571321_2985-26705   tgaagaataaagTTAATAAAATAAAGCAACATCGTCTAAAAGAATTTCTCCTGTAAAATATAAAGTAAA
DS550441_21198-48907  tgaagaat-atgTTAATAAAATAATGCAACATCATCCAAAAGAATTTCTCCTGTAAAATATAAAGTGAA

                    14353
DS571321_2985-26705   ACTGGTAATTACTTGATTTGGTAATGGAAAGGAGAAAGAATATGTTTGAACAAAAAACATTTCACAAAT
DS550441_21198-48907  ACTTGTAATAATTTGATTTGGTAATGGAAAGGAGAAATAATATGTTTGAACAAAAAACATTTCACAAAT

                    14422
DS571321_2985-26705   TTCTCCATTATTTTCTATTTCAGATATTTTAGTATATGTGAAAGATGGTAAAGTAAATGTTGAATTTGA
DS550441_21198-48907  CTCTCCATTATTTTCGATTTCAGATATTTTAGTATATGTGAAAGATGGTAAAGTAAATGTTGAATTAGA

                    14491
DS571321_2985-26705   TAGACATGTTACTTTAATTGATTGTTCATATATTTTAGAAGTGTCTTGTTCTTCTTTATTAATTATTCC
DS550441_21198-48907  TAGAGATGTTACTTTAATTGATTGGTTGTATATTTTAGAAGGGTCTTGTTCTTCTTTATTAATTATTCC

                    14560
DS571321_2985-26705   ATTATTTTTAAAAAATGAGAATTTAATTCCACTGCAATTTACTGGTTTATTCATTACAAATTTTAAAGC
DS550441_21198-48907  ATTATTTTTAAAAAATGAAAATTTAATTCCACTGCAATTTGTTGGTTTATTCATAACAAATTTTAATGC

                    14629
DS571321_2985-26705   ACATTTCATTCCTGAATTACAAACATATAATTGATGTAATGACTGATACATATTTTGATATAATTTACC
DS550441_21198-48907  ACATTTCATTCCTGAATCACAAACATGTAATTGATGTAATGATTGATACATATTTTGATATAACTTACC

                    14698
DS571321_2985-26705   ACTATATGTAGAATTACTTTCTATTGAAATATCAAACGTTGTCCCATTTACATAAAGAAGTATTTCTTT
DS550441_21198-48907  ATTATATGTTGCATTACTTTCTATTGAAATATCAAACATAGTTCCATTTACATAAAGAAGTATTTCTTT

                    14767
DS571321_2985-26705   ATTTGATTCAAAAGTACTATAATATCCACTTTCTATTGGAATAATTGATTTTCCATTTTTAAAATCCAT
DS550441_21198-48907  ATTTGATTCAAAAGTACTATAATATCCACTTTCAATTGGAATAATTGATTTTCCATTCTTAAAATCCAT

                    14836
DS571321_2985-26705   AAGTAAAGGTAAATTATCACATTTATTATCCATACTACATAAAAAATGACTTATAACATAATTATTAAC
DS550441_21198-48907  AAGTAAAGGCAAATTATCACATTTATTATCCATACTACATAAAAAATGACTTGTAACATAATTATTAAC

                    14905
DS571321_2985-26705   TAAACATCGAAGTTCATTTTCTTTTAATAAATAAGAGTTGTTTTTAAGAATATAATCAACTAATATATC
DS550441_21198-48907  TAAACAACGAAGTTCAGTTTCTTTTAATAAATAAGAATTATTTTTAATAATATAATCAACCAATATATC

                    14974
DS571321_2985-26705   ACTTTGTGCTTGTTGATATTTCATATTAAAATAATCCCCAATACCTTTTTCAATGATTAAACTACCACT
DS550441_21198-48907  ACTTTGTGCTTGTTGATATTTCATATTAAAATAATCTCCAATACCTTTTTCAATGATTAAACTACCACT

                    15043
DS571321_2985-26705   ATACTTAAATAAAATATTATCATCTCTATCTGTTTTATTAAAATAATTATATGAAGATAATAAAGGAGG
DS550441_21198-48907  ATATTTAAATAAAATATTATCATCTCTATCTGTTTTATTAAAATAATTATATGAAGACAATAAAGGAGG
```

```
                          15112
DS571321_2985-26705    GTCATATGGTGATAATTCAGGGAATGTTTCTATAAAATATGAATTCATTCCAACCATAGGATTTTCGAC
DS550441_21198-48907   GTCATATGGTGATAATTCAGGTAATGTTTCTATAAAATATGAATTCATTCCAACCATAGGATTTTGAAC

                          15181
DS571321_2985-26705    TGGAATATCTTTACAATTTAATACAAAAATACTTTCAATACTAAATTGGTTTAAATTCTCTTTTTCAAT
DS550441_21198-48907   TGGAATATCTTTACAATTTAATACAAAAATACTTTCAATATTAAATTGATTTAAATTCTCTTTTTCAAT

                          15250
DS571321_2985-26705    AAAATCAATTTCACCAATTCTTAATTTATTAATAATTCTCATAGCAACTCCTCCACCTTCTCTATCACC
DS550441_21198-48907   AAAATCAATATTACCAATTCTTAATGTATTAAGAATTCTCATAGCAACTCCTCCACCTTCTCTATCACC

                          15319
DS571321_2985-26705    AATTATTCCAATATTTTCAAAATTTAATTTTTGTCCAAAAAATTGGTTTAGTCGTTTAAATAAATAGTT
DS550441_21198-48907   AATAATTCCAATACTTTCAAAATTTAATTTTTGTCCAAAGAATTGATTTAATCGTTTAAATAAATAATT

                          15388
DS571321_2985-26705    GATTGTTTGAATAGTTAAAACAACACGTGCATTTAAATATCCTTCTTTAACACTCATTCCATTCTTTGG
DS550441_21198-48907   AATTGTTTGAATAGTTAAAACAACACGTGCATTTAAATATCCTTCTTTAACACTCATTCCATTCTTTGG

                          15457
DS571321_2985-26705    TAAAATTGAATTTCCATTTTCATCTTTATCAAGAAATCCTTGATCAAGACAAATACTAACTATTCCATT
DS550441_21198-48907   TATAATTGAATTTCCATTTACATCTTTATCAAGAAATCCTTGATCAAGACAAATACTAACAATTCCATT

                          15526
DS571321_2985-26705    CTCAGCTAAACTTTCTTGAAGATAAATATATCCCATATCTGAATCAATTAAATTCCCCATTTCACTTCC
DS550441_21198-48907   CTCAGCTAAACTTTCTTGAAGATAAACATATCCCATATCTGAATCAATTAAATTCCCCATTTCACTTCC

                          15595
DS571321_2985-26705    AAGAATTATAACAACACTTTTTGTTTCATTAGAGGTTTTAATATTTGTTGGATAATAAACAATACCATT
DS550441_21198-48907   AAGAATTATAACAACACTTTTTGTTTCATTAGAAGTTTTAATATTTGTTGGATAATAAACAATACCATT

                          15664
DS571321_2985-26705    CATTGGAATAGCAGAGCTAGTCATTCTATAATAATCTTGTGTTGTTGAACTTACATCAAACAATGAAGA
DS550441_21198-48907   TATTGGAATAGCAGAACTAGTCATTCTATAATAATCTTGTGTTGTTGAACTTACATCAAATAATGATGA

                          15733
DS571321_2985-26705    CATATTAATTGTTGGTGTTTTAATTGATGGTTGAGCATATTCTTGTCTTAAATAACTTGAATCACCAAT
DS550441_21198-48907   CATATTAATTGTTGGTGTTTTAATTGAAGGTTGGGCATATTCTTGTCTTAAATAACTTGAATCTCCAAT

                          15802
DS571321_2985-26705    ACCAAAAGTTAGTTGAGAAATATGTTTTTCAAATTCACAATAACTAATAACTTCATTAATATAACTAGT
DS550441_21198-48907   ACCAAAAGTCAATTGGGAAATATGTTTTTCAAATTCACAGTAACTAATAACTTCATTAACATAACTAGT

                          15871
DS571321_2985-26705    ATTTACAGTATCAAAACTACTTAGAAAAGAAAGGTTGTATGTATATTTTAATTTATAAAACATATTACT
DS550441_21198-48907   ATTTACAGTATCAAAAGTACTTAAAAAAGAAGGGTTGTGTTGATATTTTAATTTATAAAACATATTATG

                          15940
DS571321_2985-26705    TAATGATAAGTTAAAAGAAACTAAAATAATTATAATACAAAAGATTAGTACTAATAATGATATTATATG
DS550441_21198-48907   TAATGATAAATTAAAAGAAATTATAATAGTTATAATACAAAAGATTAATACTGATAAGGATACAATATG

                          16009
DS571321_2985-26705    ATTCTTTACTTTTTTTAATTCTGAAAAAATAAAGGAACTAAAAGATACTTTTTGATGTATGAAAATATA
DS550441_21198-48907   ATTCTTTAATTTTTTTAATTCTGAAAAAATAAATGAAGTAAGAGATGCTTTTTGATGTATAAAAATATA

                          16078
DS571321_2985-26705    AATATACCACCCTATTTCAATAGTACAAAATAATAGTATTGTTATTATCAAACTTCCCATTATAAGAAA
DS550441_21198-48907   AATATACCATCCTATTTCAATAGTACAAAATATTATTATTGTAATTATTAAACTTCCCATTGTAAGAAA

                          16147
DS571321_2985-26705    CCAAATACATGCCATAGATTTTTGTTGAAGAACTACAACCGAATTACAAAATGGAATTGTTATAATTGA
DS550441_21198-48907   CCAAATACATGCAATAGATTTATGTTGAAGAGCTACAACTGAATTACAAAAAGGAATTGTTATAATTGA

                          16216
DS571321_2985-26705    TAATAATAATATTGAAATTATTATTTCTTCTTTAAATCCACCTTTTATATAACCATAACTAACTTGAAG
DS550441_21198-48907   TAATAATAATATTGAAATTATTACTTCTTCTTTAAAACTACCTTTTACACAACCATAACTAACTTGAAG

                          16285
DS571321_2985-26705    AATAAAGAATATAGCCATTAAAATAACTTTCCAAAGAAAGAATAATGTCAAAGAAATATTAATAATGCA
DS550441_21198-48907   AATAAAGAAGATAACTACTAAAATAACTTTCCAAAGAAAGAATAACGCTAATGAAATATTAATAATACA

                          16354
DS571321_2985-26705    AACAACTGAATAAACTAAAACTTGTGTATATATTATATTGAATGATACAGTAGCTTGTTGATAAAGAAA
DS550441_21198-48907   AACAATTGAATAAACTAAAACTTGAGTATAAACTATATTGAATGATACAATAGCCTGTTGATAAAGAAA

                          16423
DS571321_2985-26705    CATCTCAATAACTACAATAATAAATCCACAAATAATTAACATTAATGAAAAGGTTTTAATATATCCTTC
DS550441_21198-48907   CATTTCAATAACTACTATTATAAATCCACAAATAATTAACATTAATGAAAAAGCTTTAATATACCCTTC
```

```
                          16492
DS571321_2985-26705     ATATTTATCACAAAATAACCTAAATAAATGATTTAATTGATTAATTGGTGAACTATTTTCTTTATTATG
DS550441_21198-48907    ATATTTATCACAAAATAATCTAAATAAATGATTTAATTGACTAAGTGGTGAACAATTTTCTTTATTATG

                          16561
DS571321_2985-26705     AACGTCAATTGTAATACAATATTTTTCAATAATCTTTTCAATGTTTGGGAGTTTAAATATTATTGAACT
DS550441_21198-48907    GGTATCTAATTTAATACAATATTTTTTAACAATTTTTTCAATGTTTGGAAGTTTAAATATTATTGAACT

                          16630
DS571321_2985-26705     ATTATCATAGTTTATATATATTATGTCACATATTTTTTTTATTATTTGATTCGATTTTAAACATGACTG
DS550441_21198-48907    ATTATCATAATTAATATACATAATATCACATATCCTTTTTATGACCTTATTTGCTTTTAAACATGACTG

                          16699
DS571321_2985-26705     AATAAAAGAAAGCCTTAATACAAATTTAATTCCACTATACCCAAGAAAAACTATTCCTTCTATTATCCC
DS550441_21198-48907    AACAAAAGAAAGTCTTAATACAAATTTAATTCCACTATACCCAAGAAAAACTATTCCTTCTATTATCCC

                          16768
DS571321_2985-26705     AACTACTAATACCCATAAATGTCCTTTTAATTCTTTATGTAATAATATAATACAAAATGTATTACTTAT
DS550441_21198-48907    AATTACTAACACCCATAGATATCCTTTTAATTCTTTATGTAATAATCCAATACAAAATGTATTACTAAT

                          16837
DS571321_2985-26705     CAGGCATATCCCAGCAAGAATAGTAACAGACAATTGTTTGTACCATCCTCGGTGTGACTCTATTGGTTG
DS550441_21198-48907    AAGACATATCCCAGCAAGAATAGTAACAGACAATTGTTTGTACCATCCTCGGTGTGACTCTTCTGGTTG

                          16906
DS571321_2985-26705     TTCTTCTTGAAAAAATAAAACCTCATTTTTTTCATTTGTTGTAATATCCTCACTCATcaataattctga
DS550441_21198-48907    TTCTTCTTGAAAAAACAAAACTTCATTTTTTTCGTTTGTTGTAATCTCCTCACTCATcaacaattctga

                          16975
DS571321_2985-26705     ttttttaattcaagttcttttttctttgaatcatcaaacaattcttaacttttatttttatttcttgta
DS550441_21198-48907    gttttcaattcaagtttttctttctttgaaccttcaaacaattcttaacttctatttttatttcttgta

                          17044
DS571321_2985-26705     atataattctcaatcacaaaaatgaataaattttgatatttcatttcttaattaaatactattctttac
DS550441_21198-48907    ataaagttatcaatcacaaaaattaacaaattttgatatttcatttattgatttaatgatattctgtac

                          17113
DS571321_2985-26705     tattcctttctacaacataatcattcaaatacttaactttactcattgaaaacatttaaaagaatttaa
DS550441_21198-48907    cactcctttcttaaccataatcattt-tatacttaactttacctacaaaagatatttaaaagaatttaa

                          17182
DS571321_2985-26705     gacttt--cataaaaaaaacattacattgttggtatatgacaaattaaaacactattaacaaacttgta
DS550441_21198-48907    gaactcaacaaaagagaaacattgcattgtcgatatttcacaaattaaagcactatcaataaactttta

                          17251
DS571321_2985-26705     ttataagaatagttatataactttttaattactgaattaatcctaaaagaaagaattgataatttttct
DS550441_21198-48907    -tataaaaacaattctataactttataaagaatgaattaattataaaagaatgaactaataaactttc-

                          17320
DS571321_2985-26705     tttcttcatttaaaaagaaaaatctccactaacagtttaattcaaaaaaaaaataaattagtaaaaaaa
DS550441_21198-48907    tctcttcacttaaaaagtaaaatctctaccaacaatatatcataaaagaaaaataaataa--aagaaat

                          17389
DS571321_2985-26705     aataaatgaactaatatttatccttagttta---TTATCCATTGAAATTAGCATTTGTGGCATTATCAT
DS550441_21198-48907    aataaatgaactaatatttatcctaactttattaTCATCCATTGAAATTAGCATTAGTAGCATTATCAT

                          17458
DS571321_2985-26705     CTTTAGCTGTGAATTGTGGGTTATTCACAGTATTATCAACAGTATTCTTTCTTGCAGAAATAACAATAT
DS550441_21198-48907    CTTTAGCTGTGAATTGTGGGTTATTCACAGTATTATCAATAGTATTCTTTCTTGCAGAAATAACAATAT

                          17527
DS571321_2985-26705     CAACAGTTTGCTTAATTCCAATAGCAATAATTACCATAACAACAACAATTACTGCAACAACAACTGCAA
DS550441_21198-48907    CAACGGTTTGTTTAATTCCAATAGCAATAATTATCATAACAACAACAATTACTGCAACAACAACCGCAA

                          17596
DS571321_2985-26705     CAGTGGTACCAGCAGCAATAGCAGCTGTATTATCAACGTCTTGCCATAACAAACAAACAGCATCTTTAT
DS550441_21198-48907    CAGTAGTACCAGCAGCAATAGCAGCAGTATTATCAACATCTTGCCATAACAAACAAACAGCTTCTTTAT

                          17665
DS571321_2985-26705     CAACCAATTCATCATTGATCTTACAATTACCACATGCATCTAATGAGAATCTATGATCTGCATAATCAG
DS550441_21198-48907    CAACTAATTCATCATTGATTTTACAATTACCACATGCATCCAATGAGAACCTATGATCTGGATAATCAG

                          17734
DS571321_2985-26705     AGTACATTGTTAAAATCCACCCACTTTCAGATTGTAATGAACATTCACAATAGAAACAATTCTTAGCTG
DS550441_21198-48907    AATACATTGTTAAAATCCATCCACTCTCAGACTGTAATGAACATTCACAATAGAAACAATTCTTAGCTG

                          17803
DS571321_2985-26705     CTTTAGACATATTAGGGAATTGAATATCAATATCACATTGAACAAAATTAGTTTCAATTTCTCCTGTTT
DS550441_21198-48907    CTTTAGACATATTAGGGAATTGAATATCAATATCACATTGAACAAAATTAGTTTCAATTTCTCCTGTTT
```

```
                    17872
DS571321_2985-26705    CTTTGTCACAATAAGCGTCTTGACATTTATTCTTATAGAGCAATGCTTTTTCATGTCCATCAATATTAC
DS550441_21198-48907   CTTTGTCACAATAAGCATCTTGACATTTATTCTTATAGAGTAATGCTTTTGCATGTCCATCAATATTAC

                    17941
DS571321_2985-26705    TTGTCCATTTATGTGTTATCACTCCTTCAGTGATAGTTTCAACACAGGTATAATTATAACATTTATCAG
DS550441_21198-48907   TTGTCCATTTATGTGTCATCACTCCATTTGTAATAGTTTCAACACATGTATAATTATAACATTTATCAC

                    18010
DS571321_2985-26705    TACAATCCCTATCATTAGATGGCTCATCACTACATTTTGTAAATTCCTTCTTGATACAATATCCTGTAC
DS550441_21198-48907   TACAATCTCTATCTTTAGATGGGTCATCATTACATTCTGTAAATGGTTTCTTAATACAATACCCTGTAT

                    18079
DS571321_2985-26705    TTGTATCACATTTACCTGTTAATGAAGCACACCCAACATCAGCAGTATTTGATGAACAAATAGAATCAT
DS550441_21198-48907   TTGTATCACATTTACCTGTTAAAGAAGCACAACCAACATCAGCAATATTTGATGTACAAATAGAATCAT

                    18148
DS571321_2985-26705    CATACACACCACTACATTTTGGAATACTTCCAGAAGCATCACAATTAGTTTTCATACAAATAACTTCTC
DS550441_21198-48907   CATATATACTACTACATTTTGGAACACTTCCAGAAGCATCACAATTAGTTTTCATACAAACTACTTCTC

                    18217
DS571321_2985-26705    CAGGTTTACTAGGAGGAAGTGATGGACAAATAGTGACATTAGTTGGTTCACAAAGTTGGGATGTTCCAG
DS550441_21198-48907   CAGGTTGTTCAAGAGGAAGTGATGGACAAATAGAGACATTAGTTGCTTCACAAAGTTGGGATGTTCCAG

                    18286
DS571321_2985-26705    TCTTTACTTGAATGAAACAATTATTAGTTGGATTACAATCTCCTTCATATTTACAATCCCCATCTGAAC
DS550441_21198-48907   TCTTTATTTGAATAAAACATTCATTACTTGGATTACACGGTCCATCATATTTACAATCTCCATCTGAAC

                    18355
DS571321_2985-26705    AAGTCATCTGATAAATATTTCCAGATGCATCTACTTGATTTGGACATTTATCAACAAGAGTAGACTCTT
DS550441_21198-48907   AAGTCATTTGATAATGATTTCCAGATGCATCTACTTGTTCTGGACATTTATCAACAAGGGTAGATTCTT

                    18424
DS571321_2985-26705    TATCCATTCCACTACAATACTTACATAAACTTTTTACCTTTTCTTCGTTTGAATTCCATTCACATTTAT
DS550441_21198-48907   TATCCATTCCACTACAATATTTACATAAACTTTTTACCTTTTCTGAGTTTGAATTCCATTCACATTTAT

                    18493
DS571321_2985-26705    ATTCATCAAATGTTGTGCCAATAATTAATTTGTAATCAAGAACATCAAGACATTTACTAGGGAAATTCT
DS550441_21198-48907   ATTCATCAAATGTTGCACCACTAATTAGTTTGTAATCAAGAACATCAAGACATTTAGTAGGGAAATTCT

                    18562
DS571321_2985-26705    CATGTTTAGTACAATCAAATTCATCATCAATATGACAACTTCCATCACGACATACATATGGAATACCAT
DS550441_21198-48907   CATGTTTAGTACAATCAAATTCATCATCAATATGACAACTTCCATCACGACATACATATGGAATACCAT

                    18631
DS571321_2985-26705    TTATATTCTTTTTATTAGTACAATCCATTTTAATAAGTTTAGCTGGGTCAACAAGAATATTATCATATG
DS550441_21198-48907   TTATATTCTTTTTATTAGTGCAGTCCATTTTAATAAGTTTGGCTGGATCAATGAGAATATTATCATATA

                    18700
DS571321_2985-26705    AACAATACATACACTGACTATCTAAAATAACTTTTGATCCATCTTTTACAATAACTTTACATTCTCCAT
DS550441_21198-48907   AACAATACATACATTGACTATCTAAAATAATTTTTGATCCTTCTTTTACAACAACTTTACATTCTCCAT

                    18769
DS571321_2985-26705    TTGCATATTCCCATTCATAATATTGTTCACAATCACTTAATCTATCTTTGAAAAGATCTTTACAATCAT
DS550441_21198-48907   TTGCATATTCCCATTCATAATACTCTTCACAATCATTTAATCTATCTTTGAAAAGATTTTTACAATCAT

                    18838
DS571321_2985-26705    ATTCATCTGGTGCTTGACATTCTCCATCTTTACAAACAAATGGAATTCCTTGTGATGTAGTCAAACCTT
DS550441_21198-48907   AGTTATCTGGTGGTTGGCATTCTCCATCTTTACAAACAAATGGAATTCCTTGTGATGTAGTTAAACCTT

                    18907
DS571321_2985-26705    CACATTTATTAACAATTTCATTTCCATCACAAATTTTACATTCTTGAATTGCTTTATCTGTAGTTACAA
DS550441_21198-48907   CACATTTATTGACAAGATCTTCTCCATCACAAATTTTACATTCTTGAATTGCTTTATCAGTAGTTACAA

                    18976
DS571321_2985-26705    GTATACATTCTCTATTTCCACTACATACATAATTAGCATATCCATCACATTTTTCAGGTACTTTTTCTT
DS550441_21198-48907   GAGCACATTCTCTATTTCCAGTACATGTATAATTAGCATATCCAAGACATTTTTCAGGTACTTCTTCTT

                    19045
DS571321_2985-26705    TACATACATATTCCTCTTGGTCTACAACATATTGAGTCGTTCCATTAGCAGTATATTCTTTACAGACTT
DS550441_21198-48907   TACATACATACTCTTCTTGAACTACAACATACTGAATTGTTCCATTAATAATTCTTTCATCACATTTAT

                    19114
DS571321_2985-26705    TTTTGTAACAACCTTCCCAAACAATATCATGTCCTTTAAGCTCTTCACAATTATCAACATTAATACATT
DS550441_21198-48907   TTTTATAACAACCTTCCCAAACAATATCATGTCCTTTAAGTTCTTTACAGTTATCAACATTAATACATT

                    19183
DS571321_2985-26705    TTGGATCCCCATCTGTATAATCACAAACTTCGCCATTAGTACATCCATTACTAGTATCACATTTACATT
DS550441_21198-48907   TTGGGTCACCATCTGTATAATCACAAACTTCTCCATTAGTACATCCATTACTAGTATCACATTTACATT
```

```
                          19252
DS571321_2985-26705    GATATGATGTAGATGCATCACATCGACAATTTCCATTAACTTTCCCACACACTTTCATTCCAGAACTAC
DS550441_21198-48907   GATATGATGTAGATGCATCACATCTACAATTTCCATTAACCTTACCACATACTTTCATTCCAGAACTAC

                          19321
DS571321_2985-26705    ATCCAACTAAATTCATAACACAATTTCCAGTTTCACTATCACATGTAGCTTCATAACATGGATTTTCTT
DS550441_21198-48907   ATCCAACTAAATTCATAACACAGTTTCCAGTGTCAATATCACAAGTAGCTTCATAGCATGGATTTTCTT

                          19390
DS571321_2985-26705    CATATGGAGATACTTTAATACATTTCTTTTCTTGAACCATACATGTTCCAGCAGAACAATAATATTCTT
DS550441_21198-48907   CATATGGAGATACTTTAATACATTTTTTTTCTTGAACCATACAAGTTCCAGCAGAACAATAATGTTCTT

                          19459
DS571321_2985-26705    CACACTTACTTGATAATTTAGGACAATAGCTAGATGATTGTAATGTATTTGGAATACATTCTCCATTTG
DS550441_21198-48907   CACATTTACTTGATAACTTAGGGCAATAGCTAGATGGTTGTAAGGTATTTGGAATACATTCTCCACTTG

                          19528
DS571321_2985-26705    TTCTATCACATGAATAAGTTCTACAAACAAATTTTTCTACTCCAGATTCAATAATTGAAGATGGCTTAA
DS550441_21198-48907   ATCTATCACATGAATAAGTTCTACAAACAAGTTTTTCTATTCCAGATTCAGTAATAGAAGTTGGCTTAA

                          19597
DS571321_2985-26705    TATCATCAGTACAATTACTTCTAAAAGCACAGTTGTTTCCTGAACAATAAGGCTCTAAACATTTAACTG
DS550441_21198-48907   TATCATCAGTACAATTACTTCTAAATGCACAGTTATTTCCTGAACAATAAGGTTCTAAACATTTAACTG

                          19666
DS571321_2985-26705    TTGAAACACATTTATTTTGTTCAATAGTACATCCAATATCTTCAACACATTTAGCTACACCTAAACAAC
DS550441_21198-48907   TTGAAACACATTTATTTTGTTCAATAGTACATCCAATATCTTCAACACATTTAGCTGTACCTAAACAAC

                          19735
DS571321_2985-26705    TTTCTGTTGTACATTTTCTTTTTTCAATTTTATGATCAGCACTTGGATAATAAACACATTCTCCATTTT
DS550441_21198-48907   TTTCTGTTGTACATTTTCTTTTTTCAATTTTATGTTCAGCACTTGGATAATAAACACATTCTCCATTTT

                          19804
DS571321_2985-26705    CAGTATTACATGTTGTATAATAACATTCACTATGACAACACATTGGACATGTACAACGAGCCCCACCAT
DS550441_21198-48907   CAGTATTACATGTAGTATAATAACATTCATTATGACAACACATTGGACATGTACAACGAGCTCCACCAT

                          19873
DS571321_2985-26705    CACAAAATCCTTTACAAGTTTTTCTATCACATAATTCAGTATTATTTGCCATAATAGTATTATATAATT
DS550441_21198-48907   CACAAAATCCTTTACAAGTTTTTCTATCACATAATTCGGTATTATTTGCCATAATAGTATTATATAATT

                          19942
DS571321_2985-26705    GTGGAAGAGAAGTGCTTAATGATGAAACAGATGATTTAAAGTAATGTTCCCCTTTATTAACTAATTCTT
DS550441_21198-48907   GTGGAAGAAGAGTACTTAATGATGAAACAGAAGATTTAAAGTAATGTTCACCTTTATTAACTAATTCTT

                          20011
DS571321_2985-26705    CAAGTTGATATTCTTCTTCAATTGATGAGTCTAAATTAAATACATAAAATGTAGTATTATGACTATAAA
DS550441_21198-48907   GAAGTAAATATTCTTCTTCAATTGATGAATCTAAATTAAATACATAAAATGTAGTATTATGACTATAAA

                          20080
DS571321_2985-26705    GAGTATTTAACATATTAGAAGCTTTTCCTGCATATTTACTGATATAAACAATATTCTTTCCTGCTTTTG
DS550441_21198-48907   GAGTTTCTAATGTACTAGAAGCTTTTCCTGCATATTTACTAATATAAATAATATTCTTTCCTGCTTTTG

                          20149
DS571321_2985-26705    AAGACATTTGGTTATATACAGTTTCTATAGCTTTTGTTTCATTTCCTTTTCTCATAACAGTAATTCCAT
DS550441_21198-48907   AAGACATTTGGTTATATACAGTTTCTATTCCTTTTGTTTCATTTCCTTTTCTCATAACAGTAATTTCAT

                          20218
DS571321_2985-26705    TTAATACATTTTTAATATTATCCTTTGTTACTAATGAATCTGAATGATATGGATTTTGAGAGTTAATAT
DS550441_21198-48907   TTAATGCACTTTTAATATTATTCTTTGTTATTAATGAATTTGAATAAGATGGATTTTGAGAGTTAACAT

                          20287
DS571321_2985-26705    AATAAGCGAATGAAGTATCATTTGGGAATAAATCAATCAAATAATTAACAATTGAACTAAATATATTTC
DS550441_21198-48907   AATAAGCAAATGAAGTATCATTTGGGAATAAGTCAATCAAATAAGAAATAATTGCATTAAATATATTTC

                          20356
DS571321_2985-26705    TTAATGTATTAGTCATATCTAAATTAACAACAATACCAACCGCCATATTATTTGTTGGTGGTAAAACAG
DS550441_21198-48907   TTAATGTATTAGTCATATCCAAATTAACAACAATACCAACTGCCATATCCTTTGTAGGTGGTAAAATAG

                          20425
DS571321_2985-26705    TAGAAGCATGACATTTAGTAATATTATTATTTTTATAGTCAGTGATAAAATTATCAGAAAATTCTGAAC
DS550441_21198-48907   TAGAAGCACGACATTTAGTAATGTTATTATTTTTATAGTCAGTGATAAAATTATCAGAAAATTCTGAAC

                          20494
DS571321_2985-26705    TACTTCTAAATGTAATAACTTTACCTCCAATATATTCAAATCTACATGGTGACATTCCTTCTGCATATT
DS550441_21198-48907   TACTTCTAAATGTAATAACTTTACCTCCAATATATTCAAATCTACATGGTGACATTCCTTCTGCATATT

                          20563
DS571321_2985-26705    TATTTGTTACATCATATGTTTTTTCAAGAAAATATGCAGCTGTTAATGGTGGTTTAGCAGCATATTTAT
DS550441_21198-48907   TGTTTGTAACGTCATATATTTTTTCTAGAAAATATGCAGCTGTTAATGGTGGTTTAGCAGCATATTTAT
```

```
                      20632
DS571321_2985-26705   AACAAGTTAATCCATTAATTGAAACAACTGGTAATTTAGAAGTATCTGATGGAATACGAGACCAATCCA
DS550441_21198-48907  AACAAGTTAATCCATTAATTGAAACAACTGGTAATTTAGAAGTATCAGATGGAATAAGAGACCAATCTA

                      20701
DS571321_2985-26705   ATTGATATTCTCTAATAGGATATCCCATTTTCTTTCTACACATATTTTCTTCTTTTGTATCACTTTTAT
DS550441_21198-48907  ATTGATATTCTCTAACAGGATATCCCATTTTCTTTCTACACATGTTTTCAACTTTTGTATCACTTTTAT

                      20770
DS571321_2985-26705   CACGGTCATAATCTTGATTAAATATCATTTCAGTAAAATATTTACTTTGGTCTTTAGTAGGAAAATTAC
DS550441_21198-48907  CACGGTCATAATCTTGATTAAATATCATTTCAGTAAAATATTTACTTTGATCATTGTTAGGAAAATTAC

                      20839
DS571321_2985-26705   TATCAATAGTTGTCCATTCTAAATTTCTCCATGGTTGCATTGTTAAATAATAAAATACACTATACTTTT
DS550441_21198-48907  TATCAATTGATGTCCACTCTAAATTTCTCCATGGTTGCATTGTTAAATAATAAAATACACTATACTTTT

                      20908
DS571321_2985-26705   TATTTACACTTCTTGACCATGAAGCTGCATTTTTACCCATTCCCATTCTTCCTTTATCTTGGATATCTA
DS550441_21198-48907  TATTTACACTTCTTGACCATGAAGCTGCATTTTTCCCCATTCCCATTCTTCCTTTATCTTGAATATCTA

                      20977
DS571321_2985-26705   CAGTTCCAGAAAACTCATTTAAATTAATTCCTAAAGCAATTGAAGCATAAAATAATACCAAAATTATCA
DS550441_21198-48907  CTGTTCCAGAAAACTCATTTAAATTAATTCCTAATGTACTTGAAGCATAAAGTAATATTAAAATTATCA

                      21046
DS571321_2985-26705   Tttcactttctttatttgaaatttctaagttcacttcatattatttttttctttttttccttcctttc-tg
DS550441_21198-48907  Tttcactttctttattttaaaattctaagttcacttcatttgatttttttcttttttccttcctttttgtg

                      21115
DS571321_2985-26705   atttttgaaaaggtcaactttcttcggaaatttcttttatttctttttttctattttctttaatttctt
DS550441_21198-48907  ttttttgaaaaggtcagctttcttctgaattttcttttattcccttttttctattttctttcatttctt

                      21184
DS571321_2985-26705   ttctattctttttcttttcctcatttcatctctgacttttctttaagttactatttttatcttttcttt
DS550441_21198-48907  ttctattctttttctttaactcatttcatctctgacttttcttgaggttactatttttatcttttcctt

                      21253
DS571321_2985-26705   tattctcatcagattgtattattattatattttatatgatatttatctatattaattaaaatttatttc
DS550441_21198-48907  aattctcatcaaattctattat-----tattttataagatatttatctttattaagtaaaaaatagttc

                      21322
DS571321_2985-26705   tttcttttcctaaaaacgtaaagtttcgtttcaaagaatattcttaaattgaaatgaaataaactttgt
DS550441_21198-48907  taacttttcctaaaaa-attaagtttcgtttcaatgaatattcttaaattgaaatggaattttatgtat

                      21391
DS571321_2985-26705   ttttacaaaaaatgatataaaaaacattttatagaagtaataaaaaaaaaaaaataaaaaaataaaaaa
DS550441_21198-48907  ttttac-aaaaatgatatgaaaaacattttatataaaaaatgaaaaa----------------------

                      21460
DS571321_2985-26705   agtaaaaaactaaaaaaaagttatttagaaat------------aaATGACAAATGAAATGTTATATTG
DS550441_21198-48907  --taaaaaactaaaaaaaaaattatttagaaataaaggaaaaaagaaATGACAAAAGAAATGTTATATTG

                      21529
DS571321_2985-26705   TTCAATCCCAATAATTGTTAACGAACATAAATTTCAATTACTTTTAATTAGTTATGACAAAATATATAT
DS550441_21198-48907  TTCAATCCCAATAATTGTCAATGAACATAACTTTCAGTTACTTCTAAGTAGTTATGACAAAAATTATAT

                      21598
DS571321_2985-26705   TTATAGTACTTCACTTATTCAAACAACTCAAAACAGTTTATTACATGATTTTATTCCAAAACAAAATAG
DS550441_21198-48907  TTACAGTAGTTCGCTTATTCAAACAAATCAAAAATGTTTATTACATGACTTTATTCCAAAACAAAATAG

                      21667
DS571321_2985-26705   ATTTATTATTCGTTATTATAAAGAAAAGGAATTTCCAATAACATTATTAATGAAGTTAAACTCATTTTC
DS550441_21198-48907  GTTTATTATTCGTTATTATAAAGAAAAAGAATTTCCAATTACATTATTAATGAAGTTAAGTTCATTTTC

                      21736
DS571321_2985-26705   TTTTGGTTCATTTAATGGATATTTTAAACCAAAATGGTTTGAAATAAATAAATTAGAATGGTATAAATA
DS550441_21198-48907  TTTTGGTTCATTTAATGGATACTTTAAACGAAAATGGTTTGAAACAAATAAACTTAAATGGTATAAATA

                      21805
DS571321_2985-26705   TAATCAATTGGCTTATAATATTCCTCATTTTATTTCAATACCTTTATGTAATTCATCTTTCTCTCTTTC
DS550441_21198-48907  TAATCAATTAGCTTATATTACTCCTCATTTTATTTCAATACCTTTATGTAATCCATCTTTCTCTCTTTC

                      21874
DS571321_2985-26705   TCAAATGAAATGTCCTTTTTCTATTTCTTTTCTTGTTTCAAATCAATTAGCATGTGTTGCCTCAACTAT
DS550441_21198-48907  TCAAATGAAATGTCCTTTTTCTATTTCTTTTCTTGTTTCTAATCAATTGGCGTGTGTTGCTTCAAATAT

                      21943
DS571321_2985-26705   TCATTTTATTTTAATGGAAAATATGGAAGTTATAGAAGAAAAAACAATTAAATATGATTTAGACACATT
DS550441_21198-48907  TCATTTTATTTTAATGGAAAATATGGAAGTGATTGAAGAAAAGACAATTAAATATGATTTAAATTCATT
```

22012
DS571321_2985-26705 CTATTGTAATTTCATTACTTCTTTTAATCAACTTCATCATCAAAGTTATCTTAGAATTATTGTTGATTT
DS550441_21198-48907 TTATTGTAATTTTATTACTTCTTTCAATCAACTTCATCATCAAAGTTATCTTAGAGTTGTTATTGATTT

22081
DS571321_2985-26705 ACTAAATGATAAAGAAACATATATAGATGGTGGTTGTTATCTTTTTGATGAACAAGGATATCTTCTAAA
DS550441_21198-48907 ACTAAATGATACAGAAACATATATAGACAGTGGCTGTTATCTTTTTGATGAACAAGGATATCTTCTAAA

22150
DS571321_2985-26705 AGAAAAATTAATTGGTTTTACAAAAAGAGAAATAAACTCAAACAAAGGTATGAAAGGTGATCCATCTTT
DS550441_21198-48907 AGAAAAATTAATTGGTTTTACTAAAAGAGAAATTAATTCAAACAAAGAAATGAAAGATGAAATGTCTTT

22219
DS571321_2985-26705 ATTAAATTTTATTCCTAATGAAAACAATAAAAATGATTCTGACAATAAAGAAAATTGGTTTCAATTCAA
DS550441_21198-48907 ACTCAGTTTTATTCCTAAAACAAACAATAAAAATGATTATAAAGATAAAGAAAATTGGTTTCAATTCAA

22288
DS571321_2985-26705 AATGACACTTCGTATAGTAGACTATAACTTATCTTATTGTAATGAAACAAAAGTCTTTAATAAAAATGG
DS550441_21198-48907 AATGACACTTCGTATTGTTGACTATAATTTATCTTATTGTAACGAAACAAAAGTTTTTAATAAAAATGG

22357
DS571321_2985-26705 AATGATTTATGAAAATGGTTATTTATATTCATTTAACTCATTCACTTCAATAAAAAATGTTAAAACTCT
DS550441_21198-48907 AATGATTTATGAAAATGGTTATTTACATTCATTTAACTCATTTACTTCAATAAAAAATGTTAAAACTCT

22426
DS571321_2985-26705 TCAAACATTATTTATTCATCAAACTTCAGATATTAATGAATCACAAATTTATTTCCAAACAGAAAATAA
DS550441_21198-48907 TCAAACAATATTTCTTCATCAAACTTCAGATATTAATGAATCACAAATTTATTTCCAAACAGAAAATAA

22495
DS571321_2985-26705 TATAATTTCATTAAATACTGTTTTAAATTCTACTTTATTCGGTAATTTAAAAGAATTTGATAAAGAATT
DS550441_21198-48907 TATAATTTCATTAGATAGTGTTTTAAATTCAACTTTATTTGGTAACTTAAAAGAAATTAATAAAGAATT

22564
DS571321_2985-26705 TATTGATAAATTAAAGTCAATTAAAATTGATACACAAATTGACTATTCTTTCATATTAGAAGTAGTAAA
DS550441_21198-48907 TATTGATACATTAAAATCTATTCAAATTGACACCCCAATTGATTATACTTTTATAATTGAAGTAGTAAA

22633
DS571321_2985-26705 ACGAAGATTTTGGTTATTAGAAATTGAAGACCTTGAATCATGTAGAAATGTATTATTATGTATTCGAAT
DS550441_21198-48907 AAGAAGATTTTGGTTATTAGAAATTGAAGATCTTGAGTTATGTAGAAATGTATTATTATGTATTAGAAT

22702
DS571321_2985-26705 GTATTGTGATTATGGAGAAAATATGTTAAAGAATTGTTATGAATGGTTATATGATAGAATTATTTCTTC
DS550441_21198-48907 ATATTGTGATTATGGAGAAAATATGTTAAGTAATTGTTATGAATGGTTATACGATAGAGTTATTTCTTC

22771
DS571321_2985-26705 TCAAATGGATTTGACTTTATTAGACCTTTTACTTTTTTCTACTTCTGGTTTTATTGAATTTTCTATTTC
DS550441_21198-48907 TCAAATGCATTTAACTTTATTAGACCTTTTACTTTTTTCTACTTCTGGTTTTATTGACTTTTCTATTTC

22840
DS571321_2985-26705 ATTATTTCATTCTTTAAAAGAACAAGAAATATTATCAAATCTTTCGTTACTAGTAATTTTCTTAATTTC
DS550441_21198-48907 ATTATTTCATTATTTAAAAGAACAAGAAATCTTATCAAATCTTTCCTTATTAGTACTTTTCTTAATTTC

22909
DS571321_2985-26705 ATTACAAGAAAAAAGTATACAACTTTTACCAAATTTATTGACTTTATTTTCTAAACCACAACATTATAT
DS550441_21198-48907 ATTACAAGAAAAAAGTATACAACTTTTACCAAATTTATTAACTTTATTTTCTAAACCACAACATTATAT

22978
DS571321_2985-26705 GTATTTCTATAAAATGTTATGGTTGTTATTAGAAAAGTTTGACCATAAAATTGCTTTTGTAGATGAATG
DS550441_21198-48907 TTATTTCTATAAAATGTTATGGTTACTGTTAGAAAAATTTGACCATAAAATTGCTTTTATGGATGAATG

23047
DS571321_2985-26705 GTTTTTGGCTTTTTCAAAACAATATCATTATTGTTACCCTGATAAAATTATTCCTTCAATTTCTGGTGT
DS550441_21198-48907 GTTTTTTGCTTTTACAAAACAATATCATATGTGTTATCCTGATAAAATTATTCCTTCAATACAAGGCGT

23116
DS571321_2985-26705 AAATGATTTAATTTCATTCATCATAAATAGTAAAGAAAAGGATAATCATATTATTACTTATCCTCTTCT
DS550441_21198-48907 AATTGATTTAATTTCATTAATCTTAAATAGTAAAGAAAAAGATAATCATATTGTTACTTATCCTCTTCT

23185
DS571321_2985-26705 TCAACCATTCTCATCAATTCCTATTTCTTCTATTCAATCAAAAATTATTAACAAATCATTAGAACATCC
DS550441_21198-48907 TCAACCTTTCTCATCAATTCCTATTTCTTCTATTCAATCAAAAATCATTAACACATCATTAGAACATCC

23254
DS571321_2985-26705 AATTATTATTACATTTCTTCATACTGATAATTCAAAATCAATGTTATTATTTAAATTTAATAAAACAAC
DS550441_21198-48907 AATTATTATCATATTTCTTCATATTGATAATTCAAAATCAATGTTATTATTTAAATTTAATAAAACTAC

23323
DS571321_2985-26705 AATAGAATCAAATCTAATGACATTTATGAAAACTGTTTCTCAATTATGTTCTTCTTCATATCAAACGTA
DS550441_21198-48907 TATTGAATCAAATCTAATGATATTCATGAAAACTATTTCTCAATTATGTTCTTCATCATATCAAACTTA

```
                   23392
DS571321_2985-26705   TCAAATTTATAATATTTCATTTGGTAAATTTGTTGAATTTATCCCTAATTGTATTCCATATTCATTTTG
DS550441_21198-48907  TCAAATTTATAACATTTCATCAGGTAAATTTGTTGAATTTATTCCTGATTGTATTCCATATTCATATTG

                   23461
DS571321_2985-26705   TGGAAAAGTAATTCCACAAATGATTCAAATCCCTTCTTTAGTTCAATTAGATACTTCAATTTTACTAAG
DS550441_21198-48907  TAGAAAAGTAATTCCACAAATGATTCAAATTCCTTTTTTACTTCAATTAGATACTTCAATTTTATTGAG

                   23530
DS571321_2985-26705   TAATGAAGTATTACAATCATTTCAAAATTATTATGAGAAGTATTATCCTTCTTTAAATTCAATTCTTCA
DS550441_21198-48907  TAATGAATTATTACAATCATTTCAAAATTATTATGAGAATTATTGTCCTTCTTTAAATTCAATTCTTCA

                   23599
DS571321_2985-26705   ACCACAATACATAAGAAAAAAAAACTTCATTGAGTCAGGTGCATTAGTCCAAATTTTTTGGTCTTTATG
DS550441_21198-48907  ACCACAATATGTAAGAAAAAAAAAATTTCATTGAATCATGTGCATTAGTGCAAATATTTTGGTCTTTATG

                   23668
DS571321_2985-26705   TTTACTTCTTGACCGTCACTTTAAAAATGTATTGTTTAATCCATTAAATGGAAATCTTTTTCATATTGA
DS550441_21198-48907  TTTACTTCTTGATCGTCACTTTAAAAATGTATTATTTAATCCATTAACTGGAAACATTTTTCATATCGA

                   23737
DS571321_2985-26705   CCTTACTTTTGCTAGAAGTTCTTCATCACAATTTATATCTCCTATTCAATACTTTATTACTGAAGGATT
DS550441_21198-48907  CCTTACTTTTGCCAGAAGTTCTACATCACAATTTATATCTCCAATTCAATACTTTATTTCTGAAGGTTT

                   23806
DS571321_2985-26705   TGAACAACCATTTCAAACATTAAATTGGTTTGGTACAATGAAAGAAATATCAAAACAGTATGTCCATAT
DS550441_21198-48907  TGAACGACCACTTCAAATACTAAATTGGTTTAGTACAATGAAAGAAATATCAAAACAATATGTCCATAT

                   23875
DS571321_2985-26705   TATTTTTCAAAATAAAATAAAACTTTGTTCATTATTAACATGTTTTAGACGTTCAAATGCATTACAAAT
DS550441_21198-48907  TATCTTTCAAAATAAAATAAAACTTTGTTCATTATTGACATGTTTTAGACGTTCAAGTGCATTACAAAT

                   23944
DS571321_2985-26705   AGATACAATGATTGAAATTTTATATTTACCAACCGCTGAAGAATTAATCAATGAAAATAAACGTTTAAA
DS550441_21198-48907  AGATACTATGATTGAAATTTTATATTTACCAACTGCTGAAAAATTAATAAATGAAAATAAACGTTTAAA

                   24013
DS571321_2985-26705   TATGTTACAAAAAGTATCAAAAAAATGTATTTAAAAAGTATAGTATTTCTACTTCTTATAAATGAaaaca
DS550441_21198-48907  TATGTTACAAAAGGCATCAAAAAAAGTGCTTAAAAAATATAGTATTTCTACTTCTTATAAATAA--ac-

                   24082
DS571321_2985-26705   aattaattccatttattcaaTTATTTCTTAATATGAATAATTAATGGAACATGACtaatacaattaata
DS550441_21198-48907  ------tcccatttattcaaTTATTTCTTAATATGAATAATTAATGGAACATGACtaagacaattaata

                   24151
DS571321_2985-26705   ttatgatttgatgtaacaaaaaaatttatctattaaataattaacaaacTCTGATGCAGAAATTTCTTT
DS550441_21198-48907  ttacaattttaaata--taaaatatttatctattta--aattaacaaacTCTGATGCAGAAATCTCTTT

                   24220
DS571321_2985-26705   TCTTCTATAAATTTGAGTAACCGAATCAACTAAATCTTTTGACACAAAGAAATAATCTAATCTCCATCC
DS550441_21198-48907  TCTTCTGTATATTTGTGTAACAGAATCAACTAAATCTTTTGATATAAAGAAATAATCTAATCTCCATCC

                   24289
DS571321_2985-26705   TGCAGTTTTAGACTTGTCTTTAAATGAAAAGAAGGTATAAAAATCTCTTTTCTTTGGATATTTTACTTG
DS550441_21198-48907  TGCACTTTTAGACTTATCTTTATATGAAAAGAAAGTATAAAAATCTCTTTTCTTTGGATATTTTACTTG

                   24358
DS571321_2985-26705   AAACGTATCAACTAAATTTAATTCTTTTATTGTTTCATTAGTACTAGCTCGTTCTTCTTTAGTAAACCC
DS550441_21198-48907  GAATGTATCAACCAAATTTAATTCTTTTAATGTTTCACTAGTACTAGCTCGTTCTTCTTTAGTAAACCC

                   24427
DS571321_2985-26705   AGCACATCTTAATCTCGTCATTGGTTTAGCAACATCAATCCAACGTAAAGCAACATTAAGATCTCCACA
DS550441_21198-48907  AGCACATCTTAATCTTGTCATTGGCTTAGCAACATCAATCCAACGTAAAGCAACATTAAGATCTCCACA

                   24496
DS571321_2985-26705   CCAAATAACATTTTTTTTCTTTTGTAATGCCTTTAAATGTTCTCTCATATCTTTATCCCATTCTGTTGT
DS550441_21198-48907  CCAAATAACATTTTTCTTCTTTTGTAATTCTTTTAAATGTTCTCTCATATCTTTATCCCATTCAGTTGT

                   24565
DS571321_2985-26705   CCTTTTGACAAGATTTTCAAGACGTTGACCACTATTCATGACATATGAATTAACCAAATAAAATTTCTC
DS550441_21198-48907  CCTTTTAACAAGATTTTCAAGATGTTGACCACTATTCATGACATATGAATTAACCAAATAAAATTTTTC

                   24634
DS571321_2985-26705   ATATTCAACTGTAATAATTCTACCATGTTCATTCTCTTTTCCATTAATTTTTTTGGTTATACTCAATGG
DS550441_21198-48907  ATATTCAACTGTAATAATTCTACCATGTTCATTCTCTTTTCCATTAAATTTTTTGGTTATACTTATTGG

                   24703
DS571321_2985-26705   CTCTTCTTTACTTAAAATGGCTGTACCAGCATAACCTTTCTTAGCCGTAGAAGCACTGAAATAACAATG
DS550441_21198-48907  NNNNnnnnnnnnnnnnnnnnnnnnnnnnnnnnnnnnnnnnnnnnnnnnnnnnnnnnnnnnnnnnnnnnn
```

```
                       24772
DS571321_2985-26705    ATATCCATCAATCATAGGATTTACTCCATCTTGTAATTTAGTTTCTTGAACACATAAAATATCAGGATT
DS550441_21198-48907   nnnnnnnnnnnnnnnnnnnnnnnnnnnnnnnnnnnnnnnnnnnnnnnnnnnnnnnnnnnnnnnnnnnnn

                       24841
DS571321_2985-26705    TTCTTCTTTGATAAATTCATTTAATCCTTTCTTCATTGCAGCATTCCAACTTGCAACATTATATGTAGC
DS550441_21198-48907   nnnnnnnnnnnnnnnnnnnnnnnnnnnnnnnnnnnnnnnnnnnnnnnnnnnnnnnnnnnnnnnnnnnnn

                       24910
DS571321_2985-26705    AATTTTAATTTCATCATTAGCTTTTTCAGGTTTTCCTTTTTCAAAGTCTTCTGTAAATACTTCAAAGTC
DS550441_21198-48907   nnnnnnnnnnnnnnnnnnnnnnnnnnnnnnnnnnnnTTTTTCAAAATCTTCTGTAAACACTTCAAAATC

                       24979
DS571321_2985-26705    ATGCTCCTTCCAATAATGGTCATTATCTTTTTCAATATCATATAATTGGTCATCTTTTCCTTTCTTTTT
DS550441_21198-48907   ATGGTCTTTCCAATACTGATCATTATCTTTTTCAATATCATATAATTGGTCATCCTTTCCTTTCTTTTT

                       25048
DS571321_2985-26705    ATTATGAACTTCTTTTTCCTCAATTTTATCTTCTTCTTCATCTAATTTTTTTTTGGCAGTTTTACAACT
DS550441_21198-48907   ACTATGAACTTCTTTTTCCTCAATTTTATCTTCTTCTACATCTAATTTTTTTTTTTGAAGTTTTACAACA

                       25117
DS571321_2985-26705    TCTTTTCATttttgtttaagatttggagtttctcttatttagacttttaaaattaacaaaagatgaatt
DS550441_21198-48907   TCTTTTCATttttatttaacatttagagtttttcttatttagaaattttaaattaagaaataac-aatt

                       25186
DS571321_2985-26705    tatattatttttaaattacttttaaaaac-ata-accgaagtaaaaacagtaaattaacatatttttt
DS550441_21198-48907   tataatatttgttaaattacttttaaaaacaatacacagaagtaaaaacagtaaattaacatatttttt

                       25255
DS571321_2985-26705    tctTCAAACACTTATGTGTTGTCTGACAAAACTGTCAATAGCTTGTTTAAGGAATAATGAACTTGTTCC
DS550441_21198-48907   tctTCAAACACTTATGTGTTGTCTGACAAAACTGTCAATTGCTTGTTTAAGGAATAATGAACTTGTTCC

                       25324
DS571321_2985-26705    AGAGTATTCTTTTAACTTTTCTCTAAAAAATTCACACGCAAGAACATATTTTTTTATCAATGGAAAGAG
DS550441_21198-48907   AGAGTATTCTTTTAATTTTTCTCTAAAAAGTTCACAGGCAAGAACATATTTTTTTATCAATGGAAAGAG

                       25393
DS571321_2985-26705    CGAGCAGAGTCGAACCAAGATTTTTAATGCTGGTGCAGCTAAGAACTTTGTATTTTGACAAAAACTATT
DS550441_21198-48907   CGAGCAGAGTCGAACCAAGATTTTTAATGCTGGTGCAGCCAAGAACTTTGTGTTTTGACAAAAACTATT

                       25462
DS571321_2985-26705    GATGAGAAAATTAACATCAAACATTGTTTCTGACAACAAAGGAATATACACCGGTTTTAAACAATCGAG
DS550441_21198-48907   GATGAGAAAATTGACATCAAACATTGTTTCTGACAACAAAGGAGTATACACCGGTTTTAAACAATCGAG

                       25531
DS571321_2985-26705    GATAACCTGCAACGCAGCTTGTCCAAATGATCCTTCATATGGCTTTGCGTTATTACAACATACAAATAA
DS550441_21198-48907   GATAACCTGCAACGCAGCTTGTCCAAATGATCCTTCATATGGCTTTGCGTTATTACAACAAACAAATAA

                       25600
DS571321_2985-26705    AAATGCAATCCCATTTTCATGAATGAATTGCTCTAATATTTTACTGTTAATTACTTCCTTTAATAAAAA
DS550441_21198-48907   AAATGCAATCCCATTTTCGTGAATGAACTGTTCTAATATTTTACTGTTAATTACCTCCTTTAACAAAAA

                       25669
DS571321_2985-26705    TCGTAATGTTCCACTAACCTTATCCCATCCAAGTAAGTGGTGAATATTTTGTCTACAATATGCATTAAT
DS550441_21198-48907   TCGTAATGTCCCATTAACTTTATCCCATCCAAGTAATTGATGAATACTTTGTCTACAATACCCATTAAT

                       25738
DS571321_2985-26705    TTCAATCTTCTTACTTGGTTTCCTTTTAACTGGTGTTTTACTATATTTATTAACAATACCTCTAAGTAA
DS550441_21198-48907   TTCAATCTTCTTACTTGGCTTCCTTTTAACTGGTGTTTTACTGTATTTATTAACAATACCTCTAAGTAA

                       25807
DS571321_2985-26705    ATCTCGATTATTTTTAGAATATATTTCATTAGAAAATTGTGAAACTGGGTCATTGTTAATATTCTCTCC
DS550441_21198-48907   ATCTCGATTATTTTTAGAATATATTTCATTAGAAAATTGTGAAACAGGGTCATTGTTAATATTCTCTCC

                       25876
DS571321_2985-26705    ATCAACCATTTTTTCAGCCATTTCTACTACCTCTGTTGATGTCATTTCTTTACATCTCCTTTGATAGAT
DS550441_21198-48907   ATCAACCATTTTTTCAGCCATTTCAACTACCTCTGTAGATGTCATTTCTTTACACCTCCTCTGATAAAT

                       25945
DS571321_2985-26705    AGCCAAACTATTTCCTTTTGGAGCACTTTGTGGAATAGACACAATAGGAATATGAGTAGCATTAAACTC
DS550441_21198-48907   AGCTAAACTATTTCCTTTTGGTGCACTTTGTTGAATTGATACAATAGGAATATGAGTAGCATTAAACTC

                       26014
DS571321_2985-26705    TAATGTTTCATCAACAGAAACTGATATTGATGACTTAAGTAATTGAGTTAAACTGATTGTTTTATCTGA
DS550441_21198-48907   TATAGTTTCATCAACAGAAACAGATATTGATGACTTAAGTAATTGAGTTAAACTAATTGTTTTATCTGA

                       26083
DS571321_2985-26705    TTGAGAAGAAATAGAAGTATATAACTTAGAAATAAATGAATACTTTAATTCTTTATCATTATCATCTTG
DS550441_21198-48907   TTGAGAAGAAATAGAAGTATATAGTTTAGAAATGAATGAATACTTTAATTCTTTATCATTATCATCCTG
```

```
                         26152
DS571321_2985-26705      GAAATAATCAACTAATTTAAGGAATAATAACATATCTGTCTGAACAGGATTTAGCAATCTAATTCTTCT
DS550441_21198-48907     AAAATAATCAACTAATTTAAGGAATAATAACATATCTGTCTGAACAGGATTTAGTAATCTAATTCTTCT

                         26221
DS571321_2985-26705      TTCAATATTCTCATTCTTAATTGCCTCTTTAGCAATTTCAATATTTTGTTCTTTAATGCCATCAATTTT
DS550441_21198-48907     TTCTGTGTTCTCGTTCTTAATTGCTTCTTTAGCAATTTCAATACTTTGTTCTTTAATTCCATCAATTTT

                         26290
DS571321_2985-26705      CTTTAATCTCATTTTAACAGTTTGAAAAACATCTTGACGTTTAACATCTGTATCTGACTCAAATAATAA
DS550441_21198-48907     CTTTAATCTCATTTTGACAGTTCGAAAAATGTCTTGGCGCTTAACATCTGTATCTGACTCAAATAATAA

                         26359
DS571321_2985-26705      TGCTTCATGATAAAAAGTTCCCCATGCTTTCTTTAAAGAATCAGATTGATTTGCAAAATCAACTACATA
DS550441_21198-48907     TGCTTCATGATAAAAAGTTCCCCATGCTTTCTTTAAAGAGTCTGATTGATTTGCAAAATCAACTACATA

                         26428
DS571321_2985-26705      AACTTCTTTTTTTCCTTTGCAAATTCTATCAAGTCTTCCTAGTGTTTGAACGGCATGAGAACCACTTAA
DS550441_21198-48907     AACTTCTTTCTTTCCTTTACAAATTCTATCAAGTCTTCCTAATGTTTGAACAGCATGTGAACCACTTAG

                         26497
DS571321_2985-26705      TACTTTATCAACATACATGCACATTAGTCTTGGTTCATCAAATCCAGTTTGTAATTTATCTGCTGCTAC
DS550441_21198-48907     TACTTTATCAACGTACATGCACATTAATCTTGGTTCATCAAATCCAATTTGTAATTTATCTGCTGCTAC

                         26566
DS571321_2985-26705      AATAATCCTAATATTCTTTTGTTGGTCCTTTAATAGCTCTGTAATATGTTTTGAATCAGAATATGTATT
DS550441_21198-48907     AATAATCCTAATATTCTTTTGTTGATCCTTTAATAATTCTGTAATATGTTTTGAATCAGAATATGTATT

                         26635
DS571321_2985-26705      ATACATTGTATTTAATTTTTCTTCACTTTCCATTACTCCATCAATATCTGTTTCACTAAATGCACCAAA
DS550441_21198-48907     GTACATTGTATTTAATTTTTCTTCACTTTCCATTACTCCATCAATATCTGTTTCACTGAATGCACCAAA

                         26704
DS571321_2985-26705      TACTCCATAATCAAGTCCTTTTTCATTAATAACTGCTCGAAGCATTTGAGTATATTTAATAACACTCTT
DS550441_21198-48907     TACTCCATAATCAAATCCTTTTTCATTAATAACTGCTCGAAGCATTTGAGTATATTTAATAACACTCTT

                         26773
DS571321_2985-26705      TCTAGTTCTACAAACTAACATTGCAATTCCTTTAAATGAAGAAGAGTCTGTTCTTTTAATCAATTCATC
DS550441_21198-48907     TCGAGTTCTACAAACCAACATTGCAATTCCTTTAAACGAAGAAGAATCTGTTCTTTTAATCAATTCATC

                         26842
DS571321_2985-26705      AAAATGATTAATAATAAATTCAGCTCTTCTTTTCATAAGATTTTCTGTCATTCTAATTTCATCTGACAA
DS550441_21198-48907     AAAATGATTAACAATAAATTCAGCTCTTCTTTTCATAAGATTCTCTGTCATTCTAATTTCATCTGATAA

                         26911
DS571321_2985-26705      ATTTTTCGCAACTTGTAATCCATCAGCAATAGGATCTACTGCTTTAGTTTTCCCTGAAAGATTAAACAT
DS550441_21198-48907     ATTTTTTGCAACTTGTAGCCCATCAGCAATAGGATCTACTGCTTTAGTTTTACCTGAAAGATTAAACAT

                         26980
DS571321_2985-26705      TTTTGGTACAACGTAATAGTTCTTTAGAACATTCATTACTAATCCTTGACTCTCTGCTTCAGCTAAACT
DS550441_21198-48907     TTTTGGTACGACATAATAATTCTTTAAAACATTCATCACTAATCCTTGACTTTCTGCTTCAGCTAAACT

                         27049
DS571321_2985-26705      AAAACAATGAAATGGTCTAATTAGTCCATCTTTATGAGTCCCAAACAATCGAAGACATTTAGAAGTTGG
DS550441_21198-48907     AAAACAATGAAATGGTCTAATTAATCCATCTTTATGAGTCCCAAACAATCTAAGACATTTAGAAGTTGG

                         27118
DS571321_2985-26705      TGTAGCAGTAAAACTAAAATATGTAATATGACGATTTTGCCGTTGTTCTCCAGATAACATTGTATGTAA
DS550441_21198-48907     TGTAGCAGTAAAGCTAAAGTATGTAATATGACGATTTTGTCGTTGTTCTCCAGATAACATTGTATGTAA

                         27187
DS571321_2985-26705      TTTTCTTGTAGCAGCAGCTCCATGTGAGCGATGTGCTTCATCAGAAATAATAGCAATATTTAAATCACT
DS550441_21198-48907     TTTTCTTGTAGCAACAGCCCCATGTGAACGATGTGCTTCATCAGAGATAATAGCAATATTTAAATCACT

                         27256
DS571321_2985-26705      TGGTAATTGTGTTTCTACAAAACTAAACTTTTGTAATGTCGTACAAAATATTCGACATTTTATATCTTC
DS550441_21198-48907     TGGTAATTGTGTCTCTACAAAACTAAACTTTTGTAGTGTTGTACAAAATATTCTACACTTTATATCTTC

                         27325
DS571321_2985-26705      CAAATCTTCTTGAAGTTTAACTGAATTTTTAACTCTTTTTACTTCTATTTTATCAATAACTCCTAAAAC
DS550441_21198-48907     AAGGTCTTCTTGAAGTTTAACTGAGTTCTTAACTCTTTTTACTTCTATTTTATCAATTACCCCTAAAAC

                         27394
DS571321_2985-26705      AGCTTTAACAGTACAATAAAGTTGAGTGTCTAAATGAACTCTATCATTCAAAACAATAACCTTATCATA
DS550441_21198-48907     AGCTTTAACAGTACAATACAATTGTGTATCTAAATGAACTCTATCATTCAGTACAATAATTTTATCATA

                         27463
DS571321_2985-26705      TTTTGGACCTGTTTCTGTATGTAAATTATATAAAAAATGGGCTAAGGATGCAATAGTCAAGGACTTTCC
DS550441_21198-48907     TTTTGGTCCTGTTTCTGTATATAAATTATATAAAAAATGGGCTAAGGATGCAATAGTCAAAGACTTTCC
```

```
                           27532
DS571321_2985-26705     AGAACCAGTAGAGTGTTGCATCAAATAATTGTTTGATGAATGGTTATTTTGAACGTCTTCAAGCAATTT
DS550441_21198-48907    AGACCCAGTAGAGTGTTGCATCAAATAATTATTTGATGACTGTTTATTTTGAACGTCTTCAAGCAATTT

                           27601
DS571321_2985-26705     TTTTAAGCATTCCATTTGATGTACATGAGGAGTCAATCCAAATGCAGAAAATATTTGGTTGACATCATG
DS550441_21198-48907    TTTTAGGCATTCCATTTGATGTTCATGAGGAGTTAGTCCAAATGCAGAAAATATTTGATTGACATCATT

                           27670
DS571321_2985-26705     TTCAACGTCTTCTGTGGTATACCGAAGTCTCGGTTTTATTATTTCTTTTAGTGGCATCCTATCCATcct
DS550441_21198-48907    TTCAACATCTTCTGTGGTATACCGAAATCTCGGTTTTATTATTTCTTTTAGTGGCATCCTCTCCATcct

                           27739
DS571321_2985-26705     cctttactttaccccttagttcttaatttctcagttctcattcctatttttggaataaccc----tttt
DS550441_21198-48907    cctttttctttacctattagttttttatttctcagttctca--cctatttttggaataaccctctatttc

                           27808
DS571321_2985-26705     aaggttttatcatgaatcgttttgttttattt
DS550441_21198-48907    agggttttgtcatgaatcgttttgttttat--
```
