# Supplementary material for: Evidence of Gene Conversion in Genes Encoding the Gal/GalNac Lectin Complex of Entamoeba
Source: PLoS Negl Trop Dis. 2011 Jun 28;5(6):e1209. doi: 10.1371/journal.pntd.0001209 (PMC3125142; doi:10.1371/journal.pntd.0001209)
Supplement: Figure S4 — Nucleotide alignment of orthologous genome regions of E. histolytica and E. dispar used to estimate inter-specific divergence around the intermediate chain lectin orthologues EHI_006980 and EDI_276450. (PDF) [file pntd.0001209.s004.pdf]

```
Alignment: /Users/garethweedall/Desktop/igl1.fas
Seaview [blocks=70 fontsize=10 A4] on Fri Nov  5 15:25:11 2010

                               1
DS571197_51170-71066  ------gagataaaaagaagatctgaaaaaaaaaaaaaaaaaaaaagataaaaactcgaaatttctata
DS548835_12593-32400  aaaatggaaatgaaaatgagatatgaaaaaaaaaaaaaagaa------tggaacttgaaattttttaga

                              70
DS571197_51170-71066  attttttttttaaattttattttcatactgaagattaATGAGTTGTGGAAAAGCTATTGGAATTACTGT
DS548835_12593-32400  ttttttttaaaaagctttgttttcattctaaaggtaaATGAGTTGCGAAAAAGCTTTTGGAATCACTAT

                             139
DS571197_51170-71066  CATTAGTTGTTTATTATCAATTCTCATAGCATTTATTGTTGCAGGATTTGTATTAGCATTTGCTATTCC
DS548835_12593-32400  TATTGGTTGTTTATTATCAGTTCTCATAGCGTTTATTGCTGCAGGATTTGTACTATCATTTTCTATTCC

                             208
DS571197_51170-71066  ATCATATGTGTATAGTAAAGATCTTGAAACGTTAACGTCATGTGATACAAAAGGTGTTCTTGAAGGATG
DS548835_12593-32400  TTCGTATGTGTATAGTAAAGATCTTGAAAGGTTAGTGACATGTGATACAGATGGTGTACTTGAAGGATG

                             277
DS571197_51170-71066  GAGATTTTCATATAAATCAGGTTATTATTTTGGGTTACTAAATATAATTTTTGTTAGTGTGTCTTTTGG
DS548835_12593-32400  GAAATTTTCATATAAGTCAGGTTATTATTTTGGGTTATTAAATATAATTTTTGTTAGCGTATCTTTTGG

                             346
DS571197_51170-71066  GATTGCAGTCTTTTCTTTCATTCCACTTGTTGGGTTATTAGATTTCATTCTTGGGATATTTACAAGTTG
DS548835_12593-32400  GATTGCTGTCTTATCTTTCATTCCAGTTGTTGGGTTGTTTGATTTTATTCTTGGATTAATTACAAGTTG

                             415
DS571197_51170-71066  TGGTTCATTAGCGTTCTTTATCATTGGTATCATTGTACTTGCCAAAGGATTATCAGTAAATGTTAATGA
DS548835_12593-32400  TGGTTCAGTTGCATTCTTTATCATTGGTATAATTGTACTTGCCAAGGGATTATCAGTAAGTATTAATGA

                             484
DS571197_51170-71066  ATTAGACAATGAAAAAAAATTATGTTTTGAAAAACAAAATAGATGTTGTTTGGATGTATTTAATTGTTC
DS548835_12593-32400  ATTAGATGAAGAAAAAAAAATATGTTTTGAAAATCAGAATAGATGTTGTTTGGATGTATTCAATTGTTC

                             553
DS571197_51170-71066  AGAATGTACATACACAGATGAAACTGGTCAATTACAACAATGTTCAGATGATTGTAATGAAGTTGCTGT
DS548835_12593-32400  AGAATGTACATATACAGATGAAACTGGTCAATTACAACAATGTTCAGATGATTGTAATGAAGCTGTTGT

                             622
DS571197_51170-71066  CACTCCAGCAGAACACTCTGTCAAAGTTATTGGGTCTATGCTAATTGTAGGTTCAGGAATGATGGGGTG
DS548835_12593-32400  CAACCCAGCAGAACATTCCGTCAGAGTTATCGGTTCTATGCTAATTGTAGGTTCAGGGATGATGGGTTG

                             691
DS571197_51170-71066  GTATCTAGTAACAGGTATTTTCACGTTAGTAGCTGCGTTTGGGTTTTGTGCTTTTTGTTGCGAAGAATT
DS548835_12593-32400  GTATATTGTAACAGGTATTTTCACGTTAGTAGCAGCGTTTGGATTTTGTGCTTTTTGTTGCGATGAATT

                             760
DS571197_51170-71066  TTAATT-AAAACTTTTTTTGACAAaaattaatcaaagaatgttaaaattgttattttaattgttatttt
DS548835_12593-32400  TTAAttaaaaatttttttgataaaaattaatcaaagaatcttgaaattattattttatttgctatttt

                             829
DS571197_51170-71066  cattatctgctgattagaaaaaatgtattaatgtaaacatt---ctctacactttcactataatatatt
DS548835_12593-32400  cgttatctgttaaatagaaaaact-tattattgtgaataataaccgttaatcatttat--ttatatttt

                             898
DS571197_51170-71066  gtctttgttatctaaaataa----aattaatttaaataatttatggatttactaatcaattaaattaaa
DS548835_12593-32400  ggctgtagtgttttaaataagaagaattaggttata-attttatgaatttactagtcaactgaattaaa

                             967
DS571197_51170-71066  ctttcattattcattttaatgaaggttgatgagagtcaaagttatatatactctttattaatcacttct
DS548835_12593-32400  cttttgctaataattctaataatgtttgataagagtcaaagtcttataaatactcta-caattacttac

                            1036
DS571197_51170-71066  attatatcaataactattatgaacaaatttaaatagaaa----------atattaaaatgaaaaaacttt
DS548835_12593-32400  actgtatcaacaagttttattaagaaaactaattagaaagttaaaattataaaaaaattaaaaaacttt

                            1105
DS571197_51170-71066  ttggaaaatttcgaaaaaaTCAATTTTCTTTCACAAGTTTATCATTTTCTTCTGCATCGATTTTCTGGT
DS548835_12593-32400  tggagaaatttcgaaaaaaTCAATTTTCTTTCACAAGTTTAGCATTTTCTTCTTCATCGATTTTCTGGT

                            1174
DS571197_51170-71066  CTAATGGTTCAATGAATGGGATTTTAACCACTCTGTTATAAACCAATGTAGCAAAGAACAAAATCACAA
DS548835_12593-32400  CTAATGGTTCAATGAATGGGAATTTAACCACTCTATTATAAACCAATGTAGCAAAGAATAAAATCACAA

                            1243
DS571197_51170-71066  ATCCAGCAAGTTCTAATAATGACCACCATGAAAGTTTTTCACCATGTCCAGCCCAACAGAATTCTATAA
DS548835_12593-32400  ATCCAGCAAGTTCTAATAATGACCACCATGAAAGTTTCTCACCATGTCCAGCCCAACAAAACTCAATAA
```

1312
DS571197_51170-71066 TAATCATTACAAGCCAAACACATGCAGTTCTTGCACTTTCAAAGAGAGTTCTGTTAACAGCATTGAAAG
DS548835_12593-32400 TAATCATTACAAGCCAAACACAGGCAGTTCTTGCACTCTCAAAGAGAGTTCTGTTAACAGCATTGAAAG

1381
DS571197_51170-71066 CGTTAGTGACCATCATACCATAAGCATTAAGTCCAAGAACAGCAATAGCAAAGATAATAGTAATAATAA
DS548835_12593-32400 CATCAGTGACCATCATACCATAAGCATTAAGTCCAAGAACAGCAATAGCAAAGATAATAGTTATGATAA

1450
DS571197_51170-71066 TAATTGGTGTACTATGGATTAAACAAACAAAAGAATCGTATGTGTTTTCAAATGAATTGTGTTGCATTG
DS548835_12593-32400 TAATTGATGAGCTATTAAATAAACAAACAAAAGAATCATATGTGTTTTCAAATGAATTGTGTTGCATTG

1519
DS571197_51170-71066 TGCTGTAATCACTACCTGGTACGAAATATCCAAATGGACAAGCAATAAAAACAGTTTCTATAAGACCAA
DS548835_12593-32400 TACTGTAATCACTACCTGGCACGAAATATCCAAATGGACAAGCAATAAAAACAGTTTCTATAAGACCAA

1588
DS571197_51170-71066 AAATACCTTCAAATGCAACAATCTTTAATGGTGGAAGAGTTAAATTCTTCATGAAAAATTCTTCAGCAA
DS548835_12593-32400 AAATACCTTCAAAGGCAACAATTTTTAATGGTGGAAGGGTTAAATTCTTCATGAAAAATTCTTCAGCAA

1657
DS571197_51170-71066 CAATTTGTCCAGCTTGGATAAATTGGGCAATTAAAATCATAAGAATACCCATTGCTGTTTGGAATCCAG
DS548835_12593-32400 CAATTTGTCCAGCTTGAATAAATTGGGCAATTAAAATCATAAGAATACCCATTGCTGTTTGGAATCCAG

1726
DS571197_51170-71066 TTGTTTCATTTAATCCAGTTGATGGTGTACGAATTGCAGATACTCCAACTAACACAAGAGCAATTACAC
DS548835_12593-32400 TTGTTTCGTTTAATCCAGTTGATGGTGTACCAATTGCAGCTACTCCAACTAACACAAGAGCAACTACAC

1795
DS571197_51170-71066 TAATAAATATACCAATCAATTGTCCATATCGTATTTTCTTTCCAATGAAAATTCTTGAAAGGAATGATG
DS548835_12593-32400 TAATAACAATACCTATCAATTGTCCATATCGTACTTTCTTTCCAATAAAAATTCTTGAAAGGAATGATG

1864
DS571197_51170-71066 AAAAAATGATCATTGAACCTCTAAGCATTTGGAATACACTAGCTGGGGTATAAATTAAACCAAATGTCA
DS548835_12593-32400 AAAAAATGATCATTGATCCTCTAAGCATTTGGAATACACTAGCTGGAGTATAAATTAAACCAAATGTCA

1933
DS571197_51170-71066 TAAGAGTTGATGCAATCAAATCAAAGGTTGTTGGAATAAGAATAAGGAATACAGAGGCTTGTTTTTCAG
DS548835_12593-32400 TAAGAGTTGATGCAATCAAGTCAAAGGTTGTTGGAATAAGAATAAGGAATACAGAAGCTTGTTTTTCAG

2002
DS571197_51170-71066 GTATTTTTTCTCCATCTTCTAATTGAGTATAAGCAGCTTCTTTGTTGGATCTTCTTTTAAATAATTCAA
DS548835_12593-32400 CAACTTTTTCTCCATCTTCTAATTGACTATAAGCAGCTTCTTTGTTGGATCTTCTTTTATATAATTCAA

2071
DS571197_51170-71066 TCAACTTTTCAATGAAAAAACATAATGACATTGAAAAGAACATAACAATTGATTGGAAAATAGGTTTGT
DS548835_12593-32400 TCAATTTTTCAATGAAAAAACACAATGACATTGCAAAGAACATAACAATTGATTGGAAAATAGGTTTGT

2140
DS571197_51170-71066 CAAATGTGTGAGGAACTCCATTATATCCAACACATTCTACATCTAATTGAAGctattttaattaatttc
DS548835_12593-32400 CAAATGTGTGAGGAACTCCATTATATCCAACACATTCTACATCTAATTGAAGctattttaattaatttc

2209
DS571197_51170-71066 ttttttcccattttttccttcttatacaattcttttaaataccaattttgagattactaatcaaatcta
DS548835_12593-32400 ttttttctcgttttttttttcttatagaattcttttaaataccaattttgagattgctaatcaaatcta

2278
DS571197_51170-71066 aatgaaaaactaataacttttataaccaattaagtataaaaaaaaaaagaattaacaaacTTTCATTGT
DS548835_12593-32400 aatgaaaaactaataacttttataaccaattaaatgtaaaaaagaaa-----taacaaacTTTCATCGT

2347
DS571197_51170-71066 AACTGAACTCCCTGTACCAAAAAGAAGCATtccacacaaaacatgaattttatgaagcaattctttgct
DS548835_12593-32400 AACTGAACTCCCTGTACCAAAAAGAAGCATtccacacaaaacatgaattttatgaagcaattctttgct

2416
DS571197_51170-71066 gatcaacatgatatttggatttattagttcttacttttcctttaagacagaattgtgaaaatttttttg
DS548835_12593-32400 gatcaacatgatatttggatttattagttcttacttttcctttaagaaagaattgtgaaattttttttg

2485
DS571197_51170-71066 taaagagtattaaattagaaacactgaaaaaagaagattttaagttttcggaaatttggtgttcattgg
DS548835_12593-32400 taaagagtattaaattagaaacactgaaaaaagaggattttaagttttcggaaatttggtgttcattgg

2554
DS571197_51170-71066 aaaagtcctaaccaaccaacgaggattagacattaagaaattaCTAATTGTTTAATACACCAACAACAT
DS548835_12593-32400 aaaagtcct-accaaccaacaaggattagacataaagaaactaCTAATTATTAAATGCACTAACAACGT

2623
DS571197_51170-71066 AACTTTTCCATTCATTTATTGGGATTTTTTTTGAAACTATTTCATCAAATATTTGAATAACTGATTTTT
DS548835_12593-32400 AACTTTTCCATTCATTTATTGGAATCTTTTTTGAAACTATTTCGTCAAATATTTGAATGACTGATTTTT

```
                              2692
DS571197_51170-71066   CATATACTTGTTGATGACTTAATTTATATTGAACAACAATAGTAGCTAATAGCTCGATTTGTTCTTCTT
DS548835_12593-32400   CATATACCTGTTGATGACTTAATTTGTATTGAACAACAATAGTAGCTAATAGTTCTATTTGTTCTTCTT

                              2761
DS571197_51170-71066   CTTTCATTAAACAAAGCCATTTTTTGTCATTAAATTTATCCACATATGATTTTAATACTTTGCATTGTT
DS548835_12593-32400   CTTTCATTAAACAAAGCCATTTTTTATCATTAAATTTATCTACATATGATTTTAATAGTTTGAATTGTT

                              2830
DS571197_51170-71066   CTTCTATTCTCAAATGACTTTCTTGTAATTCTTTAATTTCTTTTTCCATATCAATACATTCCTTTAATT
DS548835_12593-32400   CTTCTATTCTCAAATGATTTTCTTGTAATTCTTTAATTTCTTTTTCCATTTCTATGCATTCGTTTAACT

                              2899
DS571197_51170-71066   TCTTTTCTTCTTCATTTCTTATAACAACTCCTTCTTTTTTTTTGGTATTTAATTTTTCTTGTAATTCTT
DS548835_12593-32400   TCTTTTCTTCTTCACTTTTTATAACAAGTCTTTCCTTTTTTTTAGTATTTAATTTTTCTTGTAACTCTT

                              2968
DS571197_51170-71066   GAATTTCTTTCATTAAATCATCTTTGTCACTTTGTTTATCATCATTTTGTTGAATTAACTCTTTCTCTT
DS548835_12593-32400   GAACTTCTTTCATTAAATCATCTTTATCACTTTGTTTTTCATTATTTTGTTGAGCTAATTCTTTCTCTT

                              3037
DS571197_51170-71066   TCAAAATTCCAATTTCTTTATTGTATTTTAATTCATTTAATTGGTGTTTCATTGTTTCTTCTTTCTTTT
DS548835_12593-32400   TCAAAATCCCTATCTCTTTATTATATTTTAATTCATTTAATTGGTGTTTCATTGTTTCTTCTTTCTTTT

                              3106
DS571197_51170-71066   GTTCTTCTTGTCTTTTTTTACTCTCTTCTGCTATTATCTTCTTAAACTCATTTGTTTGTTGGACAAAAT
DS548835_12593-32400   GTTCTTCTTGTCTTTTTTTGCTTTCTTCTTCTATTATCTTTTTGAACTCATTTGTTTGTTGAACAAAAT

                              3175
DS571197_51170-71066   AATCTTCTAACCCTTTCATATTTTCAACCATTTGTTCATTTTCTTTGTTTTGTTCTTCAATACTTATTT
DS548835_12593-32400   AATCTTCCAGCCTTTTCATGTTTTCAATCATTTGTTCATTTTCTTTATTTTGTTCTTTAATAGTCATTT

                              3244
DS571197_51170-71066   CCATTTCTTCAATAGAACTAATTTCTTTATTCATAACAATTCGTTCTTTATAACAAATTGTTTTAATTT
DS548835_12593-32400   CCATTTCTTCAATAGATCCAATTTCTTTGCTCATAACAATTCGTTCTTTATGACAAATCGTTTTAATTT

                              3313
DS571197_51170-71066   CATTTAATACACTTTCAATTTTTTCTTGTTTATCATTTATATTCTTCAATATCTCCTTAATTCTTAATT
DS548835_12593-32400   CATTTAACACTCTTTCAATTTTTTCTTGTTTATCATTTACATTCTTCAGTATCTCCTTAATTGTTAATT

                              3382
DS571197_51170-71066   CAGTATTATCTCTTCTTTTTTCTACTTCATTTGCATTATCTTTTTGTGTTTTTTGTGCTTCTTTCATCT
DS548835_12593-32400   CGGTGTTATCCCTTATTTTTTCAATATCATTTACGTTATCTCTTTTTATTTCTTGTATCTCTTTCATTT

                              3451
DS571197_51170-71066   CATAAATTCTTTTTCTCTTGGCTTCGTTTTCTTTCTTGATTCTATTAATTTCTTCGTCTAATTTTTGAA
DS548835_12593-32400   CATGAATCCTTTTTCTCTTGGCTTCGTTTTCTTGCTTGATTCTATTAATTTCATTATCTAATCTTTGAA

                              3520
DS571197_51170-71066   GCTCTTTTGTATTTTCAATATCTTTCTCATTCTCTATTAATCTTTCTTCATGAGAAATAATTAAATTAT
DS548835_12593-32400   GCTCTTTTGTTTTGTCAATACCTTTTTCATTCTCTGTTAATCTTTCTTCATGAGAAATAAGTAATTTAT

                              3589
DS571197_51170-71066   TTTGATTGGATTCTTCTTGTGTCTCTTTGACTCCAATTTCTTGTGAAACAATTTTTTCATTAACTTGTT
DS548835_12593-32400   TTTGATTAAGTTGTTCTTGTGTCTTTTGGACTGAAATCTCTTGTGAGATATTTTTTTCTTTAATTTGTT

                              3658
DS571197_51170-71066   CTTCAGAAAGATTTATTTCAGAATGTTTTTTATCTTCATTAATTATAACTTGTGTATCACTTTTAGTAA
DS548835_12593-32400   CTTCTGAAAGATTTATTTCAGAAAGTTTTTTATCTTCATTAATTATAATTTTTGTATCACTTTTAGTAA

                              3727
DS571197_51170-71066   TTTCTTCTGACTCTGCTTTTTCTATATTTTGTTGAACTGCTTCATCACGTTTATCACTTTTTTGTTTTG
DS548835_12593-32400   TTTCTTCTGGCCCTACTTTTTCTCTACTTTGTTGAACTATTTCTTCATGTCTATCACTTTTTTGTTTTG

                              3796
DS571197_51170-71066   TATTTGGTTTTTTTCCTTTTGGTTTAAGAACATTTTTACGGTTTTGCATTTTCATAATTTTTATTTCAC
DS548835_12593-32400   TGGTTGATTTTCTTCCTTTTGGTTTAAGAACATTTTTACGACTTTGCATTTTCATAATTTTTATTTCAC

                              3865
DS571197_51170-71066   TTTCTTTAATATCTTCTTTGATAGATGCAACAACTGAATCAATCTCTTCTTTTTCTTCTGGTTTTTGAT
DS548835_12593-32400   TTTCTTTAATGCCTTCTTTAATCGATGCAACTACTGAATCAATTTCTTCTTTTTCTTCTGTTTTTTGAT

                              3934
DS571197_51170-71066   TGTCTGGATAATCAGTTGTGTATAATTTTTTCTTTTTTGAGGAAGAGACACTTCCTATTTGATTTTTTA
DS548835_12593-32400   TGTCTGGATAATCGTCTGTGCACAACTTTTTCTTTTTTGAGGAAGACACACCCCCTATTTGATTTTTTA

                              4003
DS571197_51170-71066   TTTTATCTGTTTTCTCTGTTAATGCCACTTCTGAATTATTTATTACTTGGTGTTTACTTGTTCCTTCAG
DS548835_12593-32400   TTTTATCTGTTTTTTCTGTTAATACCTCTTCTGGATTGTTTATTGTTTGGTGTTTACTTGTCCTTTCAT
```

```
                         4072
DS571197_51170-71066     AAATTACCTTTTTTTCAGCTTCAATAACTGAAGCACTTTTTTGTATCTTTCTCTTCTCCATaaacttgt
DS548835_12593-32400     AATTTAATTTTTTTTCAGCTTCAAAAACTGAAGCACCTTTTTGTATCTTTCTCTTCTCCATaaacttgt

                         4141
DS571197_51170-71066     ttttttccagatgttttgaaatagacaaaattaaaaatgatataaataacaaaaatgtgtatttaaaaa
DS548835_12593-32400     ttttttccagatgctttgaaataaccaaaattaaaaatgatataaataacaaaaatatgtatttaaaaa

                         4210
DS571197_51170-71066     caaagagctatt-tttgaagaggaaagaaaaaagaaagctcagtTTATTGTGACAAAGACAATTGTTTT
DS548835_12593-32400     catagagcaattgtttgaggagaaacaaaaaaagaaggctcagtTTATTGTGACAAAGACAATTGTTTT

                         4279
DS571197_51170-71066     TTTAAAATTTCGATTGTTTGTTGTTGTTTAGAAGAAACTTCTTTTAACTTACGAAGAATACTTTCGAGT
DS548835_12593-32400     TTTAATATTTCGATGGTTTGTTGTTGTTTAGAAGAAACTTCTTTTAATTTACGAAGGATACTTTCAAGT

                         4348
DS571197_51170-71066     TGTTCAATCTTTCCTAATAGAGAGAATTGTTCATCATACTTTGGTTCTTCCATAAAGGATTGTGTCATA
DS548835_12593-32400     TGTTCAACCTTTCCTAAGAGAGAGAGTTGTTCATCATATTTTGGTTCTTCCATAAAGGATTGTGTCATG

                         4417
DS571197_51170-71066     CATTGACGTTTACTAAGTTGTCTTGAAGCAAAAGAAGGACTTTGTTTATCCGTAACTATTCCTTTATCA
DS548835_12593-32400     CATTGACGTTTGCTATGTTGTCTTGAAGCAAAAGAAAGGCTTTGTTTATCTGTAACAATTCCTTTATCA

                         4486
DS571197_51170-71066     GCAATTGGTGTTGAAGGAAGAGATAAAGGAGAGGAGATAATTGAAATAGGACGACCTTTAGGGGTATGA
DS548835_12593-32400     ACAATTGGTGTTGATGGAAGAGACAAAGGAGAAGAGATAACAGAAATAGGATGACCCTTAGGAGTATGA

                         4555
DS571197_51170-71066     ATAGGAGATTGCCAAGTAGTTGAAGTATGAAATTGTTCTGAGTATTGAATAGGCGTAGATTTTTTTAAA
DS548835_12593-32400     ACAGGAGATTGCCAAGTTGTTGAAGTATGAAACTGTTCTGAGTATTGAAGAGATGTAGATTTTTTTAAA

                         4624
DS571197_51170-71066     TCAGCTTCAAGTGAAACAGCGTCAGGTTCTGATCTTTTTCTTTTTACTTTAGAGTCTGATATTTGATCT
DS548835_12593-32400     TCATCTTTAAGTGAAACAGCATCAGGTTCTGATCTTTTTCTTTTTACTTTAGGGTCTGATATTTGATCT

                         4693
DS571197_51170-71066     TTAGAAGGAAGACTTTTATTTATTTCTTTCAACAATTCTATGTGTGAACGTGGTAAACTTCGTTTATCT
DS548835_12593-32400     TTAGAAGGGATATTTTTATTCATCTCTTTCATTAACTCCAAGTGTGGACGTGGTAAACTTCTTTTTTCC

                         4762
DS571197_51170-71066     GGTTTTTCTTTTGGTTTTAATTGTTT---------TTCTTCAAATATTTCTTCCTTCTTAAATTCTTTC
DS548835_12593-32400     GGTTTTTCCTTTGGTTTTAATTGTTTTAATTGTCTTTCTTCAAACTTTTCTTCTTTCTTAAATTCTTTC

                         4831
DS571197_51170-71066     TTTTCAATTGGAATTTCACAGCTTTTTTCTTCTTTGATGATTCCCATCTCTTTTTGTTTTTGTGTAATT
DS548835_12593-32400     TTTTTATTTGGAATTTCACACCCCTTTTCTTCTTTTATAATTCCCATCTCTTTTTGTTTTTGTGTAATT

                         4900
DS571197_51170-71066     TCTGCAATAAAAAGTTGGTGAGGTGTTTGAGGTGGTATACGACATTCTTTTGCAGTTGTTGTTTTCTTT
DS548835_12593-32400     TCTGCAATAAAAAGTTGGTGAGGTGTTTGAGGTGGTGCACAAGATTCTTTTGGAGTTGTTGTTTTCTTT

                         4969
DS571197_51170-71066     TTGTAAGACATTatgattataacttagatctttgtctaatctaacgtgagattgtaatgttattatttt
DS548835_12593-32400     TTGTAAGACATTatgattataacttagttctttgtcttatctaacaaaagaatgtaatgttatcatttt

                         5038
DS571197_51170-71066     caaaaacatcttaactttagaattttgtaatatttcgttatttaaaagcttattttaataattttcatt
DS548835_12593-32400     caaaaacaacttaacattagaattttataatagttcgttacttaaaaatatacttttataattttcatt

                         5107
DS571197_51170-71066     ataatatatacaaactatatatattattacgttaataaacttaatt-acagtatttgatagtaaattta
DS548835_12593-32400     ataatatacacaaactataaataacattatgtaaatgaaataaattaaccgtatttgatggtaaattta

                         5176
DS571197_51170-71066     cttatttatatttattgtaaacttacattatatttctgtaagaaaaacttacataaaatctagaaagaa
DS548835_12593-32400     attatttatatttattgtaaacttatactatatttctgtaagaaaaacttacataaaatcttgaaagaa

                         5245
DS571197_51170-71066     tacattaagaatctgtctaaatattattgattatttaacttaaataaataaaaagtgaaaaccttaaag
DS548835_12593-32400     tacattaagaatctgtctaaataacattgattctttaacttaaataaatgaaaagtgaaaactttaaag

                         5314
DS571197_51170-71066     agaaattaaattaagtcatatcttgaataaaaaaaagcgATGAGCACTACTGTAAGTGAAGATGATAGT
DS548835_12593-32400     agaaataaaattaagtcatatcttgaataaaagaaagtgATGAGCACTAGTGTAAGTGAAGATGATAGT

                         5383
DS571197_51170-71066     ATCACATCACCTAAAAACATCCAATACGAGATTGATGAGGCTATTGAAAAACTAAGAACGGTTATAGAT
DS548835_12593-32400     ATCACATCACCTAAAAACATCCAATACGAGATTGACGAAGCTATCGAAAAACTAAGAACAGTTATAGAT
```

```
                 5452
DS571197_51170-71066  CTTTATAACAAGGCTCTTAAAGATAAAAATCGTTTCGAAGAAGTTGGTAAGAATGCAGACGATAAGATA
DS548835_12593-32400  CTCTATAATAGGGCACTTAAAGACAAAAATCGTTTCGAAGAAGTTGGTAAAAATGCAGACGATAAGATA

                 5521
DS571197_51170-71066  GAATTACTTAAACGGGATTTACAAGAGTGCTTGGCAAATGAACAAAAACTTCAAAACGAATTAATTGCT
DS548835_12593-32400  GAATTACTTAAACAAGACTTACAGGAATGCTTGGCAAATGAACAGAAACTTCAAAACGAATTAATTGCC

                 5590
DS571197_51170-71066  GAAAAGGAGGAACATGAAAAAGCTGAAATAGAATTAAAACAAAAAACAGTCGGAAGTAAAATTCTTACT
DS548835_12593-32400  GAAAAAGAAGAACATGAAAAAGCAGAAAAAGAATTAAAACAAAAAACTATTGGAAGTAAAGTTCTTACT

                 5659
DS571197_51170-71066  GCTCGTTGTCTTGTTTACACAAAAGATGGAATGATAGAGGATGTTGAAATGATTCCTTGGGATGATGAT
DS548835_12593-32400  GCTCGTTGTCTTGTTTACACAAAAGATGGAATGATAGAAGACGTTGAAATGATTCCTTGGGATGATGAT

                 5728
DS571197_51170-71066  TTCGATGCTTCTGCAGATGTTATTAGAAAAATTAAAAAGACACAAACAAACATAATCACTGAAAAGAAA
DS548835_12593-32400  TTCGATGCTTCTGCAGATGTTATTAGAAAAATTAAAAAGACACAAACAAACATAGTCACTGAAAAGAAA

                 5797
DS571197_51170-71066  GAAATTTCAGAAAATCTTAAAAATACTCAACCTCAATCTCAATCTAAGTTACCTCTTTCAAATGAACAG
DS548835_12593-32400  GAAATTTCAGATAATCTTAAAAACAGTCAGCCTCAATCTCAG------TTACCTCTTTCAAATGAACAA

                 5866
DS571197_51170-71066  CCAACTTCATTTAATACCACTACTATCCGTCCAAAAAATTTTTCAGTTAGTCAAATTCAAAAGGACCTT
DS548835_12593-32400  TCAACCTCATTTAATACAACT---ATTCCTCCAAAAAATTTTTTAGTTAGACAAATTCAAAAGGACCTT

                 5935
DS571197_51170-71066  GAAAAGTTTGAAAAAAGGTCTGAGGATATAATTAAAATTACACAATCACCAAATCATTTAGAAAAGAAA
DS548835_12593-32400  AAAAAATTTGAAAAACGGTCTGACGACATTATCAAAATTACACAATCACCAAATCACTTTGAAAAGAAA

                 6004
DS571197_51170-71066  ATCATTCACACTCAATCAAACCAAAGTTTTTGGTTTGTAGTTATTATTATATTCCTTTTGACAGTCATT
DS548835_12593-32400  ACCAACCTCAATTCATCAAACCAAAATTTTTGGTTTGTAGTTATTATTATATTCCTATTGACAGTCATT

                 6073
DS571197_51170-71066  TTATTTTTGATTGCATCTATTCTACTCCATTAAtttaatttgttttagtttcttcttcgtatcatcaga
DS548835_12593-32400  TTATTTTTGATTGCATCTATTCTACTCCATTAAtttaatttgtttcagtttcttctttgtatcatcaga

                 6142
DS571197_51170-71066  ttggttataaaaagaaaaaagaaaactgataaacaaga------aagataaaaaataaattcttattta
DS548835_12593-32400  cta--ta-aaaaagaaaataaagaaatgataacaaagatgaaagaacataacaaataa-----tattta

                 6211
DS571197_51170-71066  aaaatagaactgacaaaaaattcattgtgcaaaaaaaacacaattaatttttg-aaaaacaaaaatta-
DS548835_12593-32400  aaaataaaaataacataaaatttgttaatc--aaaaaacacaactgattactataaaaatgaatattaa

                 6280
DS571197_51170-71066  --aaaaattcttttatttacttttttattcttcaattctcattatt-tcatttctttcattaaaactaa
DS548835_12593-32400  ataaaatttcagttattttctctcatttttgttaatttattttctcattatttctttctttaaaattta

                 6349
DS571197_51170-71066  cttaaatatcatttatcttataaaccattaaaa-tatttCTTATTTGATTATTTAATTATGGATCAT--
DS548835_12593-32400  cttaaat---ctttttcttctaaacctttaaaattatttcttatc---ttatttaattatggatcatta

                 6418
DS571197_51170-71066  TATTCTTTTAGAACATAAATGCTAACATGACTATCATCATCATGATGAAAATGCCTTTAGCTCCATTAC
DS548835_12593-32400  tattcatTTAGAACATAAATGATAACATGACTATCACCATCATAATGAAAATTCCTTTACTTCCATTGC

                 6487
DS571197_51170-71066  ACTTTCCATCTTTTAATTTCCATCCATCTTCACATGTTGTGCATTCGCCTTCTCCTGTACATTTAGTAC
DS548835_12593-32400  AGTTTCCTTCTTCTAATTTCCATCCTTCTTCACATGATGTGCATTTTCCTTCTCCTGAACATTTAGTAC

                 6556
DS571197_51170-71066  AATGTGGCATATGTCTTGAACATGGAATACATGATCCCCAACTAGATGTGTAATATCCTTCAACACATA
DS548835_12593-32400  AATGTGCCATATGTCTTGAACATGGAACACATGATCCCCAACTTGATGTGTAATATCCTTCTACACAAA

                 6625
DS571197_51170-71066  CACATTCTCCTCCTTTTGCTTCTAAATATTCAGCATTGCATCTCAAACAGTCTTTGGAATTACATGAAT
DS548835_12593-32400  CACACTCTTCTCCTTTCACTTCTAAATATTCTGCGTTGCATTTCAAACATTCATTTGAATTACATGAAT

                 6694
DS571197_51170-71066  CACAAAACTCATTAACTTTAGCTTTGCATGCTTGACATTCAATTTTATTATCTTCTGTAGAAGTGCCTT
DS548835_12593-32400  CACAGAATTCATTAACTTTGGCTTTACATGATTCACATTCTATTGTATTATCTTTTGTTGAATTATTTT

                 6763
DS571197_51170-71066  CAACATATCCATATGCACAAACACATGCTCCATTCACTGGTACTTCAGCAATATGATCTTTTGTTGAAC
DS548835_12593-32400  GAATATATCCATATGCACATGTACATGCCCCATTCAATGGGAATTCTGAAATATGATCTTTTGTTGAAC
```

```
                                 6832
DS571197_51170-71066  ATTTAGTACATTCTTCTACTATTTTCTTCTTCTCAGAATTACATCCTGTTTCTTGTTCTTCACAATCTT
DS548835_12593-32400  ATTTTGTACAATGTTCTACTACTTTCTTCTTCTCTGAATTACATCCACTTTCTCCTTCTTCACAGTCCT

                                 6901
DS571197_51170-71066  TCTTTACTCCAACACATTTAGTGCATGGTGATGTGCATGGTATACATGTTCCTTTAATCTCATCAAAAT
DS548835_12593-32400  TCATTACTCCAATACATTTAGTGCATGGTGATGTACATGGTATACATGCTCCTTTAATTTCATCAAAAT

                                 6970
DS571197_51170-71066  AGAATCCATCTACACATGAATTACATCCATTTCCACTAATTACCTTGTATGTATCACTCTCACATACTG
DS548835_12593-32400  AGAATCCATCTGCACATGAATTACATCCTTTTCCACTAATTACCTTATATGTTTCGCTCTCACACACTG

                                 7039
DS571197_51170-71066  TACATTCTGCGGCACTACTACATTCACTACAATGATTGGTACATGGCTTACATTCACCATTTGACATAT
DS548835_12593-32400  TACATTCTGTAGCACTAGTACATTCACTACAATGATTAGTACATGGTTTACATTCTCCATTAGACATAT

                                 7108
DS571197_51170-71066  AATAACCTGTTTTACATCCATCACATTTACTTCCTACCTTTAATGGATCAGCACATGTTAAACAAGTGT
DS548835_12593-32400  AATATCCTGTCTTACATCTATCACACTTACTTCCTACTTTTAATGAATCAGTACATGTTAGACAGATGT

                                 7177
DS571197_51170-71066  CTTTATCTGAACATGTTGCACATTTATCATCACATTTAGCACACCCATT------------------CT
DS548835_12593-32400  CTTGATCTGAACAAGTCCCACATTCAGGATTACATTTAGCACATCCATTATTTTTATTACCTTCTCCCT

                                 7246
DS571197_51170-71066  TTTCTCCTTCTTTTGGATAATAATTAGACTTAGTACATGTAGTACAATTACTATAAGGACTACGTCCAC
DS548835_12593-32400  TTTCTCCATTTTTTGGATAATAGTTAGACAAAGTACATGCAGTACAATTGCTATAAGGACTACGTCCAT

                                 7315
DS571197_51170-71066  TAAAACCATCTGAACATTCTGTTGCATAGATAAATCCTGTTCTACTATCAACATTACATGAATCAGTTT
DS548835_12593-32400  TAAAATTTTCTGAACATTCTGTTGCATAAATATATCCTGTTTTAGCATCAACATTACATGTATCAGTAG

                                 7384
DS571197_51170-71066  TTTTACATCCTGGATATTTTGAAAGGTCAGAGCAACTTTGACACGTTCCATTTTCAAGTTGAACTGTTC
DS548835_12593-32400  TTTTACATCCTTCATATTTTGAAAGTTCTGAACAACTCTTACATCTTCCATTCTCAAGTTGAACTGTTT

                                 7453
DS571197_51170-71066  CTGTTGGACAAGCACATTCTGGTGCTTTAATATCTTTTAGCCTTTGTTCAGTGCATGTCACACATTCAA
DS548835_12593-32400  CTTTTGGACAAGCACATTCTGGTGCTTTAATGTCTTGAGAAGTTGTATCAGTACATGTCAAACATTCAA

                                 7522
DS571197_51170-71066  CTTTCTTTGAAGTGGTATTATATTTACATGTTTTACATTTTGCAGGACATGGACTACAATACACTCCAT
DS548835_12593-32400  TTTTCTTCGTTGGGGTATTATATTTACATGTTTTGCACTTTGCAGGACATGGACTACAATACACTCCAT

                                 7591
DS571197_51170-71066  CTGTTGCATTTTCAATTTGATAAAATCCATCTTTACATACAGTACATGGCTTTTCTCC---------GG
DS548835_12593-32400  CCGTTGGGTTTTCAATTTTATAAAATCCATCTTTACACTCAGTACATTGTACATTTGAGCCACTAGCAG

                                 7660
DS571197_51170-71066  TAAGTATTAAGTTAATGTCTTCACAGGTAATACATGCTGTTGCTGAACATGCTGCACAATGTCCATATT
DS548835_12593-32400  TAAGAATCAAATTATTATCTTCACATGTAAGACATGCCGTTGCTGAACAAGATGAACAATGTCCAAACT

                                 7729
DS571197_51170-71066  GAGATTGTTTAGTAGAACAAAGTTGGCATTTATTATTTTCATCTTTAAAAGCATTAGGAGAACATTGCG
DS548835_12593-32400  GAGCTTGTTTAGTAGAACAAAGAACACACTC---ATTTCCATTTCTATAAGCATTAGGAGAACATTGAG

                                 7798
DS571197_51170-71066  TACAAGCACCAACCTGTGAACCAACAATATAAGCTGAGTCATCACAGTCAAGACAGGTAACAAATCCAT
DS548835_12593-32400  TACAAGCACTAATTTGTGAACCAACGATATAAGCAGGATTATCACAATCAAGACATGTAACATATCCAT

                                 7867
DS571197_51170-71066  CACTAATTTCATATTTACCTTCCTTTTTACAATTATTATTTAGTTGTGCACAACTTTTCTTCTTATCTT
DS548835_12593-32400  CACTAATCTCATACTTACCTTCATTTTTACAGTTTTTATTTAATTGTGCACAACTTTTCTTCTTATCTT

                                 7936
DS571197_51170-71066  CTGCATTTGGTCCTTCTTTATAACCATCATTTATGCAAGCGCATTCATTTTCTTTATTTCTTGTAGTAA
DS548835_12593-32400  CTTCATTTGGTCCTTCTTTATAATCATTACTAACACATGCACATTTTTTGTCTTTATCCCTTGTAGTAA

                                 8005
DS571197_51170-71066  CACCTTGAATATTTTCATAACATTCACTACACACAACTTTACTTTTAGAACTATCATAAGAGCAACTTG
DS548835_12593-32400  CTCCTTGAATATTTTCATAACATGAAGTACATATGACATTATTTTTAGTTGAATCAAGAGCACAACTTG

                                 8074
DS571197_51170-71066  AACAACTAGCTGGACACTTTTTACAGACATTATCTTTGTCAAAATAATGTGCATCACCACAAGAACATT
DS548835_12593-32400  AACAGCTATCTGGACACTTTTTACAAGTATTGTCTTTGTCAAAATAATGACCATCACCACAAGAACATT

                                 8143
DS571197_51170-71066  TACCATCCTTTCCTACATGTGATCCAACTGGACATTGTGTACAAATATTTCCATTTGCTGTTCTTTCAC
DS548835_12593-32400  TACCATCACTTCCTACATGTGATCCAACTGGACATTGTGTACAAATATTTTCATTTGCTGCTCTTTCAC
```

```
                      8212
DS571197_51170-71066  ATGATGGAAGTTTGGTTGTTATTGAAACACAATCTGATGCTTTGTCAATTGATGTTGGAATATGTTTGT
DS548835_12593-32400  ATGATGGAAGTTTAGTTGCTATTGAAACACAATCTGATGATTTATCTATAGATGTTGGAATGTATTTTG

                      8281
DS571197_51170-71066  CATTACATTCACAGTCATTTGAAACATTTCTTAATTGACCAACACAACCAATAGTAATTGTATT---AT
DS548835_12593-32400  CTTCACATTCACAATCATTCTTAACATCTCTAGACTTACCAACACAACCAATGGTAATTTTAGACATAT

                      8350
DS571197_51170-71066  CTGAAGTACCTTTAGTAACACTGAAATAATATGGTAAATAAAGGTCTTTAAGTGGACAATAATAAGTAT
DS548835_12593-32400  CTTTAGAGTTCTTAGTGACATTAAAATAGTAAGGTAGGAAAAGATCTGTAAGTGGACAATAATAAGTAT

                      8419
DS571197_51170-71066  TATCAGATGCTTTAAATGCATTCAAATATTGTTCATATTGATCATATCCTCTCATCTTACACACTTGAT
DS548835_12593-32400  TATCTGATGCTTTAAATGCATTCAAATATTGTTCAAATTGATTGTACCCTCTCATTTTACACACTTTAT

                      8488
DS571197_51170-71066  GGTTGTTACCACTAGTACCAAGGACTGATTTAGTGTCAAAATTATAGCAATGAGTTCCATTCAATGTAA
DS548835_12593-32400  GATTTTTACCACTAATACCAAGTACATCATTAATAATAGTATTGTAACAATGAGTTCCTTTTAGTGTAA

                      8557
DS571197_51170-71066  GTCCATTCTCACATTTATAACAAATAGTATCATCATCACTACATAATAAACATCCTGGAAGTTTGCATG
DS548835_12593-32400  GTCCATTCTCACATTTATAACAAATAGTATCATCATCACTACATGACAAACATCCTGGAAGTTTACATG

                      8626
DS571197_51170-71066  GTCCATCAGTTGATGTTAAGTGTGAGCTTCTAAGATTTAACTTACCTGGAACTGGTGTTTTAGAAGTAA
DS548835_12593-32400  GTCCAATTGTTGAAGTTAAGTGAGAATTTTTAATATTTAATTTACCTGGAACAGGTGTTTTAGAAGTAA

                      8695
DS571197_51170-71066  GACATTCAGTAAATGGATCTGGACAAAGTGAACATCTCTTTTTATCACCATCTTTTTCTAAATAATAGC
DS548835_12593-32400  GACATTCAGTAAATGGATCTGGACAAAGTGAGCATCTCTTATTTTCTCCATCTTTTTCTAAATAATAGC

                      8764
DS571197_51170-71066  CATTTTCACATTCTGTTCTATTAGCTGTTTTGAAACCATCATACAAATTTGGTGAACATGTTCCTGAAG
DS548835_12593-32400  CTGGTTGGCATTCTGTTTTAGCTGATGTTGTGAAACCATCGTACAAATTTGGTGAACATTTTCCTTCAG

                      8833
DS571197_51170-71066  TTGTTAAGAAATATCCATTATCACATTTTGTACATTTATTTTCTTCTGTTGGTGAGAATAAACGACAAT
DS548835_12593-32400  TTGTTAGGAAATATCCATTATCACATTTTGCACATTTATTTTCTTCAGTCAAAGAGAACAAACGACAAT

                      8902
DS571197_51170-71066  GATTCTTGACACAAACTCCATCAGAAACAGTACATCTTTTATTACATGATGTGCAATGTTCAGCGTTAT
DS548835_12593-32400  GATTCTTAGTACAAAATCCACCAGAAACAGTACATCTTTTATTACATGATGTACAATGTTCAGTATTAT

                      8971
DS571197_51170-71066  AAATACTACATTCGTTACCTTCTGAAAGATTATTTGGATTATCAACAGTACATATAGTACATTTAGAAT
DS548835_12593-32400  AAATACTACATTCATTACCTTCTGAAAGATTGTTTGGATTATCAATTGTACATACATTACATTTAGAGT

                      9040
DS571197_51170-71066  CTGTTTTACTTAAATAATAACCTTCCATACATGTCAAGCATTGATTACCATTAATCTTTGAGCAATGCT
DS548835_12593-32400  CTTT------TAAATAATAGCCTTCCATGCAGGTCGAGCATTGATTACCATTCATTTTAGAACAATGGT

                      9109
DS571197_51170-71066  CCGGGAACTTATTACAAGATTTCTTATCAGTACTGAGAGAATATCCATCTGCACATTGGTTACAAGAGT
DS548835_12593-32400  CTGGATATTTGGTACA---TTTTTTCTCAGCATTAATATAATATCCATCTGCACATTGGTTACAATCAT

                      9178
DS571197_51170-71066  TTTCAACTTGTAAAATACATTTATTAATTTTATCATTCTTTTGAGTACATTTCCCACCTTGAAGAGAGT
DS548835_12593-32400  TTACTACTTGTAAAATACAATTACTAATCTTTTCATTCTTTTTAGTACATTTACCATTTTCAAGTGTAT

                      9247
DS571197_51170-71066  ACATACCGAAACATTTATCACAAGTCTTTGAAGAAGTAGAAGAGGCTAAAAGACCAACACAATTTTCTG
DS548835_12593-32400  AATTTCCAAAACATTTGTCACATGTCTTTGAAGAAGTGGTTGAAGCTAAAAGACCGATACAATTTTCTG

                      9316
DS571197_51170-71066  CATGATCTTCTGTAGTTGCATTATCACATAACTTTGAACCATCAAC---AGTAGTAGACATCCCAATTA
DS548835_12593-32400  CATGGTCATCTGTAGTTGCATTGTCGCATCCTTTTGAACTTCCAGTGTTAGTTGTAGACATTCCAATTA

                      9385
DS571197_51170-71066  TACATTCTCCACATTT---TGAATCTTTCATCAAAAGACATGACTCACAAATGTTTACATTGTTTTTGC
DS548835_12593-32400  TACATTCTTCACATTTAGTGCCACTGTCTTTAGAAAGACAAGTATCACAATTATCAACACCATTTTTAC

                      9454
DS571197_51170-71066  ATACACACTTATCATTTCCAGAGTGTGATGATGTATTTACTTCTTTACCGTTAACACAATAAGTACATT
DS548835_12593-32400  ATGTACATTTTTCAGAATTAGAGTTTGGTGGTGTATTGGCTTCTTTTCCATTTTCACAATAAACACATT

                      9523
DS571197_51170-71066  TGGGGTTTGTAGAGTTTGTTTTATCATAATAAGAAAAAGCAGTTTTACACATATCTTCTTTAAGAGTAC
DS548835_12593-32400  TTGGAGAACTAGCATTACTGTTATCATAGTAAGAAAATGTAGTTTTACAAATGTCCTGTTTAAGAGTAC
```

```
                    9592
DS571197_51170-71066  ATGTCTTATTATTTCCAGT---GGTAGTAAGTTCATAACCAGTATCACAACTAGTGCATGCTCCATTTG
DS548835_12593-32400  ATTTTTGGGTATTTGAAGAATCAGAAGAAAGTTCATAACCAACATCACAACTAGTACATGCCCCATTTG

                    9661
DS571197_51170-71066  AAACTGATGCACAATGTGGAACAGCCTCTCTTGGCTCTTTTCCGCCTATGAGCTTATCAGCAGTATAAT
DS548835_12593-32400  AAACTGATGCACAGTGTGGAACAGCAGTTCTTGGTTCTTGGCCTTTAATAAGTTTATCAGCTTTGTACT

                    9730
DS571197_51170-71066  CACCAAGTGAAATTGATATGAATAAAAGAAGAATAAACATgaataaaataaacagacgaagatctctta
DS548835_12593-32400  CACCAAGTGAAATTGATATGAATAAAAGAATAATAAACATgaataaaataagcagacgaagatctctta

                    9799
DS571197_51170-71066  aaaagggagagggtggctgaagtgaaaaccaaagtcattgcttaatcttttgtaccgttctttacttac
DS548835_12593-32400  aaaagggagagaagagctgaagtgaaaaccaaagtctttgtcttatcttttgtgctgttccttacttac

                    9868
DS571197_51170-71066  gattattttctttcttattgattgaattgaaactaatgctattttaccttttttttttttaaataatttc
DS548835_12593-32400  agttattttctttgttaataaccgaattgaaactaatattattttac---tacttttttgaataacttc

                    9937
DS571197_51170-71066  attatag-ttttttttcttttattaatagtaattaacctaaaagaatcttccttttctcgttgtgttat
DS548835_12593-32400  cttatagtttttttttcattaattaatagtaattaacctaaaagaaccttccttttttctttgtgttat

                    10006
DS571197_51170-71066  tcttattcttgtctttgtgcagaaatgttgtaatttggtttcaatgtattcagaccattcttttgatac
DS548835_12593-32400  tcttattcttgcttttgtaaagaaatgttagaatttagtttcaaggtattcagaccattcttctgacaa

                    10075
DS571197_51170-71066  tt--ttgtgtacttcttatgaaaaaagacagaaattagtacttgctatgcttgcgaaagtcttaataat
DS548835_12593-32400  tttcttgtatatctcttttgaaaaaagacagaaactagcacttgttatacttgagaaa-ttttaataat

                    10144
DS571197_51170-71066  gttttattagaatagaatcaatattttttgaggcatataaaatcgaatagttatccttatttggttcat
DS548835_12593-32400  gttttattagaat-taattagtg-tttttgagtcatatgaattcgaataattctccttatttggtttat

                    10213
DS571197_51170-71066  aactttcaaataaaggaacaaattctgttttcttagattacaagtttataa-------tatatttattt
DS548835_12593-32400  aactttcaaataaaggaatatattttgttttcttagattacaagtttataatatattttatatttattt

                    10282
DS571197_51170-71066  ttattctttttattttataactttgagaactaatttaaattacttagataattagatttattagccttt
DS548835_12593-32400  ttattctttttattttataactttgagaactattttaatttactcagataatt-ggtttattggtcatt

                    10351
DS571197_51170-71066  gttttgcttt-aataatgcttaagtcaatgtaatataatatgttcataaacataatactttaccatgac
DS548835_12593-32400  gttttgccttaaataatacgtaagtcaataggctattatgtgctcat-aacttaatgcccta-tatggt

                    10420
DS571197_51170-71066  ttttgtgttatcttttatttttaaaatgtgttgatttgtataaaattaataaaaaatataataaataaa
DS548835_12593-32400  ctttgtactatctcttc-ttttaaaatgtgttgttttctataaaattaataaaaaataaaataaata--

                    10489
DS571197_51170-71066  atgactcatttaacattgttttatgaatgtattctttgttgataaatgtttttttacagtattgttcta
DS548835_12593-32400  ---actcatttaatatcattttatgc---ttttctttgttgataaatctatttctg-agtcttattcta

                    10558
DS571197_51170-71066  taactattaatataatttgtagaaatgatgttatttagttgttgtttataattaaaatattaattccct
DS548835_12593-32400  caacta-taacatagcttataaaagtg-tattagttgattattatatataacaaaaatactaattcctt

                    10627
DS571197_51170-71066  tgttggtgtttataatgtttaaataaatagaaa-------------------------attttttcata
DS548835_12593-32400  tatt-gtgtttagattgtttaaagaaattggactactataatacgccatagtcttcttaccctttcata

                    10696
DS571197_51170-71066  ttctttcaaagtaatttaatatatcattaacattcatttcaatgaaacattattcaactgaaattgatt
DS548835_12593-32400  ctatttcaaagtaatttaat---------------------actaagcgt--------tgaaattgatt

                    10765
DS571197_51170-71066  ttaacaacaatgaatgttcaagtgtatagagtaattctacaaaatgaatcaattaaaaaaaaattaata
DS548835_12593-32400  ttaaaaacaatgaatgttcaatgaaatagattaattctacaaatttagtcaatt-aaaaaaatttaatg

                    10834
DS571197_51170-71066  taattgctataaacaaattgcaaaagataatgaaaagatttatgaaatttagatttatgttgttatgtg
DS548835_12593-32400  caattgctataaacaagttgctaaaggtagagaaagggcttgtgag------------gttgt------

                    10903
DS571197_51170-71066  agaaagatatatatgtggtaatgcaaagattttgtaagtttatttggtataaacaaagaggagaattgt
DS548835_12593-32400  ---------------------------aattctacaaatttccttattataaataaagaagagaattgt
```

```
                     10972
DS571197_51170-71066  ataaaaataacgttataatactactacttttagtattaggtataaatgttaaagaaaataattttgatt
DS548835_12593-32400  at-aaaacagtattataata--------------------------------------------tcatt

                     11041
DS571197_51170-71066  ggttttcattgaaaaaaaagtgtaataattcaaaagttttgatttatgcagggattagtaatgaataaa
DS548835_12593-32400  ggctcttattaaaaaatgagtgtaacaattcaaagtctctgatttatagagtgttcaacaatgaataaa

                     11110
DS571197_51170-71066  aagaagttgcaattagagaaagttaagtatattgttactcttattctttttta--aaacattctctcta
DS548835_12593-32400  aagaaattccaatcagagaaagttagg--cattatgactttcatcctcttttaacagacatagagaaaa

                     11179
DS571197_51170-71066  tttgttaa-aaatgttttaagtacaaaatcacaattgatttttgctagagttaagttaatttgtatgtt
DS548835_12593-32400  tctgttaataagtattttacctgctaagcctcaattgatttctggtaaagttaagttagttggttta-t

                     11248
DS571197_51170-71066  tttttattttgttttaaatttattcaaagaagaatt-tgatgaatgaaaataaaagaattgacttgtta
DS548835_12593-32400  tttttattttgtcttaagattattcaaagaagaattctgatgggtgataataaaagaatcgatttatta

                     11317
DS571197_51170-71066  aatttaattgtaattcaTTAATATGATTGATATGAGTAATATGAACCAGCTCCAAGAATGCTTTCTTCA
DS548835_12593-32400  aattaaattgtaatttgTTAGTATGACCGATACGAATAATATGAACCAGCTCCAAGAATACTTTCTTCA

                     11386
DS571197_51170-71066  AGAATGCCATATTCCATCATCTCTTTTTGAATCTCTTCTTCTGTTTTTTTTTGTTCAATCCCTTGGATA
DS548835_12593-32400  AGAATACCATATTCCATCATTTCATTTTGAATCTCTTCTTCTGTTTTTTCTTGTTCAATCCCTTTGATA

                     11455
DS571197_51170-71066  ATTTCATCAATGACTTCAGAACTTAATGAGTAACTAAATTCATTGTCACAACTCTGAAGCATTCCAAAA
DS548835_12593-32400  ATTTCATCGATGATTTCAGAACTTAATGAATAACTAAATTCATTATCACAACTCTGAAGCATTCCAATA

                     11524
DS571197_51170-71066  AACTTTCCATTTTCAATTAATCCACCGCCACTCATTCCATCAACAATACTACAATATGTACAATGAATA
DS548835_12593-32400  AACTTTCCATTGTCAATTAATCCACCACCACTCATCCCATCAACAATACTACAATATGTACAATGAATA

                     11593
DS571197_51170-71066  AGTTCTTTTCCTTTGAATGATGTACTACATATTTGCCCCTCTACTTCCTTTAAATTCTCTATATTGACT
DS548835_12593-32400  AGTTCTTTTCCTTTGAGAGATGTACTACATATTTGCCCTTCTACTTCTTTTAAATTATTTATATTGACT

                     11662
DS571197_51170-71066  GGATATCCAATGTGTTTCTTTACACCATTATTTGTTACATTATTTTTTCTTTTATTTTCTTCAATAATT
DS548835_12593-32400  GGATATCCAATATGTTTCTTTGTACTATTATTTGTTAAACTAATCTTTATTTTGTTTTCTGCAATAATT

                     11731
DS571197_51170-71066  TTCATCTTTGCATGAACAATAACTACAACAATATCAAAACACCATGAATAAAACAAAACACTTCCTTGA
DS548835_12593-32400  TTCATCTTTGTATGAGCACTAATCACAATAAGATCAAAACACCATGAATAAAACAAAATCCTTCCTTGA

                     11800
DS571197_51170-71066  ATACTTTGTTGTGTTATTGGGTTGATAATTTCTACTGGGAAATATCTGTTTTCAAATAAATGAGATGAT
DS548835_12593-32400  ATAACTTGTTGTGTTATTGGATTAATAATTTCTACTGGGGAATATCTGTTTTCAAGTAAATGAGATGTT

                     11869
DS571197_51170-71066  GTAACAAAGATTGTTTTTTCTCCCCAATGTTTTATTCCGCAACAACTTCCTTCTTGTTTTCCACTTTTT
DS548835_12593-32400  GTAACAAACATTGTTTTTTCTCCCCAGTGTTTTATTCCACAACAACTTCCTTCTCGATTTCTACTTCTT

                     11938
DS571197_51170-71066  ATTTTATAAATTCCAATTGAATTATCAGTAGTTAACTCATCGAAATTTCCTATTGCGTTCATAACAGTA
DS548835_12593-32400  ATTTTATAAATTCCAGTTGAATTATCAGTAACTAACTCATTGAAATTTCCTATTGCATTTATAACAAGA

                     12007
DS571197_51170-71066  TTAATATTGATAATAGTTGTTGTATTTTTTGCTGTATTTGTAAAAAGAATTAAACCAATAATTTGATTT
DS548835_12593-32400  TTAATATTGATAATTGTTGTTGTATTTTTTCTTGTGTCTGTAAAAAGAATTAAACCAATAACTTGACTT

                     12076
DS571197_51170-71066  TTACAATTCATGACGAAACATCCACTAGAATTTCGAGGTGAGTGTGGTTGAACAATACATCCAATAACT
DS548835_12593-32400  TTACAATTCATAACAAAACATCCACTAGAATTTCGAGGTGAGTGTGGTTGAACAATACATCCAATAAGT

                     12145
DS571197_51170-71066  GAGTTTAAATGGTAATACCTTTCTATGACTTTTGCTTTAATTTCAATGTGTTGTAAAATAGATGACCCA
DS548835_12593-32400  GAATTTAAATGATAATACCTTTCTATGACTTTTGCTTTGATTTCAATGTGCTGTAAAATAGATGGTCCA

                     12214
DS571197_51170-71066  TTAGTAGAATCTCCATATCTTATTATCACTTTATCACTAATCTCATTGCTTACAGTATTTTCATTCCAT
DS548835_12593-32400  TTAGTAGAATCTCCATACCTTATTATCACTTCATCACTAATCTCACTACCTACAGTATTTTCATTCCAT

                     12283
DS571197_51170-71066  TCAATATCACCACACATCTGATATTCAATCATTAAATATTTCTCAAAAAGGTGTTTTTCTTCTTTGTTG
DS548835_12593-32400  TCTATATCATCACACATCTGGTATTCAATCATTAAATACTTCTCAAAAAGTTGTCTTTCTTCTTTATTA
```

```
                 12352
DS571197_51170-71066  ATAATGCCTTCTTCTGTTTTAATAGTAATTATATTAACATCTTTACTTTGAATTTGAATCATTTCACCA
DS548835_12593-32400  ATAATGTCTTCTTCTATTTTAACAGTGATGATATTAACTTCTTTACTTTGAATTTGAATTAGTTCACCA

                 12421
DS571197_51170-71066  TTTCTCTTTATAGTTTTCATTTCAATAACATCATTCTGATGAATATTCCACCCGCATTTATTGGTTAAT
DS548835_12593-32400  TTTCTCTTTATAGTTTTCATTTCAATAACATCATTCTGATGAATATTCCACCCATATTTATTGGTTAAT

                 12490
DS571197_51170-71066  CCAACAATATAATTATCCATTTTTATAATAACTCCATTTGAATTAAATTCGTTATGAACAAAACTTGCT
DS548835_12593-32400  CCAACAATGTAATTATCCATTTTTATGATAATTCCATTTGAATTAAATTCATTATGAACAAAACTTGCT

                 12559
DS571197_51170-71066  TCTTTCCATTTCATtactctctttttaagatatttaatctttctcttaataagttttaattctaaaata
DS548835_12593-32400  TCTTTCCATTTCATtattctcttttt-tggtattaagtctatctcttaacaagttttcattataaaata

                 12628
DS571197_51170-71066  aaacattatcttcactttcttgctaattattttctcactccaaaaatgacaattagacaaaacagaaca
DS548835_12593-32400  gaa------cttcactttcttgttaattattttctttatccaaaaatgacaataagacaaaatagaata

                 12697
DS571197_51170-71066  aaactataactaatgactagtttttttttaacaatttcaacacaaaagaaatacaaatacgtgcattat
DS548835_12593-32400  aagtcataactaatgactagttttttt---acaatttcaacacaaaagaaa-------ataagaaatgc

                 12766
DS571197_51170-71066  caaaagctgttata--ttggtaattttaataaaatccaaaaagtgttgtgtatgagaatatttgattat
DS548835_12593-32400  caaaagcagttatatgtgagtaactatagtaaaatccaaaaagt-ttctgtatgagaacatttgattat

                 12835
DS571197_51170-71066  gtttataattgtTTAGTCATCCCATTCATCACTGTCATCCTCAAAGTCATC---ATCACCGTCATCATC
DS548835_12593-32400  gtttataattgtTTAGTCATCCCATTCATCACTATCGTCTTCAAAATCATCACCATCATCATCGTCATC

                 12904
DS571197_51170-71066  AGCAATATCTTCTCGTCTTTTTTGCATAGCACTTGCAAGTAAACTAGTTAAATCAACTTTCTGTGTTTT
DS548835_12593-32400  AGCAATATCTTCTCGTCTTTTTTGCATAGCACTTGCAAGTAAACTAGTTAGGTCAACTTTCTGGGTTTT

                 12973
DS571197_51170-71066  ATTGAGATTAGCCATTGATGGTCTTGGAGCATTAGTATCAACCTTTTTCAATTGTGTACCTTTTTGAAT
DS548835_12593-32400  ATTGAGATTAGCCATTGATGGTCTTGGAGCATTAGTATCAACTTTTTTCAGTTGTGCACCTTTTTGAAT

                 13042
DS571197_51170-71066  TTGGTCTAACAACGAAGGGGCAGCTGGTGCGGCTGGAGCAGCAGGAACTCCATGACTTCCTCTAGGAGG
DS548835_12593-32400  TTGATCTAATAACGAAGGGGCAGCTGGTGCGGGTGGTGCAGCAGGGGCTCCATGACTTCCTCTAGGAGG

                 13111
DS571197_51170-71066  AGGTGGTGGGGGTGGAAGATCGCTTTGAGTTTCCATTGCAAG---ATGTGGTGGGGGTGGTGGAGCACC
DS548835_12593-32400  ---AGGAGGGGGTGGAAGATCACCTTGAGTTTCCATTGCAAGAGGAGGGGGTGGGGGTGGTGGGGCACC

                 13180
DS571197_51170-71066  ACGAGGTGATACACCACCTCT---AGGAGGAGGGGGTGGTGGGGCACCAACACCAGACTCTTTTCTTCT
DS548835_12593-32400  ACGAGGTGATGCACCACCTCTAGGAGGAGGAGGGGGTGGTGGGGCACCAGCTCCAGAGTCTTTTCTTCT

                 13249
DS571197_51170-71066  TGGAGGAGTAGGTCTTGGGGCACTTCCAGATGGTCTTTGAGGTGCTGATTGAGCAGGACGAGTACCAAG
DS548835_12593-32400  TGGAAGGGCAGGTCTTGGAGCACTTCCAGATGGTCTTTGAGGTGCTGATTGGGCAGGACGAGCACCAAG

                 13318
DS571197_51170-71066  ACCTCTTGAAGATGGTGGTAATGGAGG---AGCAACAAGATCTGATGTGCTCAAAGAAATTTCATGAAT
DS548835_12593-32400  ACCTCTTGAAGATGGTGGTAATGGAGGGGGGACAACAGCATCTGAACTACCCAATGAAATTTCATGAAC

                 13387
DS571197_51170-71066  TTCAGGGGTATTGTCTTTACATTGTAGTTCACCTAAAATTTCTGGGGGAATATTTCCTGCTTCAACTTG
DS548835_12593-32400  TTCAGGGGTATTATCTTTACGTTGTAATTCACCTAAAATTTCTGGTGGAATATTTCCTGCTTCAACTTG

                 13456
DS571197_51170-71066  AGCAGTGATAATTCCAAACAACATTTGTACAGTATCAGGATCAGCTAAATCACGTCTTTTAACACCAGC
DS548835_12593-32400  AGCAGTGATAATTCCAAATAACATTTGGACAGTATCTGGATCAGCTAAATCACGTCTTTTAACACCAGC

                 13525
DS571197_51170-71066  GTCCTTTAAAATTTGTTTTAAATCATCTGACATCCATTTTGTATTGATAAGAATTTGTTTTTTATTCAT
DS548835_12593-32400  ATCTTTTAAAATTTGTTTTAAATCATCTGACATCCACTTTGTATTAATAAGAATTTGTTTTTTATTCAT

                 13594
DS571197_51170-71066  AATTAAGTCTTTAACTCTTCTTGGCCATACGTTTTGAGTCTTAGATAACTTTTTAATTTTAGGAGTATT
DS548835_12593-32400  AATTAAATCTTTAACTCTTCTTGGCCATACGTTTTGAGTCTTTGATAACTTTTTAATTTTAGGAGTATT

                 13663
DS571197_51170-71066  TTTTTCTAAGAACATAGCATTCAAAGAAGAAGAATTTTGTTCATCTTCCAGAAAAACGAACATACCAGT
DS548835_12593-32400  TTTTTCTAAAAACATAGCGTTCAAAGAAGAAGAATTTTGTTCATCTTCCAGAAAAACGAACATACCAGT
```

```
                    13732
DS571197_51170-71066  AATTAATTGGCAATACATCTCACACATTAAATTTGTTGTTTCAGTGAGTCCTTTAACAATTCCTTTTTG
DS548835_12593-32400  AATTAATTGACAATACATTTCACACATTAAATTTGTTGTTTCAGTGAGTCCTTTAACAATTCCTTTTTG

                    13801
DS571197_51170-71066  AACAGTTTTTTCAGCTTCATTAATATTTTCCATCAATGTTTTTAAATTATCACCTTTTTTTAAATTTTT
DS548835_12593-32400  AACAGTTTTTTCTGCTTCATTAATACTTTCCATCAATGCTTTTAAATTATCTCCTTTTTTTAAATTTTT

                    13870
DS571197_51170-71066  AGCTCGTTTTGTAGCTTTTTCTTTTGCATCTTCATTTTTCTTTCTATCATTTTTAACAGTTTCCTTTGT
DS548835_12593-32400  AGCTCGTTTTGTTGCTTTTTCTTTTGTGTCTTCGTTTTTCTTTCTATCGTTTTTAACATTTTCTTTTGT

                    13939
DS571197_51170-71066  GTCTTTAATTTTCTTTGGCATTTCACAAACTTCTCCACGATATATTCTAATAGTGTTATCAACAAAAAT
DS548835_12593-32400  GTCTTTAATTTTCTTTGGCATTTCACTAACTTCTCCACGATAGATTCTAATAGTATTGTCAACAAAAAT

                    14008
DS571197_51170-71066  TTTATTAGATTGAGTTAATGTGTTAGCTTGTGTTTCTAACATTCCAGCCACACTTTGAAGTGCTGTATT
DS548835_12593-32400  TTTATTAGATTGAGTCAATGTATTGGCTTGTGTTTCTAACATTCCTGCCACACTTTGAAGTGCTGTATT

                    14077
DS571197_51170-71066  AATTAATGTACATCCATCCATAGTTACTGACATTCCTTTTAATTGATTAGCAATACCAATAGCACATGA
DS548835_12593-32400  AATTAATGTACACCCATCCATAGTGACTGACATTCCTTTTAACTGATTAGCAATACCAATAGCACATGA

                    14146
DS571197_51170-71066  AGCGAGAGTAGTTTGAGCCTTTGAATAAGCCTCACAACTTTCAATAAGTTGTTTATATGTTGGGAGGAT
DS548835_12593-32400  AGCGAGAGTGGTTTGAGCTTTTGAATAAGCCTCACAACTTTCAATAAGTTGTTTGTATGTTGGAAGAAT

                    14215
DS571197_51170-71066  TGTAGAAAGATGCTTTAATAT--GAGAATTAATTTAAGTAAAAACAGAGACATaatttaaaatgaaaga
DS548835_12593-32400  TGTAGAGAGATGctttaatatgagagaattcatttaagtgaaataagaaata-aatttaaaaagaacaa

                    14284
DS571197_51170-71066  ataataatattgataatctagaaat-agaacaaaggaaaaaaagttgagttgtatatataaataagaaa
DS548835_12593-32400  atagtactattgac--tctagaaataaaaaaaaagaaaaaaaaattgagttgagtacat-aataagaaa

                    14353
DS571197_51170-71066  agaacaaacggtaagcatagtagatgttagttcatccatttctttaagttcagatgatgactttatagt
DS548835_12593-32400  aaaacaaacAGTAAGCATGGTTGATGTTAACTCATCCATTTCTTTAATTTCAGATGATGACTTTATAGT

                    14422
DS571197_51170-71066  aaaatcttcttgagtagaatttactgtaggattattctgattttctgtcatgtaaatgaattttaagat
DS548835_12593-32400  AAAATCTTCTTGAGTAGAATTTACTGGAGGATTATTTTGATTTTGTGTCATgcaaatgaattttaagat

                    14491
DS571197_51170-71066  tttcactaataaatgaactttttgtttcaatagcatttttgttaattccaattttcatcgttttttttc
DS548835_12593-32400  tttcactaagaaatgaactttttgtttcaatagcatttttgttaattccaattttcatctttttttttc

                    14560
DS571197_51170-71066  cttccacaaaaaaataactcca-ttagaacaattgtctttataataattacttttcatttcataataat
DS548835_12593-32400  ctttacaaaaaaaataactccgtttagagcaattgtct---taataattgcttttcatttcataaaaat

                    14629
DS571197_51170-71066  ccccaatctttaaaataatcaatttgagattgaaaaaagaaaatttaataaaatctgaactgataatta
DS548835_12593-32400  ccccaatctttaaaataatcaattcgagatggaaaaaagaaaatttaatataatctgaactgataatta

                    14698
DS571197_51170-71066  aagaaataaatagaaataaaaATGAATGAAGAAAGAAATGATCCTATTCAAGTTGTTAAAATTGAACAA
DS548835_12593-32400  aagaaataaaaagaaagaaaaATGAATGAAGAAAGAAATGATCCAATTCAAGTTGTTAAAATTGAACAA

                    14767
DS571197_51170-71066  CCAAGTGATACCATTACCACATTAAGTTCTTCTACTTGTTCATGTTTATTACAAAACAATATTTTATTA
DS548835_12593-32400  CCAATAGATACTATTACCATATTAAGTTCTTCTACTTGTTCATGTTTATTACAAAATAATATTTTATTA

                    14836
DS571197_51170-71066  TTTGGAAAGAAAGGTATGATGACTGCCATTTCATTAGATAAAAATAAAATGACTGTATTACATCAATAT
DS548835_12593-32400  TTTGGAAAGAAAGGTATGATGACTGCTATTTCATTAGACAAAAATAAAATGACTGTGTTACATCAATAC

                    14905
DS571197_51170-71066  TCATTTAGTGGAACAATAGAACAAATGTGGTATCATCAACAAATGCATTTACTTATTTGTTTAATTAAT
DS548835_12593-32400  TCATTTAGTGGAACAATAGAACAAATGTGGTATCACCAACAAATGCATTTACTTATCTGTTTAATTAAT

                    14974
DS571197_51170-71066  AGTAAACTTGAATCATTTCAAGTTGATTTAAATAAAAAAGCATCAAATGAAGTATTTAAACGTATTACT
DS548835_12593-32400  AGTAAGCTTGAATCATTTCAAGTTGACTTAAATAAAAAAGCATCAAATGAAGTATTTAAACGCATTACT

                    15043
DS571197_51170-71066  ACAAAACAAATTAAAAATATTGGACCATTTGAATGTTTTAGTACATTTGTACGTAAAGGAGTAGTTTTT
DS548835_12593-32400  ACAAAACAAATTAAAAATACTGGACCGTTTGAATGTTTTAGTACATTTGTACGTAAAGGAGTAGTTTTT
```

```
                       15112
DS571197_51170-71066   ATTGCTGCTGTAGATACAAAAAAGAAATCATCAATAACATTAATTGAATCTAAAACTGATGGAATATTT
DS548835_12593-32400   ATTGCTGCTGTAGATACAAAAAAGAAGTCATCAATAACACTGATTGAATCCAAAACCGATGGAATATTT

                       15181
DS571197_51170-71066   TATAAAGAGACTAAAGTTTGGAATGCCGAAGATAAAATTGTTGGTTGTAATTTTGTTAATCAAAACTTA
DS548835_12593-32400   TATAAAGAGACTAAAGTTTGGAATGCAGAAGATAAAATTGTTGGATGTAATTTTGTTAATCAAAATTTA

                       15250
DS571197_51170-71066   GTTCTTGTTTATCCTAACAAAATACAAATTATTTCATTCTTTGGTAAAAGTGAAACTATTTGTAATAAA
DS548835_12593-32400   GTTATTGTTTATTCCAACAAAATACAAATTATTACATTCTTTGGTAAAAGTGAAACTATTTGTAATAAG

                       15319
DS571197_51170-71066   TCTATTATTCCATTTAAAGGTAAACCAAGATTTTTAGAATTACAATTAAATCAAATTCTTGTTCATACA
DS548835_12593-32400   TCTATTAATCCATTTAAAGGTAAACCAAGATTTTTAGAATTACAATTAAATCAAATTCTTGTTCATACA

                       15388
DS571197_51170-71066   GGAAATTGTGTTTATTCAATTGAAGAAGATGCAGTAAATTTAGTTCCTAGTGATTATCCTGTTATTACA
DS548835_12593-32400   GGAAATTGTGTTTATTCAATTGAAGAAGATACAGTAAATTTAGTTCCTAGTGATTATTCTGTTATTACA

                       15457
DS571197_51170-71066   ATAAGTAAAATGTCTCCATTTATTTTCTTTATGCAAGAATTTGCACACAATAAATTTTATATTACTTAT
DS548835_12593-32400   ATAAGTAAAATGTCTCCATTTATTTTCTTTATGCTAGAATTTGCACACAATAAATTTTATATTACTTAT

                       15526
DS571197_51170-71066   AGATTAGAACCAATGACACATGCCTCACCACATTCACTTGATTTTAGAAGACCTGAATATTATAAAGGA
DS548835_12593-32400   AGATTAGAACCAATGACACATACCTCACCACATTCATTAGATTTTAGAAGACCTGAATATTATAAAGGA

                       15595
DS571197_51170-71066   AGTTATATGCATGAAGCTGTCCCAGTATTTATCAGTGGTACAAGTAAACAACTAATTTACTTTACTCCA
DS548835_12593-32400   AGTTATATGCATGAAGCTGTACCAGTATTTATCAGTGGTACAAATAAACAATTAATTTATTTTACTCCA

                       15664
DS571197_51170-71066   AATCCTAAATGGTATGAATCTGCAATTCAAAATGGACATTATCAAAGTGCAATTGAATTTATTACATTA
DS548835_12593-32400   AATCCTAAATGGTATGAATCTGCAATTCAAAATGGACATTACCAAAGTGCAATTGAATTTATTACGTTA

                       15733
DS571197_51170-71066   TTAGGAAATAGTGCAGATGAACATTCAGAACAATTTAAAAAATGTGCTTATACATTAGGAGTAATGAAT
DS548835_12593-32400   TTAGGAAATAATGCAGATGACCATTCAGAACAATTTAAGCGATGTGCTTATACATTAGGAGTAATGAAT

                       15802
DS571197_51170-71066   AAAATTATTGAATTTTATAAAAATGAAGAAAATAGAACAAGTGAAAATATTATAACCATTTTAAATGAT
DS548835_12593-32400   AAAATTATTGAATTTTATAAAAATGAAGAAAACAGAACATGTGAAAATATTATAACTATTTTAAATGAT

                       15871
DS571197_51170-71066   TTTAAAATGGGTGGAATTAGTCCAAAGAATATATTAGTCTTTTTAGAATTATTTTTATTCCATGACTTT
DS548835_12593-32400   TTTAAAATGGGTGGAATTAGTCCAAAGAATATATTAGTCTTTTTAGAATTATTTTTATTCCATGATTTT

                       15940
DS571197_51170-71066   ATTACAGATCTTCATTCATTATTCCAAGATTGGTTTACTGTTTCTATCGAAAATTGGAAGAAAGATATG
DS548835_12593-32400   ATTACAGACCTTCATACATTATTTCAAGATTGGTTTACTGTTTCTATTGAAAATTGGAAGAAAGATATG

                       16009
DS571197_51170-71066   GTTAAAACATTCCCTGGACAACTTGTAAAAAATCTTCAAGATGCTTTAGAAGAAGTTATTAAAGAAAAA
DS548835_12593-32400   GTTAAAACCTTCCCTGGACAACTTGTAAAAAATCTTCAAGATGCTTTAGAAGAAGTTATTAAAGAAAAA

                       16078
DS571197_51170-71066   AATACAGCATTAAAAAATAATAAAACTGTTCCATTACAAAATCATTTTAATTCAGAAGGACATTCTACT
DS548835_12593-32400   AATACTGCATTAAAAAATAGTAAAAGTGTTCCATTACAAAATCGTTTTAATTCAGAAGGACATTCTACA

                       16147
DS571197_51170-71066   AAAGAAATTATTCAAGCCATTGTTACTTATCTTATGGCAGTAAAATCACAAATTGTTAACAAAGAACAA
DS548835_12593-32400   AAAGAAATTATTCAAGCTATTGTTACTTATCTCATGGCAGTAAAATCACAAATCGTTAACAAAGAACAA

                       16216
DS571197_51170-71066   ATTGATGGAGAAACAAGTGAAGAAAGAGAAATATTTTATACTTCATTAGTAGTTATTGTTTATTATCTT
DS548835_12593-32400   ATCGATGGAGAAACAAGTGAAGAAAGAGAAATATTTTATACATCATTAGTAGTTATTGTTTATTATCTC

                       16285
DS571197_51170-71066   TCACCTAATTCAAAGGAAATGCTTGAATTATTACAAAATACTAAATTCCTTTTTAAAACAGTTATTTTA
DS548835_12593-32400   TCACCTAATTCTAAAGAAATGCTTGAATTATTACAAAATACTAAATTCCTTTTTAAAACAGTTATCTTA

                       16354
DS571197_51170-71066   AAAATTCTAGAAGAACATACTACAATTAATAAAAAGTTACTTATTGAATATTATATTTCTCAAAATCAT
DS548835_12593-32400   AAAATTTTAGAAGAGAATACAACAATTAACAAAAAGTTACTTATTGAGTATTATATTTCTCAAAACCAT

                       16423
DS571197_51170-71066   ATTGATGGAGTATTTAACATTAATGGAATTAGTTATTATGATAAAAGAAATGCAGTAATTCGTATATTA
DS548835_12593-32400   ATTGATGGAGTATTTAATATTAGTGGAATTAGTTATTATGATAAAAGAAATGCAGTAATTCGTATATTA
```

```
                  16492
DS571197_51170-71066  CAAAAGAAAGAAACTGGTGATAAAAATAAAGTTTATAAAAAAATTGAAGAACTTATTGAAGAAAAAATA
DS548835_12593-32400  CAAAAGAAAGAAAATGGTGATAGAAATAAAATTTATAAAAAAATTGAAGAACTTATTGAAGAAAAAATA

                  16561
DS571197_51170-71066  GAAAGTAATGGTGGAAGAAAATTTATTGAAGAAAATAATATGAAACTACAAGATGAATTATATTTAATT
DS548835_12593-32400  GAAAATAACGGTGGAAGAAAATTTATTGAAGAAAATAATATGAAATTACAAGACGAATTATATTTAATT

                  16630
DS571197_51170-71066  TTTGGAAAAGAAAAATCAGTATTAGGTATTGATGAAATTTGTACTATTATTGAAGGATGGGAGTTTGGT
DS548835_12593-32400  TTTGGGAAAGAAAAATCAGTATTAGATCTTGATGAAATTTGTAATATTATTGAAGGGTGGGAATTTGGT

                  16699
DS571197_51170-71066  ATTAAAGATGAAAATGAAGTAAATGAATTTAGAAATAGATTAAGTTGTTTGTATTGTTTAAAATGTTAT
DS548835_12593-32400  ATTAAAGAAGAAAATGAAATAAATGAATTTAGAAGTAGATTAAGTTGTTTATATTGTTTAAAATGTTAT

                  16768
DS571197_51170-71066  AAAGAACAAGGAGAAACAGAAGAACTTGGTAAAGAAGTTATTGAGAAATATATTATTCATATTAAATAT
DS548835_12593-32400  AAAGAACAAGGAGAAACAGAAGAACTTGGTAAAGAAGTTATTGGTAAGTATATTATTCATATTGGATAT

                  16837
DS571197_51170-71066  GCTTCAATAAATAATATTATATTAACTGATGAATATATTAAATCATTTATTGAAATATCAAACAAATAT
DS548835_12593-32400  GCCTCAATAAATAATATCATATTAACTGAAGAATATATTAAATCGTTTATTGAAATATCAAATAAATAT

                  16906
DS571197_51170-71066  CCTTCTATTGTAGAAAAAATTAATTATTCAATAATAAGTCCATTGTATTATAAAATTAGAGCTTGTATT
DS548835_12593-32400  CCTTCTGTTGTAGAAAAAATTAATTATTCAATAATTAGTCCATTGTATTATAAAATTAGAGCTTGTATT

                  16975
DS571197_51170-71066  GGTCTTATTCAACACCAAGAATTAATGCCTATGTTATTACAAGTTTGTAAAGAATTAAAAGAACAAGAA
DS548835_12593-32400  GGTCTTGTTCAACACCAAGAATTAATGCCAATGTTATTACAAATTTGTAAAGAATTAAGAGGACAAGAA

                  17044
DS571197_51170-71066  GAAGTTTTAGATGATATTTATAATGATTCATTTAATCAATTAGTCAAATTCATGTTAAAATTATTTAAT
DS548835_12593-32400  GAAATTTTAGATAATATTTATAATGATTCGTTTAATCAATTAGTCAAATTCATGTTAAATTTATTTAAT

                  17113
DS571197_51170-71066  CAAAAAAAAATTCCACAAAACGTAACTAAAGAATCATTAATGACATTATTATACTCTCCTTCTATTCAA
DS548835_12593-32400  CAAAAAAAAATTACACATAGTATAACTAATGAATCATTAATGACATTGTTATATTCTCCTTCTATTCAA

                  17182
DS571197_51170-71066  AAAATTCTTAAAGAAGAATATATTCCTTTGTCTCTTGTTAATAATTTGTTTGCTGAAATTTCTCTTATT
DS548835_12593-32400  AAGGTTCTTAAAGAAGAATACATTCCTTTGTCTCGTGTTAATAATTTGTTTGCTGAAATTTCACTTATT

                  17251
DS571197_51170-71066  ATCTTTACATCAACAAATCCTATTGAAGTTTATCATCATATACATCAACAGATTGTTAAAGATCATCTT
DS548835_12593-32400  ATCTTTACATCAACAAATCCTATTGAAGTTTATCATCAAATCCATCAACAAATTGTTGAAGATCATCTT

                  17320
DS571197_51170-71066  CCAGGTACAGTTATTAGATTTATATGGGATCTTGCTGATCCAATGGCATTACCTATTAAAGGAGGAGAT
DS548835_12593-32400  CCAGGTACAGTTATTAGATTTATATGGGATCTTGCTGATCCAACAACACTACCTATTAAAGGAGGAGAT

                  17389
DS571197_51170-71066  GAAGAAATTAAAGAAGAATTAAAAATATTTGAAGGTGATGAAGATAGACAAAGTATTTTAGAAGGAATT
DS548835_12593-32400  GAAGAAATTAAAGAAGAACTAAAAATATTTGAAGGTGATGAAGATAGACAAAGTATTTTAGAAGGAATT

                  17458
DS571197_51170-71066  ATTGGATATTTAGAAAAGAAAAAGACTAAAAAACCAGAAGGAATGTTATTAACTGACTGTTATCTTGAA
DS548835_12593-32400  ATTGGATATTTAGAAAAGAAAAAGACTAAAAAACCAGAAGGAATGTTATTAACTGATTGTTATCTTGAA

                  17527
DS571197_51170-71066  TATTTAAAAATTGCCACTAGTGATAGATTAAATGCTACATCTAAATTAAATGACCAAATGCAAAACACT
DS548835_12593-32400  TACTTAAAAATTGCTACTAGTGATAGATTAAATGCTACATCTAAATTAAATGATCAAATGCAAAATACT

                  17596
DS571197_51170-71066  CCAGTATTACAAAAACATATTGAAAAAGATTATGATAAATTACCTTGGATATTACGTTGTAGAATTCAA
DS548835_12593-32400  CCGTTATTACAAAAACATATTGAAAAAGATTATGATAAATTACCTTGGATATTACGTTGTAGAATTCAA

                  17665
DS571197_51170-71066  CACACTAAATGTAAATATAAAGGATTATTAGAATATTTTAAACAAATGAAAAATCAATATTCATCAACC
DS548835_12593-32400  CATACTAAATGTAAATATAAAGGATTATTAGAATATTTTAAACAAATGGAAAATCAGTATTCACCTACA

                  17734
DS571197_51170-71066  TTTGCTGAAGATTCTTTTTGTGAATTAGATAATTTTATAAGAAGATTTGATCGTACATTTAATTTCGAT
DS548835_12593-32400  TTTGCTGAAGATTCTTTTTGTGAATTAGATAATTTTATTAGAAGATTTGATTGTACATTTAATTTCGAT

                  17803
DS571197_51170-71066  GGAACTTCAGGAAGTATTGCTAAAATGGTTAGATTAATAAGACAAAATTATCGTCCATTCAATAAAGTT
DS548835_12593-32400  GGAACTTCAGGAAGTATTGCTAAAATGGTTAGATTAATTAGACAAAATTATCGTCCATTCAATAAAGTT
```

```
                   17872
DS571197_51170-71066  CATCCAAATCATATTGGAGCTGAAACAATCATGTATTGTGTTTGTCAAATGAATATCAGTACAAATACT
DS548835_12593-32400  CATCCAAATCATATTGGAGCTGAAACAATTATGTATTGTGTTTGTCAAATGAATATCAGTACAAATACT

                   17941
DS571197_51170-71066  TCTGCTTTATTATCTTTTTTGGTTCATTTATTATTGGAGAAAGATAATGACTCATTTGATAACCCATTT
DS548835_12593-32400  TCTGCTTTATTATCTTTTTTGGTTCATTTATTATTAGAGAAAGACAATAAATCATTTAATAATCCATTT

                   18010
DS571197_51170-71066  CCAATTTCATTTGCATTAGAGTTATATAGTCGTTTCTATCCTATTATTCCTTTAAAATTTACTGAAGGT
DS548835_12593-32400  CCAATTTCATTTGCATTAGAATTATATGGTCGATTCTATCCTATTATTCCTTTAAAATTTACTGAAGGT

                   18079
DS571197_51170-71066  GAATTTAAATGGCTTGGAGTTAAATTTGATACTTTTGAATTAAATATTGATATTGAAACTTGTTTATTC
DS548835_12593-32400  GAATTTAAGTGGCTTGGTGTTAAATTTGATACATTTGATTTAAATATTAATATCGAAACTTGTTTATTC

                   18148
DS571197_51170-71066  AACAAGATGCTTGATCGTGTAGAGAGTTTTGATGATTTACAAATTTTACTTCCCGCCATTTATTCTTAT
DS548835_12593-32400  AACAAGATGCTTGATCGTGTAGAAAGTTTTGATGATTTACAAATTTTACTTCCTGCCATTTATTCTTAT

                   18217
DS571197_51170-71066  GCCAGAAAGTATTTCTACCTTTCTATTTATTCAAATGCTATTAAAGGATTAACTATAAATTCTAATAAA
DS548835_12593-32400  GCTCGAAAATATTTCTACCTTTCTATTTATTCAAATGCTATTAAAGGATTAACCATAAATTCTAATAAA

                   18286
DS571197_51170-71066  CGTTATGAAGTTATTGATCGTAAGACAGTTTGTTACAAATGTCATGATCAAATATCAGCTACAAGTGCT
DS548835_12593-32400  CGTTATGAAATTATTGATCGCAAAACAGTTTGTTACAAATGTCATGACCAAATATCAGCTACAAGTGCT

                   18355
DS571197_51170-71066  TTTGTAGTGAATAATGGTCAATACGCTCATGAAGACTGCAATGAATTAAACCTTGAAAACCCAATTAAT
DS548835_12593-32400  TTTGTAGTGAACAATGGCCAATATGCTCATGAAGATTGCAATGAATTAAACCTTGAAAACCCAATTAAT

                   18424
DS571197_51170-71066  TGActtttaaacaacactattaaaatttgaaat-ataaaaagtaaaaaaaaaatagaaaTTAATTGGAG
DS548835_12593-32400  TGAtttttaaacaaca-tattaaaatttgaaataataaaaatt----aaaaaatagaaaTTAATTGGAG

                   18493
DS571197_51170-71066  AAGAGTGAAACATATGCAATTGCAGCAACACAAACTAATACTGTCATTGTTATACCAACTAATTTTAAG
DS548835_12593-32400  AAGATTGAAACATATGCAATTGCTGCAACACAAATTACTATTGTCATAGTTATTCCAACTAACTTTAAA

                   18562
DS571197_51170-71066  ACACTCATTGGATTAACAATTGATGAACATTCTCTTAAAAATCTTGAAAATTCAGGATTGTTAGAATAA
DS548835_12593-32400  ACACTCATTGGATTAACAATTGATGAACATTCTCTTAAAAATTTTGAAAGTTTAGGATTGTTAGAGTAA

                   18631
DS571197_51170-71066  GTTTCAATCATCCTTGAACAGAATAACTTTAATTCATCAGTTTGATATGTTTTCACTATTTGAAGTAAT
DS548835_12593-32400  GTTTCAATCATCCTTGAACAGAATAATTTTAATTCATCGGTTTGATATGTTTTCACTATTTGAAGTAAT

                   18700
DS571197_51170-71066  TGCTCTGAATGTTTAAGGTTATTTAAAACATAATCATCCCATGACCTAAACGCCAATGGATCAATAACA
DS548835_12593-32400  TGCTCTGAGTGTTTAAGGTTATTTAAAACATAATCATCCCATGACCTAAATGCCAGTGGATCAATAACA

                   18769
DS571197_51170-71066  AGCATTTGTGCAGCTAAATCCACTGATTCAGATAATCCAAATTCAAAGATTTTAGATGGAACTTTTCCT
DS548835_12593-32400  AGCATTTGTGCAGCTAAGTCAACTGATTCAGATAACCCAAATTCAAAGATTTTAGGTGGGACTTTTCCT

                   18838
DS571197_51170-71066  TTTAATAAACTAGGAACTTCAATTAATTCTGCATAATATTCTTTTAATGGAGTTGAATCTAATTGAAGA
DS548835_12593-32400  TTTAATAAATTAGGGACTTCAACTAATTCTACATAATATTCTTTTAATGGAGTTGAATCAATTTGAAGA

                   18907
DS571197_51170-71066  GCATATGCTGCTTGGAATGCTTTTAAAGAAACAGCCTGAGGTAATTTTGTTTTCTTAATAGCACTTAAT
DS548835_12593-32400  GTATATGCTGCTTGGAATGCTTTTAATGAAACAGCTTGAGGCGATTTAATTTTCTTAATAGCACTTAAT

                   18976
DS571197_51170-71066  AATGTCTCAATAATTTGTTTATGAAGAGTAAGATCAAATTTAGAAAGAGATTGTTGATGAACTAAAACT
DS548835_12593-32400  AATTTCTCAATAATTTGTTTATGAAGAGTAAGATCAAACTTAGAAAGAGATTGTTGATGAACTAAAACT

                   19045
DS571197_51170-71066  AAAGAATATACTTTGTAAACTTCATGTTGAATGAATAGATAAATCAAATGAATATCAATTTCTTCTTCA
DS548835_12593-32400  AAAGAATATACTTTGTAAACTTCATGTTGAATGAATAGATAAATCAAATGAATATCAATTTCTTCTTCA

                   19114
DS571197_51170-71066  GTAAATTGTAGTGTTTCAGTTGATAATGATAATGATAATGCTTCAATATAGAATAATTTTCCAAGAGAA
DS548835_12593-32400  GTAAATTGTACTGTTTCAGT------TGATAATGACAATGCTTCAATATAAAATAATTTTCCAAGAGAA

                   19183
DS571197_51170-71066  AATGATGAATCATTTTTATATTGTTTTAAAAAATTCTTTCTTTTAGATATACTAATTTGATTTAATATT
DS548835_12593-32400  AATGATGAATCATTTTTATATTGTTTTAAATAATTCTTTCTTTTAGATGCACTAATTTGATTTAATATT
```

```
                      19252
DS571197_51170-71066  TGATTAAGATCTTTATTATCGGCAGCACTCAAATGATTGAATGGATAATAATCCATATTAGCTTGTTCG
DS548835_12593-32400  TGATTAAGATCTTTATTATCAGCAGCACTTAAATGATTGAATGGATAATAATCCATATTAACTTGTTCA

                      19321
DS571197_51170-71066  TTCCATTGTTCAAAGTATTGCTTAAGCATTTCTAATTTCTCTTCTTCAGTGATAATATTCTTTTTTCTA
DS548835_12593-32400  TTCCATTGTTCAAAATATTGTTTAAGCATTTCTAATTTTTCTTCTTCATTGATAATATTCTTTTTTCTA

                      19390
DS571197_51170-71066  ATTTCTTTTGGTAATCCTTTTAAATTAATTTTACCATTTCTTTCTTTAATTTCTAACTCTACTACTTTA
DS548835_12593-32400  ATTTCTTTTGGTAGTCCCTTTAAATTAATTTTACCATTTCTTTCTTTAATTTCTAACTCTACTGGCTTA

                      19459
DS571197_51170-71066  TTTTTCTTTACTTTTTTTTCTTTAACTTCATTCTTTGTATCAAATTCTTCTAACGCATTTGATTCATGT
DS548835_12593-32400  TTTTTCTTTACTTTTTTTTCTTTAACTTCATTCTCTGTATCAAATTCTTCTAAAGCATTTGATTCATGT

                      19528
DS571197_51170-71066  ATTTCTTCTTTAGTCACAGTAATAACTTCTTCTTCAAT---TTCTAATTGAATGTTCTCAGAAGAAGGA
DS548835_12593-32400  ATTTCTTCTTTAACCACTGTACTCACTTCTTCTTCAATAACTTCCAATTGAACTTTCTCAGAAGAAGGT

                      19597
DS571197_51170-71066  TCAACAGGAATATGTTCAACTTTCTGATTAAGAATTTCTTCTTCTTTTCTTAACTTTTCTGCTTTCTTT
DS548835_12593-32400  TCAATAAGAGTTTGTTCAACTTTCTGATTAAGAGCTTCTTCCTCTTTTCTTAACTTTTCTGCCTTCTTT

                      19666
DS571197_51170-71066  TCATTACGTTCATTTCGTTTTTGTTCTTTACTTTTCTTAGTAGTTTTATTACTTTTGACTTTTTTTTCT
DS548835_12593-32400  TCATTACGTTCATTTCGCTTTTGTTCTTTACTTTTCTTTGTAGTTTTATTGTTTTTCATTTTTTTTTCT

                      19735
DS571197_51170-71066  GTTTCCTCAACTTTTTCTATATCCTCATTCTTTTCCTCTTTAATCTCTTTCTTCTCATTTCTGTCTGGT
DS548835_12593-32400  GTTTCCTCAATCTTTTCTGTGTCTTCATTCTTTTCTTCTTTGATCTCTTTTTTCTCATTTCGATCTGGT

                      19804
DS571197_51170-71066  TTGATCCCAAGTAATTTTTCTTCTCTTCTTCTTTGTTTCTCTTCTTCTTTTTTACGTTGTTCTTCTTCC
DS548835_12593-32400  TTGATTCCAAGTAATTTTTCTTCTCTTCTTCTTTGTTTCTCTTCTTCTTTTTTGCGTTGTTCTTCTTCT

                      19873
DS571197_51170-71066  TTTGCTTTTTCTAATTTTTCTTTTTCTTTCTTAGACAATTTCTTTACTCCTTTTTCTCTTCCTCCTCGT
DS548835_12593-32400  TTTGCTTTTTCTATTTTCTCTTTTTCTTTCTTGGACAATTTCTTTGCTCCTTTTTCTCTTCCTCCCCGT

                      19942
DS571197_51170-71066  TGCATctacaaatctaaac--------ttctttttttaagttctttaagtttctgttttatattacat-ta
DS548835_12593-32400  TGCATctaaaaatctaaacttattttttctttttttaagtttttttagtttctaatttatatttcatcca

                      20011
DS571197_51170-71066  attaaaaagccatagtttttggtatttgtcagtagtttcaatta
DS548835_12593-32400  agtaaaaaaccatagattttggtatttatctctagt---------
```
