# Supplementary material for: Evidence of Gene Conversion in Genes Encoding the Gal/GalNac Lectin Complex of Entamoeba
Source: PLoS Negl Trop Dis. 2011 Jun 28;5(6):e1209. doi: 10.1371/journal.pntd.0001209 (PMC3125142; doi:10.1371/journal.pntd.0001209)
Supplement: Figure S5 — Nucleotide alignment of orthologous genome regions of E. histolytica and E. dispar used to estimate inter-specific divergence around the intermediate chain lectin orthologues EHI_065330 and EDI_244250. (PDF) [file pntd.0001209.s005.pdf]

```
Alignment: /Users/garethweedall/Desktop/igl2.fas
Seaview [blocks=70 fontsize=10 A4] on Fri Nov  5 15:25:23 2010

                            1
DS571302_24704-36084        gaatgtgttgatttaatggga--tgtttgtgtgtttgaaatgtcacattcaaattggtttgaatgtt
DS548521_2946-13388_rc      aaaaaataacacttagaaagaactctttctttcaacaagagttctaattgagagcaaaaacaataac

                           68
DS571302_24704-36084        ccagaataacatcgtaaactaaaaagatgttattgTTAGTTAAAAGAAGAATGAAAAGAAGAAAGAG
DS548521_2946-13388_rc      ct--aacaaaagcataaaataaataggtttttacttTCATTTTAAGAAAGG-----------------

                          135
DS571302_24704-36084        AATAGAAAGAATGAGTGAAATTATCAAGAAATGATTTAATTTGAACAGAAGATGCATGAGGATTTGA
DS548521_2946-13388_rc      ----GAAAGAAAGAATGAAATTATCGAGAAAAGATTTAATTTGAAGAGAAGAGGCGTTTGGATTTGA

                          202
DS571302_24704-36084        ATTGAACCACGAGTTTATGATTTGAATTTCATTAGATTTACCTTGTTTTTTAAGGATATGAAGAAGA
DS548521_2946-13388_rc      AGAGAACCAAGAGTTCAAGAGAAAGATTTCATTAAATTTACCGAGTTTTTTTAATTGATGAAGAAGA

                          269
DS571302_24704-36084        CGATTTTTTTCAGAGTGAAGACTTTTGATTTTCTTCAATTCAACATCTTGAATATCTAACAAATTGG
DS548521_2946-13388_rc      CGGTTTTTTTCTGCGAGTAAAGATTTAATTTTTTTGAATTCGATGTCTTGAATGTCTAATAAATTAG

                          336
DS571302_24704-36084        ATCGTTGAATAAGTGTTGGAAGATTTGCTTGCTTGTCTTTTTGTTTCTTGTTACTCACTTCAATTTC
DS548521_2946-13388_rc      ATCGTTGAATTAATGATTCAAGGTTTTGTTGTTTTTCTTTTTGATTTTTGTTAGTTACTTTAATTTC

                          403
DS571302_24704-36084        TTCTTGAAGCTTTAATTCTTTAGGTTCTATAGTTCGTGCAATGACATGAATCGTTCCATCTAATGTA
DS548521_2946-13388_rc      TTCTTTAAGGAGTAAATTTTCTGGTTCTATAGTTCGTGCAATGATATGAATTGTTCCATCTAATGTG

                          470
DS571302_24704-36084        ATTTCACATTCAACTTCAAATCGAACTGCTCCTGCTGGAAGTTCAGGAAGTCCACTGATAGTAAATG
DS548521_2946-13388_rc      ATTTCACATTCAACTTCAAATCGAACTGCTCCTGCTGGAAGTTCTGGAAGTCCAGTTATTGTGAATG

                          537
DS571302_24704-36084        TTTCTAAATAACTTAATTCTTCTTCTGTATTTCCTTCTCCTTCAAAAATTGGAAAACATGCCGTTGT
DS548521_2946-13388_rc      TTTCTAAATAACTTAATTCTTCTTCTTTATTTCCTTCTCCTTCAAAAATAGGAAAACATGCTGTTGT

                          604
DS571302_24704-36084        TGAATATCCATTTGATGTTTTAAATTCTTGAACATGTTTTACTGGTAATCTTGTAAATCTTTTAATT
DS548521_2946-13388_rc      TGAATATTCTGTTGATGTTTTAAATTCTTGAATATGTTTAGTTGGTAGTCTTGTAAATCTTTTAATT

                          671
DS571302_24704-36084        AATGTACTAAATCCTTGTTTTGTTTTTAATTGGATTGAATGAGATGTAATATCATTATATGTTGGAA
DS548521_2946-13388_rc      AATGTACTAAATCCTTGTTTTGTTTTTAGTTGAATTGAATGTGATGTAATATCAATATATGTTGGAA

                          738
DS571302_24704-36084        TGTTTAT------------------------------------------------------------
DS548521_2946-13388_rc      TATTTAATTCTTCTTTTTTTCTTTTAATAATATCTTCTTCTTCTTCTTCTTCAATATCTTTTTTTCC

                          805
DS571302_24704-36084        -------------------------------------------------------------------
DS548521_2946-13388_rc      TTTTTTTATTATATCTTCTGTTAATTCTTCTTTTGGTTTAATTAAATCTTCTTTTGGTTTAATTAAA

                          872
DS571302_24704-36084        -------------------------------------------------------------------
DS548521_2946-13388_rc      TCTTCTTTTGGTTTAATTAAATCTTCTTTTGTTTGGATGAAATTATGTATTAAATCTTCATTAAAAT

                          939
DS571302_24704-36084        ----------------------ATCCTTTTCTTCTTTTTTTGAATTTATTTCTTCTTTCTTTGAATT
DS548521_2946-13388_rc      GATCTTTATTAAATTTATTTTT---------------------------------------------

                         1006
DS571302_24704-36084        TATTCCTTTTACTAATTCTTCATTATTTGTATCTGTTTGTTTATTTATTCCTTCTTTAAAAAGTTCA
DS548521_2946-13388_rc      ---------------------------------------TTTAATTAATCCTTCTTTAAAAAGATCG

                         1073
DS571302_24704-36084        TATCCAACTAATGCTGCTCCTCTTGCAACTGTTTGGTCTGGATCAATTTCTCGGTTTGGAATAAGAT
DS548521_2946-13388_rc      AATCCAACTAATGCAGCACCTCTTGCAACTGTTTGATCTGGATCAATTTCTCTATTTGGAATAAGAT

                         1140
DS571302_24704-36084        CAAAATATTCACTTACCATCTCTCGAATTTTAGGAATTTTACTTGTCCCACCAACAAGAATAACTTC
DS548521_2946-13388_rc      CAAAGTATTCACTAACCATTTCTCGAATTTTAGGGATTTTACTTGTTCCACCTACAAGAATAACTTC

                         1207
DS571302_24704-36084        TTCTACACTTTCTTTTCTTATACCTTTCTTTTGCATTGTTTTATCTTCAAACATACGTTCAATACAT
DS548521_2946-13388_rc      TTCAACACTTTCTTTTGTTATTCCTTTATTTTTTAATTCTATATCTTGAAACATACGTTCAATACAT
```

```
                               1274
DS571302_24704-36084     TTAATACATTTCTCAAAAATTTCTTTACTTGCTTCTTCAACTTCTTGTCTACTAATTTCAATATTTA
DS548521_2946-13388_rc   TTAATACATTTTTCAAAAATATCTTTACTTGCTTCTTCAATTTCTTTTCTAGTAATTTCAATATTTG

                               1341
DS571302_24704-36084     TTATTTCTTCTTC------TTCTTCATAAAATTCACTCAAATCAAATGTACATCGTTGATTAGTACT
DS548521_2946-13388_rc   GAATATCTTCTTCATTATCTTCTTCATAAAATTCACTTAAATCTACATTACATCTTTGATTTGTACT

                               1408
DS571302_24704-36084     TAAAATAATTTTAATTTCTTCACTTATCTTTTCAATTCGATATTTCTTTCTTTTATAACTCTCTTCT
DS548521_2946-13388_rc   TAAAATTATTTTAATTTCTTCACTTATCATTTCAATTTTATATTTATTTCTTTTATAACTTTCTTCT

                               1475
DS571302_24704-36084     GCTTTTTCTTTATCAATCTCTTTCCATTTCTTCATTAATATTTCTTCTACTTTTCTATCTATATCTT
DS548521_2946-13388_rc   GCTTTTTCTTTATCTATTTCTTTCCATTTTTTCATTACAATTTCTCCTACTTTTCTATCAATATCTT

                               1542
DS571302_24704-36084     TTCCTCCTAAATGTCTTTCTCCTTCAGTTGCTATTACTTGTTGATTTCTTTTATTCATTCTAATTAA
DS548521_2946-13388_rc   TTCCTCCTAAATGTGTTTCTCCTTCTGTTGCTATTACTCTTTGATTTCTTTTATTTGTTCTAATTAA

                               1609
DS571302_24704-36084     TGTTATATCAAATGTTCCTCCTCCAATATCAAATACAAGTATTGTTTTCCCTTCACAATATTTTTTA
DS548521_2946-13388_rc   TGTTATATCAAATGTTCCTCCTCCAATATCAAATACAAGTATTGTTTTCCCTTCTTCATATTTTTTA

                               1676
DS571302_24704-36084     TCATATCCATATGCAATTGCTGCCGCTGTTGGTTCATTTATTATTCCTATAACTTCTAATCCTGCTA
DS548521_2946-13388_rc   TCATATCCATATGCAATTGCTGCTGCTGTTGGTTCATTTATTATTCCTATAATTTCTAATCCGGCTA

                               1743
DS571302_24704-36084     TTTCTCCTGCATATATCGTTGCTTTTCTTTCTTCATCTTTAAAATGTGCTGGTACTGCAATTACTGC
DS548521_2946-13388_rc   TTTCTCCTGCATATATCGTTGCTTTTCTTTCTTCATCTTTAAAATGTGCTGGTACTGCTATCACTGC

                               1810
DS571302_24704-36084     TTTACGAGGTGCTATTTTATATTGTTCTTTAGCTTCTTCTATTAATTTCCGTATTATCATATTCCCT
DS548521_2946-13388_rc   TTTCTTTGGTGCTATTTTATATTCTTCTTTCGCTTCTTCTATTAATTTTCTTATTATCATATTCCCT

                               1877
DS571302_24704-36084     ATTTCTTCCCCTGAATACCATTCCTCTTCACTTACTCCTATCTCTACTTCCCCATTCCCTGCATTCC
DS548521_2946-13388_rc   ATTTCTTCTGCTGAATACCATTCTTCTTCATTCACTCCTATTTCGACTTCTCCATTCTTTTCATTTC

                               1944
DS571302_24704-36084     GTCGTATTCGATACTCTAATTTCTCCCCTTCTTTCTCTATCATTTCATCATATTTCTTCCCTATCAA
DS548521_2946-13388_rc   TTCGAATTTTATATGTTAATTTCTTTCCTTCTTTCTCAATTTCTGAATCATATTTCTTTCCTATAAA

                               2011
DS571302_24704-36084     TCGTTTCGTATCATATATTATCACCCCTTGTGAACTTCTTCTCGCTTCTTCCCCTATTATTCTCCCT
DS548521_2946-13388_rc   TCTCTTCGTTTCATATATTATCACCCCTTGTGAACTTCTTCTCGCTTCTTCCCCTATTATTCTTCCT

                               2078
DS571302_24704-36084     TCTTTTGTAAATCTTACTATCGACTCAATCTTTTCTTTCCCTTCTTTACTCTTTATCACTTCAACCC
DS548521_2946-13388_rc   TCTTTTGTAAATCTTACTATAGACTCTATCCTTTCTTTCCCTTCTTTGTTTTTTATCACTTCAACCC

                               2145
DS571302_24704-36084     TTTGATGATGTTCATCATATCTTGCCATACATGAATTTGTCGTTCCTAAATCTATTCCTACTACATA
DS548521_2946-13388_rc   TTTGGTGGTGCTCATCATATCGTGCTATACTTGATTTCGTTGTCCCTAAATCTATCCCTACTACATA

                               2212
DS571302_24704-36084     ---CTCTTCATTCATttttttgctttttatctttttcttttgtttttttggtttttctttagatttgaa
DS548521_2946-13388_rc   CTCCTCTTCATTCATgttt----------tctt--ctttcttttctcgtttttt-tt----------

                               2279
DS571302_24704-36084     gttggtatatcagaaacaccacacaaaccctaaaataaatcaaatcattacgtcttcagtaattatc
DS548521_2946-13388_rc   -------------------------------------------------------------------

                               2346
DS571302_24704-36084     tggttatgacgttgcttttggatagagaatatattaccaattctgcaaagagtgaagtaagatatta
DS548521_2946-13388_rc   -------------------------------------------------------------------

                               2413
DS571302_24704-36084     tgactgtaatgttgttcacttattataatatatcagaaagaatatataatataaaagataaaataca
DS548521_2946-13388_rc   -------------------------------------------------------------------

                               2480
DS571302_24704-36084     agaatcataacataagaaataaataaaaatcaagagatattacaaacatgaaaaagaatgaacgtaa
DS548521_2946-13388_rc   -------------------------------------------------------------------

                               2547
DS571302_24704-36084     tgataaaattattttaaaatgaataaatactaaatgtaattcatatattcaaaattagttacaagaa
DS548521_2946-13388_rc   -------------------------------------------------------------------
```

```
                            2614
DS571302_24704-36084        atgtattaataatttaatctataaaagttgaatacacgtaatgaaaatccaatataattgaaacatt
DS548521_2946-13388_rc      -------------------------------------------------------------------

                            2681
DS571302_24704-36084        aataaatcatacaacatttagatcatatatccctcatatacataataatgaaaaagatacaaagtta
DS548521_2946-13388_rc      -------------------------------------------------------------------

                            2748
DS571302_24704-36084        ttgagatacatattgaagtgaagaaataattaaataccataaccagtaaatgtcataaagaaataga
DS548521_2946-13388_rc      -------------------------------------------------------------------

                            2815
DS571302_24704-36084        aaatataaatatacaatatccccttcattgaataatatcgacaggttgttcaataacatttcattat
DS548521_2946-13388_rc      -------------------------------------------------------------------

                            2882
DS571302_24704-36084        aacaaggaattattgttatgaaataatatatcttcaaaacaataaaatgaaaatatctcaaccataa
DS548521_2946-13388_rc      -------------------------------------------------------------------

                            2949
DS571302_24704-36084        atttgaatgaattaaataatacaaaaattataaaaaaattgatgaatataaagataagatagtagaa
DS548521_2946-13388_rc      -------------------------------------------------------------------

                            3016
DS571302_24704-36084        agtattacagttttgataaataatgcatgaattattcttaaattaatgatagaaatctaaagaaata
DS548521_2946-13388_rc      -------------------------------------------------------------------

                            3083
DS571302_24704-36084        gaaaacgaaattaagacaaaaacaatacaaaataaatgagagagaataaacattaattataatgatg
DS548521_2946-13388_rc      -------------------------------------------------------------------

                            3150
DS571302_24704-36084        gaaattttgatgatgtaaactcataacaaaatatattaaaaaaatactcaaataattgagtgagaga
DS548521_2946-13388_rc      -------------------------------------------------------------------

                            3217
DS571302_24704-36084        tgagggttttaacaataattatatttcaaacaataaaaattaaatatatacactaatcaatatttta
DS548521_2946-13388_rc      -------------------------------------------------------------------

                            3284
DS571302_24704-36084        gattcttttgttatgtttatttttcttttacatttaggttattgtacttttcttttagacattattt
DS548521_2946-13388_rc      -------------------ttcttattttacattaagtttattattattttctttaaaatattattt

                            3351
DS571302_24704-36084        taaagaattgtttattttgttttctttttttatcttttttctacattca-tttttcatgatttgttac
DS548521_2946-13388_rc      tgaagaattacc-------tttttgtttttaacttttttgt--gttcatttttttataattttttaa

                            3418
DS571302_24704-36084        tgttagtaaacttttcattatctatgtcaattgttacgtagttcttgagagtaaaaactattaaaaa
DS548521_2946-13388_rc      tgttaggaattttatgatttcttctgtcaattgtta-gtactttttga-agtaaagattgttaaaaa

                            3485
DS571302_24704-36084        tttacacttttgattaattaactcaacatctaacaaataaaaagctactcaaaagttacgacctaac
DS548521_2946-13388_rc      tttacacttttgattaattaactcaaaatctaacaaataaaaagaaactcaacttacacagaataac

                            3552
DS571302_24704-36084        aaacaaaat-aaaaatatcattgatattcttcaattctttgttaatct------------tttcttt
DS548521_2946-13388_rc      taacaaaataaaaaatactgtttattttctctcatt-tttgttaatttattttctcattatttcttt

                            3619
DS571302_24704-36084        cattaaaactaacttaaatatcatttttcttataaaccattaaaa-tatttcttatttgattattta
DS548521_2946-13388_rc      ctttaaaatttacttaaat---ctttttcttctaaacctttaaaattatttcttatc---ttattta

                            3686
DS571302_24704-36084        attatggatcat--tattcttTTAGAACATAAATGCTAACATGACTATCATCATCATGATGAAAATG
DS548521_2946-13388_rc      attatggatcattatattcatTTAGAACATAAATGATAACATGACTATCACCATCATAATGAAAATT

                            3753
DS571302_24704-36084        CCTTTAGCTCCATTACACTTTCCATCTTTTAATTTCCATCCATCTTCACATGTTGTGCATTCACCTT
DS548521_2946-13388_rc      CCTTTACTTCCATTACAATTTCCTTCTTCTAATTTCCATCCTTCTTCACATGATGTGCATTTTCCTT

                            3820
DS571302_24704-36084        CTCCTGTACATTTAGTACAATGTGGCATATGTCTTGAACATGGAATACATGATCCCCAACTAGATGT
DS548521_2946-13388_rc      CTCCTGAACATTTAGTACAATGTGCCATATGTCTTGAACATGGAATACATGATCCCCAACTTGTTGT

                            3887
DS571302_24704-36084        GTAATATCCTTCAACACATACACATTCTCCTCCTTTTGCTTCTAAATATTCAGCATTGCATCTCAAA
DS548521_2946-13388_rc      GTAATATCCTTCTACACAAACACATTCTCCTCCTTTCAATTCTAAATATTCTGCATTGCATTTCAAA
```

```
                           3954
DS571302_24704-36084       CAGTCTTTGGAATTACATGAATCACAAAACTCATTAACTTTAGCTTTGCATGATTGACATTCAATTT
DS548521_2946-13388_rc     CATTCATTTGAATTACATGAATCACAGAATTCATTAACTTTGGCTTTACATGATTCACATTCTATTG

                           4021
DS571302_24704-36084       TATTATCTTCTGTAGAAGTGCCTTCAACATATCCATATGCACAAACACATGCTCCATTCACTGGTAC
DS548521_2946-13388_rc     TATTATCTTTTGTTGAATTATTTTGAATATATCCATATGCACATGTACATGCCCCGTTCACTGGGAA

                           4088
DS571302_24704-36084       TTCAGCAATATGATCTTTTGTTGAACATTTAGTACATTCTTCTACTATTTTCTTCTTCTCAGAATTA
DS548521_2946-13388_rc     TTCTGAAATATGATCTTTTGTTGAACATTTTGTACAATGTTCTACTACTTTCTTCTTCTCTGAATTA

                           4155
DS571302_24704-36084       CATCCTGTTTCTTGTTCTTCACAATCTTTCTTTACTCCAACACATTTAGTGCATGGTGATGTGCATG
DS548521_2946-13388_rc     CATCCACTTTCTCCTTCTTCACAGTACTTCATTACTCCAATACATTTAGTGCATGGTGATGTACATA

                           4222
DS571302_24704-36084       GTATACATGTTCCTTTAATCTCATCAAAATAGAATCCATCTACACATGCATTACATCCATTTCCACT
DS548521_2946-13388_rc     GTATACATGCTCCTTTAATTTCATCAAAATAGAATCCATCTGCACATGAATTACATCCTTTTCCACT

                           4289
DS571302_24704-36084       AATTACCTTGTATGTATCACTCTCACATACTGTACATTCTGCGGCACTACTACATTCACTACAATGA
DS548521_2946-13388_rc     AATTACCTTATATGTATCGCTCTCACACACTGTACATTCTGTAGCACTAGTACATTCACTACAATGA

                           4356
DS571302_24704-36084       TTGGTACATGGCTTACATTCACCATTTGACATATAATAGCCTGTTTTACATTCATCACATTTACTTC
DS548521_2946-13388_rc     TTAGTACATGGTTTACATTCTCCATTAGACATATAATATCCTGTCTTACATCTATCACACTTACTTC

                           4423
DS571302_24704-36084       CTATCTTTAATGGATCAGTACATGTTAAACAAGTGTCTTTATCTGAACATGTTGCACATTTATCATC
DS548521_2946-13388_rc     CTACCATTAATGGTTTAGCACATGTTAAACAAACATGTTCGTCTGAACAAGTTGCACATTCACTACC

                           4490
DS571302_24704-36084       ACATTTAGCACACCCATT------------------CTTTTCTCCTTCTTTTGGATAATAATTAGAC
DS548521_2946-13388_rc     ACATTTGGCACATCCATTATTATTTCCATCCTCTCCCTTTTCTCCTTTTTTTGGATAATAGTTAGAC

                           4557
DS571302_24704-36084       TCAATACATGTAGTACAATTACTATAAGGACTACGTCCACTAAAACCATCTGAACATTCTGTTGCAT
DS548521_2946-13388_rc     AAAGTACATGCAGTACAATTGCTATAAGGACTACGTCCATTAAAATCTTCTGAACATTCTGTTGCAT

                           4624
DS571302_24704-36084       AGATATATCCTGTTCTACTATCAACATTACATGTGTCAGTAGTTTTACATCCTGGATATTTTGAAAG
DS548521_2946-13388_rc     AAATATATCCTGTTTTAGCATCAACATTACATGTATCAGTAGTTTTACATCCTTCATATTTTGAAAG

                           4691
DS571302_24704-36084       GTCAGAGCAACTTTGACACGTTCCATTTTCAAGTTGAACTGTTCCTGTTAGACAAGCACATGTTGGT
DS548521_2946-13388_rc     TTCTGAACAACTCTTACATCTTCCATTTTCAAGTTGAACTGTTCCTGTTGGACAAGCACATTCTGGT

                           4758
DS571302_24704-36084       GGTTTAATGTCTACAGAACTTTGGTCAGTGCATGTCACACATTCAATCTCCTTTTTATCGGCACTAT
DS548521_2946-13388_rc     GCTTTAATGTCTTGAGAAGTTGTATCAGTACATGTCAAACACTCAACCTCCTGTTTAGTACTATCAT

                           4825
DS571302_24704-36084       ATTTACATGTTTTACATTTTGCAGGACATGGACTACAATACACTCCATCTGTTGGACTTTCAATTTG
DS548521_2946-13388_rc     ATTTACATGTTTTACATTTTGCAGGACATGGACTACAATACACTCCATCCGTTGGGTTTTCAATTTT

                           4892
DS571302_24704-36084       ATAAAATCCATCTTTACATACAGTACATTGTGCATTTGAACCACTAGCAGCAAGAACTAAATTAGTG
DS548521_2946-13388_rc     ATAAAATCCATCTTTACACTCAGTACATTGTACATTTGAGTCACTAGCAGCAAGAATTAAATTATTA

                           4959
DS571302_24704-36084       TCTTCACATATAATACATGCTGTTGCTGAACATGCTGAACAATGTCCATATTTGTCCTTTTTAGTAG
DS548521_2946-13388_rc     TCTTCACATGTAATACATGCTGTTGCTGAACAAGATGAACAATGTCCAAACTTATCTTGTTTAGTAG

                           5026
DS571302_24704-36084       AACAAAGTTGGCATTTATTATTTTCATCTTTAAAAGCATTAGAAGCACACTGGGCACACTCCTTTGT
DS548521_2946-13388_rc     AACATAACTGACATTTATTATTACTATCTTTAAAAGCACTAGAAGAACATTGACTACAAGCATTAAT

                           5093
DS571302_24704-36084       TTGTGAATCAACAATATAAGCTGAGTCATCACATTCAAGACATGTAATAAATCCATCACTAATCTTA
DS548521_2946-13388_rc     TTCAGAACCAACTATATATGCAGGATCATTACATTCAAGACATTTAACATATCCATTACTAATCTCA

                           5160
DS571302_24704-36084       TAGTGACCTTCTTCTTTGCAATTATTATTTAGTTGTGCACAACTTTTCTTCTTATCTTCTGCATTTA
DS548521_2946-13388_rc     TACTTACCTTCTTGATTACAGTTTTCATTTAACTCTGCACAACTTTTCTTCTTATCTTCTTCATTTG

                           5227
DS571302_24704-36084       GTCCTTCTTTATACTCAGGAGTATCCTTTTTGCAAGCGCATTCATTATCTTTATCTCTTGTAGAGAC
DS548521_2946-13388_rc     GTCCTTCTTTATA------GCGTTCATCAAGGCAAGCACATTGACTGTTTTTATCCCTTGTAGTAAC
```

```
                     5294
DS571302_24704-36084  ACCTTGAATATTTTCATAACATTCACTACATACAACTTTACTTTTAGAACTATCATAAGAGCAACTA
DS548521_2946-13388_rc TCCTTGAATATTTTCATAACATGCAGTACATACAACAGCCTTTTTATTTGAATCATAAGAACAACTT

                     5361
DS571302_24704-36084  GAACAACTAGCTGGACATTCTTGACATTTATTGTTTTGATCAAAATAATGTGCATCACCACAAGAAC
DS548521_2946-13388_rc GAACAACTATCTGGACATTTCTGGCATACATTATTTTCATCGAAATAATGACCATCACCACAAGAAC

                     5428
DS571302_24704-36084  ATTTACCATTACTATCTACATGTGATCCAACTGGACATTGTGTACAAATGTTTTCATTGGCTGCTCT
DS548521_2946-13388_rc ATTTACCATCACTTCCTACATGTGATCCAACTGGACATTGTGTACAAATATTTTCATTTGCTGCTCT

                     5495
DS571302_24704-36084  TTCACATGATGGAAGTTTTGTTTTGATTGAAACACAGTCTGATGCTTTGTCAACTGACTTTGGAATA
DS548521_2946-13388_rc TTCACATGATGGAAGTTTAGTTACTATTGAAACACAATCTGATGATTTATCTATAGATGTTGGAACG

                     5562
DS571302_24704-36084  TATTTGTCATTACATTCACAATCATTCTTAACATCTCTATCCTTACCAACACAGCCAATAGTAATTT
DS548521_2946-13388_rc TGTTTATCATTACATTCACAATCATTTGAAACATTTCTTAATTGACCAACACAACCAATAGTAATTG

                     5629
DS571302_24704-36084  TATTATCTGAACC---TTTAGTAACACTGAAATAATATGGTAAATAAAGATCCTTAAGTGGACAATA
DS548521_2946-13388_rc CATT---TGAACTATCTTTAGTAACACTGAAATAATATGGTAAGAAAAGATCTGTAAGTGGGCAATA

                     5696
DS571302_24704-36084  ATAAGTATTATCAGATGCTTTAAATGCATTCAAATATTGTTCATATTGATCATATCCTCTCATCTTA
DS548521_2946-13388_rc ATAAGTATCATCTGATGCTTTAAATGCATTCAAATATTGTTCAAATTGATTGTACCCTCTCATTTTA

                     5763
DS571302_24704-36084  CACACTTGATGGTTGTTACCACTAGTACCAAGGACTTTTTTAACATCAAAATTATAGCAATGAGTTC
DS548521_2946-13388_rc CACACTCTATGCTCTTCACCACTAGTACCAAGTACATTAACAGAGTCTAGGTTGTAACAATGGGTTC

                     5830
DS571302_24704-36084  CATTCAATGTAAGTCCATTCTCACATTTATAACAAATAGTATCATCATCACTACATAATAAACATCC
DS548521_2946-13388_rc CTCTTAAAGTAAGTCCTTCATCGCATTTATAACAAATAGTATCATCATCACTACATGACAAACATCC

                     5897
DS571302_24704-36084  TGGAAGTTTGCATGGCCCATCAGTTGATGTTAAGTGTGCACTTCTAAGATTTAACTTACCTGGAACT
DS548521_2946-13388_rc TGGAAGTTTGCATGGACCCGCATTTGATGTTAAATGAGAACTTCTAAGATTTAATTTACCTGGAACA

                     5964
DS571302_24704-36084  GGTGTTTGGGAAGTAAGACATTCAGTAAATGGATCTGGACAAAGTGAACATCTCTTTTTATCTCCAT
DS548521_2946-13388_rc GGTGTTTTAGAAGTAAGACATTCAGTAAATGGATCTGGACAAAGTGTACATCTCTTATTTTCTCCAT

                     6031
DS571302_24704-36084  CTTTTTCTAAATAATAACCTTTTTGACATTCTGTCTTTGCAGATGTTTTGAAACCATCATTTAAATT
DS548521_2946-13388_rc CTTTTTCTAAATAATAACCAGGTAAACATTCTTCTTTAGCTGATGTTTTGAAACCATCATAGAAATT

                     6098
DS571302_24704-36084  TGGTGAACACTTTCCTGCACCTGTTAAGAAATATCCATCATCACATTTTGTACATTTATTTTCTTCT
DS548521_2946-13388_rc TGGTGAACAAGTTCCAGAGCTAGTAAGGAAATATCCACTATCACATTTTGCACATTTACTACTATCA

                     6165
DS571302_24704-36084  GTTGGTGAGAATAAACGACAATGATTCTTGACACAAACTCCATCAGAAACAGTACATCTTTTATTAC
DS548521_2946-13388_rc GTCAAAGAGAACAAACGACAATGATTCTTAACACAAACTTCACCAGAAACAGTACATCTTTTATTAC

                     6232
DS571302_24704-36084  ATGATTCACAATGTTCAGCGTTATAAATACTACACTCGTTACCTTCTGAGAGATCTTTTGAGTTATC
DS548521_2946-13388_rc ATGATGTACAGTGTTTAGCACTATAAATACTACATTCATCACCTTTTGAAAGATCGTCTAGATTATC

                     6299
DS571302_24704-36084  AATTGTACATACCTTACATTCTGTACCATTTAAATAATAACCTTCCATACAACCATTACACTTATCA
DS548521_2946-13388_rc AATTGTACATACTTCGCACTCTGTACCAGTTAAATAATAACCTTCCATACATGAAGCACATTTCTCA

                     6366
DS571302_24704-36084  GAATTCATCTTTGAACAATGATCTGGGTATTTAGTACATTTTTTTTCAGTATTAATATAATATCCAT
DS548521_2946-13388_rc CCATTTACTTTAGAACAATGGTCTGGATATTTGGTACATTTTTTCTCAGCATTAATATAATATCCAT

                     6433
DS571302_24704-36084  CTGCACATTGGTTACAAGAGCTTTCAACTTGTAAAATACACTTTTCAATCTTTTCATTCTTTTTAGT
DS548521_2946-13388_rc CTGCACATTGATTACAAGAATTTTCTACTTGTAAAATACACTTATCAATTTTAGTGAGTTGATTAGA

                     6500
DS571302_24704-36084  ACATTGACCACTTTGAAGAGAATACATACCAAAACATTTATCACATTGTTTTGAAGATGAATCTTTT
DS548521_2946-13388_rc ACACATATTATTTTGAAGGGTATAACTTCCAAAACATTTATCACATGCCTTGTTTGAAGAATCTTTA

                     6567
DS571302_24704-36084  GCGGTAAGACCAACACAATTTTCTGCATGTTCGTCTGTAGTTACTGTATCACATAACTTCTGACCTC
DS548521_2946-13388_rc GCTGAAAGACCGATACAATTTTCTGCATGGTCATCTGCAATTGCATTGTCACATAACTTTTCTCCTG
```

```
                          6634
DS571302_24704-36084      CATTAGTAGTAGTAGACATTCCAATAACACATTCACCACATTTATCGTT---GTCTTTTGAAAGACA
DS548521_2946-13388_rc    T------GGTTGAATATTTTCCTATAACACATTCACCACATTTATTATCTTGTCCCATATAAAGACA

                          6701
DS571302_24704-36084      TGACTCACAAGTATCAACACCATTCTTACATTTGCATTTCTCATTATTAGA---AGATGATGTATCA
DS548521_2946-13388_rc    AGTATCACAAGTGCTAACACTATTTCTACATGTACATTTATCAGTACTAGAGGTAGATGAATCATCA

                          6768
DS571302_24704-36084      CTTTCTTTTCCATTTTCACAATAAACACATTTTGGAGAATTAGAATCACTACCGTCATAATAAGAAA
DS548521_2946-13388_rc    ACTTCTTTTCCATCTTCACAATAAGTACATTTAGGAGTTTCAGAGTTACTGGTGTCATAGTAAGAAA

                          6835
DS571302_24704-36084      AAGCACTTTTACAAGTTTCTTCTTTAAGAGTACATTTTTGAGTACTTCCACTTCCAGACTCAGTTTT
DS548521_2946-13388_rc    ATGAACTTTTACAAGTACCTTCTTTAAGGACACACTTATT---------ACTTCCAGATTCAGTTTT

                          6902
DS571302_24704-36084      AAGTTCATAACCTTCATCACAACTAGCGCATGCTCCATTTGAGACTGATGCACAATGTGGAACAGCA
DS548521_2946-13388_rc    AAGTTCATAGCCATCATCACAACTAGCACATGCTCCATTTGAAACTGATGCGCAATGTGGAACAGCC

                          6969
DS571302_24704-36084      GTTCTTGGTTCTTGGTTATTAATGAGTTTATCAGCAGTATAATCACCAAGTGAAATTGATATGAATA
DS548521_2946-13388_rc    TCTCTTGGTTCTTTGTCTCCGATGAGTTTATCAGCTTTGTAATCACCAAGTGAAATTGATATAAATA

                          7036
DS571302_24704-36084      AAAGAAGAATAAACATgaataaaataaacagacgaagatctcttaaaaagggagagaatgtatgaag
DS548521_2946-13388_rc    AAAGAAGAATAAACATgaatgaaataaacagacgaagatctcttaaaaagggagagaagagttgaag

                          7103
DS571302_24704-36084      tgaaaacccaaattattactttatattttatcccgtatttttacgataattttctttgatatcgatt
DS548521_2946-13388_rc    taaaaaccaaaattattgctttatttttatcccgtttttttactattattttctttgttaataatc

                          7170
DS571302_24704-36084      gaattgaaactaataccattttattttttttttgaataacttcattatagttttttttccttttatt
DS548521_2946-13388_rc    gaattgaaactaatactattttactattttttttgaataacttcattatagttttttttccttttatt

                          7237
DS571302_24704-36084      aatagtaattaacctaaaagaatcttccttttctcgttgtgttattcttattcttgtctttgtgcag
DS548521_2946-13388_rc    aattgcaattgacctagaagaatcttccttttctcgttgtgttattcttattcttgcttttgtgtag

                          7304
DS571302_24704-36084      aaatgttgtaatttggtttcaatgtattcagaccattcttttgatactt--ttgtgtatttcttatg
DS548521_2946-13388_rc    aaatgttgtaatttagtttcaaggtattcagaccattcttctgacagtttcttgtgtatttcttttg

                          7371
DS571302_24704-36084      aaaaaagacagaaattagtacttgctgtgcttgcgaaagtcttaataatgttttattagaatagaat
DS548521_2946-13388_rc    aaaaaagacagaaattagcacttgctgtccttgcgaaactcttaacaatgttttattagaatataat

                          7438
DS571302_24704-36084      caatattttttgaggcatataaaatcgaatagttatccttatttggttcataattttcaaataaagg
DS548521_2946-13388_rc    tagtg-tttttgagtcatatgaattcgaataattctccttatttggtttataactttcaaataaagg

                          7505
DS571302_24704-36084      aacaaattgtgttttttttagggtattactttatgacacttttatttttattttttctttagaacct
DS548521_2946-13388_rc    agtgtattatg-ttttcttagggtattaatttatgacatctttatttttatttttgctttataagtt

                          7572
DS571302_24704-36084      tgaaagtggttttacgtcacttgataaattggttttattgctttgttattttctttgtcaataactt
DS548521_2946-13388_rc    tgaaggtaattctatgtcac-taagacagtggttttattggttggttattctctttgtcaatagttt

                          7639
DS571302_24704-36084      gcctttattctcaacgaatgagtcaaaccatttgttattttaaataaaaatatgaattgcataacgt
DS548521_2946-13388_rc    gtctttattctcagagaatgagtcaagctatttgttattttaaataaaaatatgaattgcataacgt

                          7706
DS571302_24704-36084      tgttttgaaatatcctcagaacttagttttttatacaac-tttaaagaagtaaaacaaagtgttttat
DS548521_2946-13388_rc    tgttttgaaacctctgaataaatttgtttctgtccaaagcttagagaggaaaagcaaagggctttat

                          7773
DS571302_24704-36084      gaattaattcaattaatgttatacatgttggtattaactcttatttaagtatttaaaaagaatatta
DS548521_2946-13388_rc    aaattaattcaattaatgatatatgttttgaccttaaatctta-ttaagtgtttaaaaa-aatattg

                          7840
DS571302_24704-36084      cagaaaaatcata-----aaaaaaatatgtttattaaaacaaattcaatgaggaaTTAGTTTGAAAG
DS548521_2946-13388_rc    caaggaaatcataggaataaaaaaatatgtttattaaaacaaattcaatgaggaatcaatttgagag

                          7907
DS571302_24704-36084      CATGAGGAAAAACATATTTCCCATAACACTGATGAAGGTAATAAAGAAAATCAAGAAGAAGAAAAGA
DS548521_2946-13388_rc    catgaggaaaaacatatttcccataacactaaagaaggtgataatgaaaattaagaagaagaaaagg
```

```
                            7974
DS571302_24704-36084        AATAAAGAGATTATAAATGCTGTGATGAGTTTTTGTTGACAAGTCTTTTTTGGTGTTTGTGCTTCTT
DS548521_2946-13388_rc      aataaagaggttataaatcccgtgataagtttttgttgacatgtatttttgggagtttgtgtttgtt

                            8041
DS571302_24704-36084        CTTGAGTCATAGGAATTACAGTAGAGTTGTTGTCATATGGAGGAGCTAATAAAGTGTCATTAGAAGG
DS548521_2946-13388_rc      cttgagtgacaggaacttcaatagaagggttgtcatgtggaggaactaatacaatttcattaggagg

                            8108
DS571302_24704-36084        AGTTAATGGTTGTTGAGGTAGAGAAGGGGTTGGTTG---TTGGTTATAAAAAGGATATGTACTATAT
DS548521_2946-13388_rc      gactgatggttgttgagggagagaagggattggttgagattggttatgaaaagagtatgcgttatat

                            8175
DS571302_24704-36084        CCAATAGGTTGATTAGCGTACAACATAGTAGGAGGTGTTTTTATTTCATTTTGATTAGTAACAGGGT
DS548521_2946-13388_rc      ccaacagaaggattagaatacaatatagtaggaggtatttttatttctgtttgattaatgacaggat

                            8242
DS571302_24704-36084        TGTATGGTTGTGTCATTTGGTTGGGTTGACAATAATACTGATTAAATGGTTGTTGGTTTGGGAAATT
DS548521_2946-13388_rc      tatatggttgtgctatttggttggtttgaccatagtactggttaagtggttgttggctttgaaaatt

                            8309
DS571302_24704-36084        ACCATTTATGTTTGAGTTAGGAGTGTATTGGTTGTTCATCATttctttattcaattaatgtttttac
DS548521_2946-13388_rc      accatttatgtttgggttgggagtgtattggttgttcatcatttatttgttagattaatg-ttttag

                            8376
DS571302_24704-36084        agttcttcttcttggcttacgtcaagaagaataaaaa-tgaaagcctctttcaataacaacatcatc
DS548521_2946-13388_rc      agttcttctttttgattttagttaagaagaataaagattaaaagcctcttt---ttacaataacttc

                            8443
DS571302_24704-36084        tatttttattttatgtaaaccattaaaaataatacattttcaagtcatctcatttagttgaataacg
DS548521_2946-13388_rc      tatttttattttgtgtga--cacttagaataatacctcttcaaatcatagtctttgtctaaac-aca

                            8510
DS571302_24704-36084        gtataatacaaaaaaagagaaatcaactaacctcactttcttttatagttttatatttttatctatc
DS548521_2946-13388_rc      atataata-aaaatgagagaaatcaaccaacccca-tttattttaccattttata-ttttatgtatc

                            8577
DS571302_24704-36084        a-------ttcataacgcctttattttaatagaaattaccagataaatcaagtag-atttaaacatc
DS548521_2946-13388_rc      aatataatttcatatatacttcatattgatagaaatt---taataaaccaagtagtctttaaacact

                            8644
DS571302_24704-36084        aattaaatcgttagagtcagtagacaaatgttttaaagataaatgtgtagtagaaaatcatattaac
DS548521_2946-13388_rc      tagtaaatagttaaagtcagtagataaatattttaaagataaatgaataatggaaaatcatatcaat

                            8711
DS571302_24704-36084        acaaaagc------attagatgttaatgatatagatataatatttataggggaaagaattgatgaat
DS548521_2946-13388_rc      acaagaataattaaattaggtgttaatgatataggtatgatgtttataaagaaaagaattgattagt

                            8778
DS571302_24704-36084        ttgtatctttctttatagtgaaatggaaa-gaaaagaaataaaactcaattgaaaatctcttttgaa
DS548521_2946-13388_rc      ttatatctttctttataacaaaatagaaattaaaagaaataaaagtcaattg--aacctctttcgaa

                            8845
DS571302_24704-36084        taatTTATTCTTCTTGTTTCTTTTTTGATTCGAGAATAACTTTACCTTCACTCATCCAATACTTTTC
DS548521_2946-13388_rc      tgatTTATTCTTCTTGTTTCTTTTTTGATTCGAGAATAACTTTACCTTCACTCATCCAATATTTTTC

                            8912
DS571302_24704-36084        TTCAGGACAATGATTTTTTTTCCATTCTGGATATTTGGTACAAGCCTTTTTTAAGTCAATAATGACT
DS548521_2946-13388_rc      TTCAGGACAATGATTTTTTTTCCATTCAGGATATTTGGTACAAGCTTTTTTTAAGTCAACAATAACT

                            8979
DS571302_24704-36084        TGACCAATCATACTCTTAGTAGCTTTGTTAACAACCCATGTAGGAATCCATCCATTCCATGAATTCC
DS548521_2946-13388_rc      TGACCAATCATACTCTTAGTCGCTTTATTAACAACCCATGTAGGAATCCATCCATTCCATGAATTCC

                            9046
DS571302_24704-36084        AACTAAAGAAACTAAGTTTAGTTCCCTCTGGTGTTTTTTCAATAATATAACCAGTTTTAAGACTTTG
DS548521_2946-13388_rc      AACTAAAGAAACTAAGTTTAGTTCCCTCTGGTGTTTTTTCAATAATATAGCCAGTTTTAAGACTTTG

                            9113
DS571302_24704-36084        AGCACGAACAAAATTTTTGTCAACAGGTGCTTTATCATGTTCTACTGAATGATTCATAATAATATAC
DS548521_2946-13388_rc      AGCACGAACAAAATTTTTGTCAACAGGTACTTTATCATGTTCTACTGAATGATTCATAATAATATAC

                            9180
DS571302_24704-36084        AAAGATTTGTCTTCATTGAACCACCATGATCTCATATTAACCCAATCACGGTTAGCTACTGTAAAAG
DS548521_2946-13388_rc      AAAGATTTGTCTTCATTGAACCACCATGATCTCATATTAACCCAATCACGGTTAGCTACTGTAAAAG

                            9247
DS571302_24704-36084        GCATTTTTACACTATAGTAACCAATTTCTGTATTTTCATCAATTTGTTCAACAAGATGTTGTTCTTT
DS548521_2946-13388_rc      GCATTTTTACACTGTAATAACCAATTTCTGTATTTTCATCAATTTGTTCAACAAGACGTTGTTCCTT
```

```
                         9314
DS571302_24704-36084     CATTGAACCATCCCATTCAGTTCTAAATTGTGGATCTTGAATTACATCATGTAATACATCAATAGGA
DS548521_2946-13388_rc   CATTGAAGTATCCCATTCAGTTCTAAATTGTGGGTCTTGAATTACATCATGTAATACATCAACAGGA

                         9381
DS571302_24704-36084     ATATCATGTAATGCCATTGACTTAAGTTTAATTTGTAATACACTTTCATTTGCCGCctttattaaat
DS548521_2946-13388_rc   ATATCATGTAAGGCCATTGATTTAAGTTTAATTTGTAATACACTTTCATTTGCTGCctttaataaat

                         9448
DS571302_24704-36084     tagtttttaggttttgtatttcttctgttattaacaaacATCTCTGAACAATACCTTAGTATCATT
DS548521_2946-13388_rc   tagttttttggttttatatttcttctgttattaacaaacATCTCTGAATAATACCTTAGTATCGTT

                         9515
DS571302_24704-36084     TTTATCTTGGTCAACAGTCCATCCTTCATCATCATCAAGTGTTTTTTAAATTCCATAAATTCATTT
DS548521_2946-13388_rc   TTTGTCTTGATCAAGAGTCCATCCTTCGTCATCATCGAGAGTCTTCTTGAATTCCATAAATTCGTTT

                         9582
DS571302_24704-36084     AAAGTTGGAACAGGATATTTTTCATTATGAGTGAATGTAAAAACATCCCCATATTCTTTTTTTACCC
DS548521_2946-13388_rc   AGAGTTGGAACGGGATATTTTTCATTATGAGTGAATGTGAAAACATCTCCATATTCTTTTTTTACCC

                         9649
DS571302_24704-36084     ATTCACTCATttcttgaactttaattacttaatataagttccaagttcttgatctaaataatcagat
DS548521_2946-13388_rc   ATTCACTCATttcttgaactttaaataattaatattagttctcagttcttgatctaaaaatccaaac

                         9716
DS571302_24704-36084     aagatttattgttttttttttattccttcaaatactactttttttattttatttttatttttttga
DS548521_2946-13388_rc   attttttattg----ttttttattcctacaaatacaattttcttatttt-attttt-attt---tga

                         9783
DS571302_24704-36084     tacttaaaagtaattgactatttgtaattgttcttgatttaaatctaaatgaatcaaattttaagaa
DS548521_2946-13388_rc   ---ttaaaagtaattgattatttgtaatcgttcttgatttatatttaaatgattcaaattttaataa

                         9850
DS571302_24704-36084     attacaatcaatattcattttgttttgttaaa-tgaaaagacaagaatattaaaagaaaaaaagtta
DS548521_2946-13388_rc   atcacaatcattatttcttttgttttattaaagtgaaaagacagaaatattaaaagaaaaaaagtta

                         9917
DS571302_24704-36084     taataaataaaaTTAGTATATAATATGAACCAGTTCAATATCTCCTCGTTTGATATCTAATCGTTTA
DS548521_2946-13388_rc   taataaataaaaTTAGTATATAATATGAACTAGTTCAATATCTCCTCGCTTGATATCTAATCGTTTA

                         9984
DS571302_24704-36084     ATTCCCATCTGAAACCGATGTACTAAAGCAGAATGGATTGCCCTTTCTTTCTGATGTATGTCATCTG
DS548521_2946-13388_rc   ATTCCCATTTGAAATCGATGTACTAGAGCAGAGTGGATTGCTCTTTCTTTCTGATGTATGTCATCTG

                        10051
DS571302_24704-36084     TTTTAATTAGTCCTTTCCCATACCTATATACACATCTAATTCCTTTTTTATTTCTTGAAATTTCAAT
DS548521_2946-13388_rc   TTTTAATTAATCCTTTCCCATATCTATATACACACCGAATTCCTTTTTTATTTCTTAAAATTTCAAT

                        10118
DS571302_24704-36084     TCCTTTTTCGTAAAGTAAATCCTCCAATAATTCAATCGTGTGTTGAATTCTTAAAGACTTAATCTTT
DS548521_2946-13388_rc   TCCTTTTTCGTAGAGTAAATCCTCCAATAATTCAATAGTGTGTTGAATTCTTAGAGATTTAATCTTT

                        10185
DS571302_24704-36084     TCATAGCCAAGTTTTGTTCTTATTCCATTTGTGTCAATAGGCTTTATTTCATTTTTCACTTTCTCAT
DS548521_2946-13388_rc   TCATAGCCAAGTTTTGTTCCAATTTCATTTGTGTTAATAGGTTTTATTTCGTTTTTCACTTTCTCAT

                        10252
DS571302_24704-36084     TAATTATATTTTCCAATTCAATAACCCCATCACCAAAATCAATTCCACTGATTTTTGGGCATCCATA
DS548521_2946-13388_rc   TAACTATATTTTCTAATTCAATGACCCCATCACCAAAATCAATTCCACTGATTTTTGGGCATTCATA

                        10319
DS571302_24704-36084     AGGAATTTCTTTCTTTTTACTAAACCGTTCAATTGTGATATTACACCATTTACTTAACATCCCAAGA
DS548521_2946-13388_rc   TATGATTTCTTTCTTTTTATTAAACCGTTCGATTGTGATATTACACCATTTACTTAACATCCCAAGA

                        10386
DS571302_24704-36084     AATAATGATATCTCATATACATTTTCTGGAACTTTTCTCTTGTGATGTTGTTTAAGAGGAGTTCCAA
DS548521_2946-13388_rc   AATAATGATATCTCATATACATTTTCTGGAACTTTTCTTTTATGATGTTGTTTAAGAGGAGTTCCAA

                        10453
DS571302_24704-36084     ACAATTGAAGGCGTTGTTGATTAATGATGAAGACTGGATTAATAGGGATTGAATTTGAATGCATttt
DS548521_2946-13388_rc   ACAATTGAAGGCGTTGCTGATTAATGATGAAGATCGGATTAATAGGGAT------TGAATGCATttt

                        10520
DS571302_24704-36084     ggtttattagtttcttcacctaaaaaatgaagaagaaaaaatagagtattaatataatttattaat
DS548521_2946-13388_rc   ggtttattagtttcttcacctaaaaaatgcagaagaaaaaatatagtattaatataatttattaat

                        10587
DS571302_24704-36084     attttctagtttatataaagattaatgattaTTACAATGACTTACTTTTAATAGCCTCTGTTTTTTT
DS548521_2946-13388_rc   attttctagtttatataaagatttatgactaTTACAATGACTTACTTTTAATAGCCTCTGTTTTTTT
```

```
                         10654
DS571302_24704-36084     CTTCTTAATAGCTTGTTTCAAATAGTTTGGTAATTGTGGTAATGTCATCCGTTTAGCACGTGTTTCT
DS548521_2946-13388_rc   CTTCTTAATGGCTTGTTTTAAATAATTTGGTAATTGTGGTAGTGTCATTCGTTTGGCACGTGTTTCT

                         10721
DS571302_24704-36084     ATTTCTGTCTTATTTAATAAAGATTTTCTTTTACGATCAATTTCACGAATAACTTTTTCTGCTCTTA
DS548521_2946-13388_rc   ATTTCTGTTTTATTTATTAAAGATTTTCTTTTACGATCAATTTCACGAATAACCTTTTCTGCTCTTA

                         10788
DS571302_24704-36084     ATTTAATTGCTCTATTAGCAGCATTTCGTTCTTTAAAGTCTTTAATGTCTTTTGTTGTGGTGGTTTG
DS548521_2946-13388_rc   ATTTAATTGCTCTGTTAGCAGCGTTCCGTTCTTTAAACTCTTTAATATCGTTTGTTGCAGTGGTTTG

                         10855
DS571302_24704-36084     ACGTTCTATCTTTTTTATTAATGATTGAGTGTCTGCAATTGTTTTATGTGTTTCTTTTAGAACATGT
DS548521_2946-13388_rc   ACGTTCTATTTTTTTTATTAATGATTGAGTATCTGCAATTGTTTTGTGTGTTTCTTTTAGAACGTGT

                         10922
DS571302_24704-36084     TCAGTTCGTTTTATTTGTTCATTTGTCTTATGCTTAATTTCTAATGACTTCCTTTTTTCTAACCTGT
DS548521_2946-13388_rc   TCAGTTCGTTTTATTTGTTCATTTGTGTTATGTTTGATTTCTAATGACTTCCTTTTTTCTAGTCGAT

                         10989
DS571302_24704-36084     TCATTGTTTGTATATGTGTTGTTTCAGTAAGTCTCTTTTTTGTTGGTGTTGAAGGTTTTTTTAGAAG
DS548521_2946-13388_rc   TCATTGTTTGTATATGTGTTGTTTCAGTAATTCTTTTTTTTGTTGGTGTTGGAGGTTTCTTTAGAAG

                         11056
DS571302_24704-36084     TTGTTGAACTCCAATCATTAATGGCTTTGTTTTAGTTTTTTTGTTAATGTTTATATGACTAAGATAA
DS548521_2946-13388_rc   TTGTTGAACTCCAATCATCAATGGTTTTGTTTTAGTTCTTTTATTATTAGTTATATGGTTAAGGTAA

                         11123
DS571302_24704-36084     GAAGGCATTATCGCTGTGTGCATTCCTATTTCTAACTTGGTATTTCCATTTCTCTCTTTAACTTTTT
DS548521_2946-13388_rc   GAAGGCATTATTGCTGTGTGCATTCCAATCTCTAACTTAGTAGTTCCATTTCTCTCTTTAACTTTCT

                         11190
DS571302_24704-36084     TCTTATTTGTTTTTAACATAGACAAAGCCTGAGAGGGTTCTTTTGGGATACAAGAAACGTCTACTAA
DS548521_2946-13388_rc   TCTTATTAGGTTTTAATACAGATAAAGCCTGAGAGGGTTCTTTGGGGATACAAGAAATATCTATTAA

                         11257
DS571302_24704-36084     ACTATAATCAGAAGGAACAGTTTCTTTACTTATTAGGTTAGTATTTTCATTAATTTCTTCTTTTTCT
DS548521_2946-13388_rc   GCTATAATCAGATGGAACAATTTCTTTACTTATTAGGTTAGTATTTTCATTAAC---TTCTTTTTCT

                         11324
DS571302_24704-36084     TCTTGAGGGTTTTTAAGTTCTTTTTCTGAGTGTGACAGTAAAGGAATTTCACTCACACACCCTTTTT
DS548521_2946-13388_rc   TCTTGAGGGTTTTTGAATTCTTTTTCTGAGTGTGCAAATAAAGGAGTTTCACTTGTACATCCTTTTT

                         11391
DS571302_24704-36084     TTATTTCAGACTTATCTGTTTTTGATGTTCTAACACCAGTAAGTAAGTGAAGAATATTAGCATTTAA
DS548521_2946-13388_rc   TTATTTCAGATTTATCTATTTTTGATGTTCTAACACCAGCAAGTAAGTGAAGAATATTAGAATTTAA

                         11458
DS571302_24704-36084     CTCTTGTGTAGTCGGAATTTTTGGTTGGAACTGTGTTAAGTCGATATGACGAGGTGGTCTAAATTCT
DS548521_2946-13388_rc   GTCTTGTGTAATCTGAATTTTTGGTTGGAATTCTGTTAAATCGATATGATGGGGTGGTCTAAATCCT

                         11525
DS571302_24704-36084     AAAATGTCAAGAATTTCTTTATCTTCAGAGATATTGATTGTTTTTGACTTTTTCATtccattatttt
DS548521_2946-13388_rc   AAGATGTCAAGAATTTCTTTATCTCCAGTGATATTGATTGTCTTTGACTTTTTCATtccattatttt

                         11592
DS571302_24704-36084     ccgatctctagtttttctcttttctacttctcagaaaattcgtttattgaaaaaagttgaaatatag
DS548521_2946-13388_rc   ccgatcttcagtttttctcttttctacttctcagaaaattcgtttaatgaaaaaagatttaataaag

                         11659
DS571302_24704-36084     gagaaaaagattatttttgtta
DS548521_2946-13388_rc   gagaaaaatattgtttttgtta
```
