# Supplementary material for: Evidence of Gene Conversion in Genes Encoding the Gal/GalNac Lectin Complex of Entamoeba
Source: PLoS Negl Trop Dis. 2011 Jun 28;5(6):e1209. doi: 10.1371/journal.pntd.0001209 (PMC3125142; doi:10.1371/journal.pntd.0001209)
Supplement: Figure S6 — Nucleotide alignment of orthologous genome regions of E. histolytica and E. dispar used to estimate inter-specific divergence around the light chain lectin orthologues EHI_049690 and EDI_071530. (PDF) [file pntd.0001209.s006.pdf]

Alignment: /Users/garethweedall/Desktop/lgl1.fas
Seaview [blocks=70 fontsize=10 A4] on Fri Nov 5 15:23:39 2010

```
                         1
DS571152_90156-108875    agaaaaaataacaaaaaatatgaataacaataataaaa--aataaaaaaataaaaacttcataagaac
Ed_consensus             A-AAAAAATAACAAAAAATAAGAATAACAAAAATAAAAATAAAAAAAAAATAAAAACTTCATGAGAAC

                        69
DS571152_90156-108875    ttaaaagaaaatgacaaaaaaataaaaaactgacATGAGTTTATCTCAACAGCAAATTGATCAATTGA
Ed_consensus             TTAAAAGAAAAAGAGAAAAAAACAAAAAAGT-ATATGACTTCATCTCAACAACAAATTGATCAATTGA

                       137
DS571152_90156-108875    TTCAAAACCTATATGGCAATGACAATGAAATTAAAACACAAACACTGAAAGAAATTAATGCTGTTATT
Ed_consensus             TTCAAAAGCTATATAGTAATGACAACGAAATTAAAGTACAAACATTAAAAGAAATAGATGGTGTTATT

                       205
DS571152_90156-108875    ACAACACATTGGGCAGAAATTTCAGAAGAACTTCCTAAATTGATTGAATTAAGCGAAACAATAGAAGG
Ed_consensus             ACAACACATTGGGCAGAAATATCAGAAGAACTTCCTAAATTAATTGAATTGAGTGAAACAATAGAAGG

                       273
DS571152_90156-108875    AATAGGAAAACAATATGCATACCTTGTTATTAGTAAGTCATATTTTTACATTGAAAGTTATGATGAAG
Ed_consensus             AATAGGAAAACAATATGCATATCTTGTTATTAGTAAATCATACTTTTATATTGAAAGTTATGATGAAG

                       341
DS571152_90156-108875    CTGTTAATTATGCATTAAAGGCAGATGAATTATTCAAATTTGAAGGAAATGATAATTATAGTGTAAAA
Ed_consensus             CAGTTAATTATGCATTAAAAGCAAATGAATTATTCAAATTTGAAGGAAATGATAATTATAGTGTAAAA

                       409
DS571152_90156-108875    ATGATTACACACTTAATAGATATGTATATTAAAGAACGTAGACAAAAGAAAGAAGTTGATAAATTAAT
Ed_consensus             ATGATTACACATTTAATAGATATGTATATTAAAGAACGAAGACAAAAGAAAGAAATTGACAAATTAAT

                       477
DS571152_90156-108875    GGAAGATAAAATGAATAAATTCTTTAAAGAGGCTATTGAGAAAGAAGAAGAATATCTAACGATTGGAA
Ed_consensus             GGAAGAAAAAAATGAATAAATTCTTTAAAGAAGCTATTGAGAAAGAAGAAGAGTATTTAACAATTGGAA

                       545
DS571152_90156-108875    TTGCATTAGATTGTAGAAGAGTTGATGTAATTAAAGAGATATTAGGAAAAACTAAAGAGAAAGATGAA
Ed_consensus             TTGCAATAGATTGTAGAAGAGTTGATGTTATTAAAGAGATATTAAGAAAAACTAAAGAGAAAGAAGAA

                       613
DS571152_90156-108875    ATAATAGAATATCTTAAGAAGATAACTAACAGTTCAAGTGTTGATTATTCATTAAAGAATGAAGTTAT
Ed_consensus             ATAATAGAATATCTTAAGAAAATAACTAATAGTTCAAATGTTGATTATTCATTAAAAAATGAAATTAT

                       681
DS571152_90156-108875    GGAAATTGTTTCAGAAAGTCTTAATGAAAATAGTCAAATTGATTTAGAAAGTATCAGTGAATGTTGGG
Ed_consensus             GGAAATTATTTCAGAAAGTCTTAATGAAAATAGTCAAGTTGATTTAGAAAGTATAAGTGAATGTTGGG

                       749
DS571152_90156-108875    TAAAAAAGAATGATGGAGATGGGTTTATGAAAATGTTTAACCAATTAAGTGAAGAAATGAAAATGCAA
Ed_consensus             TAAAAAAGAATGATGGAGATGGATTTATGAAAATGTTTAATCAATTAAGTGAAGAAATGAAAAAACAA

                       817
DS571152_90156-108875    GTATTACTTGATTATGAAGGAATTCCACAAAAATTTAGAGAAGAATTAATAAGTAAATTACCAGAGAA
Ed_consensus             GTATTACTTGATTATGAAGGAATTCCACAAAAATTTAGAGAAGAATTAATAAGTAAATTACCAGAGAA

                       885
DS571152_90156-108875    GTATCATAAATATATTGATGGAAAAATCCAAGAGAAGTTGTATTTAGACTTTTTATTCTCTAGAGATA
Ed_consensus             ATATCATAAATATATTGATGGAAAAATTCAAGAAAAATTATATTTAGACTTTTTATTCTCTAGAGATA

                       953
DS571152_90156-108875    AAACTGATAATTTATTATTGAATAATATTAAATCATCAAATTGTATTAAAAGTAGTGTAATTCAAACA
Ed_consensus             AAACTGATAATTTATTATTAAATAATATTAAATCATCAAATTGTATTAAAAGTAGTGTAATTCAAACA

                      1021
DS571152_90156-108875    GCAGTATTATATGCTAATGCATTTATGCATTATGGAACAACTAATATTATGTTTTTAAAAGATAATAA
Ed_consensus             GCAGTATTATATGCTAATGCATTTATGCATTATGGAACAACTAATATTATGTTTTTAAAAGATAATAA

                      1089
DS571152_90156-108875    TGATTGGATTATTAATGCAAGTAATTGGGGAAAATTTGCTACTACAGCTTCTCTTGGAGTTTTATTTA
Ed_consensus             TGATTGGATTATTAATGCAAGTAATTGGGGAAAATTTGCTACTACAGCTTCACTTGGAGTTTTATTTA

                      1157
DS571152_90156-108875    AAGGAAGAGAAAATGAAGCACTTAATTTAATGTCACCATATACAGCAAATGGAACAGGAGGAAAAAGT
Ed_consensus             AAGGAAGAGAAAATGAAGCAATTAATTTAATGTCACCATATACAGCTAATGGAACAGGAGGAAAAAGT

                      1225
DS571152_90156-108875    GTTTATGCACAATCAGGAAGATTATATGCATTAGGACTTATCTTTGGAGGACATGGAAAAGAGATTTT
Ed_consensus             GTTTATGCACAATCAGGAAGATTATATGCATTAGGACTTATATTTGGAGGACATGGAAAAGAAATTTT
```

```
                           1293
DS571152_90156-108875      AACAACAATAACTGATGATTTAAAACAATCAAAAAGTAGTAAAAATGAAGTTCTTCAACATGGAGCAT
Ed_consensus               AACAATAATAAATGAAGATTTAAAACAATCAAAAAGTAGTAAAAATGAAGTTCTTCAACATGGAGCAT

                           1361
DS571152_90156-108875      GTTTAGGAGTTGGATTAGCTGGAATTGCTACAGAAAATTATGAATTATATCAAGAAGTTAAAGAAATA
Ed_consensus               GTTTAGGAGTTGGATTAGCTGGAATTGCAACAGAAAATTATGAATTATATCAAGAAATTAAAGAAATA

                           1429
DS571152_90156-108875      TTAATTAATAATGATAGTGCAGTAGCAGGAATGACAGCTGGATTATCCCTTGGATTAATAATGATGGG
Ed_consensus               TTAATTAATAATGATAGTGCAGTAGCAGGAATGACAGCAGGATTAGCACTTGGATTAATAATGATGGG

                           1497
DS571152_90156-108875      ATCAGGTAATTTAGAAGTTGCACAAGAAATGCTTACACATTGTCATGAAACAGAACATGATAAAATTA
Ed_consensus               AACAGGGAATTTAGAAGTTGCACAAGAAATGCTTACATATTGTCATGAAACAGAACATGACAAAATTA

                           1565
DS571152_90156-108875      TTAGAGGAACTAGTGTTGGAATAGGATTAGTAATGTTTGGAATGCAAGATAAAGCTGATGGAATTATA
Ed_consensus               TTAGAGGAACTAGTGTTGGAATAGGATTAGTAATGTTTGGAATGCAAGATAAAGCTGATGAAATTATA

                           1633
DS571152_90156-108875      GATTTAATGGTTAATGATGCTAATCATGTATTAAGATATGGAGGAATTTATACAATTGGGTTAGCATA
Ed_consensus               GAATTAATGATTAATGATACTAATAATGTATTAAGATATGGAGGAATGTATACAATTGGATTAGCATA

                           1701
DS571152_90156-108875      TTGTGGAACTGCTAATGAAAAGGCTATTAGTAAATTATTACATTTTGCAGTCACAGATAGAAGTGATG
Ed_consensus               TTGTGGAACTTCTAATGAAAAAGCTATTAGTAAATTATTACATTTTGCAGTCACAGATAGAAGTGATG

                           1769
DS571152_90156-108875      AAGTTAAAAGAGCAGCAGTATTAGTGCTTGGATTTATTTTGAATAAGAGATTAGATGAGTTATGTAAA
Ed_consensus               AAGTTAAAAGAACAGCAGTATTAGTACTTGGATTTATTTTAAATAAAAGATTAGATGAATTATGTAAA

                           1837
DS571152_90156-108875      ACAATTTTATTATTAATTGATTCATATAATCCACATGTTAGATATGGAGCTGCACTTGCATTAGGTAT
Ed_consensus               ATAATTTTATTAATAATTGATTCATATAATCCACATGTTAGATATGGAGCTGCACTTGCATTAGGTAT

                           1905
DS571152_90156-108875      TGCAGGATGTGCATCAAATGATAGTACTGTTATTGGATTACTTGAGCCATTATTAAAAGATCCAAATG
Ed_consensus               TGCAGGATGTGCATCAAATAATAGTACAGTTATTGGATTACTTGAACCATTATTAAAAGATCCAAATG

                           1973
DS571152_90156-108875      ATTTTGTCAAACAAGGAGCTGCCATTGCACTTGGAATGGTTTTGATGGAAACATCTATTAAAGAAAAT
Ed_consensus               ATTTTGTAAAACAAGGAGCTGCTATTGCACTTGGAATGGTTTTAATGGAAACATCAATTAAAGAAAAT

                           2041
DS571152_90156-108875      GACAAAGTTGAAAAGTTTATTAAAGACTTACAAAATAAAATATCATTTAAAGGTGAAGGAATGTTAAC
Ed_consensus               AATAAAATTGAAACATTTATTAAAGATTTACAAAATAAAATATCATTTAAAGGTGAAGGAATGTTAAC

                           2109
DS571152_90156-108875      TCAATTTGGAAGTATTCTTGGATTAGGAATTGTTAATGCTGGAGGTAGAAATTGTACTATTTCAATGT
Ed_consensus               TCAATTTGGAAGTATTCTTGGATTAGGAATTGTTAATGCTGGAGGTAGAAATTGTACTATTTCAATGT

                           2177
DS571152_90156-108875      ACAATAATTTAGGTACATTTAATTTAAAAGCTGTTGCAGGATTAGTATTGTTTAACCAATATTGGTAT
Ed_consensus               ATAATAATTTAGGTACATTTAATTTAAAAGCAGTTGCAGGATTAGTATTATTTAATCAATATTGGTAT

                           2245
DS571152_90156-108875      TGGTATCCATTTACTTTATGTCTTTCATTGTCATTTATTCCAACAACAATCATTGGGGTTACAGAAGA
Ed_consensus               TGGTATCCATTTACTTTATGTCTTTCATTATCATTTATTCCAACAACAATTATTGGAGTTACAGAAGA

                           2313
DS571152_90156-108875      TTTAAAGTATGTTGAGTCATATCAATATATTTCTAATGCACCATCAGACCAATTTGATTATTTACCAA
Ed_consensus               TTTACAGTATATTGAATCATATCAATATATTTCTAATGCACCATCAGATCAATTTGATTATTTACCAA

                           2381
DS571152_90156-108875      TGACTAAACCACCAACAAAAACTGGACATACAAAAATGAACCAAACTAAATTATCTTATGGGAATAAA
Ed_consensus               TAACTAAACCACCAACAAAAACTGGACATACAAAAATGAATCAAACTAAATTATCTTATGGAAATAAA

                           2449
DS571152_90156-108875      CAAGCTGGACAATCACTTAATTTGTCTTCTAGTCTTTTAATTAGTGAAAAAATAACAAATGAAATTAT
Ed_consensus               CAAACTGGACAATCACTTAATTTGTCTTCTAGTCTTTTAATTAGTGAAAAAATTACAAATGAAATTAT

                           2517
DS571152_90156-108875      TCCAGAAGAAAAGAAAATGGAAGAAGAACCAATTCAAATACAACCATTTGTTACTTTACAAAATGGAA
Ed_consensus               TCCTGAAGAAAAGAAAATGGAAGAAGAACCAATTCAAATACAACCATTTGTTACTTTACAAAATGGAA

                           2585
DS571152_90156-108875      GTAGAGTCACTCCTCGTCAACTTGAATTTATTACTGAGATAAAAGGCAGTAGATTTGTTGGAGTTAAA
Ed_consensus               GCAGAGTTACTCCTCGTCAACTTGAGTTTATTACTGAGATAAAAGGTAGTAGATTTGTTGGAGTTAAA
```

```
                          2653
DS571152_90156-108875     AAACCAGGAAGAGGTATTTTGATCTTGAAAGATACTCAACCTCAAATGGAAGAAGAAAATTCAGGAGT
Ed_consensus              AAACCAGGAAGAGGTATTTTAATTTTAAAAGATACCCAACCTCAAATGGAAGAAGAAAACTCAGGAAT

                          2721
DS571152_90156-108875     TATTAAAGATCAAGGAATTGAAATGACAGAAGAACATAAAGCACTTGCTGATGCCTTATTATCCAATA
Ed_consensus              TATTAAAGACCAAGGAATTGAAATGACAGATGAACATAAAGCACTTGCTGATGCCTTATTATCCAATA

                          2789
DS571152_90156-108875     ACAATGATCCAAAACCACCTGAACCATTTGAATGGAACTTTTAAttgaaataaagttattt-gg---t
Ed_consensus              ACAATGAACCAAAACCACCAGAACCATTTGAATGGAACTTTTAATTAAAATAAAGTTATTTTGGTGTT

                          2857
DS571152_90156-108875     tttttttgtattatcattttaaactactttaaatttaatacaaaatagtttttt--------------
Ed_consensus              TTTTTTTATATTATCATTTTAATTCGTTTTAAACTTAATGAAAAATAGTCTTTTGATGTTAATTAAGA

                          2925
DS571152_90156-108875     -------------------------------------------ttttggtactattcattaatttgat
Ed_consensus              ATATTTAATCAAATGAAGAAGAAATTCTATTATTATTATATTCTCACAATATCATTTATTAATTTAAT

                          2993
DS571152_90156-108875     tgaattatttgaatgtgacatagtcg-aattaatatttatattatggtttttgatgcatttattttat
Ed_consensus              TGACTTATT-GAA-GTAATAAAGTAATATTTAATATTTATATTACAATTTCTGATATATTTATCTTAT

                          3061
DS571152_90156-108875     tgaattgatatatactctttatttattacaatattgcattattaactttttggtttattcatttattg
Ed_consensus              TAAATAAATAGATAATTCTTTCTTATT----TATTATATTATT-ACATTTTTATTTATTCATTTATTA

                          3129
DS571152_90156-108875     tttttgtgtttgttaattatgtattgtaacgacacagtgtt-ta-aatgaaatta-------tgaatt
Ed_consensus              TTTTTATGTTTATTGTTTATATATTGTAATAGCACAGTATTGTATAATGAAACTATTATTAGTGAATT

                          3197
DS571152_90156-108875     acaacaacaaaagggttttaaaatttattattcttagtttgtctgaattattattactttgtttcttc
Ed_consensus              --AATATAAATGATGTTTTGAACATTGTT--------TCTGTTTTAATTTTTATTA--TTCTTTCTTC

                          3265
DS571152_90156-108875     tttatattatttttatgatgtcttgtttgtacattgaactatagtatgtaatattaatcttcattgtc
Ed_consensus              TTTATTTCATTTTTAT----------------------CT-----------TATCAATCGTGGTTAT-

                          3333
DS571152_90156-108875     tattcatttctgtttatgaagtatttttaattttagaatgtttaactcttcatgatt-tgacaatttt
Ed_consensus              TAT------CTGTTTATGAAATAAATTTAATTTTAGAACGATTCACTCCTCTTGGTTATAACAAATTC

                          3401
DS571152_90156-108875     ttta-attctttcatgaaattctctttattctttgtcttattcaaattcaatttattatgaaatagat
Ed_consensus              TTTATATTCCTTC-----ATTCTATTTA-TCTTTGTCTTATTCAAATCCAATTTATTATGAAATAGAT

                          3469
DS571152_90156-108875     gcaaatcagtata-aatctttatactatcctaatttaacttcttatcattctataattgttttcacat
Ed_consensus              ATATATCAATACGTTTTCTTCATATTATACTAGTTAAAGT-----TCATACTAATACTCATTTTATGT

                          3537
DS571152_90156-108875     tatatttattactatgttttactacattcttttaatctttatttcatcgttatccatagaattatctt
Ed_consensus              TATTATTATCACTTCATTTCAT----TTGTTTTAATCTTTGTTCATTCATTATTCATTTAATTA---T

                          3605
DS571152_90156-108875     caataattctattgtaaatactctatttttatcaataatttgcataaaaagaatttttctt-ttataa
Ed_consensus              CAATAACT-TATT----------TATTTTTATCATTAATTTATTTGAAACAAATCTTTCTTCTTATTA

                          3673
DS571152_90156-108875     ttcaaaATGAAACAAAAATAACTTAATTTCCAAAAGTTATGCAAATACTGGAATAAAAACCTGTCTTG
Ed_consensus              TT-AAAACAAAACAAAAATAACTTAATATCAGAAAGTTAGGCAAAAACTGGAATAAATATCTGTCTTG

                          3741
DS571152_90156-108875     GTTCAGTTGCATTACACTTTGATGCTATAATTTGATAACTTATACTTGTTGTATTAAGGTGATTGTTT
Ed_consensus              ATTCTGTTGCACTACATCTTGAAGCTGTGATTTGGTACCCATTGGTAAGTGTCTTGAGGTGTTGATTT

                          3809
DS571152_90156-108875     CCAGTCCATTTTTCTTTTGCTTCATTCTTTTTCCTTGCTTGATAACAAAGGTCAACATCAAGTCGATA
Ed_consensus              CCAGTCCATACAGATCTACTATCAGATGCTTTTTTGGCTTGATAACAAAGGTCAAGATCAAGTCGATA

                          3877
DS571152_90156-108875     AGGAAGATAGAAGTGTCTTTTATATGTAGGCCATTCAAGAGTTTTTTCAACGACTCTTCCCTTATTAT
Ed_consensus              AGGAAGATAGAAATGTCTTTTATATGTAGGCCATTCAAGAGTTTTTTTAACCCATCTTCCACCCTGAT

                          3945
DS571152_90156-108875     TATACTTAACTTTGGAGTGGTATTCTGGATACCAATCAGTCGAATAAGGATGAACATAAACAAAACGT
Ed_consensus              TGTATTCAATAGTTGAATGATATTCAGCATACCATTCATTTGGATATGGATGGACATAAACAAAACGT
```

```
                         4013
DS571152_90156-108875    TGGTTTTTAGTACCTGGTGCATCTGATAATGGTGGATTTTTGGACCAAATAATAAGTGAATGTCCTTC
Ed_consensus             TGATTTTGAGTACCTGGTGCGTCTGATAATGGTGGATTTTTGGACCAAATAATAAGAGTTGACCCTTC

                         4081
DS571152_90156-108875    AAAAGCTCCTGCTCTAATATCGTGTACTCTTTTTGGAATTGCATAACTTGTGTACATTCTGTATGGTG
Ed_consensus             ATTTGCCCCTCCTCTAATATCATGTACTCTTTTTGGCATTGAATAGCATGTATACATTCTATATGGTG

                         4149
DS571152_90156-108875    CAAATTCAAAGAATTGAAGTTCATTTCCTTGTTTCAATGGTCTTAATAAAATGTTTTGTTCATAACCT
Ed_consensus             CAAATTCAAAGAATTGAAGTTCATTTCCTTGTTTCAATGGTCTTAATAGAATGTTTTGTTCATAATTT

                         4217
DS571152_90156-108875    CCATTTGCATATCTTACTGATCTTTTATCATCAAATTCCATATCCATAACATATCTATCATCTCCGTT
Ed_consensus             CCATTAGGGTATCTAACTGATCTTTTATCATCAAACTCCATATCCATAACATATCTGGTATCACCATT

                         4285
DS571152_90156-108875    TTTATCATCTGTATCAGTAAACTGTTTTTCAGTTTTGTAATTATAATCAGAAGCAAAAATAACTCTAC
Ed_consensus             TATATCGTCTGCATCAGTAAATTGCCTTTGTGTTTTATAATTGTAATCAGAAGCAAAAATAACTCTAC

                         4353
DS571152_90156-108875    AACATGTTTTACACTTGGCACGAACATAATTCTCTTGATTTGCACTGAAAACTCCACTCTCAGCAAAC
Ed_consensus             AACATGTTTTACACTTAGCACGAACATAATTTTCTTGATTTGCACTGAAAACACCATTTTCAGCAAAT

                         4421
DS571152_90156-108875    TGTTGAATCTGATAACTGTTAACATCACTCGTAAAATACGTGTTAAATTTTGTACCTTGGTTTTGTTT
Ed_consensus             CTTTGAATTTGATAACTGTTTACATCACTTGTAAATTCTGAATTAAAATTTGTATTACGATTCTCCAT

                         4489
DS571152_90156-108875    TCCATATGGATAATTTGGAGAAAATTGATCCCTATTATCATTGGTTTTACCAAATGAATAACTTATCA
Ed_consensus             TCCATAAGGATAGTTAGTAGAAAATTGATCTCTGTTATCAGAGGTCTTACCAATAGAATAAACTATCA

                         4557
DS571152_90156-108875    ATAAGATTAATATAATCATTgacaatattttagatgtctctatcgtttaagaataa--tctaaatact
Ed_consensus             ATAAGAATAATGCAATCATTGATAATATTTCAAATGAGTTTATAGTTTTATAATGAATTATGAATATT

                         4625
DS571152_90156-108875    tttttga-agaacatattc-----tactttaattctaataactattagactcaataatctattttttt
Ed_consensus             TTTTTAATAGAACATCTTCTGACATACTTTTTTCACAACGAATATTCG------TGGTTTA--TTTTT

                         4693
DS571152_90156-108875    aaaaaaaga--taaatcactttgttttaaaagaagagtcattattgaatataaagttagtcaaatcgc
Ed_consensus             AAAAA--GAAATA-------------------AAGGCTCCT---------------------------

                         4761
DS571152_90156-108875    attaattagcaatatgattttacacattatatattattacttatttaaggtaagtaaaataaaaaata
Ed_consensus             ---------------------------------------CTTTTTTAAGCAAAGT-------------

                         4829
DS571152_90156-108875    ttgagtaagtgatagtgtaatgtaatttttattacaaaaaaaaataatttcattctattttttagtat
Ed_consensus             -------------------------TTTTTGTTAAATATAGACACAGTTTT-----------------

                         4897
DS571152_90156-108875    aaaacaaaataacgtcagaagtaatatttattttttagaatttattccttctgtatgagaattgaa-t
Ed_consensus             --------ATAAAGTCAGAA-TAATATTGTTCATAAACAATACAATCTTTTTAAATGATAATTAAACT

                         4965
DS571152_90156-108875    agaaaacaagataaattcctttcacctagatcatgaacaaggacatatttcgtttcaacattctccta
Ed_consensus             AATAATTAATGTGCAT----------TAAATAATTA-------------TTATTTTAAATTTTTGC--

                         5033
DS571152_90156-108875    acagaaaaagaaaaaagatttttttaatgaacgaagaaaagaaaaaatgaagttatttaaaacaatat
Ed_consensus             -------------ACAGCTTTATTT-----------AAAAGAAAAA-------TATTTAAA-------

                         5101
DS571152_90156-108875    aaattgttgtattaaaaaaagtcaattaatagatatatccactattgaatattatgaatattgagaag
Ed_consensus             ---------TATAAAAATAA---------------------------------ATGAATAT-------

                         5169
DS571152_90156-108875    tgattatttcagtttcttcaatatgctctattggtgtaatagagttcatttctagcatgctcgcatta
Ed_consensus             ----------AGTTTAT--AATTTACTTCAATAATTTAA----------------------------A

                         5237
DS571152_90156-108875    gattgtttttcttctaatgtgctgccagaattgaatgtgtaaaacattaagtctttcatattttttat
Ed_consensus             CATAGTTTTTTGTTT-------------------ATGATTTAAATTTTAGGG-TTT------------

                         5305
DS571152_90156-108875    aattattgttcgtatgattttggaaaattc-taattgtaatttcagtgattttaatcttcttgctgac
Ed_consensus             --------------TGTTTTTAAAAGATTCATAGCTAT-TTTTTATTTATTTTATTTTTATTAAAAAG
```

```
                      5373
DS571152_90156-108875 tctttatgtattaatccatttattgataatataacaggtattatttctacatatattatttctctatt
Ed_consensus          TTTT------TTAATTCA---------AATA-----------------ACACAAATTAAT----TATT

                      5441
DS571152_90156-108875 atttcttaatttctcttgtaattctttatacttatttattttatgaatataatttaattctaaattat
Ed_consensus          ATTATTGAAT-----------------------------------AATATCATTGAGTTTTG------

                      5509
DS571152_90156-108875 tatcttgacatatagcaacatctataatatatcatttcttttctttcatattttgataatatatatct
Ed_consensus          ----TAAAGAAAAAGCAGGATATA-------------------------------ATAAAATACAAGG

                      5577
DS571152_90156-108875 ggtattctatcattgaatgaattatcaataaccaataaatctttattgtaagttattacaattttatc
Ed_consensus          GGATAAAGAAGAATGAATGAAATAACAATA-------------------AAATGATAATAAAAATGGA

                      5645
DS571152_90156-108875 attattaatacatttatgatgattattaaaatcaatagcatcaaattcattaaaatgtgtaattaata
Ed_consensus          AATATAAATACAATGAAAAAGG--GTTCAAA--AAGAGAAATAA-------GAAACGAAAAATGAAT-

                      5713
DS571152_90156-108875 tctgtttatatatttgtttacaaacatattcatgttttaatatttgattcttcttcattacatcataa
Ed_consensus          ---------------------AAATATAATGA-----TAATA------------------------AA

                      5781
DS571152_90156-108875 caagatatgactaatagatgcaatagtgttagggcaatgtttacaatattttaatgcatcattttgga
Ed_consensus          GAAAATAGGAATAA-AAATCAAAGGACATTAAGAGAG---------------AATGAA----------

                      5849
DS571152_90156-108875 ttattttagatttaatatttcctgtcatagttactcattcttgtatctttactatttggaggaatgca
Ed_consensus          -----------------------------------------------------ATAAAGAGAAA----

                      5917
DS571152_90156-108875 tttctttttatgtttaatgacctccatgtggctactgttaattttttatcattgaaagtgccatttgc
Ed_consensus          ---------------AATAA---------------------------------GAAAATAACG-----

                      5985
DS571152_90156-108875 acatcttatatattcaccacatttcttttctttcaattcatttaaatatctatcatttaaatcattat
Ed_consensus          ---------------------------------CAAGTTAATTGAAAAATGAGGATATGAAG------

                      6053
DS571152_90156-108875 ttacccattcaaatatttcctttacattgtgctttctttcatttaattctattacattcattaattca
Ed_consensus          ----------AAATA-----------------------------------------------AATAAA

                      6121
DS571152_90156-108875 ttaaaattaatcttcttacaaaatactttcttcattcttttaattattcaatttggattttcttttat
Ed_consensus          TAGAAAAGAA-------------------------------AAACATACAACAAGAAGT---------

                      6189
DS571152_90156-108875 tgcttctatcatttctcctatttctgtatttgatatttttattttaaatatattcttaataattcttt
Ed_consensus          -----------------------------------------------ATATA----------------

                      6257
DS571152_90156-108875 accacattcatctcttgctgacattaatcctaatcccaattcatttattttaatatataatctacttt
Ed_consensus          ------------------------TAATCCTGA---------------------ATAAAATGAAAATA

                      6325
DS571152_90156-108875 ttgggaatgtttctactatataattggagttgtattaatgaatttcttatttgtttatctaccttctc
Ed_consensus          GAGAGAGT----------TAAAGATGAAATTATAGAAAAAA---------------------------

                      6393
DS571152_90156-108875 caattctcctagtttccatgggacaatactaaataaatatcttaatttggagattatatcactattaa
Ed_consensus          -----CATCTAG-ATCAAGAAGAAAATAATAAAAGAAAGAAACAATCGAAAGA---------------

                      6461
DS571152_90156-108875 tctttcttattgtaatgaggctgctatatttcatagtgtttaatgatgataaaatagaagatatatca
Ed_consensus          --------------------------------------------GAGAAATAAGTAAAAGATGTAAAA

                      6529
DS571152_90156-108875 tttaatattcttgtacaatatttgttaatatttactttatctcctatttctataccaagatatttata
Ed_consensus          TACAA-------GAACAATAGATATGAAAG--------------AAATAAAATAACAAGCTGTGTATT

                      6597
DS571152_90156-108875 atgattgtcaatagttactcttggtagttcagctaattcaccatttatttgactattaatattagtca
Ed_consensus          AT--TTTGCAATA------------AATTGGTTAAATAAAAAATTAAACTGGAAATTAAAAAAAGAAA

                      6665
DS571152_90156-108875 ttatctccatcttttctatattaatttttaatcctattgcattaaatatttctttagttaatttgtgg
Ed_consensus          TTA-------------GATATAAATCACTTATCGT------------------------------TAG
```

```
                           6733
DS571152_90156-108875  gctttttccatttcatcgagattattaattattatctttaaatcattcatatataatatgtggtttaa
Ed_consensus           GTTTTTATCAT---GTTGAGA-----AGTGATTGT-TTCAGTTTATTCA--------ACACGCTTTA-

                           6801
DS571152_90156-108875  ctggaattgtttttcttctccatttattttaatatttgatttgttaatttgtttatctaattgattag
Ed_consensus           -----------TTTC---------TATTCAAATATTGTTTTTTCAAATATGCTCAACT----------

                           6869
DS571152_90156-108875  ataatatgtcaatagataatacaaataaaagtggagataaactgtcatttgtattattcctctttcta
Ed_consensus           ------------TAAATAACACCA-------TGGCTGTGATCTATCTTCTTTATCATTATTACTGTTT

                           6937
DS571152_90156-108875  cattaatcatacccacactttattgattataatataaattaagatgtgtattatttaatatatataat
Ed_consensus           AAATAATC-------CATTTTTCTTAATAACACACAA------------CGTATTCAGCATA---AAT

                           7005
DS571152_90156-108875  atactattaattactacaagtgggatattaaaataaattaa-acaatgtttaatcaattgatgattaa
Ed_consensus           CTATTACTAATAAATATAATTCAG---TTGTAGTAAAATAACACAAT---------------------

                           7073
DS571152_90156-108875  tgctatcatatgctttggatatgtcataatatgtttctactaacttcaatttagcctttaataataat
Ed_consensus           ---------------------TATCATAATA-------------------------------AATA--

                           7141
DS571152_90156-108875  tgttggattatagtgttatataataatccttcttttgatgcatatgtatatttcttacatccaatttg
Ed_consensus           ---------------------AATGGTCTTTATCATAATAAACA---ATACCTC-----CCCAAATCG

                           7209
DS571152_90156-108875  atttaatgatagttgtttttcaattggatttagcatcaattgattttctattttcatttttaatattg
Ed_consensus           ----------------------------CCCATCATAAA-----------------------------

                           7277
DS571152_90156-108875  atgtatatacttttaacaaacaatttaagcacgttattggtctgtaattctgtgggtctaatttatct
Ed_consensus           -------------TAATAACCAACTAA-----------------------------------------

                           7345
DS571152_90156-108875  catttcttatatactaatattgttcttccttgaataaagtgaagaagtatttcattaggattgtttaa
Ed_consensus           ---------------------------CCTTGAA-----TGGGGCATTATCTCACCAAGG-----TAA

                           7413
DS571152_90156-108875  ccattcattaaataattttaataaatatttcattggttattctaaatatttaatccaaaatccttgta
Ed_consensus           C--TTCATGAAAAGAC-------AATA----ACTGATGATTCAAAG----------------CTTG--

                           7481
DS571152_90156-108875  ttttatcattttcaatttactttcaaattattaatgaatttaatagcacagtctagttcattgggagt
Ed_consensus           --------------------------------GACGATTTTTAGGGTTCTGTGTAGTGTATCTGATGT

                           7549
DS571152_90156-108875  gattattgacattctattattaattatattttcaattaatatttcatataaattaatatttagataat
Ed_consensus           GACACCTTAGAACCTAT----------------GCTGAAGGTATCA-----------AGTTAAATAA-

                           7617
DS571152_90156-108875  ctataatagtattcgtttcatagattcctttccagaaattaatagtggagatattatctggatattca
Ed_consensus           CTAAAATCGAATTGATAAAATAAA---------AGAAGTCAATAG-----------------------

                           7685
DS571152_90156-108875  tcttctttagatttatttaaatgttgcattttccaatatttcatcattttataattccatcttattct
Ed_consensus           ----------------------------------------------CCTTGTAATTA-----------

                           7753
DS571152_90156-108875  tttccaattattctttcttgcttctatttctattattttcatctttcagaggtatttataatagttta
Ed_consensus           --------------------------------------------------------------------

                           7821
DS571152_90156-108875  tttgttcatttgtttttattttaatatgatatatatcatatttcttaatatgtaatattctttttaat
Ed_consensus           --------------------------------AGAACATAGCTGATGTTATAGGGCACTGTTTCTAAC

                           7889
DS571152_90156-108875  ttcatatattgtttattattaagtaaatctctcaatctacatatttttgactgtaatattctttctat
Ed_consensus           ATCATAGAGGA-----TACTTTGTAGAGCTGCTAGAGAACG-ACTCGGGACGGGATTAGTCTGCCC--

                           7957
DS571152_90156-108875  acttctctttccacttctttcttgtttatttttattaataatgttaatagtggaagctacagatttaa
Ed_consensus           ----------------------------------TGAATAGTAACAA------AAGATACCTATTC--

                           8025
DS571152_90156-108875  tatagtgataataatcaactaatgtattgattaatatttcatttttattaataatattaattatttga
Ed_consensus           -----TGTTAACAATAAATTAA---------------TTGCTTTTTATTAATAAAATTAGTT-TTTG-
```

```
                          8093
DS571152_90156-108875     tgtactttcttcttattgtaataataattaataaattgtacatcttcttgttctttcatttcttcttc
Ed_consensus              --------------------------------------------------------------------

                          8161
DS571152_90156-108875     tttaaatattttcattacttcaaattcttctatttcttctttcttgttcatttttgatattatttctt
Ed_consensus              -----------TCATTAACTCAGA--------------------------------------------

                          8229
DS571152_90156-108875     ttaattctttatctttcaattgatgttttattatttcttcctccattattctattgatttctctttta
Ed_consensus              --------------------------------------------------------------------

                          8297
DS571152_90156-108875     atttcattctttattttaaatgtcttctttaatatttctttatattcattaatatcatttactttaat
Ed_consensus              --------------------------------------------------------------------

                          8365
DS571152_90156-108875     tccaaattcttgtattagtctttctattatttcttctttaagttgacatcttaattttctttcaaata
Ed_consensus              --------------------------------------------------------------------

                          8433
DS571152_90156-108875     attttaaagcaaaacatttataaagtgttaaaatttcttcttttctatctgtttgcttcccccgtttt
Ed_consensus              ----AAAAGAAAAG-------GAAATGAAAAAAT----------------------------------

                          8501
DS571152_90156-108875     ttttgtttgtctaattctaatgtggtgatattcttatgctgaatatccatttcttcaatggatttctg
Ed_consensus              --------------------------------------------------------------------

                          8569
DS571152_90156-108875     cattaactttcttttttttggaagatcatcttcacttgatgatatttcatcagctactaccacattgg
Ed_consensus              ---------------------AAGA-------------------------------------------

                          8637
DS571152_90156-108875     gggtgagatcattcacaatttgtgtttcagatgagtctgttatattgtcatttatctcatctttcttt
Ed_consensus              ----------------------------------------AATATTGTTATTTAAAACA---------

                          8705
DS571152_90156-108875     ttttcttcttgtgtgttttcattttctaatttagataatttatcattcaggaataataattattatga
Ed_consensus              --------ATATATATTTTTGTATT-------------------------AAATAACAA--AATAAGA

                          8773
DS571152_90156-108875     tagattgtttaattcatacattttcctctcagattcagcatgaaattcaaatacttttttatcgcact
Ed_consensus              ------------------------------AAAGTAAGAAGAAAATTC--------------------

                          8841
DS571152_90156-108875     cgtggtttttttgtgcttatctccttctcatgcttcttctttaatgtcataatatcttttgttagttt
Ed_consensus              --------------------------------------------------------------------

                          8909
DS571152_90156-108875     ttaatggtttattcttttgcttttatttcatttttcatttcttctttctctttcatatcctcctccac
Ed_consensus              --------------------------------------------------------------------

                          8977
DS571152_90156-108875     tttcttttctatttcttttactctttttttcttttttactgttttcttcttccttttctaccagcctct
Ed_consensus              --------------------------------------------------------------------

                          9045
DS571152_90156-108875     tcttctctcctttagtaaacctcttcttctcctcagcaagcctctaaaagaaaagttagaagaaaatt
Ed_consensus              ---------------------------------------------AAAAGAAA----AAAAGAAAATT

                          9113
DS571152_90156-108875     caaaagaaag-aaaaagagaaaagaaaattcaaaagaaagattaaaagagtttattcatcaaaaagaa
Ed_consensus              CAAAAGAAAGAAAAAAGAAAATAGAAAAGTAAAAAGAATAAATAAATAAGTTTTTTCATCAAAATGAA

                          9181
DS571152_90156-108875     aagttgattgaaaagtattattaaattgtttttcatgaaaatcaatgatttaaacaacacaaaactga
Ed_consensus              AAGTCGTTTGAAAAGTAATATTAAA-TGTTTTTCATGAAAATTAATGATTTAAACCATACAAAACTGA

                          9249
DS571152_90156-108875     aattattcaaataATGATATTCTTCTTAAGTGTAGTTACTTTGGCTTTAGCAACTGAAGTCAATTTGA
Ed_consensus              AATTAATCAAATAATGATATTCTTCTTAAGTGTTATTACTTTGACATTGGCTACTGAAGTCAATTTGA

                          9317
DS571152_90156-108875     TGATGCCACTTGATACAGTCAATTCCAATGGAGTTAATAATAAAGGACAACTTCAAAATGACTTAAAT
Ed_consensus              TGATGCCACTTGATACTGTCAATTCTAATGGAGTTAATAATAAAGGACAACTTCAAAATGATTTAAAT

                          9385
DS571152_90156-108875     AAAATTAAATCAGGAGGAGTTGCTGGTGTTATGGCAGATGTTTGGTGGGGACTTGTAGAAACATCACC
Ed_consensus              AAAATTAAATCAGGAGGAGTTGCTGGTGTCATGGCAGATGTTTGGTGGGGACTTGTAGAAACATCACC
```

```
                         9453
DS571152_90156-108875   AAGAAATTACAATTGGAATGGATATAAAGAATTAGTTCAAATGGTTAAAAAAGCAGGATTAAAATTCC
Ed_consensus            AAGAAATTACAATTGGAATGGATATAAAGAATTAGTTCAAATGGTTAAAAATGCAGGATTAAAATTCC

                         9521
DS571152_90156-108875   AAGCAGTTATGTCATTCCATAAATGTGGAGGAAATGTAGGAGATTCAGTAACAATTGAAATTCCCCAA
Ed_consensus            AAGCAGTTATGTCATTCCATAAATGTGGAGGAAATGTAGGAGATTCAGTAACAATTGAAATTCCACAA

                         9589
DS571152_90156-108875   TGGGTAAGAAATGCAGGAGCAGCAAATGATGCGTTCTTTAAAGACAATGAAAATAATGTTAATAATGA
Ed_consensus            TGGGTAAGAAATGCAGGAGCAGCAAATGATGCATTCTTTAAAGATAATGAAAATAATGTTAATAATGA

                         9657
DS571152_90156-108875   ATATATTTCATTTGCATATGATGATTCATCTATTTTTGAAGGAAGAACACCAATTGAAATTTATAAAG
Ed_consensus            ATATATTTCATTTGCATATGATGATTCATCTATTTTTGAAGGAAGAACACCAATTGAAATTTATAAAG

                         9725
DS571152_90156-108875   ACTTTATGACATCATTTAAACAAAACTTCCAAAGTTATATTGATGATGGAACAATTAATGAAATTCAA
Ed_consensus            ATTTCATGACTTCATTTAAACAAAACTTCCAAAGTTATATTGATGATGGAACAATTAATGAAATTCAA

                         9793
DS571152_90156-108875   GTTGGTATGGGACCATGTGGAGAAACTAGATATCCATCATATCCACTTTCAAGGTGGAGTTATTGTGG
Ed_consensus            GTAGGTATGGGACCATGTGGAGAAACAAGGTATCCATCATATCCACTTTCAAGATGGAGTTATTGTGG

                         9861
DS571152_90156-108875   AGTAGGAGAGTTCCAATGTAATGATGGTAAGTCAAAAGAATTACTTAAAAAAGCAGCAACAGATAAAG
Ed_consensus            AGTAGGAGAATTCCAATGTAACGATGGTAAATCAAAAGAATTACTTAAAAAAGCAGCAACAGCTAAAG

                         9929
DS571152_90156-108875   GACATTCAGAATGGGGAAATGGATCACCATCAAATGCAGGAAATTATAATTCTAAACCACCCTCATCA
Ed_consensus            GACATTCAGAATGGGGAAATGGATCACCATCAAATGCAGGAAATTATAATTCTAAACCACCATCATCA

                         9997
DS571152_90156-108875   ACAGGATTCTTTGGAAATGGATTTGATAACTACCAAAGTGAATATGGACGATTCTTCCAAGAATGGTA
Ed_consensus            ACAGGATTCTTTGGAAATGGATTTGACAATTATCAAAGTGAATATGGAAGATTCTTCCAAGAATGGTA

                        10065
DS571152_90156-108875   TTTTGACTTGTTATTAAGTCATACAGATAAAGTTCTTTCAGCAGCAAGAAATGTATTTGGAAATACAC
Ed_consensus            TTTTGACCTGTTATTAAGTCACACAGATAAAGTTCTTTCAGCAGCAAGAAATGTATTTGGAAATACAC

                        10133
DS571152_90156-108875   TTGCACTTGCAGGTAAAATATCAGGAGTTCATTGGTGGTATAATGACCAATCACATGCAGCTGAAATG
Ed_consensus            TTGCACTTGCAGGTAAAATATCAGGAGTTCATTGGTGGTATAATGATCAATCACATGCAGCAGAAATG

                        10201
DS571152_90156-108875   ACAGCTGGATATTACAACTCAAATGGAAATGATGCATACAAAACACTTTCTAATACATTCAAAAATAA
Ed_consensus            ACAGCTGGATATTATAACTCAAATGGAAATGATGCATACAAAACACTTTCTAATACATTCAAAAATAA

                        10269
DS571152_90156-108875   TAATGTTAGATTTGACTTTACCTGTCTTGAAATGAGTGGAACTGATGGAAATTGTGGGTCATCACCAG
Ed_consensus            TAATGTTAGATTTGATTTTACATGTCTTGAAATGAGTGGAACTGATGGAAGTTGTGGATCATCACCAG

                        10337
DS571152_90156-108875   CTAATTTAGTTGATCAAGCATTTAATGCAGCAGGAACAGTTGGAATTGGCAAATGTGGAGAAAACGCA
Ed_consensus            CTAATTTAGTTGATCAAGCATTTAATGCAGCAGGAACAGTTGGAATTGGAAAATGTGGAGAAAATNNN

                        10405
DS571152_90156-108875   CTTGAATTATGTGGATATGGAGGATGTAATACTAATGGATTTAATCAAATCATCAATAAATGTAAACA
Ed_consensus            NNNNNNNNNNNNNNNNNNNNNNNNNNNNNNNNNNNNNNNNNNNNNNNNNNNNNNNNNNNNNNNNNNNN

                        10473
DS571152_90156-108875   ACATGGATTAACAGCATTCACATACTTGAGAATGACAAGAGGACTTCTTGATGATGGAAATGCATGGG
Ed_consensus            NNNNNNNNNNNNNNNNNNNNNNNNNNNNNNNNNNNNNNNNNNNNNNNNNNNNNNNNGGAAATGCATGGG

                        10541
DS571152_90156-108875   GACAATTCACTAATTTTGTTAGTAGAATGAAATAATacaacttaatttaataatgtttaactttcatt
Ed_consensus            GACAATTCACTAATTTTGTTAGTAGAATGAGATAATA-AAATTAATTTAATAACATGTTTACATCATT

                        10609
DS571152_90156-108875   tcatgtttaactttcattaattacattaaaaaactttaaatgaaatactgaattcaaattaacataat
Ed_consensus            TCATATTTAACTTTCGTTAATTACAATAAATAA-TTTAAATGAAATACAGGATGAAAATTAAAATAAT

                        10677
DS571152_90156-108875   gaattatgacattaaggtaatgaaatgtattgaaactatgaattgataaatcaattgtgatgaatgaa
Ed_consensus            -----------TTAATGCGTTGAAATGCACTGAAACAATGAATTAATACATTGACTGTGATTAATGAA

                        10745
DS571152_90156-108875   ataataaatattgtagttgatttataaaacataagattttgtacaacatattaatttattctttcaa
Ed_consensus            ACAATAAATGTTCTATTTGATTTATAAAACAACAAATGTTGTATATTATATCTATTTTGTTCTTTATT
```

```
                          10813
DS571152_90156-108875    atttattcaaaacattaacttacacaatttatgtatatttcttctttgatttgaagataacaatgaat
Ed_consensus             ACTTATTCATTCCA-TAATCCAAACAGTTTATTTATATTTC-TCTTTGATTTGGAAATAAAAATGTAT

                          10881
DS571152_90156-108875    gatattcatac-a-ataaacaaaaatatttcttaataaattaatcgtcttagaattacaacatgttca
Ed_consensus             TATGTTCAAATGATATAAACAAAAACATTTCTTATTGAACTAATCATCTTAGAATTACAAGATATTCA

                          10949
DS571152_90156-108875    ttttcagtaaaagacattttcacattaacacacagagaaaca--------------------------
Ed_consensus             TTTATAATAGAGAACATTTTCACATTAACATTAAGAAGAACCAGAATTATCAATGAACATTTATTTTC

                          11017
DS571152_90156-108875    --gaataact------------gtcttgaatcaaaaatacatttttcttactaataaacattttcttt
Ed_consensus             ATGAATAACTTAATCATTCATCATCTTCAATCAAAAACATATTTATCTTAATGATAAATTCATTATTT

                          11085
DS571152_90156-108875    gattcggttgaaagtaaccaattaaacgacaatgaaacattaaagaaaataattgctcaaacacatta
Ed_consensus             GATTTATTTGAAATTGAATATTTAAACAATACTGA---ATTTGAAAAAACAGTTATTCATACACTTTA

                          11153
DS571152_90156-108875    ataccatattctcaacatcacttaggaaaatagattttgtagaacag-aaaataaaaagcataagact
Ed_consensus             ATA--ACATTCCAAATATCAAATAGAAAAATGGATTTTGTTGAACAGAAAAATAAGAAGAACAAGATT

                          11221
DS571152_90156-108875    catttatttctaaaaataaagtttcaatcaaattcaatctacaaaaaatgtttgaattattaggtatg
Ed_consensus             TATTTATTTTT-AGAATAA---TCTAATCTAATTCAATATACAAAGATTGTTTAAATTATTAGGTATG

                          11289
DS571152_90156-108875    ttattgatacaaaatattccttattatattaatagtatatagtct-----gtgaaatgaataattata
Ed_consensus             TCATTGATACAAAATAAGTACTATTATATTAATGATAGATAGTTTATTTCGTGACAAGAATAATTATG

                          11357
DS571152_90156-108875    tgaaataggataaacaattcttcatttagtagacgatggtttaaaagaaaagtgaataattgtttcaa
Ed_consensus             TGAAATAGTTTTAAAGATTCTTAGTTATTTTAATAATTTCTTAAAAGAAAAAT-AATCAATGTTTCGA

                          11425
DS571152_90156-108875    aaaactataatttctttcattgctgtaaaatacacactaaaacaaaacttccaattataatgtgttga
Ed_consensus             AGAAATCTAGTCTTTTGCAT--ATGTAAAATGCCTATTAAAATAAAACTACTAAATATAATGTTTTGG

                          11493
DS571152_90156-108875    taaataataagatatttaattatatggtgttgaagtactaatcccattttcaaaagttactataaaca
Ed_consensus             TAAATAATAATACAATTAA-TAAAT-GCATTGAAGTACTAATTTTGTTTTTAAAAGTTACTATAAAAG

                          11561
DS571152_90156-108875    acggaataaaatacaaagaaaatataaaacaaaaacttagTTACTTCTTTGCTATCAAAGCAAAACAC
Ed_consensus             AAGGAATAAAATACAAAGAAAATATAAAACAGAAATCTAGTTACTTCTTTGCTATTAAAGCAAAACAC

                          11629
DS571152_90156-108875    CTTGGTGAGATGGAAGAATCAAATACTCTTTGACAACTCACTTGTTTACAATATTGTTGGAGATAAAG
Ed_consensus             CTTGGTGAGATAGAAGAATCAAATACTCTTTGACAACTCACTTCTTTACAATATTGTTGGAGATAAAG

                          11697
DS571152_90156-108875    AACTCTATCAACAACAATTGATGATTCTAATACTGGTGCAATTAATGCACCAAGGATAATAAATGCTG
Ed_consensus             AACTCTATCAACAACAATTGATGACTCTAACACTGGTGCAATTAATGCACCAAGGATAATAAATGCTG

                          11765
DS571152_90156-108875    CTCGTTCAACTAAGATTCCATTTATTGTTAGTTGATTAAAGATACTTTGTACATTCATGTTTGGTTCT
Ed_consensus             CTCGTTCAATTAGGATTCCATTTATTGTTAGTTGATTAAAGATATTTTGTACATTCAAATATTGTTCT

                          11833
DS571152_90156-108875    TTTGTGTTTAAATATTCTTGAATGAACGTCATCAATCTCTCATTATAAGTTTTGTCTCTATAAGTAAA
Ed_consensus             TTTTTATTTAAATATTCTTGAATGAAAGTCATTAATTTCTCATTATAAGTTTTATCTCGATAACTAAA

                          11901
DS571152_90156-108875    TGAAGTTCGTTTCTGAATATTCCTTAAAGCACTTTCAAAATATTCATGAAAACTACCATTCTTTGTTT
Ed_consensus             TGAAGTTCTTTTTTGAATATTCATTAAAGCAGTTTCAAAATATTCTTTAAAATTACTATTCTTTGTTT

                          11969
DS571152_90156-108875    TACCAGTTGGATGGTCTTCTTGTGACCCAAGAGTTTTATACATAATAAATTCAAATACTGCTTTGAAC
Ed_consensus             TACCAGTTGGATGATCTTCTTGTGATCCAAGAGTTTTATACATGATATATTCAAATACTGATTTAAAA

                          12037
DS571152_90156-108875    GATTGTTGTTTCATCATTTTAATAAAGTCATCTTTACTCATTTTCATTGGATCAGCAAATGTTGATAT
Ed_consensus             GATTGTTGTTTCATCATTTTAATGAAATCATCTTTACTCATTTTCAATGGATCAGCAAATGTTGATAT

                          12105
DS571152_90156-108875    TTCAGAATGTGCTAAATGGAAATGAATGGATTGTTTGACAAATTCACTCATAGGAAAACCATATTGTT
Ed_consensus             TTCAGAATGTGCTAAATGGAAATGAATAGATTTTTTAACAAATTCACTCATAGGAAAACCATATTGTT
```

```
                              12173
DS571152_90156-108875     TTTCATTGACATATGTTTGTTTCTTTTCAAGTGGATTTGTTTTCCAATTGTCTATCTCTTCTGTTAAT
Ed_consensus              TTTCATTAACATAAAACTGTTTCTTTTCAAGTGGATTTATTTTCCAATTATCTATTTCTTCTGTTAAT

                              12241
DS571152_90156-108875     TTATGATAACAACAACCAACAGCACAAATCATTTTAATTTGAGGGATATTAATTGAAAGTCTTAATAA
Ed_consensus              TTATGATAACAACAACCAACAGCACAAATCATTTTGATTTGAGGAATATGAATTGAGAGTCTTAATAA

                              12309
DS571152_90156-108875     TGTTGGAGTGAGATTACCACAAGCATGTAAACAAGTCATAAGAAATTCTTGTGATGAAGTTAATTTTT
Ed_consensus              TGTTGGAGTAAGATTACCACAAGCATGTAAACAAGTCATAAGAAATTCTTTTGATGAAGTTAATTTTT

                              12377
DS571152_90156-108875     GAACAATTTCCATGAATGCTTCTGATGTAATATCACTTGATAAATATTCACTAGCGACTTTGACGTCT
Ed_consensus              GAACAATTTCCATAAATGCTTCAGATGTAATATCACTTGATAAATATTCACTAGCAACTTTAATTTCT

                              12445
DS571152_90156-108875     TTCTTTGGTTCAATCTTTTCTATTTTAGCAACCTTTTGATTTCTATCTTTCATTGCTTTAACATATTC
Ed_consensus              TTCTTTGGTTCAAATTTTTCTATTTTAGCAACTTTTTGATTTCTCTCTTTCATTGCTTTGATATATTC

                              12513
DS571152_90156-108875     TTCTTTTCCTTCAATACCAATACATTCAATTCCCTTCTTTTGAAGCTCTTCCATCAAATAACCTTTTC
Ed_consensus              TTCTTTTCCTTCAATACCTATACACTCAATTCCTTTTTTTTGAAGTTCTTCCATTAAATAACCTTTTC

                              12581
DS571152_90156-108875     CACTTCCTATATCAATAACACATCCTTTCCAATATTGATTAATTAATGAACTTAATTCTTTACACTCA
Ed_consensus              CACTTCCAATATCAATAACACATCCTTTCCAATATTGATTAATTAATGAACTTAATTCTTTACATTCA

                              12649
DS571152_90156-108875     TATCCCTTCTTTTCATTCATCAATATCCATTGTTCTTTCTTTCCTTCAATATTTCCTTTAGGATATAA
Ed_consensus              TATCCCTTCTTTTCATTCATTAATATCCATTGTTCTTTCTTTCCTTTAATATTTCCTTTAGGATATAA

                              12717
DS571152_90156-108875     TAGTTTACATTTATCAATAAATTGTTGTAAACTTAATGGTACTTCAATATAATATTTTTTTGGAATAA
Ed_consensus              TAATTTACATTTATCAATAAATTGTTGTAAACTTAATGGTATTTCAATATAATATTTTTTTGGAATAA

                              12785
DS571152_90156-108875     TAGCTTTAATTTCGGGGTGAATTAATGCAAAGATATTTAATAAAATAATATCACTTTGTTCAGGGTAT
Ed_consensus              TCTCTTTAATCTCTGGTTGAATTAATACAAGAATATTTAATAAAATGATATCACTTTGTTCAGGATAT

                              12853
DS571152_90156-108875     TGCTTTCCCATTGTTTTAATCAAATCAAAATATTGATTCCTCCATTCTTCATTTATTCTTTCATAAAT
Ed_consensus              TGTTTTGCTAATGTTTTAATCAAATCAAAATATTGATTCCTCCATTCTTCATTTATTCTTTCATAAAT

                              12921
DS571152_90156-108875     TCCATTAATTAATACTCTTGTAGGTTTTTCAGTATTTAAATATTGACTTTCATTGTTATAATTATTTA
Ed_consensus              TCCATTAATTAATATTCTTGTAGGTCTTTCAGTATTTAAATATTGACCTTCATTATTATAATTATTTA

                              12989
DS571152_90156-108875     CACTAAAACAAACTTCATTTTGATTTAAGAAAGTAATCATTTCATTTTCATATTGTTGATAAGTTTCA
Ed_consensus              CACTAAAACAAATTTCATTTTGATTTAATAAATTTATCATTTCATTTTCATAATGTTGATAAGTTTCA

                              13057
DS571152_90156-108875     AATGAAGAAGGTAATATTTCATGTTGTGTAGATAATAATTGCATgatata-agttcaaatcagaaata
Ed_consensus              AATATAGAAGGTAATATTTCTTGTTGTATAGATACTAATTGCATTTTAAAGAATTTAAGTCAGAATTA

                              13125
DS571152_90156-108875     tgagtttttaaaaaagacttttaaaaagaagaaataaataaaaaaaaaaa----caaa-taaac-aaa
Ed_consensus              TAAGTTTTTAAAAAAGATTTTTAAAAAGAAGAAATAAATAGAAAAAAAAAAAAACAAAATAAATAAAA

                              13193
DS571152_90156-108875     caaataagaaagtagaataCAATAAACTATTTTACTTAACAATCACTTCTAATAATTATAATTCTATT
Ed_consensus              CAAATAAGAAAATAGAATACAAAAAACTATTTTACTTAACAATCAGTTCTAATAA-TATAA-TCGATT

                              13261
DS571152_90156-108875     ACATGTGGTTATGTTCTAATATCATTTTAGTAATATTCCACGTTCTTGTTTTTGTTCTATAATTTAAA
Ed_consensus              ACATATGGTTATGTTCTAATATCATTTTAGTAATATTCCAAGTTTTTGTTTTAGTTCTATAATTTAAA

                              13329
DS571152_90156-108875     TTAATTCTAAATGGACATTCAGTCTTTTTTGACCTTCTTTTCTTTATTCCTTTTGTCTGACAACAAAT
Ed_consensus              TTAATTCTAAATGGACATTCAGTCTTTTTTGATCTTCTTTTCTTTATTCCTTTTGTTTGACAATAAAT

                              13397
DS571152_90156-108875     CTCTTCAGTAGGAGATCCTGAACAACTACGATACTTTCCACTGCATTGACACACAAGAACTACTTTCT
Ed_consensus              TTCTTCAGTAGGAGATCCAGAACAACTACGATATTTTCCACTACATTGACATACAAGAACTACTTTCT

                              13465
DS571152_90156-108875     TCTTAGTTCCATCTTTCATTGTTTTATTATTACCACTACCTTTACGTAAAACTACTCCACATCCTTCA
Ed_consensus              TCTTAGTTCCATCTTTCATTGTTTTATTATTACCACTACCTTTACGTAAAACTACTCCACATTCTTCA
```

```
                         13533
DS571152_90156-108875    GCCCATTCAGTAAGGAATTTTATTGCTTCTTCATATGACTGAAATTCTTTCTTTCCTTCATATTTTGT
Ed_consensus             GCCCATTTAGTAAGTAATGATATAGCTTCTTCATATGATATAAATTCTTTCTTTCCTTCATATTTTGT

                         13601
DS571152_90156-108875    ATATCGTTTAACGTCTAAATCCTTTGGAACAGTAAGTTCACTTTGGTTATAATTACCTGGAACACTTT
Ed_consensus             ATAACGTTTAACTTCTAAATCCTTTGGAATAGTAAGTTCAGTTTGATTATAATTATTAGGAACGCTTT

                         13669
DS571152_90156-108875    GTGGTCGACTTCCATGCAATATTTCATTCTGGTTTGGAAGGGCAATTGGAGAAAACATTGATCGGTGT
Ed_consensus             GTGGTCTACTTCCATTTAACATTTCATTCTGATTTGGAAGAGTAATTGGAGAAAACATTGATCGATGT

                         13737
DS571152_90156-108875    ACTGGCTGAAGTACAACCTCTCCTGTTGCTGAAAATGGACGAGGATGTCCCAAAGTACTAATTGGTTT
Ed_consensus             ATTGATTGAAGTACAATCTCTCCTGTTGCTGAAACTGGACGAGGATATCCCCTAGTATTAATTTGTTG

                         13805
DS571152_90156-108875    AGTTGAGAATGACGTAGTATTAATAGGAGAAAATAATGTTCTTTCAATCATTGGAGATATAGAAGTAC
Ed_consensus             TGTTGAAAATGAATTAGTATTAATTGGAGAATATAATGTTCTTTCAATCATTGGAGATATAGAAGTTC

                         13873
DS571152_90156-108875    GTTGTGTAGTATAAAGTTTAGAAGGCAATGATGGTTGAAGGGAAGTTTCGTTTAAATTAGGAGTATCT
Ed_consensus             GTTTTGTAGTATAAATTTTAGATGGTAATAATGGTTGAAGAGGAATTTCATTTAAATTAGGAGTATCT

                         13941
DS571152_90156-108875    TGTCTCTCTATTTTTATTTCTGACATTTCCTGATCTTGAAAAGGTAATGGATGTTGTTGTGAAGATGT
Ed_consensus             TGTCTTTCTATTTTTATTTCTGACATTGCTTGATCTTGAAAAGGTAATGAATGTTGTTGTAAAGATGT

                         14009
DS571152_90156-108875    ATTTGTTATATGACTGATTTCACTGGAATAATTATAAGTTTGATTAACAACTGGAAGTTGTATAGAAC
Ed_consensus             ATTTGTTATATGACCATTTTCACTAGAATAATTATAACTTTGATTAACAATTGGAAGTTGTATAGAAC

                         14077
DS571152_90156-108875    CAAGTCCAGAAGAAATACGACATGACATAGTTTCTTCATTAGGAAGTTGTATTTGTGTGTTTTGACTT
Ed_consensus             CAAGTCCAGATGAAATACAACTCGATATATTTTCTTCATTAGGAAGTTGTATTTGTGTATTTTGACTT

                         14145
DS571152_90156-108875    ATAGATAATTGGTGTGGACTATCCATTGTAATAGGTCTAAAATGACATGTTTGAGGTTGAACAACATC
Ed_consensus             ATAGATAATTGATGTTGATTATCAATTGTAATAGGTCTAAAATGACATGTTTGAGGTTGAATAACATC

                         14213
DS571152_90156-108875    ATTAAGATTTGGTTCTAACATTGATTCGTATTGATGTTCTGAACTTATTGGAGAGAATTGAGTATATC
Ed_consensus             ATTAAGATTTGGTTCTAACATTGATTCATATTGATGTTCTGAGTTTATTGGAGAGAATTGAGTATACC

                         14281
DS571152_90156-108875    TAATTGGTTGTTTATTGAATTGGGTTTTTCTTTGATTAAATAGAGAATAATGTTGGTTTGTGGTATCA
Ed_consensus             TAATTGGTTGTTGATTAAATTGGCTTTTTCTTTGATTAAAAGAAGAATAAGGTTGGTTTGTGGTATCA

                         14349
DS571152_90156-108875    ATAAGCGGAAACATcaattataaaaggttgtttcatttttgtttttctctctttttattttctaataa
Ed_consensus             ATAGGCGGAAACATCAATTAAAAAAGGTTATTTCATTTTTGTTTTTCTCTCTTTTTATTTTCTAATAA

                         14417
DS571152_90156-108875    gtt---------------aaaaagaaatgaagtaatttttactatatttgtattatattttttatgtc
Ed_consensus             GTTAATTAAAAAAAAAAAAAAAAGAAATAAAGTAATTTTTACTATATTTGTATTATATTTTTT-TATA

                         14485
DS571152_90156-108875    tctaaaaatagaaaaataataataaaattctttgtt-atttatttt-ttgcagcttccttagtttcaa
Ed_consensus             TCTAAAAATAGAAAAATATTTATAAAATTCTTTGTTAATTTATTTTATTTTAGCTTCCTTAGTTTCAA

                         14553
DS571152_90156-108875    atgaagaatgaacgtaaaataacgatagtttaaaataatattttttattataaatttactataaacta
Ed_consensus             ATGAGGAAAAAATATAAAATAATTATAATTTAAAATAATATTTATTATTATAAATTTACTATAAATTA

                         14621
DS571152_90156-108875    tgaat-atattaatgatgttTCACACTATTTCTTCTTCTGTAGGAAGTTTTATCTCACTGTCATCTTT
Ed_consensus             TGAATAATATTAATGATATTTCACACTATATCTTCTTCTGTAGGTGGTTTTATATCATCTTCATCTTT

                         14689
DS571152_90156-108875    TGAAAGCGAAGTATCTTCAGTAATACAAATTTCTTTATCTAATTCTTCATCTATTTGATCAATATCTT
Ed_consensus             AGAAAATGAAATATTTTCAGTAATATATAATTCTTTATCTAATTCTTCATCTATTTGATCAATATCTT

                         14757
DS571152_90156-108875    CACTATTAAAAAATTCTTCTTCTATTTCTTGTGCTTTAGAAATATTATATCTTTGTTCATTATCTAAC
Ed_consensus             CACTATTAAAAAACTCTTCTTCTATTTCTTGTGTTTTAGAAATATTATATCTTTGTTCATTATCTAAC

                         14825
DS571152_90156-108875    CAACACCCATCTGTAAAAATAAATCCATCTCTCTTTTCATCAGTGATGATCATGATTGCAGTAAGTGC
Ed_consensus             CAACATCCATTAGTAAAAATAAATCCATCTCTTTTCTCATCAGTAATAATCATTATTGCAGTAAGTGA
```

```
                      14893
DS571152_90156-108875 AACAAATTGAATATTTTCTTTTCTCTCACAAACTAAATAATATGATTCATCATCTCTCTTTTCTCCTA
Ed_consensus          AACAAATTGAATATTTTCTTTTCTCTTACAAATTAAATAATATGATTCATCATCTCTCTTTTCTCCTA

                      14961
DS571152_90156-108875 ACTCAACATACCCAACACATTTTCCATTCTCTAAGACATTTAATTTTCCATATTTGTCTTGTCTAATT
Ed_consensus          ATTCAATATATCCAATACATTCTCCTTTCTCTATAATATTTAATTTTCCATATTTGTCTTGTTTAATT

                      15029
DS571152_90156-108875 ACAATTGAATCTGATTCATCTCCCCAAACAATTGAATTTTCATATAATCCTAAATACCATTTACCATG
Ed_consensus          ACAATTGAATCTAATTCATCTCCCCAAACAATTGAATTTTCATATATTCCTAAATACCATTTACCATG

                      15097
DS571152_90156-108875 TACTAATTTTGCTGTAGAGTCATACGTTCCTATTAATCTAATGATTGGTTCACAATTTTCATTTCTTT
Ed_consensus          TACTAATTTTGCTGTATAATCATAAGTTCCTATTAACCTAATGATTGGTTCACAATTTTTATTTCTTT

                      15165
DS571152_90156-108875 GAACACTAATAACACTTCTTCGTTGTTTTTTACAATGAAGTATAACCCTAAAAAATGGAGTTCCTGGA
Ed_consensus          GAATACTAATAACACTTCTTCGTTGTTTTTTACAATGAAGTATAACCCTAAAATATGGAGTTCCAGAA

                      15233
DS571152_90156-108875 ATAATATCTTTATTTGGTATTTCATTTAATAAACAGAGATAAAAGTAATCTTTACCAAAGAGTTGACT
Ed_consensus          ATGATATCTTTATTTGGTATTTCATTTAACAAACATAGATAAAAGTAATCTTTACCAAAAAGTTGACT

                      15301
DS571152_90156-108875 AGTTAATGTTATTTCTTTAGAAAGACTCATTTTACTAAATAATAAAGGTGTATTAATACAAAAATAAT
Ed_consensus          ACTTAATACTATTTCTTTAGAAAAATTTATTTTATTAAATAATAATGGTGTATTAATACAAAAATAAT

                      15369
DS571152_90156-108875 TTTGGTTTTTAATACAAATAAGACCAACTTGACCAAAAGGTTCTTCATCTTTTTTTGGGTATATTCCT
Ed_consensus          TTTGATTTTTAATACAAATAAGTCCAACTTGACCAAAAGGTTCTTCATTTTTTTTTGGGTATATATTT

                      15437
DS571152_90156-108875 TTTTCTTCTTGCAAATAAGCATATAAAATTCCATTCTTTAATTGCCATCTTGTACATCCTTTTTCTTT
Ed_consensus          TTTTCTTCTTGTAAATATGCATATAACACTCCATTCTTTAATTGCCATCTTGTATATCCTTTTTCTTT

                      15505
DS571152_90156-108875 TTCTGTAATAGTAAGTTTGAATGGATAAGTTCGTTCATATGTTAGTCGCATATTTTTACCTATACATG
Ed_consensus          TTCTGTAATAATAAGTCTGAATGGATAAGTTCGTTCATATGTTAATCGAATATTTTTACCTATACATG

                      15573
DS571152_90156-108875 GAACTCCAAGTATTGAACTAACAGAAATACAACATGTTTGAGAAAGTACATCTGGTTCAATCATTTCA
Ed_consensus          GAACTCCAAGTATTGAACTAACAGAAATACAACAAGTTTGAGAAAATACATCTGGTTCAATCATTTCA

                      15641
DS571152_90156-108875 AAGAGTGTTTGATTTTGTTTATTATTATTTGGAACAAGAAGTATTTTATTTCTTAATAATCCAGGTAC
Ed_consensus          AAGAGTGTTTGATTTTGTTTATTATTATTTGGAACAAGAAATATTTTATTTCTTAATAATCCAGGTAC

                      15709
DS571152_90156-108875 ACAAGACATATATTGATATGTACTTTCATCAGACCCAATTCTATAAGAGAAAGTCCAATCACTAATCA
Ed_consensus          ACAAGACATATATTGATATGTACTTTCATCAGATCCTATTCTATAAGAGAAAGTCCAATCACTAATCA

                      15777
DS571152_90156-108875 Tctaatttatgatagaaattactgataattttaagttttaattttactgtcatcttctattcttttca
Ed_consensus          TTTGATTTATTAAAGAAACTATTGTTAATTTTAAGTTTTAATATTACTGTCATTTTCTATTCTTTTCA

                      15845
DS571152_90156-108875 tt-tatttaattagatat----------aaaaaagaagttaattgacaaataagaaaca----caaaa
Ed_consensus          TTAGATTTAATAATGAATAAAATAATAAAAAAAAGAAGATAATTGATAAATAAGAAAAAATATAAAAA

                      15913
DS571152_90156-108875 ccttctattagttaaataaaattcctTTAATTTAGAAAGTACATAATTATTTTTATTTTAAGTAAATT
Ed_consensus          CTTTCAAATAATTAAATAAAATTCCTTTAATTCAGAAAGTACATAATTATTTTTATTTTAAGTAAATT

                      15981
DS571152_90156-108875 TAATACCATCAGAATTACCAAAAATATTGATAAGTAGAGGATTATATTAATTGTTTCATCAATAACTG
Ed_consensus          TAATACCATAAGAATTATCAAAAATATTGATAAATAGAAGATTATATTAATTGTTTCATTAATCACTG

                      16049
DS571152_90156-108875 CATATTGTTCATTTCTTTTATTTTCTTCTTTTGTTTCTTCTTCATGTTCAAGAAAATGTTTTATTTTC
Ed_consensus          TATATTTTTCATTTCTTTCAGTTTCTTCTTTTATTTCTTCTTTATTTTCAAGAAAATGTTTTATTTTT

                      16117
DS571152_90156-108875 TCTTTTTCTGCTTCTTGTCTTTTTTTTAAAATTGTTAAAACATCATCTTCTTGAGTAATTTCTTCTTC
Ed_consensus          TCTTTTTCTGCTTCTTGTCTTTTTTTTAAAATTGTTAAAACATCATCTTCTTGATTTATTTCTTCTTC

                      16185
DS571152_90156-108875 TTTTTCTTTTGTGTCTTTTACAATTTCATC---TTCTAATTTTGTAAGTAATTCCTCCTTACTTTCAC
Ed_consensus          TTTTTCTTTTGTTTCTTTATTAATTTCATCATTTTCTATTTTTGTAAGTAATTCTTCTTTACATTCAT
```

```
                        16253
DS571152_90156-108875   TATTTATTTCATTTTGAGTTATTACATTACTTTCTTCTTTCATAATCTCTTGATTTTGATTATTATTA
Ed_consensus            TATTTATTTCATTTTGAGTTATTATATCATTTTCTTCTTTTATAATATCTTGTTGTTGATTATTATTA

                        16321
DS571152_90156-108875   TTTTCTTTTTCGGTGATTATTTCATTATCTTTTTCTACATTTTCTAATTTATGTTCAATCTTATCAGA
Ed_consensus            TTTTCTTTCTCAATAATTATTTTATGATCTTTTTCTACATTTTCTAATTTATATTCAGTTTTATCATA

                        16389
DS571152_90156-108875   GTCTGACAATTTGGATAGTACTTCTTTAGAAGTAGTACTCGTTTTTGATTGATATTCAACACTTTCAG
Ed_consensus            ATTGGATAATTTAGATGTAACTTCTGTAGAAGAAGTATTAATCTTTAATTGATGTTCAATATTTTCAG

                        16457
DS571152_90156-108875   TTGAATTCTTTTTACCACTTTCATTTTCTAGCATAAATACTTTCTTTAATTCTTGGTGATTTGCACCA
Ed_consensus            TTAAATTCTTTTT------ATCATTTTCTAGTAAAAATATTTTCTTTAATTCTTGATGATTTGCACCA

                        16525
DS571152_90156-108875   CAAATAGGACAAGTGTAATATTGTGATTTTATAGCAAATTCTTTTTTTATATCTGAACAAGTTTCTAA
Ed_consensus            CAAACAGGGCAAGTATAATATTGTGATTTTTTAGCAAATTCTTTTTTTACATCTGAATGACTTTCTAA

                        16593
DS571152_90156-108875   CCCACCAACAGCACCTGCTGCAGGGGTTAACATAAATGCTCGAATTGCTTCAAGCATTGAAGAGATAC
Ed_consensus            TCCACCAACAGCACCTGCAGCAGGTGTTAACATAAATGCTCTAATTGCTTCAAGCATTGAAGAGATAC

                        16661
DS571152_90156-108875   TCCAAACAGGAGTCCATGTTTCTGGATGAAATGAAGTAATAGAAAGACATATTTTTGTGTTAATAGCA
Ed_consensus            TCCAAACAGGAGTCCATGTTTCTGGATGAAATGAAGTAATAGAAAGACATATCTTTGTATTAATAGCA

                        16729
DS571152_90156-108875   AATCTACCATTTGGAGTAAAGAAATATACTTCAGGTGGATTTTTTGGATAGTCATAAGGGAAAACAAA
Ed_consensus            AATCTACCATTTGGAGTAAAGAAATATACTTCAGGAGGATTTTTTGGATAATCATAAGGGAAAATAAA

                        16797
DS571152_90156-108875   TTTACCATGATATATTCCTTCAGCAAAGTCTGTTTCACTTTGTCCTTTGAAACTAAAATGACATTCAT
Ed_consensus            TTTACCATGATATATTCCTTCAGCAAAATCTGTTTCACTTGGTCCTTTGAAACTAAAATGACATTCAT

                        16865
DS571152_90156-108875   GCACAGATGATTCAATCAAATAAATAGTCATGTCTGGTGGTGGTGATTTTAAGAAAGCAGCATATTCT
Ed_consensus            ATACTGAAGATTCAATCAAATAAATAGTCATATCTGGTGGTGGTGATTTTAAAAAAGCAGCATATTCT

                        16933
DS571152_90156-108875   CTTTGAAGTCGTTTAACACTATTCAAATTACTGGTTTGTTGCATtgttattatttttatg---ttttt
Ed_consensus            CGTTGAAGTCGTTTAATACTACCTAAATTATTGGTTTTTTGCATTGTTTTTGTTTTTATTTTCTTTTT

                        17001
DS571152_90156-108875   ttttctaatttgttagtttaacattttttttatataaatttccttttttttttttgttttaaaaaataaaa
Ed_consensus            TTTTTCAGTTTATTAGTTTAACAATTTT-------------TTTTTTTTTTT-------AAAATAAAA

                        17069
DS571152_90156-108875   ttgttaaagaactaattaaaattgtgttatttttttattacaaaagaATGTGGGTTATTCTTGTTTTA
Ed_consensus            TTGTTAAAGAACTAATTAAATTTGTGTTATTTTTTTATTACAAAAGAATGTGGGCTATTCTTGTTTTA

                        17137
DS571152_90156-108875   TTAGTGGCAATTCTCTCTATTATTATTTTATTGTTAATGTTTCAATTTATTGATCCATTGAAAACAAA
Ed_consensus            TTAGTGGCAATTCTTTCTATTATAATTTTATTATTAATGTTTCAATTTATTGATCCATTAAAAACTAA

                        17205
DS571152_90156-108875   ATGGTATGTAACATTCTTTGTTTTTCTTGGTTGGGGAATGTCATTTGCTATTCCTATTTTATTACCAA
Ed_consensus            ATGGTATGTAACATTCTTTGTTTTTCTTGGTTGGGGAATGTCGTTTGCTATTCCTATTTTATTACCAA

                        17273
DS571152_90156-108875   TTGATATTTCATCAgtttgatttatttcatttaaattattattaattaatattcattcatctaatgta
Ed_consensus            TTGACATTTCATCAGTTTGTTTTATTTTATTTATATTATCACTATTTAATATTAAATTATCTAATTTA

                        17341
DS571152_90156-108875   gTCTTTATTTGATAAATGCGTGGAACAACAAAATAAAGTTTGTGATGAGCCATTTACATATGTTGATA
Ed_consensus            GTCTTTATTTGATAAATGTGTAGAACAACAAAATAAAGTTTGTGAAGAACCATTTACATATATTGATA

                        17409
DS571152_90156-108875   AAAAAACTTTAGTTGTATTATGGAATTTATTATATTGGGGAACAACCATATTATGTTGGACAGCCATT
Ed_consensus            AAAAAACATTAGTTATATTATGGAATTTATTATATTGGGGAACAACTATATTATGTTGGACAGCCATT

                        17477
DS571152_90156-108875   CCATTTTTACAATCATATTGTTCAGCAGGAGATTTTCATGTATTAGAACGAATAAAAACATCATTACG
Ed_consensus            CCATTTTTACAATCATATTGTTCAGCAGGAGATTTTCATATATTAGAACGAATAAAAACATCATTACG

                        17545
DS571152_90156-108875   TGAAAATATAATATTTTATTTGGTTGTAGGATTTGTTTGTGGAGTATTTTTGATATTATTCTTAATTT
Ed_consensus            TGAAAATATAATATTTTATTTAGTTGTAGGATTTGTCTGTGGTATATTTTTGATATTATTCTTAATTT
```

```
                  17613
DS571152_90156-108875  GGAATGAAAATGGAGATTGGTTAGGTATTGCTATTGCAGCAGCAAATGCATGGGGATTAATTATGGTT
Ed_consensus           GGAATGAAAATGGGGATTGGTTAGGTATTGCTATTGCAGCAGCAAATGCATGGGGATTAATTATGGTT

                  17681
DS571152_90156-108875  ATTGGAATGATGGGGTATGGTATTGTAGCTGTTCCAGCTCGATTAATTAAGAACATATCATCAAAACA
Ed_consensus           ATTGGAATGATGGGGTATGGTATTGTAGCAGTTCCAGCTCGATTAATTAAAAATATATCATTAAAACA

                  17749
DS571152_90156-108875  TTATTTAAATTCATTATATTCTGAAATTAATGATCTTACAGAAGAACATGAAGAAGAGGAAGGAATAT
Ed_consensus           TTATTTAAATTCATTATATTCTGATATTAATGATCTTACAGAAGAACATGAAGAAGAAGAAGGAATAT

                  17817
DS571152_90156-108875  TATCAGAACTTATTACTCTTGTTAAGAAAGCAGATGAAATTATTCCAATTACTGACCCAATGAGGAAA
Ed_consensus           TATCAGAACTTATTACTCTTGTTAAAAAAGCAGATGAAATTATTCCAATTACAGACCCAATGAGGAAA

                  17885
DS571152_90156-108875  TGTGTTCTTATAATTATAAATGAAATTGACCCAAAACGTTATGAAGCAACAGAACCATCAAGAGACTT
Ed_consensus           TGTGTTCTTATAATTATAAATGAAATTGATCCAAAACGTTATGAAGCAACAGAACCATCTAGAGATTT

                  17953
DS571152_90156-108875  TGTTAAGTCATATGAAAATCTTAGTGAATTACATGCAAATATTCAATTTCAACAATTAAAAGTTAAAC
Ed_consensus           TGTTAAATCATATGAAAATCTTAGTGAATTACATGCAAATATTCAATTTCAACAATTAAAAGTTAAAC

                  18021
DS571152_90156-108875  AAATATTTTACACATTACATAGTAATGTAGATCAAGTATTAAAATATGAAAGTATTAATAATAAAAAA
Ed_consensus           AAATATTTTATACTTTACATAGTAATGTTGATCAAGTATTAAAATATGAAAATATTAATAGTAACAAA

                  18089
DS571152_90156-108875  GCTCCTTTATTTCAAAGAATTTATTTCAATGTAATCAAAAAAATAGTTTCAGTCATTGCATTAATTTT
Ed_consensus           GCTCCTTTATTTCAAAGAATTTATTTTAATATAATCAAAAAAATAGTTTCAATTATTGCATTAATTTT

                  18157
DS571152_90156-108875  ATTTATTATTTATTCTTTAACAGTCTTTTCAAGTGAATTATTACTTCCATTCAATCTCCCTATATTAT
Ed_consensus           ATTTATTATTTATTCATTAACAGTATTTTCAAGTGAATTATTACTTCCATTTAATCTTCCTATATTAT

                  18225
DS571152_90156-108875  CTCCTCTTTATTACATTATTCAATCAATTGAATCATCTGCATTTCTTTTACTTATTGTAATTACTGTT
Ed_consensus           CTCCTCTTTATTATATTATTCAATCAATAGAATCATCTGCATTTCTTTTACTTATTGTAATTACTGTT

                  18293
DS571152_90156-108875  TTTGTTATTTATATTGCTTGGTGTGTTTATCAAACATTAATATCAATGAAATTATTTGATTATTATCA
Ed_consensus           TTTGTTATTTATATTGCTTGGTGTGTTTATCAAACATTAATATCAATGAAATTATTTGATTATTATCA

                  18361
DS571152_90156-108875  ATTATTTAATAATCGTTTATCTGATCCTGGGTCAATGCTTTTTTCTGCAGCATATTTATGTCGTTTAT
Ed_consensus           ATTATTTAATAATCGTTTATCTGATCCTGGTTCAATGCTTTTTTCTGCAGCATATTTATGTCGTTTAT

                  18429
DS571152_90156-108875  GTGCACCTTTAGCATTAAATATTCTTCATATGATCAAATTTGATGGCGTTCATTTCAATGGGACACAA
Ed_consensus           GTGCTCCATTAGCATTAAATATTCTTCATATGATTAAATTTGATGGTATTCATTTCAATGGAACACAA

                  18497
DS571152_90156-108875  ACTGCTTTTCAATCAGTAATGTCTTCAATGGAAGATATTCCATTCTTTGGTCAAAATAGTTTCAATGA
Ed_consensus           ACTGCTTTTCAGTCAGTAATGTCTTCAATGGAAGATATTCCATTCTTTGGTCAAAATAGTTTCAATGA

                  18565
DS571152_90156-108875  TTTCTTCCCTGTTTGTATTGTTATTGTTTCTGCATTCAGTTTGTTGAATCATTTTATTCCAATTCCTT
Ed_consensus           TTTCTTCCCTGTTTGTATTGTAATTGTTTCTGCATTTAGTTTGTTGAACCATTTTCATTCCAATTCCTT

                  18633
DS571152_90156-108875  TGATTTTCAATTCAATTTTGAAATTTTTTGGAGTTAGAGACCATAATAAACTAACTACTGCACAAAAA
Ed_consensus           TAATTTTCAATTCAATTTTGAAATTCTTTGGTGTTAGAGATCATAATAAACTAACTACTGCTCAAAAA

                  18701
DS571152_90156-108875  ATAAAAAGAGGAAAATACATTTCTCAAATATTCTACACTCGATATGCTGATTATATGTTTACTTTAAA
Ed_consensus           ATAAAAAGAGGTAAATACATTTCTCAAATATTTTACACTCGATATGCTGATTATATGTTTACTTTAAA

                  18769
DS571152_90156-108875  TGCAAGTACTCATTTATTAAAACAAAAAGATGATTAATaatttattatatctttca-ttttatggttt
Ed_consensus           TGCAAGTACTCATTTATTAAAACAAAAAGATGATTAATTTTTTATTATATCTTTCATTTTTTTGGTTT

                  18837
DS571152_90156-108875  aatgatatttttattttattttttatttcttatcttatcttattgttttgatttgtatttcatcaact
Ed_consensus           AATCAGATTTTTAT----TCTTCTATT-----TCCTATCTTATTGTTTTGATTTCTAATTAATCAAGT

                  18905
DS571152_90156-108875  gt--------
Ed_consensus           ATTTTTATAT
```
