# Supplementary material for: Evidence of Gene Conversion in Genes Encoding the Gal/GalNac Lectin Complex of Entamoeba
Source: PLoS Negl Trop Dis. 2011 Jun 28;5(6):e1209. doi: 10.1371/journal.pntd.0001209 (PMC3125142; doi:10.1371/journal.pntd.0001209)
Supplement: Figure S7 — Nucleotide alignment of orthologous genome regions of E. histolytica and E. dispar used to estimate inter-specific divergence around the light chain lectin orthologues EHI_159870 and EDI_325130. (PDF) [file pntd.0001209.s007.pdf]

```
Alignment: /Users/garethweedall/Desktop/lgl2.fas
Seaview [blocks=70 fontsize=10 A4] on Fri Nov  5 15:24:29 2010

                            1
DS571167_92206-110299       taatacttttatactaaggtgttgaa-atattctttacatgaaaaaaagaattgtattatagagaa
DS548606_16670-34840_rc     --at-cttttatacccatttcttcaataca---attaccttaaaatgaaagtagtttgacagatag

                           67
DS571167_92206-110299       atgtatt-----aaaagagtataaataaaaaagaggtaatTTAATCTTCTTTCTTATCTTCTGGTA
DS548606_16670-34840_rc     aaatatctcttaaaaagagtatgaataaaaaagaggtaacTTAATTTTCCTTCTTGTCTTCTGGTG

                          133
DS571167_92206-110299       TTGATGTAGGAATTTCTTTATGACCTTCACAACA---------------ATTACATTCACTTTCTG
DS548606_16670-34840_rc     TTGAAGTAGGAATTTCTTTATGACCTTCACAACAATTACATTCATTGCAATTACATTCACTTTCTA

                          199
DS571167_92206-110299       ATGTGGTATGGTT------------------------GTGGTCATGTTGTTGACAACTACAACAAT
DS548606_16670-34840_rc     ATGTAGTGTGATTGTTCTCATTACAAGAACATTGTTTGTGGTCATGTTGTTGACAACCAGAACAAT

                          265
DS571167_92206-110299       TTGATGGATGTTCAGGTTCAGCATCAGTAATTGCGAAACAAGAATGTGCACGAATAATTATAACAG
DS548606_16670-34840_rc     TTGATGGGTGTTCAGGTTCAGCATCAGTAATTGCGAAACAAGAGTGTGCACGAATAATTATAACAG

                          331
DS571167_92206-110299       CAATTACTAAAGGAAGAAGGAATGGTAAGAAACAATCATTGAAATAAGCAGTAATGGAAGCAGAAA
DS548606_16670-34840_rc     CGATTACTAAAGGAAGAAGGAACGATAAGAAACAATCGTTAAAATAAGCAGTAATAGAAGCAGAAA

                          397
DS571167_92206-110299       TATACCCGATACAAATAGCTATGAAAGAAGGAGGGAATTCTTGAGGAAGGTTTATAGGAATAGAAA
DS548606_16670-34840_rc     CATATCCGATGCAAATGGCTATGAAAGAAGGAGGGAATTCTTGAGGAAGGTTTATAGGGATAGAGA

                          463
DS571167_92206-110299       TATTTAATTTAGTACAGACTTTGTATATGACTATTGGAATAGCTTGCATTAATAAAAGAAGAAGAG
DS548606_16670-34840_rc     TATTTAATTTAGTACATACTTTGTAAATGATTATTGGAATAGCTTGCATTAATAAAAGAAGAAAAG

                          529
DS571167_92206-110299       TGAAAGGCTTGATTACActaaaattattatgagcattatacttttaaaag--aataataatgagtc
DS548606_16670-34840_rc     TGAAAGGTTTGAATAAActaaaattattacgaatactacactattaaaaaataataacaataagcc

                          595
DS571167_92206-110299       aacaaacATTCCAATAAATACACAAATACCACAAAGCATAACATAAAGAAATCTGAAATTATAAAT
DS548606_16670-34840_rc     aacaaacATTCCAATGAACACACAAAGACCGCAAAGCATAACATAAAGAAATCTGAAATTATAAAT

                          661
DS571167_92206-110299       ATTAACTTTTGTTCTTAAATTCATtatagcaacatcactgaatattccatattttgtttgttttaa
DS548606_16670-34840_rc     ATTAACTTTTGTTCTTAAATTCATTATAGCAGTATCACTGAACATTCCATATTTTGTTTGTTTTAA

                          727
DS571167_92206-110299       taaatctaattcataataaacttcttgcataaaagagtttattgaagttttaatatcttccatttt
DS548606_16670-34840_rc     TAAATCTAATTCATAATAAACTTCCTGCATAAAAGAATCCATTGAAGTTCTGATATCTTCCATttt

                          793
DS571167_92206-110299       ggaatgaatgttttttttttga-ttttttatttttttgatttagattctcaataatgaaaagatta
DS548606_16670-34840_rc     gaaatgaatggtttttttttgatttttttactttttttgatttagattatcaataatgaaaagatta

                          859
DS571167_92206-110299       aactaaa---taatatcgtttcattcttatcacaatTAATGCAAACAATAACTTGGAAACAAGGTC
DS548606_16670-34840_rc     aactaaaaaata-tatcgcttcattcttatcctaatttATGCAAACAATTACTTGGAAACAAGGTC

                          925
DS571167_92206-110299       TTGAAGAATTAAAAGGAATGTTCCCAAATATTGACTCAAAATTAATAGAACAAACCTTACGTGATA
DS548606_16670-34840_rc     TTGAAGAATTAAAAGGAATGTTCCCAAATATTGATTCAAAACTAATAGAACAAACGTTACGTGATA

                          991
DS571167_92206-110299       ATGGTGGACATATGGAACAAACGGTTGGTGTCTTGTTAAGTTTCACAGATGATACAATACCTACAC
DS548606_16670-34840_rc     ATGGTGGACATATGGAACAAACGATTGATATCTTGTTAAGTTTCACAGATAAT---ACACCTACAC

                         1057
DS571167_92206-110299       AAAATCAAAAgtttgtctattaattgaatggttttaacgcatcaagaaatgtaagttcatattatt
DS548606_16670-34840_rc     AAAATCAAAAgtttgtctattagttaaacagttttaatatatcaaaaaac-taaattcatgttatt

                         1123
DS571167_92206-110299       agCCATGAAGAATTTATTAGTACAAGCAATGATGATATTAGTGACGAAGAATTAGCTCGACGTATT
DS548606_16670-34840_rc     agTGATGAAGAAATTATTAATACAAATAAAGATGAGATTAGTGATGAAGAGTTAGCTCAACGTATT

                         1189
DS571167_92206-110299       CAAGAACATGAATTTAATATGTATAAATCATTAGGAGGAAGAAAGGCTATGGATCAATTTTTAAAA
DS548606_16670-34840_rc     CAACAACATGAATTTAATATGTACAAATCATTAGGAGGAAGAAAAACTATGGACCAATTTTTAAAA
```

```
                          1255
DS571167_92206-110299     GACTTAGAAAACCCAAACCATTCTACTCCCAAACACAATCAAGAAGTTAAATCTTTTTGGAACCAA
DS548606_16670-34840_rc   GATTTAGAGAATCCAAATCATTCTAATTCTAAACAAAATCAAGAAGTTAAATCTTTTTGGAATCAA

                          1321
DS571167_92206-110299     TTAAGTGCTAGTGCACAAAATGTATTTAAAGAAATGTTTAAAACAAAGGGTCAGTATCAgtttgtt
DS548606_16670-34840_rc   CTAAGTACAAGTGCACAAAATGTATTTAAAGAAATGTTCAAAACAAAAGGTCAGTATCAgtttgtt

                          1387
DS571167_92206-110299     ttacattatatattttagaataaagtctctgtat--ttctaattattttagAGAATTACCTGAAAA
DS548606_16670-34840_rc   ttacatt-t-tattttagagtaaaatctctatattattctaactattttagAGAATTACCTGAAAA

                          1453
DS571167_92206-110299     TGAACCAACTAAAGAAGAGTgtttgtttatgattattctttttgttttgatttagtagttttagaa
DS548606_16670-34840_rc   TGAGCCAACTGAAGAAGAATgtttgtttattattattctttttgttttagtgtaagaattttagaa

                          1519
DS571167_92206-110299     taaaatgtttattgatgttagCTAAAAAAGATCGGTAAGATAATATTTCTCTTTTATA-TTACTTA
DS548606_16670-34840_rc   taaaatgcttatgagtgatagCTAAGAAAGACCGATAAgataatatttctcttccatatttactta

                          1585
DS571167_92206-110299     AATACAATTTATTTTTCACTtcaacatgttaatttttatgttctttcatcaaaaaagaaaatgaaa
DS548606_16670-34840_rc   aatacaatttaattttcacttcaacttgttaatttttatgttctttcatcaaaaaagaaaataaaa

                          1651
DS571167_92206-110299     aatctccaaaaataaaaag-aaaaaagaacttaaaattattttataataaagggaaagagtATGAG
DS548606_16670-34840_rc   aatctccaaaaataaaaagaaaaaaagaacttaaaattattttataataaagagaaagagtATGAG

                          1717
DS571167_92206-110299     TCAAAGGAATCGTACAACACTAAGAGATAACCCAACTAGTTCAAAGAAGTTAATGTCAAAAATAGA
DS548606_16670-34840_rc   TCAAAGGAATCGTACAACATTAAGAGATAATCCAAGTGGTTCAAAGAAGTTAATGTCAAAAATAGA

                          1783
DS571167_92206-110299     AGAGAAAGAGGATATTGGATTTTTTGTAGGAAGTGATTATACAAATTTTATTAAATCAAGATTTAA
DS548606_16670-34840_rc   AGAGAAAGAAGACATTGGATTTTTTGTAGGAAGTGATTATACAAATTTTATTAAATCAAGATTTAA

                          1849
DS571167_92206-110299     GACAGAAGAAGCCATTTGTTGTGTATTTGCATTAAAAGAAAAAGTAATAGTAGGTTCAACAGTAGG
DS548606_16670-34840_rc   GACAGAAGAATCCATTTGTTGTGTATTTGCATTAAAAGAAAAAGTAATAGTAGGGTCAACAGTAGG

                          1915
DS571167_92206-110299     AAGAGTTTATTTTTTTAATAAAGGGGAAGAGGATATTGGAGGAGAAGGTATTTTTAATTCAACACC
DS548606_16670-34840_rc   AAGAGTTTATTTCTTTAATAAAGGAGAAGAAGATATTGGAGGAGAAGGTATTTTTAATTCAACTCC

                          1981
DS571167_92206-110299     AGTTCTTGGATTTGCATTAGACTCACGTGGACATGTTTTAGTTATTCAAGAGAATAATATTCATGG
DS548606_16670-34840_rc   AGTTCTTGGATTTGCATTAGACTCACGTGGACATGTTTTAGTTATTCAAGAGAATAATATTCATGG

                          2047
DS571167_92206-110299     AATTGATCCAAATGATGGAAGAATAGAAATGAATTTAGTTTCATTTACAGACGAACAAGTTACAAA
DS548606_16670-34840_rc   AATTGATCCAAATGATGGAAGAATTGAAATGAATTTAGTTTCATTTACTGATGAACAAGTTACAAA

                          2113
DS571167_92206-110299     TACAACATTTGATCAACAAAATCCATTACAGTATTGTTGTGTTATTAAACATTTAAAAACTAAACA
DS548606_16670-34840_rc   TACAACATTTGATCAACAAAACCCATTACAATATTGTTGTGTTATTAAACATTTAAAAACTAAACA

                          2179
DS571167_92206-110299     AATTACTTTAAAAATTACATTAATTAGAAGTCCAGGAGAACGACCAATTATATTAACTGATAGTTT
DS548606_16670-34840_rc   AATTACTTTAAAAATTACATTAATTAGAAGTCCAGGAGAACGACCGACCATATTAACTGATAGTTT

                          2245
DS571167_92206-110299     ACAAAGAAGTTATATTAATCAAATGAAATGGATTGGAAATACTATTGTAATTGTTATGTCAAATAG
DS548606_16670-34840_rc   ACAAAGAAGTTATATTAATCAAATGAAATGGATTGGAAATACTGTTGTAATTGTTATGTCAAATAG

                          2311
DS571167_92206-110299     GAGTGGAGATGGAATGAATAGAGATATCATTGTTTATTTTTATGATATTAATGCTAGACAACATTC
DS548606_16670-34840_rc   AAGTGGGGATGGAATGAATAGAGATATTATTGTTTATTTTTATGATATTAATGCTAGACAACATTC

                          2377
DS571167_92206-110299     AATTATACCTTTTAATAATATTGAAGTTAAAAGTAGTACTTCTTGTTGTGTTTCACCATTAAATAA
DS548606_16670-34840_rc   AATTATACCTTTTAATAATATTGAAGTTAAAAGTAGTACTTCTTGTTGTGTTTCACCATTAAATAA

                          2443
DS571167_92206-110299     AGAAGTTATGGTTTTAACATGGGGAAATTTAAAAGGAGAATTTCGTGCAGTTCCTGGATATGATGT
DS548606_16670-34840_rc   AGAGGTAATGGTTTTAACATGGGGAAATTTAAAAGGAGAATTTCGTGCAGTTCCTGGATATGATGT

                          2509
DS571167_92206-110299     TTCAACTCCAAAAACTTTTAATGCACCAACAGCATTTTTTGTTCCACGAGGAGTTAATGAAATTTA
DS548606_16670-34840_rc   TTCAACTCCAAAAACTTTTAATGCACCAACTGCATTTTTTGTTCCACGAGGAGTTAATGAAATTTA
```

```
                              2575
DS571167_92206-110299        TAGTCTTGGGTTATTAAATGATAAAATGGTAGGATTTGTAAATTGTAAAGATCAAGGAGCAAAATA
DS548606_16670-34840_rc      TAGTCTTGGATTGTTAAATGATAAAATGGTAGGATTTGTAAATTGTAAAGATCAAGGAGCAAAATA

                              2641
DS571167_92206-110299        TGGACTTAAAGTTCTTGTAAGTAATGATGAACGAGATAAAAAAGCAACATTTTCATTAATGCCAAA
DS548606_16670-34840_rc      TGGACTTAAAGTTCTTGTAAGCAATGATGAACGAGATAAAAAAGCAACATTTTCATTGATGCCAAA

                              2707
DS571167_92206-110299        AGAAGATATTAGTGAATATTTTGAATTATATTCAGATAGAACAGAAGAAGAAGAATATCTTTGGTT
DS548606_16670-34840_rc      AGAAGATATTAGTGAATATTTTGAGTTATATTCTGATAGAACAGAAGAAGAAGAATATCTTTGGTT

                              2773
DS571167_92206-110299        ATTAACAGAAAGAAAATTTATGAAAGTTAAAATGTTACAAGATAGAGATATTATTGATATATGGAA
DS548606_16670-34840_rc      ATTAACAGAAAGGAAATTTATGAAAGTTAAAATGTTACAAGATAGAGATATTATTGATATATGGAA

                              2839
DS571167_92206-110299        AGAACAAAAAAGATATGATTTAATTCTTGAAAGAGTTATTAAAAAAGATATGGATATTGATAAAGA
DS548606_16670-34840_rc      AGAACAAAAAAGATATGATCTAATTCTTGAAAGAGTTATTAAAAAAGATATGGATATTGATAAAGA

                              2905
DS571167_92206-110299        AGAATTAATTGAGTATTGGCAAGATTTAATCTTATCAGTTATGGATGGAGCAGTAACTGAAGTAGA
DS548606_16670-34840_rc      AGAATTAATTGAGTATTGGCAAGATTTAATCTTATCAGTTATGGATGGGGCAGTAACTGAGGTAGA

                              2971
DS571167_92206-110299        AGAACCATATACCTTAATGGAAGAATATTTAGAATCAGATTTAATGGAATTAGAAGATTTCTTAGA
DS548606_16670-34840_rc      AGAACCATATACCTTAATGGAAGAATACTTAGAATCAGATTCAATGGAATTAGAAGATTTCTTAGA

                              3037
DS571167_92206-110299        AATTACTCAAAACTTTCATATTGAACCAAAGGAATTTAGAGAAAAGGATAGTAAAGAAGCAAGAGA
DS548606_16670-34840_rc      AATTACTCAAAACTTTCATATTGAACCAAAAGAGTTTAGAGAAAAAGATAGTAAAGAAGCAAGGGA

                              3103
DS571167_92206-110299        ATATGAAAAATTAAAAGTCCAAATGAAAGATAGTTTGAATAAAACATTATTATTCTTTACTAATTA
DS548606_16670-34840_rc      ATATGAAAAATTAAAAGTCCAAATGAAAGATGGTTTGAATAAAACATTATTATTCTTTACTAATTA

                              3169
DS571167_92206-110299        TACTATTTTCTCATTAAAAAAGAATGAACAAAGATATTATGAATTAATTAGTACATACAATATATT
DS548606_16670-34840_rc      CACGATTTTCTCATTAAAAAAAAATGAACAAAGATATTATGAATTAGTTAGTACATATAACACATT

                              3235
DS571167_92206-110299        TTACAATAATAAAGATAGAAATTATGCATATGAAGATGCATATAATACATTAGTTGCATACGCTAA
DS548606_16670-34840_rc      TTATAATAATAGAGATAGAAATTACGCATATGAAGACGCATACAATACATTAGTTACATACTCTAA

                              3301
DS571167_92206-110299        ATTAAAAGAAACAATTCAAAGAGTAAAAAGTGTAGATAAATATGATAAAATTCCACAAGATGAATT
DS548606_16670-34840_rc      ATTAAAAGAAACAATTCAAAGAGTAAAAAGTGTAGATAAATATGATAAAATTCCACAAGATGAATT

                              3367
DS571167_92206-110299        GTTAGGATTAATGAGAAAAGAATTAAATAAATATAAAACATTACTTCTTTATCCAAATTTACCAAC
DS548606_16670-34840_rc      ATTAGGATTAATGAGAAAAGAATTAAACAAATATAAAACATTACTTCTTTATCCAAATTTACCAAC

                              3433
DS571167_92206-110299        AAACTTTCAAGAAATTATATCAAATAATACTATCGGGTCAAAATTTGATGGAAGAAATGAAGATAG
DS548606_16670-34840_rc      AAATTTTCAAGAAATTATATCAAATAACACAATTGGATCAAAATTTGATGGAAGAAATGAAGATAG

                              3499
DS571167_92206-110299        AATTCGATTTCAAAAATTTAATATGCATTATAAAATAGAAAATTGTATGTTTGAAGAAGCACAAAA
DS548606_16670-34840_rc      AATTCGATTTCAAAAATTTAATATGCATTATAAAATAGAAAATTGTATGTTTGAAGAAGCACAAAA

                              3565
DS571167_92206-110299        TTTAATTTTTAAGTTAAAAGACCCAAATTTATTTAAAATGTTTTTAGAAAAGCTTCAATCAATTTA
DS548606_16670-34840_rc      TTTAATTTTTAAATTAAAAGACCCAAATTTATTTAAAATGTTTTTAGAAAAGCTTCAATCAATTTA

                              3631
DS571167_92206-110299        TGACTTACATATGCAAAATATTATTGAAAAAGAAAAACAAGAAATAGAAACTAAAAAAGTATTTGA
DS548606_16670-34840_rc      TGATTTACATATGCAAAATATTATTGAAAAAGAAAAACAAGAAATGGAAATTAAAAAAGTATTTGA

                              3697
DS571167_92206-110299        AAAAGAAGGATATAACCTTAAAAATCTTAGTGATGTTTATGTAAATGAAGTAGAAGAAGTTATGAA
DS548606_16670-34840_rc      AAAAGAAGGATATAATCTTAAAAACCTTAGTGATGTTTATGTAAATGAAGTAGGAGAAGTTATGAA

                              3763
DS571167_92206-110299        AAAAGCAGAGTCAAATCCATTATTACAATTTAGATTATATCAATTATTATTTAAATTATTAAGTGA
DS548606_16670-34840_rc      AAAAGCAGAATCAAATCCATTATTACAATTTAGATTATATCAACTATTATTTAAATTATTAAGTGA

                              3829
DS571167_92206-110299        AAAAAAATATCCTATTGATAATTATGAATCTAGAGTAGGTGGTGGTATTCCTAAATTATTATTTGA
DS548606_16670-34840_rc      AAAAAAATATCCTATTGATAATTATGAATCTAGAGTCGGTGGTGGGATTCCTAAATTATTATTTGA
```

```
                              3895
DS571167_92206-110299         AGCTACAAAAGGAAGTAAAAATACTCATGAAATGCAACAAATTGTTATTGCTTTTATTTATTCTAC
DS548606_16670-34840_rc       AGCTACAAAAGGAAGTAAAAATACTCATGAAATGCAACAAATTGTGATTGCTTTTATTTATTCTAC

                              3961
DS571167_92206-110299         AATTAATGATTATAATAATTTATTTGACTTTAGAAAAAATGAAAAAGCTGTTGAATTAATTACAAG
DS548606_16670-34840_rc       AATTAGTGATTATAATAATTTATTCGATTTTAGAAAAAATGAAAAAGCTGTTGAATTAATTACAAG

                              4027
DS571167_92206-110299         TGTTATGGAAATTGTTAATTCTTTAGAAGTCTCTATTTCTCGTGAAGTTTTAAAAGCTCAAAATTA
DS548606_16670-34840_rc       TGTTATGGAAATCGTTAATTCTTTAGAAGTCTCTATTTCTCGTGAAGTTTTAAAAGCTCAAAATTA

                              4093
DS571167_92206-110299         TGGAAATAAAAAGTGGATTATTACTTTATTATATGAATTGTCAGTTTATAAAGATCAATTATTAAA
DS548606_16670-34840_rc       TGGAAATAAAAAATGGATTATTACTTTATTGTATGAATTATCTGTTTATAAAGATCAATTATTAAA

                              4159
DS571167_92206-110299         TGTATTAGATGAAGACGAAACAATGGAAACTTTTGCTCAACCTGTTTGTGATGGTTTAAGAAATGC
DS548606_16670-34840_rc       TGTCTTAGATGAAGATGAAACAATGGAAACTTTTGCTCAACCTGTTTGTGATGGTTTAAGAAATGC

                              4225
DS571167_92206-110299         TTCTCTAAGATATGAAGCTTTATTATCTGCTCAACACATTGCTCATCAACAACAATTTGATTATGT
DS548606_16670-34840_rc       TTCTTTAAGATATGAAGCCTTATTATCTGCTCAACACATTGCCCATCAACAACAATTTGATTATGT

                              4291
DS571167_92206-110299         CAATAGTATTATTGACTATTCTAAACAAGGAATAATAGTAGAAGAAGTCGAAGGAAAATGTTGTAG
DS548606_16670-34840_rc       CAATAGTATTATCGATAATTCTAAAAAAGGAATAATAATAGAAGATATAGAAGGAAAGTGTTGTAG

                              4357
DS571167_92206-110299         TTGTAATAGAACTGTTGTAAAAAGAACTATATTTAAATGTGGTCATTGTATGTGTGATCAGTGTTT
DS548606_16670-34840_rc       TTGTAATAGGTCTGTTGTAAAAAGAACTTTATTTAAATGTGGTCATTGTATGTGTGATCAGTGTTT

                              4423
DS571167_92206-110299         GGAAAAACTTACCACTAAAGGAACAAATATTGAATGTTCTGTTTGTAAGAGTCAAAGATTAACAGA
DS548606_16670-34840_rc       GGAAAAACTTACTGCTAAAGGAGCAAATATTGAATGTTCTGTTTGTAAGAATCAAAGATTAACAGA

                              4489
DS571167_92206-110299         CCAACAACAAAAATCTCTTTCTTTTATTCAAACTCCTCGTCATAAAAAGAAGTAAtttattatttc
DS548606_16670-34840_rc       ACAAAAACAAAAATCTCTTTCTTCTATTCAAACCCCTCATCATAAAAAGAAGTAAtttattatttc

                              4555
DS571167_92206-110299         atttatttcttta---ttataaattcagttaatttaaaaagaataattaatacgtaagaaatatat
DS548606_16670-34840_rc       a-ttatttttttatattaacaaaatcaattaattaaaaaggaataa-taataagtaataaatatat

                              4621
DS571167_92206-110299         ttacgattgtattattttcaagtgctaagttgtttatttctctatt----tatg--ttagttgtta
DS548606_16670-34840_rc       ttattattgtattattttctaatgtttaattgtttatttttctttgtttttatgtgtttaatatta

                              4687
DS571167_92206-110299         caaaaattatttttcaaaagttttatgaaatataacattcaatttattagttagtattattcattg
DS548606_16670-34840_rc       caaaaattatcttttaaatattttattaaa-ataaaattcaatttatttgttagtgttatgtattt

                              4753
DS571167_92206-110299         tttgttcaaactattttattcaacagatgataaatatatattctttgtactgtgttactatggtta
DS548606_16670-34840_rc       gttgttcaaatt-ttttaatcaacgaatgataaatatgtcttctttgtattttgttactatggtta

                              4819
DS571167_92206-110299         ttattttacttttctttcaatttctacc-aatatgctttttgtttattgttttatacaatatttct
DS548606_16670-34840_rc       ttattttatttttctttaaacgtccactgaatgaacttttcatttatagttgtatattttatttct

                              4885
DS571167_92206-110299         ataaataaaactacaaacttattttgaaaatatgataaactttattctaaaaaaaattctaattgg
DS548606_16670-34840_rc       ataaataaaactttatattta-ctcgggagtattattctctttattctaaaagagccattagtt-g

                              4951
DS571167_92206-110299         tgtttaaatttatttt-ttttcattcaataatggattatgacatacttttatttaaaagttttatg
DS548606_16670-34840_rc       tgtttaacttcactttgttttcatgtgataccttacaatgaattattcttattt-aaagtaataag

                              5017
DS571167_92206-110299         acaaataacgtaatgtattaagaattt-gaatttat----tctacggtaaaataagaaacaag---
DS548606_16670-34840_rc       gt-atttatataatgttttaagaatttagagtttatctcatcttggttttttttagagataatatg

                              5083
DS571167_92206-110299         ------------------------aaagaataataat--------aaaagaa--aaagaaag--aa
DS548606_16670-34840_rc       ttttgtatctttctagtttatgccaatgtattataacccattttgaaaataattaatgaaagtaaa

                              5149
DS571167_92206-110299         agaaaagagaatgataaagaga-------aagagtgtcaatattaatagatgaaagatgaag----
DS548606_16670-34840_rc       aggaaataaagtgttaaagagaatagtgtaaaggttctaaaacgaataagagaattatgaaggaga
```

```
                        5215
DS571167_92206-110299     --aaatgtaaagataataaaggaataaaaaaagaataaa----------------aaaaaaataaa
DS548606_16670-34840_rc   taaaatatcacaacaattaaagaactaatatggaacagagttattcaaactatttaatagaataaa

                        5281
DS571167_92206-110299     gataaa-gaaatga---tagaagaaggaaataaataggaagagaagttcaaaaatgataattgaat
DS548606_16670-34840_rc   gaagaatggaatgaatgtaaacgtgtagaatatat---gagtgtgatagagtaatggtaatagaat

                        5347
DS571167_92206-110299     aaagt-----------------tgaagattatatgaaaataaaaaggagattaaagatatagaata
DS548606_16670-34840_rc   taaattcaatacttaaacattatgaataatacataaaattaaaaagttatacttaattatacaata

                        5413
DS571167_92206-110299     taagtattaaaaatataagaaataaatatgatagtttttaatataaatgaagaaatga---gagat
DS548606_16670-34840_rc   caagtatttaaaacatataaaccatttacatatatttttattccaaaactatcatttactcgttat

                        5479
DS571167_92206-110299     gttttat-aaatttatgagataatgattgaataaaaatg-aataaagtagaaatggtaaatcaaat
DS548606_16670-34840_rc   tttt-attaattatatatcattggtttttaataaaaatgaaatatacaagaaatgat-atttaagt

                        5545
DS571167_92206-110299     gatagtggtaatgaaggaatataaagtc-acaaaagatgaagaaaggaaataa------tcaagga
DS548606_16670-34840_rc   gataacactatcatattaatataaaaactccaaataataaagaaatagagcaatttgtttcatata

                        5611
DS571167_92206-110299     aatgaa--tattgaaaatgataagaatattcaaaac-aaaagaaacaacataatagtaatatattg
DS548606_16670-34840_rc   attgcatttattgaattcaatatcacttttcaattcaaaaagaatcaatattattttattatgttg

                        5677
DS571167_92206-110299     acaa---ca---tatgtaataattgattttcataataattctaattaatgataataagatgaaata
DS548606_16670-34840_rc   gtaataccaaattatgtactatttcattttcttcatatttatcattattattaa-atcatgaactg

                        5743
DS571167_92206-110299     tgttcaatgatg--ttaataatca-ttaaagttatcaaatagtataatacttaattatcataacaa
DS548606_16670-34840_rc   cgttcaatactgtattgttctttatttataaacactaaatattataataaccacttattaaatcaa

                        5809
DS571167_92206-110299     aaataaaaaattaagacaaaagaagaaa--taaattaagagagatgaaca-aataaaaa-cataaa
DS548606_16670-34840_rc   aaataaacaatcaaagaaaaaaaaggagattaaattaagagagatgaaaagaataaaaagcaggaa

                        5875
DS571167_92206-110299     caattaacataatattctttattgtttcatctaagaaattaagaata-tttgaattgaatattatt
DS548606_16670-34840_rc   caaataccatgacactctttattgtttcatctaagaagactagagcactttgaatgtgatattgta

                        5941
DS571167_92206-110299     ttattcattgtttaatatatatttatatatttaagactattataatg-attattaagaaattttta
DS548606_16670-34840_rc   ctagtcaatgactaa--------tccatatttaagatcattatattgtgtttttgaaaaatcccta

                        6007
DS571167_92206-110299     tatttcaacaatctaattttgatgttattttatttttgatgataatgatatataagtgttatgaga
DS548606_16670-34840_rc   tacctcaacaatccaattttcatgttattttttttt---tgataatgatttctatttgttgtgaaa

                        6073
DS571167_92206-110299     tatgaaaaaaaaacacaaaatggaaatgattttaaga--aagtggaaagtattaattatgagaaaa
DS548606_16670-34840_rc   tatgaaaaacttgt---cattggaattgattttaagaggggtagaaagcattatttatatgaaaa

                        6139
DS571167_92206-110299     tgaaTCATTTAACATAATTTGAAGGGAAAAATCCAATATTTCCTTGACATTCTCCTTGCCACCAAT
DS548606_16670-34840_rc   ttaaTTATTTAACATAATTCGAAGGGAAAAAACCAATATTCCCTTGACACTCTCCTTGCCACCAAT

                        6205
DS571167_92206-110299     CACCTTTTTTATTAAGAATTTTAATTTTATCTCCTTCTTTAAGTGAAAGTTCTGTTTCATTTTCTG
DS548606_16670-34840_rc   CACCTTTTTTATTAAGAATCTTAATTTTATCTCCTTCTTTAAGTGAGAGTTCAGTTTCATTTTCTG

                        6271
DS571167_92206-110299     CTGAATATGCAAATTGACAAACTACAACTTCTTCATGTTCTTCTTCTTTCTTCATTGATTCTTGTA
DS548606_16670-34840_rc   CGGAATATGCAAATTGACAAACTACAATTTCTTCATGGTCTTCTTCACTGTTCATTGGTTGATGTA

                        6337
DS571167_92206-110299     TCACATTTTCTATATTTTCATATTTATATTCTTTTATTTCATTATTATGTTGTTTAGTTTCATTAA
DS548606_16670-34840_rc   TCACTGTTTCTACGATTTCATCTTTACATTCTTTTAATTCATTAATACGTTGATTAGACTCATTAA

                        6403
DS571167_92206-110299     TCATTGATTGATCATTTGGAGTATATGTTGGTTGTGTATTAATATCTCCTGTATTCATGTTTCTAA
DS548606_16670-34840_rc   TCATAGATTGATCATTTGGTGTATATACTGGTTGTGTGTTGATATCACCATTACTCATGTTTCTAA

                        6469
DS571167_92206-110299     GCATTTCTGGAACTTTATCCCATGATAAATTTAAACAATTAATATATTCTGTAAATCCTAATATAA
DS548606_16670-34840_rc   GCATTTCTGGGACTTTTTCCCAAGATAGATTTAAACAATTAATATATTCTGTAAATCCTAACATGA
```

```
                               6535
DS571167_92206-110299     TAGAACTTTCAATTTTTTCATTTATCTCATTTATTCTTTCATTCAATTTACTAATATCATTAATTA
DS548606_16670-34840_rc   TAGAACGTTCTACTTTTTCATTCATCTCATCTATTCTTTCATTCAATTTATTAATATCATTAATTA

                               6601
DS571167_92206-110299     TTTCATTTCTTATTAATATATATGTTTCTTTAGATGCTTTATATTTTGCTTCAGCTTCTTTCTTTC
DS548606_16670-34840_rc   ATTCATTTCTTAATAATATATATGATTCTTTTGATGCTTTGTATTTTGCTTCTGCTTCATTAAGTC

                               6667
DS571167_92206-110299     CATTTTGTTTCTTTTGTCCTTTCTTTTTAATTTGTTCTAATTTTTCAAAATGTCTATCCATATCTA
DS548606_16670-34840_rc   CATTTTGTTTCTTCAATCCTTTCTTTTTAATTTGTTCTAATTTTTCAAAGTGTCTGTCCATATCTA

                               6733
DS571167_92206-110299     TCTTTCGATTTTTCATTATATTTATTCTTTTATAATAAACTTTCCATTCTTCAATAAATATTTTCA
DS548606_16670-34840_rc   TCTTTCTATTTTTCAGTATACTTATTCTTTTATAATAAACATTCCATTGTTCAATAAATATTTTCA

                               6799
DS571167_92206-110299     TAGGTTCTATAACTTGACAATATATTATTTCTTTAATATGTTTTCCTCCTTCTTCCATTGAAGAAA
DS548606_16670-34840_rc   TAGGTTCTATAACTTGACAATATAAAATTTCTTTAATATGATTTCCTGCATCTTCCATTGAATCAA

                               6865
DS571167_92206-110299     ATACATTAATCATTCTCTCACTTTCTTCTTTAGCTTTGCTTTTCTCCCCTGAACAATCGTGTAAAA
DS548606_16670-34840_rc   AGACATTAATCATTTTCTCACTTTCTTCTTTAGCAATGGATTTTTCTCCAGAACAATCATGTAAAA

                               6931
DS571167_92206-110299     TAAGAAATACTTCTTTTGGAATTCTAATTAATTTCTCAGCACTTGTTGTAATTTTACTTACTCGTT
DS548606_16670-34840_rc   TTCGAAATACCTCTTTTGGAAATCCAATTAAATTCTCTGCACATACTGGAATTTTACTTACTCGTT

                               6997
DS571167_92206-110299     CAATTATTTCTTTCATTTTCTTTTCTTTCTCAATTATTTCTTTATTAATGTTTTGAAATTCTTTAT
DS548606_16670-34840_rc   CAATAATTTGTTTGATTCTCTTTTCATTCTCAATAATTTGTTTATTTAATATTTTAAAATCTTTAT

                               7063
DS571167_92206-110299     CTTCACTTTTCTTACTGATTCCAACACTCGTTTTAATTTGAAATATCCCTCTTTTAATATTCATat
DS548606_16670-34840_rc   CAACTGTTTTCTTACTGATTCCTACTGTTGATTTAAAGTGAAATAACCCTCTTTTAATATTCATAT

                               7129
DS571167_92206-110299     ttctt---ct---cttttcttattatctttatatcttcttacctt-----ttcatattcacttctc
DS548606_16670-34840_rc   TTCTTAATCTTTTC--TTCTTCATatctttatatattcttaccttttcccttcacattcacttgtc

                               7195
DS571167_92206-110299     attaatcttttcagatatttgattttctgtttgttctcctttctactttttactcttttttcctgt
DS548606_16670-34840_rc   attaatcatttgagatattttaaaaagagt-----------------------------------t

                               7261
DS571167_92206-110299     gtttcttttatctttgtttttctttgatttttcttactcttactttgattattatctttctttatt
DS548606_16670-34840_rc   gtttctttcat-tcaatgttttttagttgtattctattcataatttgactgttg---------gtt

                               7327
DS571167_92206-110299     tattattt---ttttattgtgaatgattattattattataaataagaaagattaataagaa----a
DS548606_16670-34840_rc   tatcatttaaattcaaataaaattaataaatatcatgactcttatgaaag-----tgagaatatta

                               7393
DS571167_92206-110299     gattaataagaaagaaaaacatatatatt-----tgctttatttaatgttaaattggatttataaa
DS548606_16670-34840_rc   atttgatgagaacaaaaagaataaaagatgataacggttcgtttgtagttgaa--ggacttattaa

                               7459
DS571167_92206-110299     tggagattatatctcattttcctttttatctcttcatatcttcaaaatttaaaaaaaaaacttaaa
DS548606_16670-34840_rc   tggag-ttttatcctaatctcatttgt-tctcttcatatcttcaaaaaaataaaaaagaactaaaa

                               7525
DS571167_92206-110299     agaaaacacagaaattaggatgtgtgattgtcccactattaatggaaataacgcaatacaatcatt
DS548606_16670-34840_rc   agaaaatataaagaataatatgtgtgattgccctactattaatggacacaatgcaatgcaatcgtt

                               7591
DS571167_92206-110299     tgtgtatcaaaatttatttcgagtttgtttatactctttcttggatgaaatatcaatttgggcaag
DS548606_16670-34840_rc   tgtgtatcaaaatttgtttcgagtttgtttgtactctgtctttgatgaaatatcaatttgggcagg

                               7657
DS571167_92206-110299     ttatataaaattggtattaaacattctattaattggtaattcattgttgaaactatttaatcaaca
DS548606_16670-34840_rc   ttatataaaagtgatattaaacattttattaattggaaactatttgtctaaactaagtaatcagca

                               7723
DS571167_92206-110299     acatgttaaaattgtacattcattcgcaatatttttctttgtaatcttaggtttgttattcaattt
DS548606_16670-34840_rc   acaagttaaacatgtgtattcagttggaatactttgtttgtagtcttgggtttgtagttcaagtt

                               7789
DS571167_92206-110299     ctttgattattataagatataaaataactgatatgaattgtctagattttcatggtataatttata
DS548606_16670-34840_rc   atttaa--atgataaaggataaaattacttatagaaaatgactagatttccatggagtgatttata
```

```
                         7855
DS571167_92206-110299     gtatttccaatggattttttgcaacacaaacactgacttgcattcttaatttcgtctctactatat
DS548606_16670-34840_rc   gcatttccaatgggttttttgcaacacaaacactcacaagcattcttaaatttgtatcaacagtgt

                         7921
DS571167_92206-110299     catactatgctattgtcatca-caaaaactagaaccaaccaatggcgtcatttattcttttccttc
DS548606_16670-34840_rc   catactatattattgtcattaacaaaa-caaagaccaaccaatggcgttatttattcataagtttc

                         7987
DS571167_92206-110299     tttctttgctttttgatatggtggtactcttctccttctcttaaatcatgctttgttaggaatctc
DS548606_16670-34840_rc   tttatttgtactttgatatggtggtattcttctccttctcttaaatcatgctttgttagacatctc

                         8053
DS571167_92206-110299     ccatcttcc-tttttctcttctatttatgtcatcatcggtgctatcttctttaacataagatcttt
DS548606_16670-34840_rc   ccaacatcactttttt-catctatttatgttgtcattggtgcaattgtatttaacataagaaacga

                         8119
DS571167_92206-110299     atttgatcttcttatttggcttgctactactaacttaaataatacctcattttcctttttatctct
DS548606_16670-34840_rc   aattgaacaaatcatttggcttttcactactaatttaaataatgtctcctttttcaacttaaatct

                         8185
DS571167_92206-110299     tcatctcttcctttatctccttaaattctttgctgtcattttttatggtgctcatccatcttcaat
DS548606_16670-34840_rc   tcatatcttcgtttatctccttaagttcgttactgtcattttttatggtgcccatccatcttctgt

                         8251
DS571167_92206-110299     gttatggtattgtctattatttatttatgtttcaggagcatttcttccattagtaatattcttgat
DS548606_16670-34840_rc   gctatggtattgtctattacttgtttatgtttcaggaacatttcttccattagttatattctttat

                         8317
DS571167_92206-110299     tatgtttttacgaaagcttctttgttactctagaactaagaaaagtcatagatatgaaaatatcac
DS548606_16670-34840_rc   tatgtatttacgaaaacttctttgttattctagaattaaaaaaaactatagatatgaaaatatcac

                         8383
DS571167_92206-110299     aagaaatgaagaggattgccatggagataaatttaaaaactattaaaaa-ttttttt---tgtttt
DS548606_16670-34840_rc   aagaaatgaagaaaattatcaaggagataaattcaagaattattagaacctttttt-ctctgtttt

                         8449
DS571167_92206-110299     tgttggaagtttaaaagaac---ttattatttcttttatgactattaaatcttgtttagtttttgg
DS548606_16670-34840_rc   tgttggaagattaaaagagtttattattattcattttatgagtatt-aatcttgtttagtttttgg

                         8515
DS571167_92206-110299     ttatcaaaa--tatttgtcttaaaagtc----aaTTAATTTTCTTCATCTTCTTCTTTTATTGCCA
DS548606_16670-34840_rc   ttatcaaaacctttttatcctaaaagttaataaaTTAATTTTCTTCATCTTCTTCTTTTATTGCCA

                         8581
DS571167_92206-110299     ATCCAAATTTTTTTCCCCACTCAAAACACTCTTCTAATTTTTCCTGTCCTGGTTGGAGACGACAAG
DS548606_16670-34840_rc   ATCCAAATTTTTTTCCCCACTCAAAACACTCTTCTAATTTTTCCTGTCCTGGCTGGAGACGACAGG

                         8647
DS571167_92206-110299     ACAATGGCTCAACTGGTTGTTTTGCTTTTAGTTGTTGAAGTCTTGCTGAAAGATTCCCAACACCTT
DS548606_16670-34840_rc   ACAATGGCTCAACTGGTTGTTTTGCTTTTAACTGTCGAAGTCTTGCTGAAAGATTCCCAACACCTT

                         8713
DS571167_92206-110299     CTCCACTCCATCCGTGTGAACCAAAACATTGAATAAATCTTTGGCAATGAATATAAGGATTTAAAT
DS548606_16670-34840_rc   CTCCACTCCATCCATGTGACCCAAAGCATTGAATAAACCTTTGACAATGAAGATAAGGATTTAAGT

                         8779
DS571167_92206-110299     TACATGCTATTGTTAATATTGGGGGAAGAGCATCTCCAACAATTGTTGGCGTCCCAAGTAATAAAC
DS548606_16670-34840_rc   TACAGGCTATTGTTAGTATTGAGGGAAGAGCATCTCCAACAATTGTTGGAGTCCCAAGTAATAAAC

                         8845
DS571167_92206-110299     CTTTACAGTCACTGAAATCTTTTAAGACATTTTCAACACTAGTATTTTCTACATTATGCATGGACA
DS548606_16670-34840_rc   CTTTACAATTATCGAAATCTTTTAAGACTTCTTCACTACTAGTATTTTCAATATTGTGCATTGATA

                         8911
DS571167_92206-110299     CCTCAACACCAACAGATTTAATTCCAGCAGCAATTGTATTTGCCATAGTTTCTGTATATCCATAAA
DS548606_16670-34840_rc   CCTCAATACCAACAGATTTAATTCCAGATGCAATTGTATTTGCCATAGTTTCTGTGTATCCATAAA

                         8977
DS571167_92206-110299     CTGACCCATAGGCAATAACAACTTTATTTTTTAATGGTGGTGGTTGAGCCCATTGACGCCAAAGTT
DS548606_16670-34840_rc   CTGATCCATAAGCAATGACAACTTTATTTTTTAATGACGGGGGTTGAGCCCATTGACGCCAAAGGT

                         9043
DS571167_92206-110299     CAACACGTTCTGGTATATAACCACGTATTATTGGACCATGACTACAACAAATAGCTTTACAGTTTT
DS548606_16670-34840_rc   CAACACGTTCTCGTATATAACCACGAAGTATTGGACCATGACTACAACAAATAGCTTTACAATTTT

                         9109
DS571167_92206-110299     TTAAATCGAATCCAGGTTGATTTTCAAGGGTTTGAATGCCTTTTAAAACAAATCGTTTAAAAGGAC
DS548606_16670-34840_rc   TTAAATCGAATCCAGGTTGATGTTCAAGCATTTGAATACCTTTTAAAACAAATCTTTTAAAAGGAC
```

```
                           9175
DS571167_92206-110299     CAAAGACTGAGTTAAAATAGTCATAACTTATTTGAAGATATTGATCTTTTCTATCAACAATTAAAT
DS548606_16670-34840_rc   CAAAGACTGAGTTGAAATAGTCATAACTTATCTGAAGATATTGATCTTTTCTATCAATAATTAAAT

                           9241
DS571167_92206-110299     CATCAAACATTCGTTCATCAGCAAAATGAGTACTAAACATATCACATGTAACTAAAACATTCATTT
DS548606_16670-34840_rc   CGTCAAACATTCGTTCATCAGCAAAATGAGTACTAAACATATCACATGTAACTAAAACATTCATTT

                           9307
DS571167_92206-110299     CTTCTATTAACGTGATCATTGTATCAGGCCAATGTAAAAATGGTTGATAAATAAACTTAAGATGAA
DS548606_16670-34840_rc   CTTCTATTAACGTGATCATTGTGTCAGGCCAATGTAAAAACGGCTGATAAATAAACTTAAGATGAA

                           9373
DS571167_92206-110299     TATCACCCAGATTTAGTTGTTTAGTTTTAACACTATTAATTGTTTTTGTATCATCTCTAATATGAC
DS548606_16670-34840_rc   TATCTCCCAGATTTAGTTGTTTAGTTTTGACAGTATTAATTGTTTTTATATCATCTCTAATGTGAC

                           9439
DS571167_92206-110299     CAATAAATTGTATATTATTTAAAGCAGCAGTAGAGGCTATTACTGTTGCGTTAGGATATCTTTTAA
DS548606_16670-34840_rc   CAATAAATTGTAGATTATTTAGAGCAGCAGTAGAAGCCACTACTGTTGCATTAGGATATCTTTTAA

                           9505
DS571167_92206-110299     GTATAGGACTTAATGAACCACTATGATCAGGTTCACTGTGGTCTACTATGATATAATCAATACAGT
DS548606_16670-34840_rc   GTATAGGACTTAATGAACCACTGTGATCAGGTTCACTATGGTCCACTATGATATAATCAATACGAT

                           9571
DS571167_92206-110299     CTTCTTTACCAATAACTTGTTCTATTTTATCTAAACACTCATTTGCAAATTCTTGCTTCACTGTTT
DS548606_16670-34840_rc   CTTCTTTACCAATAACTTGTTCTATTTTATCTAAACATTCATTTGCAAATTCTTGCTTAACTGTTT

                           9637
DS571167_92206-110299     CAAATAAAACGTTTCCTTCTTGGGTTTGAAGAAGGTATGAATTGTAAGTAGACCCATAAGGTGCAG
DS548606_16670-34840_rc   CAAACAAAACATTTCCTTTTTGAGTTTTAAGAAGGTATGAATTATAAGTAGACCCATAAGGTGCAG

                           9703
DS571167_92206-110299     GATATAATACATCATAATTCTTTAGTTCATAATCCATTACACCAACCCAATACAAACCAGGAAGTA
DS548606_16670-34840_rc   GATATAACACATCATATTTCTTTAGTTCATAATCCATTACACCTACCCAATACAAACCAGGAAGTA

                           9769
DS571167_92206-110299     CTTCTAACACTTTACTTGTCATttaatgtttgttagttcttatgttcttttttcattaagaacagc
DS548606_16670-34840_rc   CTTCTAATACTTTACTTGTCATttaatgtttgttagttctcatattcttttttcgttaagaaggac

                           9835
DS571167_92206-110299     tcaggtggagaaaacaaatgaaatgattttctcgtttcctttttggtagtttttagggtagttcaa
DS548606_16670-34840_rc   tcaagtggagaaaacatatgaaatgattttctcgtttcctttttggtagtttttagggtagttcag

                           9901
DS571167_92206-110299     tttacgatatttctatacttttaacttacataaatag--tttccttttcattttaattctcaaaat
DS548606_16670-34840_rc   ttttctatatttcgatacttttaactcacacaaatagtttttccttttcattttaatattcaaaag

                           9967
DS571167_92206-110299     ttaataaataaataaagaatatctctcaaataccgataaaataaatactaaattacagtactatga
DS548606_16670-34840_rc   ttaataaatggataaagaatatttcttaaatactgataacctaaatacttaattacagtactatga

                          10033
DS571167_92206-110299     ttttgagttatgaatgaattacattgattaaatgaaaaaagttattgaacatttattataaatttg
DS548606_16670-34840_rc   tttgaagttgtgaataaattactttgattagataaaaaaaattattgaacatttattataaatatg

                          10099
DS571167_92206-110299     gagttttttttactta-ttttaattctttgttttctgaaaagagcgttgaatgatttaaaaattaa
DS548606_16670-34840_rc   gaattatttttacttatttttaattctttgttttatgaaaagtatattaaatgattt-gaaattaa

                          10165
DS571167_92206-110299     atatctttattttataaccattcaatgaac-gaaacacaatttatttttgatagaattaataaaaa
DS548606_16670-34840_rc   acactattattttat--ttattcaatgaataaaaacacattttatttttgacagagtcaattaaag

                          10231
DS571167_92206-110299     tatctaagaaatat-tttttaatcatttagagtaa------gtttaatttatcaacataaataagc
DS548606_16670-34840_rc   tacctaaaaaatatatttttaatcaattagaataaagatatgtttagtttatcaaaatgaataagt

                          10297
DS571167_92206-110299     ctcattaaaaatatgaattacccagctatttcctattaaagtaatcatttttttagagtgtcatat
DS548606_16670-34840_rc   ttcaatgaaaacataaattagtcaaatatctcttaatacaataattg-ttttttagcttgttatat

                          10363
DS571167_92206-110299     cacttgaatattaatttaataataaataatgaggttattttattgagaaaattaattacccccatt
DS548606_16670-34840_rc   cttttgaatactaatttaagaagaaataaagaggttaatttattgagaaaattaattcttttcttt

                          10429
DS571167_92206-110299     tgtatga--atattctattttcattacgttatattttgaaatactctcttttcataaaattagaag
DS548606_16670-34840_rc   tatatacatatattccattttcattattttttaattt-aaata--ttcttttcatcaatttagaag
```

```
                                10495
DS571167_92206-110299       tgttaattctatctttcattaatgaagtaaattatcctttgtttagttctcaataattaatacaaa
DS548606_16670-34840_rc     tgttaattctatcttttaataatgaagtatgttattctttatttatttctcaataattaata-gaa

                                10561
DS571167_92206-110299       aaatattatttattgttagagtaactgtcagtaaagaaagaataaaattaatgatataattacaaa
DS548606_16670-34840_rc     aaatataatttatttttagagtaacagtcagtaaagaaagataatagt--atcataaaataacaaa

                                10627
DS571167_92206-110299       tactgaaaagtgaatagaaccaaagactgctttgtttgttgtttattttt-----------gtttt
DS548606_16670-34840_rc     tattgataagcgaataaattcaaatattattttgtttattgtttatttatagagtgtttccgtttc

                                10693
DS571167_92206-110299       atgtcaaagtaccaatagaatattttatatattaaacttgttatt---attagtctctgttcaatg
DS548606_16670-34840_rc     attttaaagtatccatacaatattt----taataaacttgttattgtcatgaacctctattcggta

                                10759
DS571167_92206-110299       tggttattctttgttttgcacttcatttcttttataaagaaataacatccattactttttattagt
DS548606_16670-34840_rc     t--ttattctttt-tttcgtatttcatttcttctctgaaaaatgaagaacc----ctttttattatt

                                10825
DS571167_92206-110299       aagaaataatctaactctatcatattcaagtactttttctttatttcttaaatagaataactatca
DS548606_16670-34840_rc     tagaaataatattatttggtcatatttcattttattttctttatttcttaaatagaataattatca

                                10891
DS571167_92206-110299       accactttattgattaaaaataaaacctcaatattaattctctcattctattaattaaataatgaa
DS548606_16670-34840_rc     attccaatattgattgcaaataaaatcatagtattatttctatc-tcttattaactaa-ta-tgaa

                                10957
DS571167_92206-110299       agttttattgaacaacttttcattaattgtactatgcttctacactaaaaaaaatgatatactaat
DS548606_16670-34840_rc     agattta-tgaataactttttatcaattgtaatatatttatactctaaaaaaaaagatgtactaat

                                11023
DS571167_92206-110299       tgaattattattaattcattttcaaagacgatatataatgtattttctaatatatttatagtaata
DS548606_16670-34840_rc     agaatta-tattaaatcatttt--acagcagtatctaatctaacttctagtgtattcaaagtaata

                                11089
DS571167_92206-110299       aagttcaaaacctaattgtctagagcaataaaataaaattatcattaacatgatttataatga-gt
DS548606_16670-34840_rc     aaattcaagattgaattgtctaaaataatagaataaaattatcattgactcgatttataatggttt

                                11155
DS571167_92206-110299       tgtactt-ttctgattgataagatag-tttattattcttcaattaagtttgttcattaaacttaat
DS548606_16670-34840_rc     tatacttattctgtttaataagatagtttttttcttattcaaatgggtttgaccattaaactcaat

                                11221
DS571167_92206-110299       aatttgtagtaaag--tatttttcatccacgtatcaagacattacatataattgcaaaaaaagaac
DS548606_16670-34840_rc     aatttgtactaaaaacactttttcatctacaaatcaagacatttcaaatagttgtaaaaaa-----

                                11287
DS571167_92206-110299       caattaatggaaagaaataaatacctctaattatcatgtaaaagtagtctgctacattaattattt
DS548606_16670-34840_rc     ca-ttaactaaaagaaatagatacctctaactatcatataaaagtagtctgttgaattactttttt

                                11353
DS571167_92206-110299       ttcatgtctttttttaactatatcgaaactatcatttcagtggttcgtttcaataatgttttaata
DS548606_16670-34840_rc     ttcatgtccatttttaactgtattgaggtgataacttcaatggttcgcttcaataatgttttaata

                                11419
DS571167_92206-110299       ataataaagctgttattggtaattttttaaataaccaaatactttcttataattttattttgttct
DS548606_16670-34840_rc     ataataaag-tcctattagttattttttaaacgaccaaatactcaacaataatttaagtttgttcc

                                11485
DS571167_92206-110299       tactttatcttacaataaatagcatatatcagcatgtgccttttattgtaaataataaaaaatgga
DS548606_16670-34840_rc     tactttgttttac-ataaataaaatgtactagaacatacctttta-tgtagtaacgaaaaaataga

                                11551
DS571167_92206-110299       tatttaaacaatatcaata--ttaatcaaaattta-aaagagtgttaattatatgagttatattct
DS548606_16670-34840_rc     tatttaaacaacaacaaaaacataatcaagatttataaagagtgtgaattatataatttatattct

                                11617
DS571167_92206-110299       cttctttctctatatataaaaaatagactattaataaatgaataaaataga---gtaaactaaata
DS548606_16670-34840_rc     ctt-tttctcaatatacaaaaattag-ttattaattaatcaaaaggataaaaaggtaaactaaata

                                11683
DS571167_92206-110299       ctttaaaaaacgaata-----gcattaataaaagaagctactaaagaattaattaatTTAAGCAAA
DS548606_16670-34840_rc     attc-caaaatgagtaataatgaattaatgaaagaagacactaaaaaatcaattaatTTAAGCAAA

                                11749
DS571167_92206-110299       GATTGGAATAAACTTTTGTTTTTTTGCATGCATCAAAGAAGCACCAGTTGA------TTGCTTTTC
DS548606_16670-34840_rc     GATTGGAATGAATTTTTGTTTCTTTGCATGTATCAAAGA---ATCCGTAGATGACGATTGCTTTTC
```

```
                   11815
DS571167_92206-110299   ATTTACACAATCTTGAGCCTTTAAACCTTTGTAATAACCATCAACATTACATTCATTATAAGCTGA
DS548606_16670-34840_rc ATCTACACAATCTCGAGCTTTCAACCCTTTATAATAACCGTCAGTCGTACATTCATGATAATCTGA

                   11881
DS571167_92206-110299   CCCACAATATGCTTTAACGTTATCATCATCAACTTCATAACACATTTCTTTAAATGCATATCTATT
DS548606_16670-34840_rc TCCACAAAATGCTCTAACATTATCATCATCAACTTCATAACACATTTCTTTAAATGCATATCTGTT

                   11947
DS571167_92206-110299   ATAATCCCAACTAATTTGATGTTCATTACAATACCACCAATTACTACAGTCACTAGGTACATAATA
DS548606_16670-34840_rc ATAATTCAGATTAATTTGATGTTCATAACAATAACCCCAATCACTACAACCACTAGGTACATAATA

                   12013
DS571167_92206-110299   TTCTGCAATAAAAGCCTTAACATAAGGTTTATGTCTTGAGTCAGTGAAAGGATAATCATATATATA
DS548606_16670-34840_rc TTCAGCAATAAAAGCTTTAACATAAGGTTTATGTCTTGAATCAGTGAAAGGATAATCATAAATATA

                   12079
DS571167_92206-110299   AACAAATCTTTGATTATTAGTACTTTCTAATAAAGGTTCTTTTCTTCTCCAAATAATTAAAGGATT
DS548606_16670-34840_rc AACGAATCTTTGATTATTAGTAGTTTCTAATAAAGGTTCTTTTCTTCTCCAAATGATTAAAGGAGT

                   12145
DS571167_92206-110299   TCCAAATTTAGTATTATAAAGCATATCCATAACTCTATTTGGATATGAATATAAACAAATCATTTG
DS548606_16670-34840_rc TTCAAATTTAGTATTATAAAGCATATCCATAACTCTATTTGGATATGAATATAAACAAATCATTTG

                   12211
DS571167_92206-110299   ATATGATGCAAATTCCCAATATTGAAGTTCTCTTTCAGTAGTTAGTTTTCTAACTCTCATTTGTTG
DS548606_16670-34840_rc ATAAGATGCAAACTCCCAATATTGAAGTTCTCTTTCGGTACTTAATTTTCTAACTCTCATTTGTTG

                   12277
DS571167_92206-110299   TGTATTATAAGCATTAATATCAATACGTAACATAGACATTGTTTCAAATTCTACATCAAGTACATA
DS548606_16670-34840_rc TGTGTTGTAAGCATTAATATCAATACGTAACATAGAAAGTGTTTCAAATTCTACATCAAGTACATA

                   12343
DS571167_92206-110299   CCTATCAGAAGAATCTTTCAATTTCTCAAAATTATTTGTTGGGTCATATTCCATCGCAAATAATAC
DS548606_16670-34840_rc TCTATCAGAAGTATCTTTTAATTTCTCAAAATTATTATTTGGGTCATATTCCATCGCAAATAATAC

                   12409
DS571167_92206-110299   TCTACAACAAGTCTCACATTTATCTCTTTGAATAATTTCTTGAACAACCACTTTTTCATCAATAAG
DS548606_16670-34840_rc TCTACAACATGTTTCACATTTATCTCTTTGAATAATTTCTTGAACAACTACCTTTTCATCAATAAG

                   12475
DS571167_92206-110299   TGCATTAGTATGTATTACAAGTAATACACAAAGAATGAATATCATgttatactgtttgaagttcta
DS548606_16670-34840_rc TGCATTAATATGTATTACAAGTAATACACAAAGAATGAATCTCATgttatactgtttgaagttctt

                   12541
DS571167_92206-110299   ctatcctttatttttcatagttcttggattggatactaaaaagtttgttgagttgaatttccttca
DS548606_16670-34840_rc ctatcctttatttttcttagttctt------gatactaagaagtttgttgagttgaatttctttta

                   12607
DS571167_92206-110299   tttcaattctatttattcttttataatttcttttattataattttactatta-tctttcttttgaa
DS548606_16670-34840_rc tttgaa-tctatttctttttttataatttcttttattactttttttactattattcttccttttgaa

                   12673
DS571167_92206-110299   atttcattttaacttttcgacttttttcatttttattcttttcctcctttaggaacttaatacatt
DS548606_16670-34840_rc atttcttttttacttttcgacttttttcatttgtattcttttcttcctttaggaacttaattcatt

                   12739
DS571167_92206-110299   attgatttattctattttttcatttttctcctcaaaatgaatgaaaagacatttacttcatgaaaa
DS548606_16670-34840_rc attgatttattctattttttgatttttctcctcaaaataactgaaaagacatacacttcatgaaaa

                   12805
DS571167_92206-110299   cttaacctactcacccatatgaattataacctacttgaaaccctcttaaaatcctctgtctcatac
DS548606_16670-34840_rc tattaactactcaaccagatcaattacaacccacttgaaa-cctcttaatctctttt-tttcatac

                   12871
DS571167_92206-110299   taaaagagtctagagaaactcattaaacaaagtcattaattaaaaaggatatttttaaagtcaaag
DS548606_16670-34840_rc agaagtatcatagagataatcacaaaacaaagccattaactgaaaaaggtattt------------

                   12937
DS571167_92206-110299   ataaaaacatacagaaagaaatgaatagaaagaagtagataaatattaaa-agataa-aaaaaaga
DS548606_16670-34840_rc acaagaacaaagacagagaaatgaatgg------ttagataaaagttaaatggataagaagaaaga

                   13003
DS571167_92206-110299   agaagaaaacaacaaacacaagatacaataaagaaaatttaatgaaaagaaaaaaaaaagaaagaa
DS548606_16670-34840_rc agaagaaaataa---------------------aaaatttaataaaaagaaaaaa----gaa-g--

                   13069
DS571167_92206-110299   gaagtaaaataaaagTCATTTTTTAATAAATTTTAAGAGAAATTGTTCAATAGTTTTAGAATCACA
DS548606_16670-34840_rc ---ttaaaataaaagTCATTTTTTAATAAATTTTAAGAGAAATTGTTCAATAGTTTTAGAGTCACA
```

```
                          13135
DS571167_92206-110299     TTGACGACGAAAACCATCTTTAGGACGAACAAAAGAAGAAGAAAAGTATTTGATAAGATCTTCTGG
DS548606_16670-34840_rc   TTGACGACGAAGACCATCTTTAGGACGAATGAAAGAAGAAGAAAAGTATTTGATAAGATCTTCAGG

                          13201
DS571167_92206-110299     ATGAAGAGAAGAATTTTGACGAAGAATTTCTTTAATAAAAGATAGTAAATAAATAAAAAGGTTATA
DS548606_16670-34840_rc   ATGAAGAGAAGGATTTTGACGAAGAATTTCTTTAATAAAAGATAGTAAATAAATAAAAAGATTGTA

                          13267
DS571167_92206-110299     ATGAGAATTTGGAATAGAAGCATTATTTCTAATAAAGAATTTATCTATTTCAACATCAGGTTTATC
DS548606_16670-34840_rc   ATGAGAGTTTGGAATAGAAGCATTGTTTTTAATAAAGAATTTATCTATTTCGATATCAGATTTATC

                          13333
DS571167_92206-110299     AGCGGATAAAATAACATGTTCATATAAAGAAGAAGGAATAATAGAGTCATTAAGATTTACTAAAAA
DS548606_16670-34840_rc   AGCGGATAAAATAACATGTTCATACAAAGAAGAAGGAATAATAGAATCATTGAGATTTAATAAAAA

                          13399
DS571167_92206-110299     GATTAATAAGAATTCTAAAACACGATGATAATTTAAAGAAGGATTAAAAGAAGAATGAGTATCAAG
DS548606_16670-34840_rc   GATTAATAAGAATTCTAAAACACGATGATAATTTAAAGAAGGATTAAAAGAAGAATGAGTATCAAG

                          13465
DS571167_92206-110299     AGAATGTAATATTTCATAATAAATAGGAACATAATCAATTGAAGGATGTGATAAAGAAATAAAAGT
DS548606_16670-34840_rc   AGAATGTAATATTTCATAATAAACAGGAACATAATCAATTGAAGGTTGTGATAAAGAAATAAAATT

                          13531
DS571167_92206-110299     AGGGTCATGCATAAAAGGAAGTACATAATCACAAAGTCTCCAAATTTCTTTAGGAATAACTAAAGG
DS548606_16670-34840_rc   AGGGTCATGAATAAAAGGAAGTACATAATTACAAAGTCTCCAAATTTCTTTAGGAATAATTAAAGG

                          13597
DS571167_92206-110299     AGTATAAGCTTGAGGTTGAGATAAAATATAACCATTAGGATGTAAATTTAAGTCATCAATAGATTT
DS548606_16670-34840_rc   AGTATAAGCTTGAGGTTGAGGTAAAATATAGCCATTAGGATGTAGATTTAAATCATCAATAGATTT

                          13663
DS571167_92206-110299     ACCAAATGAAGTTTGTAATAAAACTAATTTAAAAGTAATAAATAAACTTTTTCCACCTTGAATTTC
DS548606_16670-34840_rc   AGCAAAAGAAGTTTGTGATAAAATTAATTTAAAAGTAATAAATAAACTTTTTCCACCTTGAATATC

                          13729
DS571167_92206-110299     AAGAATCAAATTATAATCAAAAGACCCATTTTTAAATAAACTTGATGTATCAATAAAATTGAATTG
DS548606_16670-34840_rc   AAGAATCAAATTATAATCAAAAGATCCATTTTTAAATAAATTTAATGTATCAATAAAATTAAATTG

                          13795
DS571167_92206-110299     AAAATAGAAAGTAATACTTTTAGGTTCAAAATCATTTTTTGATATTTCTAAATGTTGTGGTTCAAT
DS548606_16670-34840_rc   AAAATAAAATGTAATACTTTTAGGTTCAAAATCATTTTTAGATATTTCTAAATGTTGTGGTTCAAT

                          13861
DS571167_92206-110299     ATGTAACCAATCTGGATATCCACCAGCTTTTTCTAATTGATGATCTCTAAAAAGTATTGATGTTTT
DS548606_16670-34840_rc   ATGTAACCAATCAGGATATCCACCAGCTTTTTCTAATTGATGGTCTCTAAAAAGAATTGATGTTTT

                          13927
DS571167_92206-110299     AGCATTTCCATCATTTTGAACAAGTAATGTAACTTCTGTTTGTTTATATGGATAAATTTCTACTAT
DS548606_16670-34840_rc   AGCATTTCCATCATTTTGAACAAGTAATGTAATTTCTGTTTGTTTATATGGATAAATTTTTAATAT

                          13993
DS571167_92206-110299     AAATTCTTGTTTATTAATTTTAACTGATGGTCTAACAATTCTAAGTAATTTATTTTCCATTTCAAT
DS548606_16670-34840_rc   AAATTCTTGTTTATTAATTTTAACTGATGGTCTAACAATTCTAAGTAATTTATTTTCCATTTCAAT

                          14059
DS571167_92206-110299     AAGTTCTTTTTCAATTAATGTATATTTTTCTTTTATAATACTTCTTGTTTCAACACTATAACATCC
DS548606_16670-34840_rc   AAGTTCTTTTTCAATTAACATATATTTTTCTTTTATAATACTTCTTGTTTCAACACTATAACATCC

                          14125
DS571167_92206-110299     AATAACTGGTTTATGGTCTGATTCAATTGCTTCTTTTTTACTTGTATATTTTAAACATTTAATTTC
DS548606_16670-34840_rc   AATAACTGGTTTATGATCTGATTCAATTGCTTCTTTTTTACTTGTATATTTTAAACATTTAATTTT

                          14191
DS571167_92206-110299     TAATCCTTTACATTCTTTATATAATATTCTATCACAATATGCTGGTTTTCTTTTTTCTGAATAATT
DS548606_16670-34840_rc   TAATCCTTTACATTCTTTATATAATATTCTATCACAATATGCTGGTTTTCTTTTTTCTGAATAATT

                          14257
DS571167_92206-110299     TTGTGTATTTGGATAAAATTTATATGTTGGTTCAAATTCAATTTCTGCTTCTTTAAATTTTCCTAA
DS548606_16670-34840_rc   TTGTGTATTTGGATTAAATTTATATGTTGGTTCAAAATTAATTTCTGCTTCTTTAAATTTTCCTAA

                          14323
DS571167_92206-110299     TAATTCATTATGTTTTTGTTGTACTTTTAATTGATCTTTTTCTTGTATTATTTCTGTAATACTTCC
DS548606_16670-34840_rc   TAATTCATTATGTTTTTGTTGTACCTTTAATTGATCTTTTTCTTTTATTATTTCTGTTATATTTCC

                          14389
DS571167_92206-110299     TTTATTAATTCTTTCTCTAATAATGCTATCTTCTTCATCAATTCTATAATTTAAATCTCCCATCCA
DS548606_16670-34840_rc   TTCAATAATTTTTTCTCTAATAATACTATCTTCTTCATCAATTCTATAATTTAAATCTCCCATCCA
```

```
                          14455
DS571167_92206-110299     AAATATAATATCATGTCCTTCAATTTTATT---CCATTTAGTTTGTTCTCCTTCTTCTGTTCTTAT
DS548606_16670-34840_rc   AAATATAATATCATGTTCTTCAATTTTATTACAAATCACTTCTTCTTCTCCTTCTTCTATTCTTAT

                          14521
DS571167_92206-110299     TAATTTAATATTATTATAAATATCACTAAAATTTCGATTTCTTTTATTAATTTCAGGTTGATGTGC
DS548606_16670-34840_rc   TAATTTAATATTATTATAAATATCACTAAAATTCCTATTTCTTTTATTAATTTCTGGTTGATGTGC

                          14587
DS571167_92206-110299     TGCAAGATGTGATCCAACTAAACATATAGTTGTATCATACATTTCAAATGAAATTGCAATCCCCCC
DS548606_16670-34840_rc   TGCAAGATGTGATCCAACTAAACAAATTGTTGTATCATATATTTTAAATGAAATTCCAATTCCTCC

                          14653
DS571167_92206-110299     TTTATTTGCCATTCCCATAGCTCCAACTGCTTCATATCCACTTGCAAATTGTTTTATTTTATTTTC
DS548606_16670-34840_rc   TTTATTTGCCATTCCCATAGTTCCAACTGCTTCATATCCACTTGCAAAATATTTAATTTTATTTTC

                          14719
DS571167_92206-110299     CATTTCTTTTCTAACAAATCCAATTATACAAACCCCTCCTAATTGTGAAACATATACTTGTTTATA
DS548606_16670-34840_rc   TATTTCTTTTCTAACAAATCCAATTATACAAACTCCTCCTAATTGTGAAACATTTACTTGTTTATA

                          14785
DS571167_92206-110299     TTCTTCTTTGTAAATATTTTTTAATGCTTCTTGAATATTTTGTGCTAAATTTCTTGCTTTTAAACT
DS548606_16670-34840_rc   TTCTTCTTTATAAATATTTTTTAATGCTTCTTGAATATTTTGTGCTAAATTCCTTGCTTTTAAACT

                          14851
DS571167_92206-110299     TAATGTATTTCCTTTAAGAAATCCTGAAACACTCATATCTATTTCTTCAACAACTATAAATATCAT
DS548606_16670-34840_rc   TAATGTATTTCCTTTAAGAAATCCTGAAACACTCATATCAATTTCTTCAACAACTATAAATATCAT

                          14917
DS571167_92206-110299     ATCAATTTGATATTCTTTTATGTCTAAAATATATTCAAATGTTTCTTTTTTAATTATTTTTTCATT
DS548606_16670-34840_rc   ATCAATTTGATATTCTTTTATTTCTAAAATATATTCAAATGTCTCTTTTTTAATTATTTTTTCATT

                          14983
DS571167_92206-110299     AATGTTATATGTTACTAATAACATATTCACATCTTTTCTTATTGAATATTCATCTCTTCGTTCTTT
DS548606_16670-34840_rc   AATATTATATGTTACTAATAACATATTTACATCTTTTCTTATTGAATATTCATCTCTTCGTTCTTT

                          15049
DS571167_92206-110299     TATTTTATCATATCTCCAATTTAATGGGTCATCTGCATATCTTTCAActatattttgttattctct
DS548606_16670-34840_rc   TATTCTATCATATCTCCAATTTAATGGATCATCTGCATATCTTTCAActatatattattattat--

                          15115
DS571167_92206-110299     tattattattattattattatttgtagtaacaaacTTTCTTCTTGAGGAATGTTCATttc-tttcc
DS548606_16670-34840_rc   --ttattatcat--ttattatatgtattaacaaacTTTCTTCTTGAGGAATGTTCATttcttttcc

                          15181
DS571167_92206-110299     tttctttatttctcctttgttt------tttagtttctttttttaat--------ttttcttttct
DS548606_16670-34840_rc   tttctttaattctcttttgttttcttagttttgtttttttttttaatttttttaatttttttttct

                          15247
DS571167_92206-110299     tctttttctta-ttttttttgttattctttgcaaaacaataaacccaaaaactaacaactttactt
DS548606_16670-34840_rc   tctttttcttatttttttttgttattctttggaaaacaataaacccaaaaactaacaagtttactt

                          15313
DS571167_92206-110299     cATGAACCTGTTATTCCAAAATGAACATCTTTTTGATGAAGCTTGTACAAATTATTTCAGACATCC
DS548606_16670-34840_rc   cATGAACCCGTTATTCCAAAATGAACATCTTTTTGATGAAGCTTGTACAAATTATTTTAGACATCC

                          15379
DS571167_92206-110299     TAATCAATCACATGTATTAAATAACTATTTAAATACCCCATTACCAAGTGATTTAGTCGATCAATT
DS548606_16670-34840_rc   TAACCAAACACATGTATTAAATGATTATTTAAATAGCCCATTACCAAGTGATTTGATCGATCAATT

                          15445
DS571167_92206-110299     AGTCAAATTTTTTTCATCAAGAAATATTTATACAACACAATACATATCACAATTACTTATTCGTCA
DS548606_16670-34840_rc   AGTAAAATTTTTTTCATCAAGAAATATTTATACAACACAATATATTTCACAATTACTTATTCGTCA

                          15511
DS571167_92206-110299     CTTAAATAATCATACATTGAATTATTCATATGACAAACTTATTCATTCATTAGTTCCATACGCATG
DS548606_16670-34840_rc   ATTAAATAATCATACATTAAACTATTCATATGATAAACTTATTCATTCATTAGTTCCATATGCATG

                          15577
DS571167_92206-110299     TATTGTAGGATCGCCATATTCAGATTTATATTGTAAATTATTAATTAAAAAATGGAATAAAATGCC
DS548606_16670-34840_rc   TATTGTAGGATCACCATATTCAAATTTATATTGTAAATTATTAATAAAAAAATGGAATAAAATGCC

                          15643
DS571167_92206-110299     AGATATGATGGAACAATCAACATACGAATTATGTCAAGCATGTGAAAGTAAAATAGCTACAGAAGA
DS548606_16670-34840_rc   AAATATGATGGAACAATCAACGTATGAATTATGTCAAGCATGTGAAAGTAAAATAATTACAGAAGA

                          15709
DS571167_92206-110299     AGGAAAAGGGATAATGTATTTAATAGGAAATCTTATTAAAGAATTTATTGAAACAAAAGAAGTTGA
DS548606_16670-34840_rc   AGGAAAAGGGATAATGTATTTAATAGGAAATCTTATTAAAGAATTTATTGAAACAAAAGAAAATGA
```

```
                         15775
DS571167_92206-110299    TTCAATTACAATTCAAATATTTAAAGAGAAGTGTTTATTAAGAATAGTTGAAACAATAATCACTAT
DS548606_16670-34840_rc  TTCAATTACAATTCAAATATTTAAAGAGAAATGTTTATTAAGAATAATTGAAACAATAATTACTAT

                         15841
DS571167_92206-110299    TATTCGATGGATTCAAATAGAAAATAATGAACAAGAAATTCAATGTAAAATTGCATTAGAAACATT
DS548606_16670-34840_rc  AATTCGATGGATTCAAATAGAAAATATTGAACAAGAAATTAAATGTAAAATTGCATTAGAAACATT

                         15907
DS571167_92206-110299    GAAAATAGCATTAGAATATCCATTTAGTAATGGATATGAAGAGGAAGAAGACATTGTTTTAGAAAT
DS548606_16670-34840_rc  AAAAATAGCATTAGAATATCCATTTAGTAATGGATATGAAGAAGAAGAAGATATTATTTTAGAAAT

                         15973
DS571167_92206-110299    TCCAATGGGATATGCGTCAATACTTTCAAGCATTGGATTACAAGAAAAAATGATTAGAATGTTTGT
DS548606_16670-34840_rc  TCCAATGGGATATGCATCAATACTTTCAAGTATTGGATTACAAGAAAAAATGATTAGAATGTTTGT

                         16039
DS571167_92206-110299    TCCAGGAAATCATCTTGAAGCATTAGTTGAGTGTATGATAACATTAGCAAGTGTTAGAGAGAATTT
DS548606_16670-34840_rc  TCCAGGAAATCATCTTGAAGCATTAATTGAATGTATGATAACATTAGCAAGTGTTAGAGAGAATTT

                         16105
DS571167_92206-110299    ATTTAGTGGAACAACACGTCAACAATATAATGAGTTTTTAAGTAAGTTTGGAGGAATGATTTGTGA
DS548606_16670-34840_rc  ATTTAGTGGAACAATTCGTGAACAATATAATGAATTTTTAAGTAAATTTGGAGGAATGATTTGTGA

                         16171
DS571167_92206-110299    AGCAGCAGAAAATGAAAAAATGGCAGTTGCAGCAGGGAGATATTTAGATAGAATGACAAGAACAAG
DS548606_16670-34840_rc  AGCAGCAGAAAATGAAAAAATGGCAATTGCAGCAGGGAGGTATTTAGATAGAATGACAAGAACAAG

                         16237
DS571167_92206-110299    AAGTGTTAAAGCACAATATCATTTAACACAAGCAGCAAAATTGATAGAAAGAATTAGTGAAAATTA
DS548606_16670-34840_rc  AAGTGTAAAAGCACAATATCATTTAACACAAGCAGCAAAATTAATAAAACGAATTAGTGAAAATTA

                         16303
DS571167_92206-110299    TCTAGAACATAAAAATAGTTTATATTATTTATTAACAACATTTGCAAGATGGTCACATAGATTATA
DS548606_16670-34840_rc  TATAAAACATAAAAATAGTTTATATTATTTATTAATAACATTTGCAAGATGGTCACATAGATTATA

                         16369
DS571167_92206-110299    TGAATGTGGAGAAGAAAAAGGAAATGTTATTGAAACACTAAAAGAAGTTAGTAAAAGTGTAATTGA
DS548606_16670-34840_rc  TGAATGTGGAGAAGAAAAAAGATTTGTTATTGAAACATTAAAAGAAGTTAGTATAAATGTAATTGA

                         16435
DS571167_92206-110299    AGGAGCATTTAATAATAGTGAAATCATTGAAGAAGAAGATATTGAAGGAATAATGGAAGGATTTGG
DS548606_16670-34840_rc  AGGAGCATTTAATAATAGTGAAATAATTGAAGAAGAAGATATTGAAGGAATAATGGATGGATTTGG

                         16501
DS571167_92206-110299    AGAAATAACAAGAGTTAATTTAATGTTTACTGTAGAAGAAATGACAAAACATATTGAAAGAGTTAC
DS548606_16670-34840_rc  AGAAATATCAAGAATTAATTTAACAATTACAGTAGAAGAAATGATAAAACATATTGAAAAAGTTAA

                         16567
DS571167_92206-110299    AAAAGAAATAGAACATATAATAGAAATTAAAGGAAGTTTACAAAGAATACAAAAAGAATGTGAAAG
DS548606_16670-34840_rc  AAAAGAAATAGAAGAAATAATAAAAATGAAAGGAAGTTTACAAAGAATACAAAAAGAATGTGAAAG

                         16633
DS571167_92206-110299    ATTAAGTATTGGGATAGGATTAATAAGTATTATTATAAATCACCAAGTAATTAATGGAACAAATGA
DS548606_16670-34840_rc  ATTAAGTATTGGAATAGGATTAATAAGTATTATTATAAATCATCAAATAATTAATGGAACAAATGA

                         16699
DS571167_92206-110299    AACACAAGAACAATTAGATATTAAATTAATGAAAATTGGAATTGAAATTATTAATAAAGAAATGAA
DS548606_16670-34840_rc  AACACAAGAAAAATTAGATATTAAATTAATGAAAATAGGAATTGAAATTATTAATAAAGAAATGAA

                         16765
DS571167_92206-110299    TATTATAAAAACTATTCCATTTGGGTATGGAGTAAAAGAAATAGAAATAAATATATTAAGATTTTT
DS548606_16670-34840_rc  TATTATAAAAAATATTCCATTTGGATATGGAATAAAACAAATAGAAATAAATATATTAAGATTTTT

                         16831
DS571167_92206-110299    TGAGAGTATTCGTAATGTATTTCTTAATGAAAGTATTTCAAGAACAACAGAAAAATATATAATAGA
DS548606_16670-34840_rc  TGATAGTATTCGTAATATATTTCTTAATGAAAGTATTTCAAGAACAACAGAAAAATATATAATAGA

                         16897
DS571167_92206-110299    AATATTAGAAATTCAAAATTTAAATGATTTAATAATTGCAATTATGAATAAAATCATTTTTAATTT
DS548606_16670-34840_rc  AATATTAGAAATTCAAAATTTAAATGATTTAATTATTGCAATTATGAATAAAATCATTTTTAATTT

                         16963
DS571167_92206-110299    ACATTATTTTATAGATATTCATTGTGAATCTATTATTAAACAATCATTACTTATTATTGAAAAATA
DS548606_16670-34840_rc  ACATTATTTTATTGATATTCATTGTGAATCTATTATTAAAGAATCTTTATTTATTATTGAAAAATA

                         17029
DS571167_92206-110299    TTCAAGAAAAGAAAAAACATCAAAAATGTTATTAGAATATGGATTACTTGATAAAATATTAGATGA
DS548606_16670-34840_rc  TTCAAGAAAACAAAAAACTTCAAAAATGTTATTAGAATATGGATTACTTGATAAAATATTAGATAA
```

```
                       17095
DS571167_92206-110299     ACCATTAgtttgtttattttctttatattcttatttattattattatttattaatatttattatta
DS548606_16670-34840_rc   ATCTTTAgtttgttt----------tattttta-ttattattattttttattattatttattatta

                       17161
DS571167_92206-110299     gACTCAAAAAATAACAAAATTATCTGTTAAAACAAAAAAAATGTTTTTCCAAGCTATTTGTAATTT
DS548606_16670-34840_rc   gACTCAAAAATTAACAAAATTATCTATTAATACTAAAAAAATGTTTTTCCAAGCAATTTGTAATTT

                       17227
DS571167_92206-110299     AATTATAACTATTCCTAAAGTACATTTTTCTGTATTATTAAATAAACTTGAATCTTCTTTTCATCT
DS548606_16670-34840_rc   AATTATAACTATTCCTAAAGTACATTTTTCTGTATTATTAAATAAATTGGAATCTTCTTTTCTTCT

                       17293
DS571167_92206-110299     TCAACCAAATGACTACGAAAGTAAATTACTTTTATATCATTCTTTCTTCTTATCATTACCTCCTGA
DS548606_16670-34840_rc   TCAATCAAATGATTATGAAAATAAACTACTTTTATATCATTCTTTCTTTTTATCATTACCTCCTGA

                       17359
DS571167_92206-110299     AAGTATTCATTATCTTGGATTTATTGATTTTGTTCATAAGATATTACCTATTTTTATTAAAAACAC
DS548606_16670-34840_rc   AAGTATTCATTATCTTGGATTTATTGATTTAGTTCATAAATTATTACCTATCTTTATTAAAAATAC

                       17425
DS571167_92206-110299     ATCTTTACCTTCATTTGTTTCATTCTTATCTGATATTGCAAACATTAAACCTTTTAGATCACTTTA
DS548606_16670-34840_rc   ATCTTTATCTTTATTTGTTTCTTTCTTATCTGATATTGCAAATATTAAACCTTTTAGATCACTTTA

                       17491
DS571167_92206-110299     TTCTATACAAACTCCAGACGCATTAATATTATATCATGACATTTGTGAATCTTTAATGAAGATATT
DS548606_16670-34840_rc   TTCTATACAAACTCCTGATGCATTAATATTATATCATGATATTTGTGAATCTTTAATGAAAATATT

                       17557
DS571167_92206-110299     ACCTTCTATTATCGAATTAAATGAAGATGAGATGGTGTCATATTTAGAAGATTGTATCCAATCAAT
DS548606_16670-34840_rc   ACCTTCTATTATTGAATTAAATGAAGACGAGATGGTATCTTATTTAGAAGATTGTATCCAATCAAT

                       17623
DS571167_92206-110299     TAATTCTATTTTATCAAGTGATACCATCCCTTTTGGTGCTTTGTATGTCTATAATGATCGAACTCA
DS548606_16670-34840_rc   TAATTCTATTTTATCAAGTGATACTATCCCTTTTGGTGCTTTATATGTTTATAATGATCGAACTCA

                       17689
DS571167_92206-110299     TCATGTATTGATGCAAACAATTATTTCTATCTGCATTTCACATAAATGGGATTTTGTTAGTTTTTA
DS548606_16670-34840_rc   TCATGTATTAATGCAAACAATTATTTCTATTTGTATATCACACAAATGGGATTTTGTTAGTCTTTA

                       17755
DS571167_92206-110299     TCCAAAATATACTAAACTTATTTTTACTCTCTTTTGTAATATTGGTATGGTGTCTTGTGATGACTT
DS548606_16670-34840_rc   TCCAAAATATACTAAACTGATTTTTACTCTATTTTGTAATATTGGAATGGTATCCTGTGATGACTT

                       17821
DS571167_92206-110299     CTTTGGTAATCATCTTCATGAAACATTACTATTTTTGTTTAATGCTTTACAATCTGGTGAAGAGTC
DS548606_16670-34840_rc   CTTTGGTAATCATCTTCATGAAACATTACTATTTTTATTAAACGCTTTACAATCTGGTGAAGAATC

                       17887
DS571167_92206-110299     TGCTATCCCAGTTTTTGAACAAATCATCTTATTCACTTTTAAAAGCCATCTTCTAAAATCTGTAAG
DS548606_16670-34840_rc   TGCTATCCCTGTTTTTGAACAAATTATCTTATTCACTTTTAAAAGTCATCTTCTAAAATCTGTAAG

                       17953
DS571167_92206-110299     AATCATCACCACTCCTTCAACCGATCATTCTTTGTTATTGTCTCAACATTTGGACTTAGTCAAAAA
DS548606_16670-34840_rc   AATCCTCACTACTCCTTCAACAGATCATTCTTTGTTATTGTCTCATCATTTGGATTTAATCAATAA

                       18019
DS571167_92206-110299     CATCATTGAAATTCTCCTTCAAAATCTACTTAATGGAAACATGGATTTATATTGTACTTCTAAAGC
DS548606_16670-34840_rc   AATCATCGAAATTCTCCTTCAAAGTCTAATTAATGGAAACATGGACTTATACTGTATTTCAAAAGC

                       18085
DS571167_92206-110299     CTTATTACCTTCTCTTCTTCTTTATCCTAAAATATATCACCATTTGAAATCTTCTCTTTTACTCAA
DS548606_16670-34840_rc   TTTGTTACCTTCTCTTCTTCTTTATCCTAAAATATATCACCACTTGAAATCTTCTCTTTTACTCAA

                       18151
DS571167_92206-110299     ATACTCAAATTCTCCTGACTTAAATCTCGCATTCTGCCAACTCGACGCTTCTATCTCTTCTTCTTG
DS548606_16670-34840_rc   TTACTCAAACTCTCCTGATCTAAATCTAGCTTTCTCCCAACTCGACGCTTCTCTCTCTTCTTCTTG

                       18217
DS571167_92206-110299     TGATGGGGATGCTTACGATAACTTCTTTAACGCTTGCCAAGTCTTCCAACATACTTCTTTATCTCT
DS548606_16670-34840_rc   TGATTCTGATGCTTATGATAACTTCTTTAACGCTTGCCAAGTCTTCCAACATACTTCTTTATCTCT

                       18283
DS571167_92206-110299     GCTCAAACAATGAcatgtttccaattcaa-ttcaatcattttgatagcgagtattgtgggaattaa
DS548606_16670-34840_rc   GTTCAAATAATaa---g-----aa--caaaaataaaagatttgattggttgtacttt-----ttat

                       18349
DS571167_92206-110299     tcaacaaaaaaacatcaagaaacgagaaaatccca------gaatcat------acaact
DS548606_16670-34840_rc   tgaactaagcaattgttctatttactttcattccattttatgtattactttttaacaat-
```
