# Supplementary material for: Evidence of Gene Conversion in Genes Encoding the Gal/GalNac Lectin Complex of Entamoeba
Source: PLoS Negl Trop Dis. 2011 Jun 28;5(6):e1209. doi: 10.1371/journal.pntd.0001209 (PMC3125142; doi:10.1371/journal.pntd.0001209)
Supplement: Figure S8 — Amino acid multiple alignment of intermediate chain lectin ( igl ) gene family members from E. histolytica and E. dispar , used to generate a gene phylogeny. (PDF) [file pntd.0001209.s008.pdf]

```
Alignment: /Users/garethweedall/Desktop/igl_4seqs.fas
Seaview [blocks=70 fontsize=10 A4] on Fri Nov  5 15:17:30 2010

            1
EHI_006980  MFILLLFISISLGDYTADKLIGGKEPREAVPHCASVSNGACTSCDTGYELTT---TGNNKTCTLKEDMCK
EDI_276450  MFIILLFISISLGEYKADKLIKGQEPRTAVPHCASVSNGACTSCDVGYELSSD--SSNTQKCTLKQDICK
EHI_065330  MFILLLFISISLGDYTADKLINNQEPRTAVPHCASVSNGACASCDEGYELKTESGSGSTQKCTLKEETCK
EDI_244250  MFILLLFISISLGDYKADKLIGDKEPREAVPHCASVSNGACASCDDGYELKTE--SG-SNKCVLKEGTCK

           71
EHI_006980  TAFSYYDKTNSTNPKCTYCVNGKEVNTSSHSGNDKCVCKNNVNICESCLLMK-DSKCGECIIGMSTTVD-
EDI_276450  TTFSYYDNSNASSPKCVYCENGKEANTPPNSNSEKCTCKNGVDNCDTCLSKDSGTKCEECIIGMSTTNTG
EHI_065330  SAFSYYDGSDSNSPKCVYCENGKESDTSS-SNNEKCKCKNGVDTCESCLSKD-NDKCGECVIGMSTTTNG
EDI_244250  SSFSYYDTSNSETPKCTYCEDGKEVDDSSTSSTDKCTCRNSVSTCDTCLYMGQDNKCGECVIGKYSTT--

          141
EHI_006980  GSKLCDNATTEDHAENCVGLLASSTSSKTCDKCFGMYSLQGGKCTQKNDKINKCILQVENSCNQCADGYS
EDI_276450  SSKGCDNATTDDHAENCIGLLASTTSSKTCDKCFGNYTLENGKCTKKNEKISNCILQVVNDCNQCADGYY
EHI_065330  GQKLCDTVTTDEHAENCVGLTAKDSSSKQCDKCFGMYSLQSGQCTKKNEKIEKCILQVESSCNQCADGYY
EDI_244250  GEKLCDNAIADDHAENCIGLSAKDSSNKACDKCFGSYTLQNNMCSNQLTKIDKCILQVENSCNQCADGYY

          211
EHI_006980  LSTDKKSCNKFPEHCSKINGNQCLTCMEGYYLSKTDSKCTICTVDNPNNLSEGNECSIYNAEHCTSCNKR
EDI_276450  INAEKK-CTKYPDHCSKMNGNQCSTCMEGYYLKDSK--CNVCTIDNPNNLSEGNECSIYNTEHCTSCNKR
EHI_065330  INTEKK-CTKYPDHCSKMNSDKCNGCMEGYYLNGTE--CKVCTIDNSKDLSEGNECSIYNAEHCESCNKR
EDI_244250  INAEKK-CTKYPDHCSKVNGEKCASCMEGYYLTGTE--CEVCTIDNLDDLSKGDECSIYSAKHCTSCNKR

          281
EHI_006980  CTVSDGVCVKNHCRLFSPTEENKCTKCDNGYFLTTSGTCSPNLYDGFKTANRTECENGYYLEKDGDKKRC
EDI_276450  CTVSGGFCTKNHCRLFSLTEENKCAKCDNGYFLTTEGKCSPNLYDGFTTSAKTECQPGYYLEKDGENKRC
EHI_065330  CTVSDGVCVKNHCRLFSPTEENKCTKCDDGYFLTGAGKCSPNLNDGFKTSAKTECQKGYYLEKDGDKKRC
EDI_244250  CTVSGEVCVKNHCRLFSLTDSSKCAKCDSGYFLTSSGTCSPNFYDGFKTSAKEECLPGYYLEKDGENKRC

          351
EHI_006980  SLCPDPFTECLTSKTPVPGKLNLRSSHLTSTDGPCKLPGCLLCSDDDTICYKCENGLTLNGTHCYNFDTK
EDI_276450  SLCPDPFTECLTSKTPVPGKLNIKNSHLTSTIGPCKLPGCLSCSDDDTICYKCENGLTLKGTHCYNTIIN
EHI_065330  SLCPDPFTECLTSQTPVPGKLNLRSAHLTSTDGPCKLPGCLLCSDDDTICYKCENGLTLNGTHCYNFDVK
EDI_244250  TLCPDPFTECLTSKTPVPGKLNLRSSHLTSNAGPCKLPGCLSCSDDDTICYKCDEGLTLRGTHCYNLDSV

          421
EHI_006980  SVLGTSGNNHQVCKMRGYDQYEQYLNAFKASDNTYYCPLKDLYLPYYFSVTKGTSD-NTITIGCVGQLRN
EDI_276450  DVLGISGKNHKVCKMRGYNQFEQYLNAFKASDNTYYCPLTDLFLPYYFNVTKNSKDMSKITIGCVGKSRD
EHI_065330  KVLGTSGNNHQVCKMRGYDQYEQYLNAFKASDNTYYCPLKDLYLPYYFSVTKGSDN--KITIGCVGKDRD
EDI_244250  NVLGTSGEEHRVCKMRGYNQFEQYLNAFKASDDTYYCPLTDLFLPYYFSVTKDSSN--AITIGCVGQLRN

          491
EHI_006980  VSNDCECNDKHIPTSIDKASDCVSITTKLPSCERTANGNICTQCPVGSHVGKDGKCSCGDAHYFDKDNVC
EDI_276450  VKNDCECEAKYIPTSIDKSSDCVSIATKLPSCERAANENICTQCPVGSHVGSDGKCSCGDGHYFDKDNTC
EHI_065330  VKNDCECNDKYIPKSVDKASDCVSIKTKLPSCERAANENICTQCPVGSHVDSNGKCSCGDAHYFDQNNKC
EDI_244250  VSNDCECNDKHVPTSIDKSSDCVSIVTKLPSCERAANENICTQCPVGSHVGSDGKCSCGDGHYFDENNVC

          561
EHI_006980  KKCPASCSSCSYDSSKSKVVCSECYENIQGVTTRNKENECACIND--GYKEGPNAEDKKKSCAQLNNNCK
EDI_276450  KKCPDSCSSCALDSTKNNVICTSCYENIQGVTTRDKDKKCACVSN--DYKEGPNEEDKKKSCAQLNKNCK
EHI_065330  QECPASCSSCSYDSSKSKVVCSECYENIQGVSTRDKDNECACKKDTPEYKEGLNAEDKKKSCAQLNNNCK
EDI_244250  QKCPDSCSSCSYDSNKKAVVCTACYENIQGVTTRDKNSQCACLDE--RYKEGPNEEDKKKSCAELNENCN

          631
EHI_006980  KEGKYEISDGFVTCLDCDDSAYIVGSQVGACTQCSPNAFKDENNKCQLCSTKQSQYGHCAACSATACITC
EDI_276450  NEGKYEISDGYVTCLDCDNPAYIVGSQISACTQCSPNAYRN-GNECVLCSTKQAQFGHCSSCSATACLTC
EHI_065330  EEGHYKISDGFITCLECDDSAYIVDSQTKECAQCASNAFKDENNKCQLCSTKKDKYGHCSACSATACIIC
EDI_244250  QEGKYEISNGYVKCLECNDPAYIVGSEINACSQCSSSAFKDSNNKCQLCSTKQDKFGHCSSCSATACITC

          701
EHI_006980  EDINLILTGEKP---CTVCKDGFYQIENATDGVYCSPCPAKCKTCKYNTTSKKVECVTCTEQRLKDIKAP
EDI_276450  EDNNLILTASGSNVQCTECKDGFYKIENPTDGVYCSPCPAKCKTCKYNTPTKKIECLTCTDTTSQDIKAP
EHI_065330  EDTNLVLAASGSNAQCTVCKDGFYQIESPTDGVYCSPCPAKCKTCKYSADKKEIECVTCTDQSSVDIKPP
EDI_244250  EDNNLILAASDSNVQCTECKDGFYKIENPTDGVYCSPCPAKCKTCKYDSTKQEVECLTCTDTTSQDIKAP

          771
EHI_006980  ECACPTGTVQLENGTCQSCSDLSKYPGCKKTDSCNVDSRTGFIYATECSDGFSGRSPYSNCTTCTKSNYY
EDI_276450  ECACPKETVQLENGRCKSCSELSKYEGCKTTDTCNVDAKTGYIYATECSENFNGRSPYSNCTACTLSNYY
EHI_065330  TCACLTGTVQLENGTCQSCSDLSKYPGCKTTDTCNVDSRTGYIYATECSDGFSGRSPYSNCTTCIESNYY
EDI_244250  ECACPTGTVQLENGRCKSCSELSKYEGCKTTDTCNVDAKTGYIYATECSEDFNGRSPYSNCTACTLSNYY
```

```
             841
EHI_006980  PKEGEK-------NGCAKCDDKCATCSDKDTCLTCADPLKVGSKCDGCKTGYYMSNGECKPCTNHCSECSS
EDI_276450  PKNGEKGEGNKNNGCAKCNPECGTCSDQDICLTCTDSLKVGSKCDRCKTGYYMSNGECKPCTNHCSECTS
EHI_065330  PKEGEK-------NGCAKCDDKCATCSDKDTCLTCTDPLKIGSKCDECKTGYYMSNGECKPCTNHCSECSS
EDI_244250  PKKGEKGEDGNNNGCAKCGSECATCSDEHVCLTCAKPLMVGSKCDRCKTGYYMSNGECKPCTNHCSECTS

             911
EHI_006980  AAECTVCESDTYKVISGNGCNSCVDGFYFDEIKGTCIPCTSPCTKCVGVKKDCEEQETGCNSEKKKIVEE
EDI_276450  ATECTVCESETYKVISGKGCNSCADGFYFDEIKGACIPCTSPCTKCIGVMKDCEEGESGCNSEKKKVVEH
EHI_065330  AAECTVCESDTYKVISGNGCNACVDGFYFDEIKGTCIPCTSPCTKCVGVKKDCEEQETGCNSEKKKIVEE
EDI_244250  ATECTVCESDTYKVISGKGCNSCADGFYFDEIKGACILCTSPCTKCIGVMKYCEEGESGCNSEKKKVVEH

             981
EHI_006980  CTKCSTKDHIAEVPVNGACVCAYGYVEGTSTEDNKIECQACKAKVNEFCDSCNSKDCLRCNAEYLEAKGG
EDI_276450  CTKCSTKDHISEFPLNGACTCAYGYIQNNSTKDNTIECESCKAKVNEFCDSCNSNECLKCNAEYLEVKGE
EHI_065330  CTKCSTKDHIAEVPVNGACVCAYGYVEGTSTEDNKIECQSCKAKVNEFCDSCNSKDCLRCNAEYLEAKGG
EDI_244250  CTKCSTKDHISEFPVNGACTCAYGYIQNNSTKDNTIECESCKAKVNEFCDSCNSNECLKCNAEYLELKGG

            1051
EHI_006980  ECVCVEGYYTSSWGSCIPCSRHMPHCTKCTGEGECTTCEDGWKLKDGKCNGAKGIFIMMMIVMLAFMF
EDI_276450  ECVCVEGYYTSSWGSCVPCSRHMAHCTKCSGEGKCTSCEEGWKLEEGNCNGSKGIFIMMVIVMLSFMF
EHI_065330  ECVCVEGYYTSSWGSCIPCSRHMPHCTKCTGEGECTTCEDGWKLKDGKCNGAKGIFIMMMIVMLAFMF
EDI_244250  ECVCVEGYYTTSWGSCIPCSRHMAHCTKCSGEGKCTSCEEGWKLEEGNCNGSKGIFIMMVIVMLSFMF
```
