# Supplementary material for: Evidence of Gene Conversion in Genes Encoding the Gal/GalNac Lectin Complex of Entamoeba
Source: PLoS Negl Trop Dis. 2011 Jun 28;5(6):e1209. doi: 10.1371/journal.pntd.0001209 (PMC3125142; doi:10.1371/journal.pntd.0001209)
Supplement: Figure S9 — Amino acid multiple alignment of light chain lectin ( lgl ) gene family members from E. histolytica and E. dispar , used to generate a gene phylogeny. (PDF) [file pntd.0001209.s009.pdf]

Alignment: /Users/garethweedall/Desktop/lgl_13seqs.fas
Seaview [blocks=70 fontsize=10 A4] on Fri Nov  5 15:16:36 2010

```
           1
EHI_049690 ----MIILILLISYSFGKTNDNRDQFSPNYPYGKQNQGTKFNTYFTSDVNSYQIQQFAESGVFSANQENY
EDI_071530 ----MIALFLLIVYSIGKTSDNRDQFSTNYPYGMENRNTNFNSEFTSDVNSYQIQRFAENGVFSANQENY
EHI_159870 ---MIFILCVLLVIHTNALIDEKVVV----------------------------------------QEII
EDI_325130 ---MRFILCVLLVIHINALIDEKVVV----------------------------------------QEII
EHI_058330 ----MFILFLLISVAFVNAADSSQGI-DNYPYGKKNTNANFNVAFDSSYSKYQVQQYAESGVFSANQENY
EHI_148790 ----MITLFLLIVYSIADTSDGRNQLSEKYPYGMDNRNTKFDHDFTSDVNSYQIQKFAESGVFSANQENY
EHI_183400 MIQSMIPILLLILHCNSKTTDGRDQLSQNYPFGMRNQNTKFNEYFSSAVDSYQIQQFAESGVFNANQENY
EHI_035690 ----MIILVLLISYSFGKTQDGKDQLSPNYPYGKMNKDVNFNKPFTSAVDSYQIQQYAENGVFSANQENY
EHI_027800 ---------------------------------MRNQNTKFNEYFSSAVDSYQIQQFAESGVFNANQENY
EDI_131690 ----MIALFLLIVYSIGKTSDNRDQFSTNYPYGMNNYNTNFNSEFTSDVNSYQIQRFAENGVFSANQENY
EDI_213170 ----MISLFLLISVAFVNATDSSQGV-ENYPYGKVNKQANFGKAFDSSYNSYQVQRYAESGVFSANQENY
EDI_352500 ----MISLFLLISVAFVNATDPTDIT--NYPY-EKSQYFDTNKKFTSALNSYQVQRYAERGVFSANQENY
EDI_023210 ----------------------------------------------------------------------

          71
EHI_049690 VRAKCKTCCRVIFASDYNYKTEKQFTDTDDKNGDDRYVMDMEFDDKRSVRYANGGYE-QNILLRPLKQGN
EDI_071530 VRAKCKTCCRVIFASDYNYKTQRQFTDADDINGDTRYVMDMEFDDKRSVRYPNGNYE-QNILLRPLKQGN
EHI_159870 QRDKCETCCRVLFAMEYD--PTNNFEKLKD--SSDRYVLDVEFETMSMLRIDINAYNTQQMRVRKLTTER
EDI_325130 QRDKCETCCRVLFAMEYD--PNNNFEKLKD--TSDRYVLDVEFETLSMLRIDINAYNTQQMRVRKLSTER
EHI_058330 VRAKCKTCCRVIFASDYNYKTKKPFTDADDIRGDDRYVMDMEFDDKRSVRFYQGNYE-QNILLRPLKQEN
EHI_148790 VRAKCKTCCRVIFASDYNYETQKQFTTDDDVKGTTRYVMDMEFDDKRSVRFYQGNYE-QNILLRPLKMGN
EHI_183400 VREKCKTCCRVIFASDYNYKTEKQFTDEDDKNETPRYVMDMEFDDKRSVRYPDGNYE-QNVLLRPLKQGN
EHI_035690 VRAKCKTCCRVIFASDYNYKTNTQFTDEDDKKGDERYVMDMEFDDKRSVRFRNGGYE-QNILLRPLKQGN
EHI_027800 VREKCKTCCRVIFASDYNYKTEKQFTDEDDKNETPRYVMDMEFDDKRSVRYPDGNYE-QNVLLRPLKQGN
EDI_131690 VRAKCKTCCRVIFASDYNYKTQRQFTDADDINGDTRYVMDMEFDDKRSVRYPNGNYE-QNILLRPLKQGN
EDI_213170 VRAKCKTCCRVIFASDYNYKTKKQFTNADDVRGDTRYVMDMEFDDKRSVRYYQGQYE-QNILLRPLKMGN
EDI_352500 VRAKCKTCCRVIFASDYNYETGKKFTDADDVRGDTRYVMDMEFDNKNSLRMYQGDYE-QNILLRPLKMGN
EDI_023210 ----------------------------------------------------------------------

         141
EHI_049690 ELQFFEFAPYRMYTSYAIPKRVHDIRAGAFEGHSLIIWSKNPPLSDAPGTKNQRFVYVHPYS--------
EDI_071530 ELQFFEFAPYRMYTCYSMPKRVHDIRGGANEGSTLIIWSKNPPLSDAPGTQNQRFVYVHPYP--------
EHI_159870 ELQYWEFASYQMICLYSYPNRVMDMLYNTKFGNPLIIWRRKEPL--LESTNNQRFVYIYDYPFTDSRHKP
EDI_325130 ELQYWEFASYQMICLYSYPNRVMDMLYNTKFETPLIIWRRKEPL--LETTNNQRFVYIYDYPFTDSRHKP
EHI_058330 ELQFFEFAPYKMYTSFSIPRRVHDIRGGANKGATLIIWMKKAPL--DPGTNNQRFVYVHPYK--------
EHI_148790 ELQFFEFAPYKMYTSFAIPRRVHDIRGGAIRGATLIIWMKKAPL--DPGTNNQRFVYVHPYP--------
EHI_183400 ELQFFEFAPYRMYTY-------------------------------APGTRNQRFVYVHPYP--------
EHI_035690 ELQFFEFAPYRMYTSYAIPKRVHDIRGGANEGATLIIWPKNPPLSDAPGTRNQRFVYVHPYP--------
EHI_027800 ELQFFEFAPYRMYTCYAIPKRVHDIRAGAVEGHTLIIWSKNPPLSDAPGTRNQRFVYVHPYP--------
EDI_131690 ELQFFEFAPYRMYTCYSMPKRVHDIRGGANEGSTLIIWSKNPPLSDAPGTQNQRFVYVHPYP--------
EDI_213170 ELQFFEFAPYKMYTSFSIPRRVHDIRGGANRGATLIIWMKKAPL--DPGTNNQRFVYVHPYK--------
EDI_352500 ELQFFEFAPYKMYTSFSIPRRVHDIRGGANKGATLIIWMKKAPL--DPGTNNQRFVYVHPYP--------
EDI_023210 -----------MYTCYSMPKRVHDIRGGANEGSTLIIWSKNPPLSDAPGTQNQRFVYVHPYP--------

         211
EHI_049690 --TDWYPEYHSKVKY-NNKGRVVEKTLEWPTYKRHFYLPYRLDVDLCYQARKKNEAKEKWTGNNHLNTTS
EDI_071530 --NEWYAEYHSTIEY-NQGGRWVKKTLEWPTYKRHFYLPYRLDLDLCYQAKKASDSRSVWTGNQHLKTLT
EHI_159870 YVKAFIAEYYVPSDC-SNWWYCNEHQISWD-YNRYAFK------EMCYEV--DDDNVKAYCGSAYNECNV
EDI_325130 YVKAFIAEYYVPSGC-SDWGYCYEHQINLN-YNRYAFK------EMCYEV--DDDNVRAFCGSDYHECTT
EHI_058330 --TEWYPEY--------------NNRNKWPSYSKHFYLPFSNN-NLCYQAKSEGEERSTWTGNRHLTTTT
EHI_148790 --TSYYQN---------------SNQNKWKDYPKHFYLPFSNN-NLCYQAKSEGEERSTWTGNAHLK-LA
EHI_183400 --DSWYPEYHTVIKYRNSRGALVDKKLEWPTYKRHFYLPYRLDVDLCYQAKSAADIPSKWYGNRHLNTIG
EHI_035690 --TEWYPEYNSTTKY-TQNGKTVIKTLKWPTYKRHFYLPYRLDVDLCYQARKATDGRSTWTGNKNLNTTS
EHI_027800 --DSWYPEYHTVIKYRNSRGALVDKKLEWPTYKRHFYLPYRLDVDLCYQAKSAADIPSKWYGNRHLNTIG
EDI_131690 --NEWYAEYHSTIEY-NQGGRWVKKTLEWPTYKRHFYLPYRLDLDLCYQAKKASDGRSVWTGNQHLKTLT
EDI_213170 --TEWYLEY--------------SKTNKWPSYSKHFYLPFSSN-NLCYQAKSEGQQRSTWTGNAHLTTTS
EDI_352500 --TSFYPEY--------------SKTNKWPSYSKHFYLPFSSN-NLCYEAKKKTDGRSTWTGNAHLTTTS
EDI_023210 --NEWYAEYHSTIEY-NQGGRWVKKTLEWPTYKRHFYLPYRLDLDLCYQAKKASDGRSVWTGNQHLKTLT

         281
EHI_049690 ISY--QIIASKCNATEP-----------RQVFIPVFA
EDI_071530 NGY--QITASRCSATES-----------RQIFIPVFA
EHI_159870 DGYYKGLKAQDCVNEKQSTGA-SLMHAKKQKFIPIFA
EDI_325130 DGYYKGLKARDCVDEKQSSSTDSLIHAKKQKFIPIFA
EHI_058330 TSY--QIEAASCDAGEA-----------RQIFIPVFA
EHI_148790 NSY--QIEAASCVANEP-----------RQIFIPVFA
EHI_183400 DSY--QITASVCNAKEP-----------RQIFIPVFA
EHI_035690 KSY--QIIASRCSATEA-----------RQIFIPVFA
EHI_027800 DSY--QITASVCNAKEP-----------RQIFIPVFA
EDI_131690 NGY--QITASRCSATES-----------RQIFIPVFA
EDI_213170 TSY--QIEAASCVANEP-----------RQIFIPVFA
EDI_352500 TSY--QIAAAMCNPSEP-----------RQMFVPVFA
EDI_023210 NGY--QITASRCSATES-----------RQIFIPVFA
```
