# Supplementary material for: Evidence of Gene Conversion in Genes Encoding the Gal/GalNac Lectin Complex of Entamoeba
Source: PLoS Negl Trop Dis. 2011 Jun 28;5(6):e1209. doi: 10.1371/journal.pntd.0001209 (PMC3125142; doi:10.1371/journal.pntd.0001209)
Supplement: Figure S10 — Nucleotide multiple alignments of intermediate chain ( igl ) lectin gene family members from E. histolytica and E. dispar , used for sequence similarity plot. (PDF) [file pntd.0001209.s010.pdf]

Alignment: /Users/garethweedall/Desktop/igl_4seqs.fas
Seaview [blocks=70 fontsize=10 A4] on Fri Nov 5 15:17:11 2010

```
             1
EHI_006980   ATGTTTATTCTTCTTTTATTCATATCAATTTCACTTGGTGATTATACTGCTGATAAGCTCATAGGCGGAA
EDI_276450   ATGTTTATTATTCTTTTATTCATATCAATTTCACTTGGTGAGTACAAAGCTGATAAACTTATTAAAGGCC
EHI_065330   ATGTTTATTCTTCTTTTATTCATATCAATTTCACTTGGTGATTATACTGCTGATAAACTCATTAATAACC
EDI_244250   ATGTTTATTCTTCTTTTATTTATATCAATTTCACTTGGTGATTACAAAGCTGATAAACTCATCGGAGACA

            71
EHI_006980   AAGAGCCAAGAGAGGCTGTTCCACATTGTGCATCAGTTTCAAATGGAGCATGCACTAGTTGTGATACTGG
EDI_276450   AAGAACCAAGAACTGCTGTTCCACACTGTGCATCAGTTTCAAATGGGGCATGTACTAGTTGTGATGTTGG
EHI_065330   AAGAACCAAGAACTGCTGTTCCACATTGTGCATCAGTCTCAAATGGAGCATGCGCTAGTTGTGATGAAGG
EDI_244250   AAGAACCAAGAGAGGCTGTTCCACATTGCGCATCAGTTTCAAATGGAGCATGTGCTAGTTGTGATGATGG

           141
EHI_006980   TTATGAACTTACTACC---------ACTGGAAATAATAAGACATGTACTCTTAAAGAAGATATGTGTAAA
EDI_276450   TTATGAACTTTCTTCTGAT------TCTTCAAATACCCAAAAATGTACTCTTAAACAGGACATTTGTAAA
EHI_065330   TTATGAACTTAAAACTGAGTCTGGAAGTGGAAGTACTCAAAAATGTACTCTTAAAGAAGAAACTTGTAAA
EDI_244250   CTATGAACTTAAAACTGAA------TCTGGA---AGTAATAAGTGTGTCCTTAAAGAAGGTACTTGTAAA

           211
EHI_006980   ACTGCTTTTTCTTATTATGATAAAACAAACTCTACAAACCCCAAATGTACTTATTGTGTTAACGGTAAAG
EDI_276450   ACTACATTTTCTTACTATGATAACAGTAATGCTAGTTCTCCAAAATGTGTTTATTGTGAAAATGGAAAAG
EHI_065330   AGTGCTTTTTCTTATTATGACGGTAGTGATTCTAATTCTCCAAAATGTGTTTATTGTGAAAATGGAAAAG
EDI_244250   AGTTCATTTTCTTACTATGACACCAGTAACTCTGAAACTCCTAAATGTACTTATTGTGAAGATGGAAAAG

           281
EHI_006980   AAGTAAATACATCATCACACTCTGGAAATGATAAGTGTGTATGCAAAAACAATGTAAACATTTGTGAGTC
EDI_276450   AAGCCAATACACCACCAAACTCTAATTCTGAAAAATGTACATGTAAAAATGGTGTTGATAATTGTGATAC
EHI_065330   AAAGTGATACATCATCT---TCTAATAATGAGAAATGCAAATGTAAGAATGGTGTTGATACTTGTGAGTC
EDI_244250   AAGTTGATGATTCATCTACCTCTAGTACTGATAAATGTACATGTAGAAATAGTGTTAGCACTTGTGATAC

           351
EHI_006980   ATGTCTTTTGATGAAA---GATTCAAAATGTGGAGAATGTATAATTGGGATGTCTACTACTGTTGAT---
EDI_276450   TTGTCTTTCTAAAGACAGTGGCACTAAATGTGAAGAATGTATAATTGGAATGTCTACAACTAACACTGGA
EHI_065330   ATGTCTTTCAAAAGAC---AACGATAAATGTGGTGAATGTGTTATTGGAATGTCTACTACTACTAATGGA
EDI_244250   TTGTCTTTATATGGGACAAGATAATAAATGTGGTGAATGTGTTATAGGAAAATATTCAACCACA------

           421
EHI_006980   GGTTCAAAGTTATGTGATAATGCAACTACAGAAGATCATGCAGAAAATTGTGTTGGTCTTTTAGCCTCTT
EDI_276450   AGTTCAAAAGGATGCGACAATGCAACTACAGATGACCATGCAGAAAATTGTATCGGTCTTTTAGCTTCAA
EHI_065330   GGTCAGAAGTTATGTGATACAGTAACTACAGACGAACATGCAGAAAATTGTGTTGGTCTTACCGCAAAAG
EDI_244250   GGAGAAAAGTTATGTGACAATGCAATTGCAGATGACCATGCAGAAAATTGTATCGGTCTTTCAGCTAAAG

           491
EHI_006980   CTACTTCTTCAAAGACTTGTGATAAATGTTTCGGTATGTACTCTCTTCAAGGTGGGAAATGTACTCAAAA
EDI_276450   CCACTTCTTCAAAGACATGTGACAAATGTTTTGGAAATTATACACTTGAAAATGGTAAATGTACTAAAAA
EHI_065330   ATTCATCTTCAAAACAATGTGATAAATGTTTTGGTATGTATTCTCTTCAAAGTGGTCAATGTACTAAAAA
EDI_244250   ATTCTTCAAACAAGGCATGTGATAAATGTTTTGGAAGTTATACCCTTCAAAATAATATGTGTTCTAATCA

           561
EHI_006980   GAATGATAAAATTAATAAATGTATTTTACAAGTTGAAAACTCTTGTAACCAATGTGCAGATGGATATTCT
EDI_276450   GAATGAAAAGATTAGTAATTGTATTTTACAAGTAGTAAATGATTGTAACCAATGTGCAGATGGATATTAT
EHI_065330   GAATGAAAAGATTGAAAAGTGTATTTTACAAGTTGAAAGCTCTTGTAACCAATGTGCAGATGGATATTAT
EDI_244250   ACTCACTAAAATTGATAAGTGTATTTTACAAGTAGAAAATTCTTGTAATCAATGTGCAGATGGATATTAT

           631
EHI_006980   CTCAGTACTGATAAGAAATCTTGTAATAAGTTCCCGGAGCATTGCTCAAAGATTAATGGTAATCAATGCT
EDI_276450   ATTAATGCTGAGAAAAAA---TGTACCAAATATCCAGACCATTGTTCTAAAATGAATGGTAATCAATGCT
EHI_065330   ATTAATACTGAAAAAAAA---TGTACTAAATACCCAGATCATTGTTCAAAGATGAATTCTGATAAGTGTA
EDI_244250   ATTAATGCTGAGAAAAAA---TGTACCAAATATCCAGACCATTGTTCTAAAGTAAATGGTGAGAAATGTG

           701
EHI_006980   TGACATGTATGGAAGGTTATTATTTAAGTAAAACAGATTCTAAATGTACTATATGTACTGTTGATAATCC
EDI_276450   CGACCTGCATGGAAGGCTATTATTTAAAAGACTCTAAA------TGTAATGTATGTACAATTGATAATCC
EHI_065330   ATGGTTGTATGGAAGGTTATTATTTAAATGGTACAGAA------TGTAAGGTATGTACAATTGATAACTC
EDI_244250   CTTCATGTATGGAAGGTTATTATTTAACTGGTACAGAG------TGCGAAGTATGTACAATTGATAATCT

           771
EHI_006980   AAATAATCTTTCAGAAGGTAACGAATGTAGTATTTATAACGCTGAACATTGCACATCATGTAATAAAAGA
EDI_276450   AAACAATCTTTCAGAAGGTAATGAATGTAGTATTTATAATACTGAACATTGTACATCATGTAATAAAAGA
EHI_065330   AAAAGATCTCTCAGAAGGTAACGAGTGTAGTATTTATAACGCTGAACATTGTGAATCATGTAATAAAAGA
EDI_244250   AGACGATCTTTCAAAAGGTGATGAATGTAGTATTTATAGTGCTAAACACTGTACATCATGTAATAAAAGA
```

```
                841
EHI_006980  TGTACTGTTTCTGATGGAGTTTGTGTCAAGAATCATTGTCGTTTATTCTCACCAACAGAAGAAAATAAAT
EDI_276450  TGTACTGTTTCTGGTGGATTTTGTACTAAGAATCATTGTCGTTTGTTCTCTTTGACTGAAGAAAATAAAT
EHI_065330  TGTACTGTTTCTGATGGAGTTTGTGTCAAGAATCATTGTCGTTTATTCTCACCAACAGAAGAAAATAAAT
EDI_244250  TGTACTGTTTCTGGTGAAGTTTGTGTTAAGAATCATTGTCGTTTGTTCTCTTTGACTGATAGTAGTAAAT

                911
EHI_006980  GTACAAAATGTGATAATGGATATTTCTTAACAACTTCAGGAACATGTTCACCAAATTTGTATGATGGTTT
EDI_276450  GTGCAAAATGTGATAATGGATATTTCCTAACAACTGAAGGAAAATGTTCACCAAATTTGTACGATGGTTT
EHI_065330  GTACAAAATGTGATGATGGATATTTCTTAACAGGTGCAGGAAAGTGTTCACCAAATTTAAATGATGGTTT
EDI_244250  GTGCAAAATGTGATAGTGGATATTTCCTTACTAGCTCTGGAACTTGTTCACCAAATTTCTATGATGGTTT

                981
EHI_006980  CAAAACAGCTAATAGAACAGAATGTGAAAATGGCTATTATTTAGAAAAAGATGGTGATAAAAAGAGATGT
EDI_276450  CACAACATCAGCTAAAACAGAATGCCAACCAGGCTATTATTTAGAAAAAGATGGAGAAAATAAGAGATGC
EHI_065330  CAAAACATCTGCAAAGACAGAATGTCAAAAAGGTTATTATTTAGAAAAAGATGGAGATAAAAAGAGATGT
EDI_244250  CAAAACATCAGCTAAAGAAGAATGTTTACCTGGTTATTATTTAGAAAAAGATGGAGAAAATAAGAGATGT

               1051
EHI_006980  TCACTTTGTCCAGATCCATTTACTGAATGTCTTACTTCTAAAACACCAGTTCCAGGTAAGTTAAATCTTA
EDI_276450  TCACTTTGTCCAGATCCATTTACTGAATGTCTTACTTCTAAAACACCTGTTCCAGGTAAATTAAATATTA
EHI_065330  TCACTTTGTCCAGATCCATTTACTGAATGTCTTACTTCCCAAACACCAGTTCCAGGTAAGTTAAATCTTA
EDI_244250  ACACTTTGTCCAGATCCATTTACTGAATGTCTTACTTCTAAAACACCTGTTCCAGGTAAATTAAATCTTA

               1121
EHI_006980  GAAGCTCACACTTAACATCAACTGATGGACCATGCAAACTTCCAGGATGTTTATTATGTAGTGATGATGA
EDI_276450  AAAATTCTCACTTAACTTCAACAATTGGACCATGTAAACTTCCAGGATGTTTGTCATGTAGTGATGATGA
EHI_065330  GAAGTGCACACTTAACATCAACTGATGGGCCATGCAAACTTCCAGGATGTTTATTATGTAGTGATGATGA
EDI_244250  GAAGTTCTCATTTAACATCAAATGCGGGTCCATGCAAACTTCCAGGATGTTTGTCATGTAGTGATGATGA

               1191
EHI_006980  TACTATTTGTTATAAATGTGAGAATGGACTTACATTGAATGGAACTCATTGCTATAATTTTGACACTAAA
EDI_276450  TACTATTTGTTATAAATGTGAGAATGGACTTACACTAAAAGGAACTCATTGTTACAATACTATTATTAAT
EHI_065330  TACTATTTGTTATAAATGTGAGAATGGACTTACATTGAATGGAACTCATTGCTATAATTTTGATGTTAAA
EDI_244250  TACTATTTGTTATAAATGCGATGAAGGACTTACTTTAAGAGGAACCCATTGTTACAACCTAGACTCTGTT

               1261
EHI_006980  TCAGTCCTTGGTACTAGTGGTAACAACCATCAAGTGTGTAAGATGAGAGGATATGATCAATATGAACAAT
EDI_276450  GATGTACTTGGTATTAGTGGTAAAAATCATAAAGTGTGTAAAATGAGAGGGTACAATCAATTTGAACAAT
EHI_065330  AAAGTCCTTGGTACTAGTGGTAACAACCATCAAGTGTGTAAGATGAGAGGATATGATCAATATGAACAAT
EDI_244250  AATGTACTTGGTACTAGTGGTGAAGAGCATAGAGTGTGTAAAATGAGAGGGTACAATCAATTTGAACAAT

               1331
EHI_006980  ATTTGAATGCATTTAAAGCATCTGATAATACTTATTATTGTCCACTTAAAGACCTTTATTTACCATATTA
EDI_276450  ATTTGAATGCATTTAAAGCATCAGATAATACTTATTATTGTCCACTTACAGATCTTTTCCTACCTTACTA
EHI_065330  ATTTGAATGCATTTAAAGCATCTGATAATACTTATTATTGTCCACTTAAGGATCTTTATTTACCATATTA
EDI_244250  ATTTGAATGCATTTAAAGCATCAGATGATACTTATTATTGCCCACTTACAGATCTTTTCTTACCATATTA

               1401
EHI_006980  TTTCAGTGTTACTAAAGGTACTTCAGAT---AATACAATTACTATTGGTTGTGTTGGTCAATTAAGAAAT
EDI_276450  TTTTAATGTCACTAAGAACTCTAAAGATATGTCTAAAATTACCATTGGTTGTGTTGGTAAGTCTAGAGAT
EHI_065330  TTTCAGTGTTACTAAAGGTTCAGATAAT------AAAATTACTATTGGCTGTGTTGGTAAGGATAGAGAT
EDI_244250  TTTCAGTGTTACTAAAGATAGTTCAAAT------GCAATTACTATTGGTTGTGTTGGTCAATTAAGAAAT

               1471
EHI_006980  GTTTCAAATGACTGTGAATGTAATGACAAACATATTCCAACATCAATTGACAAAGCATCAGATTGTGTTT
EDI_276450  GTTAAGAATGATTGTGAATGTGAAGCAAAATACATTCCAACATCTATAGATAAATCATCAGATTGTGTTT
EHI_065330  GTTAAGAATGATTGTGAATGTAATGACAAATATATTCCAAAGTCAGTTGACAAAGCATCAGACTGTGTTT
EDI_244250  GTTTCAAATGATTGTGAATGTAATGATAAACACGTTCCAACATCTATAGATAAATCATCAGATTGTGTTT

               1541
EHI_006980  CAATAACAACCAAACTTCCATCATGTGAAAGAACAGCAAATGGAAATATTTGTACACAATGTCCAGTTGG
EDI_276450  CAATAGCAACTAAACTTCCATCATGTGAAAGAGCAGCAAATGAAAATATTTGTACACAATGTCCAGTTGG
EHI_065330  CAATCAAAACAAAACTTCCATCATGTGAAAGAGCAGCCAATGAAAACATTTGTACACAATGTCCAGTTGG
EDI_244250  CAATAGTAACTAAACTTCCATCATGTGAAAGAGCAGCAAATGAAAATATTTGTACACAATGTCCAGTTGG

               1611
EHI_006980  ATCACATGTAGGAAAGGATGGTAAATGTTCTTGTGGTGATGCACATTATTTTGACAAAGATAATGTCTGT
EDI_276450  ATCACATGTAGGAAGTGATGGTAAATGTTCTTGTGGTGATGGTCATTATTTTGACAAAGACAATACTTGT
EHI_065330  ATCACATGTAGATAGTAATGGTAAATGTTCTTGTGGTGATGCACATTATTTTGATCAAAACAATAAATGT
EDI_244250  ATCACATGTAGGAAGTGATGGTAAATGTTCTTGTGGTGATGGTCATTATTTCGATGAAAATAATGTATGC

               1681
EHI_006980  AAAAAGTGTCCAGCTAGTTGTTCAAGTTGCTCTTATGATAGTTCTAAAAGTAAAGTTGTGTGTAGTGAAT
EDI_276450  AAAAAGTGTCCAGATAGCTGTTCAAGTTGTGCTCTTGATTCAACTAAAAATAATGTCATATGTACTTCAT
EHI_065330  CAAGAATGTCCAGCTAGTTGTTCTAGTTGCTCTTATGATAGTTCTAAAAGTAAAGTTGTATGTAGTGAAT
EDI_244250  CAGAAATGTCCAGATAGTTGTTCAAGTTGTTCTTATGATTCAAATAAAAAGGCTGTTGTATGTACTGCAT
```

```
            1751
EHI_006980  GTTATGAAAATATTCAAGGTGTTACTACAAGAAATAAAGAAAATGAATGCGCTTGCATAAATGAT-----
EDI_276450  GTTATGAAAATATTCAAGGAGTTACTACAAGGGATAAAGACAAAAAATGTGCATGTGTTAGTAAT-----
EHI_065330  GTTATGAAAATATTCAAGGTGTCTCTACAAGAGATAAAGATAATGAATGCGCTTGCAAAAAGGATACTCC
EDI_244250  GTTATGAAAATATTCAAGGAGTTACTACAAGGGATAAAAACAGTCAATGTGCTTGCCTTGATGAA-----

            1821
EHI_006980  -GGTTATAAAGAAGGACCAAATGCAGAAGATAAGAAGAAAAGTTGTGCACAACTAAATAATAATTGTAAA
EDI_276450  -GATTATAAAGAAGGACCAAATGAAGAAGATAAGAAGAAAAGTTGTGCACAATTAAATAAAAACTGTAAA
EHI_065330  TGAGTATAAAGAAGGACTAAATGCAGAAGATAAGAAGAAAAGTTGTGCACAACTAAATAATAATTGCAAA
EDI_244250  -CGCTATAAAGAAGGACCAAATGAAGAAGATAAGAAGAAAAGTTGTGCAGAGTTAAATGAAAACTGTAAT

            1891
EHI_006980  AAGGAAGGTAAATATGAAATTAGTGATGGATTTGTTACCTGTCTTGACTGTGATGACTCAGCTTATATTG
EDI_276450  AATGAAGGTAAGTATGAGATTAGTGATGGATATGTTACATGTCTTGATTGTGATAATCCTGCTTATATCG
EHI_065330  GAAGAAGGTCACTATAAGATTAGTGATGGATTTATTACATGTCTTGAATGTGATGACTCAGCTTATATTG
EDI_244250  CAAGAAGGTAAGTATGAGATTAGTAATGGATATGTTAAATGTCTTGAATGTAATGATCCTGCATATATAG

            1961
EHI_006980  TTGGTTCACAGGTTGGTGCTTGTACGCAATGTTCTCCTAATGCTTTTAAAGATGAAAATAATAAATGCCA
EDI_276450  TTGGTTCACAAATTAGTGCTTGTACTCAATGTTCTCCTAATGCTTATAGAAAT---GGAAATGAGTGTGT
EHI_065330  TTGATTCACAAACAAAGGAGTGTGCCCAGTGTGCTTCTAATGCTTTTAAAGATGAAAATAATAAATGCCA
EDI_244250  TTGGTTCTGAAATTAATGCTTGTAGTCAATGTTCTTCTAGTGCTTTTAAAGATAGTAATAATAAATGTCA

            2031
EHI_006980  ACTTTGTTCTACTAAACAATCTCAATATGGACATTGTGCAGCATGTTCAGCAACAGCATGTATTACCTGT
EDI_276450  TCTTTGTTCTACTAAACAAGCTCAGTTTGGACATTGTTCATCTTGTTCAGCAACGGCATGTCTTACATGT
EHI_065330  ACTTTGTTCTACTAAAAAGGACAAATATGGACATTGTTCAGCATGTTCAGCAACAGCATGTATTATATGT
EDI_244250  GTTATGTTCTACTAAACAAGATAAGTTTGGACATTGTTCATCTTGTTCAGCAACAGCATGTATTACATGT

            2101
EHI_006980  GAAGACATTAACTTAATACTTACCGGAGAAAAGCCA---------TGTACTGTATGTAAAGATGGATTTT
EDI_276450  GAAGATAATAATTTGATTCTTACTGCTAGTGGCTCAAATGTACAATGTACTGAGTGTAAAGATGGATTTT
EHI_065330  GAAGACACTAATTTAGTTCTTGCTGCTAGTGGTTCAAATGCACAATGTACTGTATGTAAAGATGGATTTT
EDI_244250  GAAGATAATAATTTAATTCTTGCTGCTAGTGACTCAAATGTACAATGTACTGAGTGTAAAGATGGATTTT

            2171
EHI_006980  ATCAAATTGAAAATGCAACAGATGGAGTGTATTGTAGTCCATGTCCTGCAAAATGTAAAACATGTAAATA
EDI_276450  ATAAAATTGAAAACCCAACGGATGGAGTGTATTGTAGTCCATGTCCTGCAAAGTGCAAAACATGTAAATA
EHI_065330  ATCAAATTGAAAGTCCAACAGATGGAGTGTATTGTAGTCCATGTCCTGCAAAATGTAAAACATGTAAATA
EDI_244250  ATAAAATTGAAAACCCAACGGATGGAGTGTATTGTAGTCCATGTCCTGCAAAATGTAAAACATGTAAATA

            2241
EHI_006980  TAATACCACTTCAAAGAAAGTTGAATGTGTGACATGCACTGAACAAAGGCTAAAAGATATTAAAGCACCA
EDI_276450  TAATACCCCAACGAAGAAAATTGAATGTTTGACATGTACTGATACAACTTCTCAAGACATTAAAGCACCA
EHI_065330  TAGTGCCGATAAAAAGGAGATTGAATGTGTGACATGCACTGACCAAAGTTCTGTAGACATTAAACCACCA
EDI_244250  TGATAGTACTAAACAGGAGGTTGAGTGTTTGACATGTACTGATACAACTTCTCAAGACATTAAAGCACCA

            2311
EHI_006980  GAATGTGCTTGTCCAACAGGAACAGTTCAACTTGAAAATGGAACGTGTCAAAGTTGCTCTGACCTTTCAA
EDI_276450  GAATGTGCTTGTCCAAAAGAAACAGTTCAACTTGAGAATGGAAGATGTAAGAGTTGTTCAGAACTTTCAA
EHI_065330  ACATGTGCTTGTCTAACAGGAACAGTTCAACTTGAAAATGGAACGTGTCAAAGTTGCTCTGACCTTTCAA
EDI_244250  GAATGTGCTTGTCCAACAGGAACAGTTCAACTTGAAAATGGAAGATGTAAGAGTTGTTCAGAACTTTCAA

            2381
EHI_006980  AATATCCAGGATGTAAAAAAACTGATTCATGTAATGTTGATAGTAGAACAGGATTTATCTATGCAACAGA
EDI_276450  AATATGAAGGATGTAAAACTACTGATACATGTAATGTTGATGCTAAAACAGGATATATTTATGCAACAGA
EHI_065330  AATATCCAGGATGTAAAACTACTGACACATGTAATGTTGATAGTAGAACAGGATATATCTATGCAACAGA
EDI_244250  AATATGAAGGATGTAAAACTACTGATACATGTAATGTTGATGCTAAAACAGGATATATTTATGCAACAGA

            2451
EHI_006980  ATGTTCAGATGGTTTTAGTGGACGTAGTCCTTATAGTAATTGTACTACATGTACTAAGTCTAATTATTAT
EDI_276450  ATGTTCAGAAAATTTTAATGGACGTAGTCCTTATAGCAATTGTACTGCATGTACTTTGTCTAACTATTAT
EHI_065330  ATGTTCAGATGGTTTTAGTGGACGTAGTCCTTATAGTAATTGTACTACATGTATTGAGTCTAATTATTAT
EDI_244250  ATGTTCAGAAGATTTTAATGGACGTAGTCCTTATAGCAATTGTACTGCATGTACTTTGTCTAACTATTAT

            2521
EHI_006980  CCAAAAGAAGGAGAAAAG------------------AATGGGTGTGCTAAATGTGATGATAAATGTGCAA
EDI_276450  CCAAAAAATGGAGAAAAGGGAGAAGGTAATAAAAATAATGGATGTGCTAAATGTAATCCTGAATGTGGGA
EHI_065330  CCAAAAGAAGGAGAAAAG------------------AATGGGTGTGCTAAATGTGATGATAAATGTGCAA
EDI_244250  CCAAAAAAAGGAGAAAAGGGAGAGGATGGAAATAATAATGGATGTGCCAAATGTGGTAGTGAATGTGCAA

            2591
EHI_006980  CATGTTCAGATAAAGACACTTGTTTAACATGTGCTGATCCATTAAAGGTAGGAAGTAAATGTGATGGATG
EDI_276450  CTTGTTCAGATCAAGACATCTGTCTAACATGTACTGATTCATTAAAAGTAGGAAGTAAGTGTGATAGATG
EHI_065330  CATGTTCAGATAAAGACACTTGTTTAACATGTACTGATCCATTAAAGATAGGAAGTAAATGTGATGAATG
EDI_244250  CTTGTTCAGACGAACATGTTTGTTTAACATGTGCTAAACCATTAATGGTAGGAAGTAAGTGTGATAGATG
```

```
            2661
EHI_006980  TAAAACAGGTTATTATATGTCAAATGGTGAATGTAAGCCATGTACCAATCATTGTAGTGAATGTAGTAGT
EDI_276450  TAAGACAGGATATTATATGTCTAATGGAGAATGTAAACCATGTACTAATCATTGTAGTGAATGTACTAGT
EHI_065330  TAAAACAGGCTATTATATGTCAAATGGTGAATGTAAGCCATGTACCAATCATTGTAGTGAATGTAGTAGT
EDI_244250  TAAGACAGGATATTATATGTCTAATGGAGAATGTAAACCATGTACTAATCATTGTAGTGAATGTACTAGT

            2731
EHI_006980  GCCGCAGAATGTACAGTATGTGAGAGTGATACATACAAGGTAATTAGTGGAAATGGATGTAATTCATGTG
EDI_276450  GCTACAGAATGTACAGTGTGTGAGAGCGAAACATATAAGGTAATTAGTGGAAAAGGATGTAATTCATGTG
EHI_065330  GCCGCAGAATGTACAGTATGTGAGAGTGATACATACAAGGTAATTAGTGGAAATGGATGTAATGCATGTG
EDI_244250  GCTACAGAATGTACAGTGTGTGAGAGCGATACATATAAGGTAATTAGTGGAAAAGGATGTAATTCATGTG

            2801
EHI_006980  TAGATGGATTCTATTTTGATGAGATTAAAGGAACATGTATACCATGCACATCACCATGCACTAAATGTGT
EDI_276450  CAGATGGATTCTATTTTGATGAAATTAAAGGAGCATGTATACCATGTACATCACCATGCACTAAATGTAT
EHI_065330  TAGATGGATTCTATTTTGATGAGATTAAAGGAACATGTATACCATGCACATCACCATGCACTAAATGTGT
EDI_244250  CAGATGGATTCTATTTTGATGAAATTAAAGGAGCATGTATACTATGTACATCACCATGCACTAAATGTAT

            2871
EHI_006980  TGGAGTAAAGAAAGATTGTGAAGAACAAGAAACAGGATGTAATTCTGAGAAGAAGAAAATAGTAGAAGAA
EDI_276450  TGGAGTAATGAAGGACTGTGAAGAAGGAGAAAGTGGATGTAATTCAGAGAAGAAGAAAGTAGTAGAACAT
EHI_065330  TGGAGTAAAGAAAGATTGTGAAGAACAAGAAACAGGATGTAATTCTGAGAAGAAGAAAATAGTAGAAGAA
EDI_244250  TGGAGTAATGAAGTACTGTGAAGAAGGAGAAAGTGGATGTAATTCAGAGAAGAAGAAAGTAGTAGAACAT

            2941
EHI_006980  TGTACTAAATGTTCAACAAAAGATCATATTGCTGAAGTACCAGTGAATGGAGCATGTGTTTGTGCATATG
EDI_276450  TGTACAAAATGTTCAACAAAAGATCATATTTCAGAATTCCCATTGAATGGGGCATGTACATGTGCATATG
EHI_065330  TGTACTAAATGTTCAACAAAAGATCATATTGCTGAAGTACCAGTGAATGGAGCATGTGTTTGTGCATATG
EDI_244250  TGTACAAAATGTTCAACAAAAGATCATATTTCAGAATTCCCAGTGAACGGGGCATGTACATGTGCATATG

            3011
EHI_006980  GATATGTTGAAGGCACTTCTACAGAAGATAATAAAATTGAATGTCAAGCATGCAAAGCTAAAGTTAATGA
EDI_276450  GATATATTCAAAATAATTCAACAAAAGATAATACAATAGAATGTGAATCATGTAAAGCCAAAGTTAATGA
EHI_065330  GATATGTTGAAGGCACTTCTACAGAAGATAATAAAATTGAATGTCAATCATGCAAAGCTAAAGTTAATGA
EDI_244250  GATATATTCAAAATAATTCAACAAAAGATAATACAATAGAATGTGAATCATGTAAAGCCAAAGTTAATGA

            3081
EHI_006980  GTTTTGTGATTCATGTAATTCCAAAGACTGTTTGAGATGCAATGCTGAATATTTAGAAGCAAAAGGAGGA
EDI_276450  ATTCTGTGATTCATGTAATTCAAATGAATGTTTGAAATGCAACGCAGAATATTTAGAAGTGAAAGGAGAA
EHI_065330  GTTTTGTGATTCATGTAATTCCAAAGACTGTTTGAGATGCAATGCTGAATATTTAGAAGCAAAAGGAGGA
EDI_244250  ATTCTGTGATTCATGTAATTCAAATGAATGTTTGAAATGCAATGCAGAATATTTAGAATTGAAAGGAGGA

            3151
EHI_006980  GAATGTGTATGTGTTGAAGGATATTACACATCTAGTTGGGGATCATGTATTCCATGTTCAAGACATATGC
EDI_276450  GAGTGTGTTTGTGTAGAAGGATATTACACATCAAGTTGGGGATCATGTGTTCCATGTTCAAGACATATGG
EHI_065330  GAATGTGTATGTGTTGAAGGATATTACACATCTAGTTGGGGATCATGTATTCCATGTTCAAGACATATGC
EDI_244250  GAATGTGTTTGTGTAGAAGGATATTACACAACAAGTTGGGGATCATGTATTCCATGTTCAAGACATATGG

            3221
EHI_006980  CACATTGTACTAAATGTACAGGAGAAGGCGAATGCACAACATGTGAAGATGGATGGAAATTAAAAGATGG
EDI_276450  CACATTGTACTAAATGTTCAGGAGAAGGAAAATGCACATCATGTGAAGAAGGATGGAAATTAGAAGAAGG
EHI_065330  CACATTGTACTAAATGTACAGGAGAAGGTGAATGCACAACATGTGAAGATGGATGGAAATTAAAAGATGG
EDI_244250  CACATTGTACTAAATGTTCAGGAGAAGGAAAATGCACATCATGTGAAGAAGGATGGAAATTAGAAGAAGG

            3291
EHI_006980  AAAGTGTAATGGAGCTAAAGGCATTTTCATCATGATGATGATAGTCATGTTAGCATTTATGTTC
EDI_276450  AAACTGCAATGGAAGTAAAGGAATTTTCATTATGATGGTGATAGTCATGTTATCATTTATGTTC
EHI_065330  AAAGTGTAATGGAGCTAAAGGCATTTTCATCATGATGATGATAGTCATGTTAGCATTTATGTTC
EDI_244250  AAATTGTAATGGAAGTAAAGGAATTTTCATTATGATGGTGATAGTCATGTTATCATTTATGTTC
```
